# Stereoselective Synthesis of Cyclobutanes by Contraction of Pyrrolidines

**DOI:** 10.1021/jacs.1c10175

**Published:** 2021-11-08

**Authors:** Chunngai Hui, Lukas Brieger, Carsten Strohmann, Andrey P. Antonchick

**Affiliations:** † Max Planck Institute of Molecular Physiology, Department of Chemical Biology, Otto-Hahn-Strasse 11, 44227 Dortmund, Germany; ‡ Technical University Dortmund, Faculty of Chemistry and Chemical Biology, Otto-Hahn-Strasse 6, 44221 Dortmund, Germany; § Nottingham Trent University, School of Science and Technology, Department of Chemistry and Forensics, Clifton Lane, NG11 8NS Nottingham, United Kingdom

## Abstract

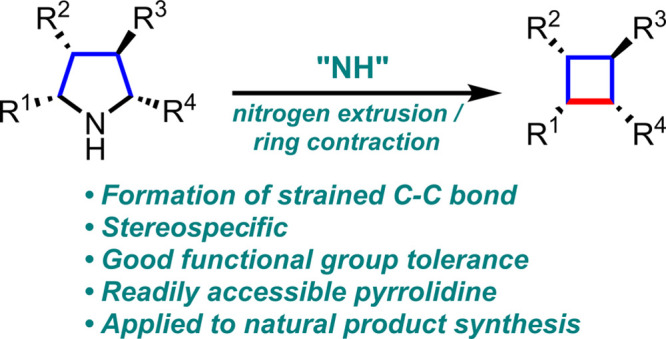

Here we report a contractive synthesis of multisubstituted cyclobutanes containing multiple stereocenters from readily accessible pyrrolidines using iodonitrene chemistry. Mediated by a nitrogen extrusion process, the stereospecific synthesis of cyclobutanes involves a radical pathway. Unprecedented unsymmetrical spirocyclobutanes were prepared successfully, and a concise, formal synthesis of the cytotoxic natural product piperarborenine B is reported.

Cyclobutanes are four-membered carbocycles which present a unique structural feature in bioactive natural products^1−3^ (Figure 1A). Many natural cyclobutanes contain various substituents and possess an array of stereocenters in a contiguous fashion in a limited space. The congested, sp^3^-enriched small carbocycle has attracted wide interest from the synthetic community due to its efficient synthesis.^4−7^ Synthetic methods directly en route to multisubstituted cyclobutanes, such as [2 + 2] cycloaddition,^8^ radical cyclization,^9,10^ and ring contraction reactions including a Wolff rearrangement,^11−13^ and oxidative pinacol rearrangement,^14^ have been well documented. In particular, many recent advances in [2 + 2] cycloaddition based on photocatalysis, organocatalysis, and Lewis acid catalysis have been reported, in which heterocoupling of two different alkenes presents a very challenging task. The development of a new synthetic method for the direct stereocontrolled preparation of substituted cyclobutanes is of great importance.

**Figure 1:**
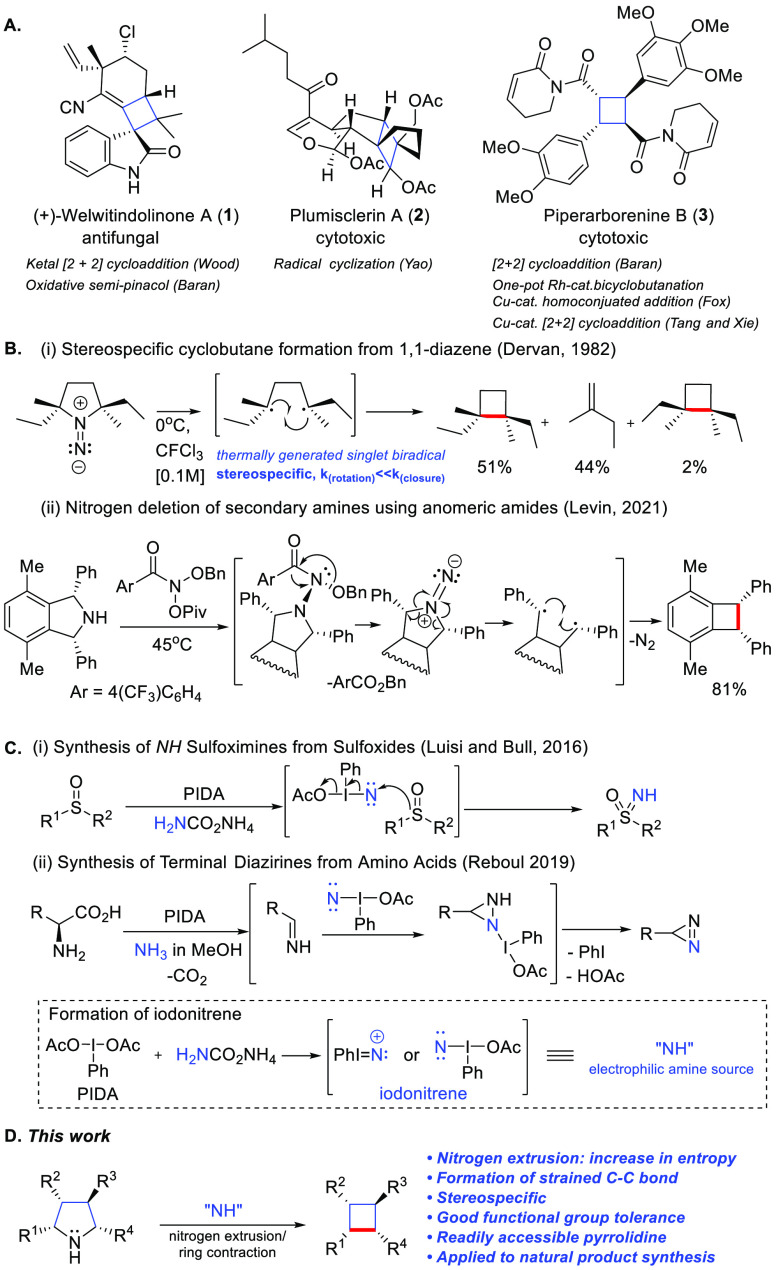
(A) Cyclobutane containing bioactive natural products. (B) Stereospecific synthesis of cyclobutanes via 1,1-diazene. (C) Iodonitrene chemistry. (D) Chemical features of this work.

Among the reported synthetic methods for cyclobutanes, the direct conversion of pyrrolidines to cyclobutanes has attracted less attention (Figure 1B). The stereospecific synthesis of cyclobutane from 1,1-diazene^15−18^ (isodiazene) derived from pyrrolidine was pioneered by Dervan’s group.^19−23^ The stereospecificity of the ring contraction is a result of the rapid C–C bond formation from the thermally generated singlet 1,4-biradical via nitrogen extrusion, affording the cyclobutanes stereoretentively. Byproducts such as the alkene resulting from β-fragmentation and a small quantity of stereoinverted cyclobutanes were also identified. In 2021, Levin’s group disclosed the nitrogen deletion of secondary amines using an *N*-anomeric amide,^24^ postulating the concept of skeletal editing of organic molecules.^25,26^ An *N*-anomeric amide acts as a nitrogen transfer reagent to a secondary amine to produce a 1,1-diazene, which generates a biradical via nitrogen extrusion, affording cyclobutane. Another nitrogen-deletion protocol of secondary amines featuring a Curtius-type rearrangement/nitrogen extrusion was reported by Lu’s group very recently.^27,28^ Electrophilic nitrogen transfer using *in situ* generated iodonitrene has been an emerging research area^29−34^ (Figure 1C).

In 2016, Luisi, Bull, and coauthors reported that the iodonitrene generated *in situ* from (diacetoxyiodo)benzene (PIDA) and ammonia or its surrogates can serve as an electrophilic amination reagent to synthesize *NH*-sulfoximines from the corresponding sulfoxides via a nitrene transfer process.^29^ Later, Reboul’s group disclosed the preparation of terminal diazirines from the corresponding amino acids.^33^ Here, PIDA serves as a decarboxylative reagent and as a source of iodonitrene, promoting electrophilic amination of the resulting imine, which gives a diazirine after oxidative cleavage of the iodine species. Inspired by these elegant synthetic methods and our long-term interest in both catalytic [3 + 2] cycloaddition reactions^35,36^ and hypervalent iodine(III) chemistry,^37−53^ we were curious about the potential outcomes of the electrophilic amination of pyrrolidines using iodonitrene, which has not been documented (Figure 1D). Here, we report a mild and efficient method for the highly stereoselective synthesis of cyclobutanes carrying diverse functionalities with different substitution patterns from readily accessible pyrrolidines using iodonitrene chemistry. A plausible mechanism is proposed with the support of experimental evidence. The developed method is applied to the formal synthesis of the cytotoxic natural product piperarborenine B (**3**).

The study was initiated by employing pyrrolidine **4** as a model substrate. A range of hypervalent iodine reagents, sources of nitrogen, and solvents were examined (Table 1; for details, see the Supporting Information). With the use of 2.5 equiv of hydroxy(tosyloxy)iodobenzene (HTIB) and 8 equiv of ammonium carbamate as an ammonia surrogate with 2,2,2-trifluoroethanol (TFE) as the solvent, pyrrolidine **4** was converted to cyclobutane **5** in 69% yield, which was unambiguously confirmed by X-ray crystallography. To gain a better understanding of the reaction system, a series of control experiments were conducted. First, TFE gave a slightly higher yield in comparison to methanol when PIDA was used with 4 equiv of ammonium carbamate (entries 2 and 3). TFE forms an H-bonded adduct with a hypervalent iodine(III) compound and the iodonitrene^54^ and enhances the reactivity of λ^3^ iodanes as a strong Lewis acid,^55^ which may have a positive effect on the nitrogen transfer to the pyrrolidine. Next, HTIB was found to be the optimal hypervalent iodine(III) reagent (entries 4 and 5). The readily leaving tosylate on HTIB is prone to displacement by nucleophiles^56,57^ and facilitates the formation of iodonitrene. 1-Hydroxy-3-oxobenziodoxole (**6**) failed to form an iodonitrene, which requires two transferable groups on λ^3^ iodine. Replacing ammonium carbamate with other N sources such as ammonium acetate^34^ failed to give **5** (entry 6). During optimization of the reagent amounts, the formation of imine **7** (32%) was observed (entry 7). This may be due to the presence of free HTIB, which oxidizes pyrrolidine to the corresponding imine^58−60^ and can be suppressed by increasing the amount of ammonium carbamate from 4 to 8 equiv (standard conditions, entry 1). Finally, a decrease in the reaction temperature from 80 to 20 °C gave **5** in 49% yield (entry 8). This observation is consistent with a previous report that iodonitrene is formed at room temperature.^29,33^ We were puzzled about the necessity of heating the reaction. It should be noted that pyrrolidine is sparingly soluble in methanol and dissolves completely upon short heating (ca. 1 min) at 80 °C. However, the pyrrolidine crystallized after standing at room temperature. We therefore suggest that the optimized reaction temperature compromises for the solubility of the starting material, iodonitrene formation, nitrene transfer, and nitrogen extrusion.

**Table 1:**
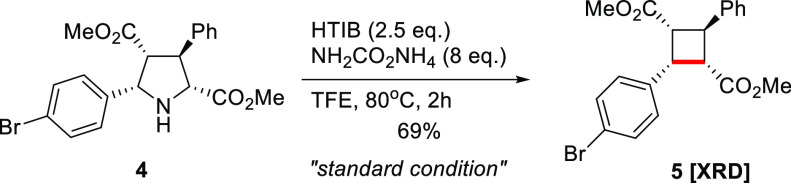
Optimization of the Reaction Conditions^a^

entry	change from the “standard conditions”	yield (%)^b^^,^^c^
**1**	**none**	**(69)**^c^
2	PIDA (3 equiv), NH_2_CO_2_NH_4_ (4 equiv), MeOH	48,^b^ (44)^c^
3	PIDA (3 equiv), NH_2_CO_2_NH_4_ (4 equiv)	54^b^
4	**6** (3 equiv), NH_2_CO_2_NH_4_ (4 equiv)	nd
5	HTIB (3 equiv), NH_2_CO_2_NH_4_ (4 equiv)	63^b^ (69)
6	HTIB (3 equiv), NH_4_OAc (4 equiv)	nd
7	HTIB (2.5 equiv), NH_2_CO_2_NH_4_ (4 equiv)	(32)^c^ + **7** (38)^c^
8	20 °C instead of 80 °C	(49)^c^

a All reactions were run on a 0.1 mmol scale for 2 h. XRD denotes X-ray diffraction analysis (for details, see the Supporting Information).
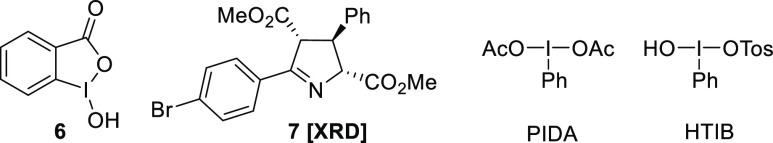

b NMR yield, using 0.1 mmol of mesitylene as an internal standard.

c Isolated yield in parentheses. nd denotes not detected.

With the optimized conditions in hand, the scope of easily accessible complex pyrrolidines was investigated (Scheme 1). A range of pyrrolidines bearing both electron-deficient and -rich α-aryl substituents afforded the desired cyclobutanes (**8**–**18**) in 42–88% yields. Pyrrolidines with electron-rich α-aryl substituents gave cyclobutanes (**9**, **14**) in lower yield in comparison to those with electron-deficient groups (**11**, **15**). This is due to the known overoxidation of electron-rich arenes by hypervalent iodine reagents.^37^ In general, the position of substituents on the α-aryl ring did not have a prominent effect on the yield. Next, pyrrolidines bearing an α-heteroarene gave cyclobutanes (**19**–**22**) in 24–42% yield. Despite the relatively low yield, the developed method represents an effective approach for the stereoselective synthesis of previously inaccessible, pharmaceutically important cyclobutanes with heterocyclic substituents.

**Scheme 1:**
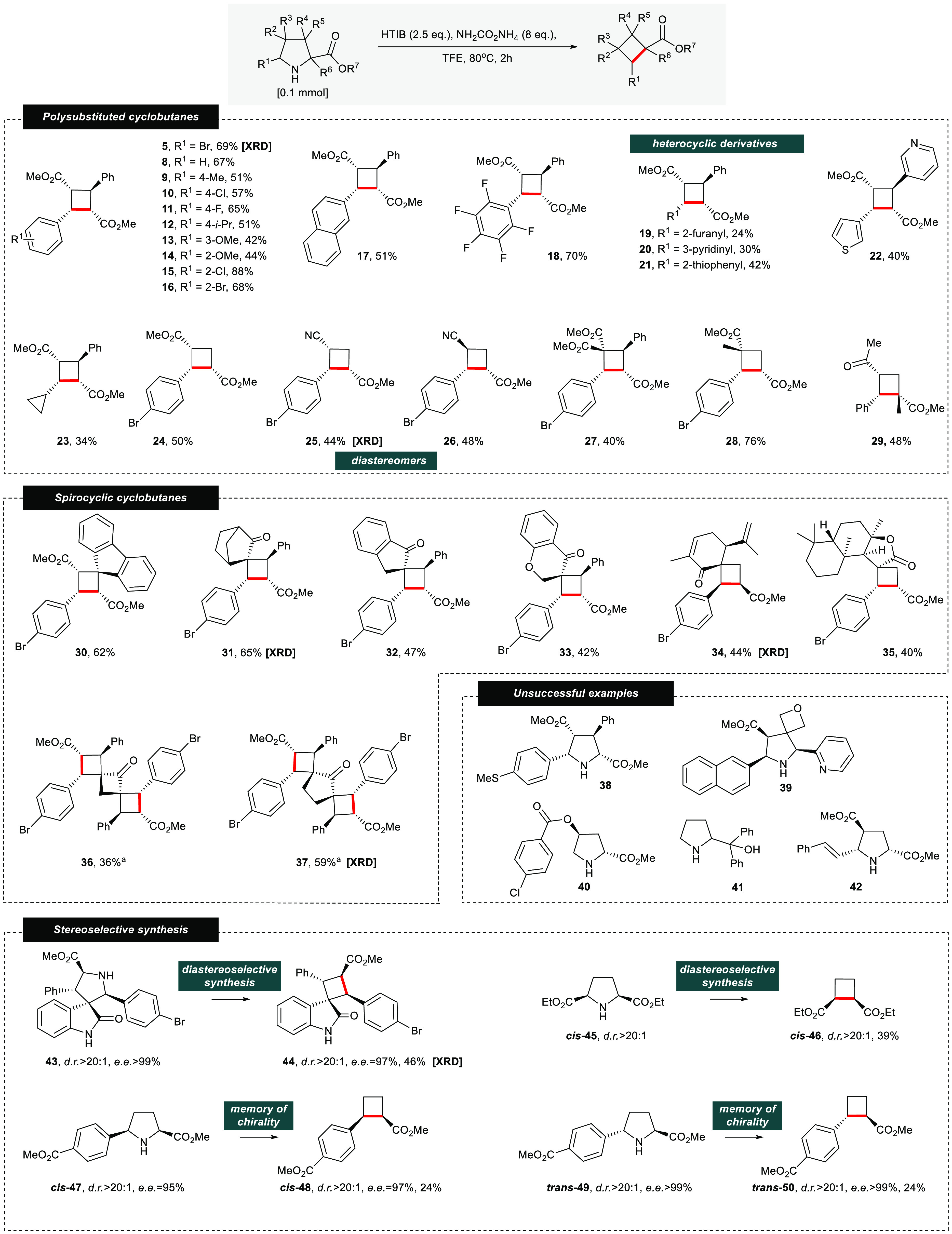
Scope of Contractive Synthesis of Multisubstituted Cyclobutanes from Pyrrolidines Using 5 equiv of HTIB for 12 h. XRD denotes X-ray diffraction analysis (for details, see the Supporting Information).

Afterward, the scope of pyrrolidines with different substitution patterns was explored. The replacement of the aryl group with an alkyl group such as cyclopropyl gave cyclobutane **23** as the exclusive product. Removal of the β-aryl group of pyrrolidine reduced the yield to 50% (**24**) from 69% (**5**). Replacing the β-ester group of **24** with nitrile group delivered **25** and **26** in comparable yields from the corresponding *exo*- and *endo*-pyrrolidines. Remarkably, the diastereoselective formation of cyclobutanes is not affected by the stereochemistry at the β-position of the corresponding pyrrolidines, allowing efficient selective access to the corresponding diastereomers (**25**, **26**).

Further studies of the substitution pattern of pyrrolidines were performed by the introduction of a quaternary carbon center. Installation of a diester group at the β-position gave cyclobutane **27** in 40% yield, while an additional β-methyl group gave **28** in 76% yield (without a β-methyl group (**24**), 50% yield). The β-quaternary carbon on pyrrolidine may positively guide the formation of a strained C–C bond via a Thorpe–Ingold effect.^61^ Notably, a pyrrolidine containing a quaternary carbon contiguous to the nitrogen atom successfully gave cyclobutane **29**. We expanded the substrate scope by preparing challenging spirocyclobutanes.^62^ Spirocyclobutanes, including **30**–**33**, were obtained stereoselectively from the corresponding pyrrolidines in 42–65% yields. The natural-product-derived spirocyclobutanes **34** (from carvone) and **35** (from (+)-sclareolide) were prepared successfully. Importantly, our method allows the synthesis of complex scaffolds, connecting bioactive structures with a cyclobutane to form spirocycles, in order to explore the biochemical space.^63^ Simultaneous ring contraction of two pyrrolidines in the same compound was accomplished using 5 equiv of HTIB to give **36** and **37**. It should be noted that all products described above were formed stereoselectively and the formation of only one diastereomer was observed (dr > 20:1). In addition to the broad scope of the developed contraction method, the use of pyrrolidines **38**–**42** under the standard reaction conditions did not result in the formation of the desired products. These reactions usually yielded complex mixtures of byproducts.

Finally, being very impressed by the stereoselective formation of cyclobutanes **25** and **26**, we were keen to extend the scope of stereoselective transformations. The optically pure spirooxindole **43** was transformed to the corresponding product **44** in 46% yield with excellent stereocontrol (dr > 20:1, ee = 97%). Ring contraction of the *cis*-substituted pyrrolidine-2,5-dicarboxylate (*cis*-**45**) led to the stereoselective formation of the cyclobutane *cis*-**46** in 39% yield. This result is very important because all stereocenters in the molecule were destroyed during the reaction, but the stereoinformation on the starting pyrrolidine was transferred to the product without erosion. Furthermore, in subsequent experiments optically pure pyrrolidines were subjected to the standard reaction conditions. To our delight, pyrrolidine **47** was converted to cyclobutane **48** in low yield (24%) but with outstanding diastereo- and enantiocontrol (dr > 20:1, ee > 97%). Moreover, *trans*-pyrrolidine **49** was transformed to *trans*-cyclobutane **50** with exceptional diastereo- and enantiocontrol (dr > 20:1, ee > 99%). Those extraordinary results indicate an unaltered memory of chirality for the developed novel ring contraction method, allowing access to novel enantiopure cyclobutane derivatives. This also indicates that the last step of the reaction, ring closure, proceeds with a very high reaction rate.

After demonstrating the substrate scope, we conducted a further investigation to elucidate the possible reaction mechanism (Scheme 2). Under the optimized conditions, pyrrolidine **51** underwent ring contraction to give **52** in 32% yield along with methyl cinnamate (**53**) in 9% yield, formed via β-fragmentation (Scheme 2A). These results coincide with Dervan’s finding^19−23^ and strongly hint at the involvement of a thermally generated singlet 1,4-biradical as a possible intermediate (Figure 1B). Radical trapping experiments using various scavengers were conducted (Scheme 2B). The formation of **5** was suppressed when TEMPO, 1,1-diphenylethylene, or 9,10-dihydroanthracene were added. Moreover, anthracene was observed via GCMS in the last experiment, which was not identified in the control without adding the starting material, implying that a radical pathway might be involved^64,65^ (for details, see the Supporting Information). To verify our initial proposal that the contraction synthesis of cyclobutane from pyrrolidine involves electrophilic amination of pyrrolidines, the *N*-aminated pyrrolidine **54** was prepared and was exposed to the standard conditions to give **52** in 79% yield, which is a significant improvement to the direct ring contraction from pyrrolidine (i.e., 25% yield) (Scheme 2C, also see the Supporting Information). The linear secondary amine **55** underwent oxidation to give benzonitrile (**56**) in 77% yield under the optimized conditions, in which HTIB and the ammonium salt could oxidize amines into nitriles^66^ (Scheme 2D).

**Scheme 2:**
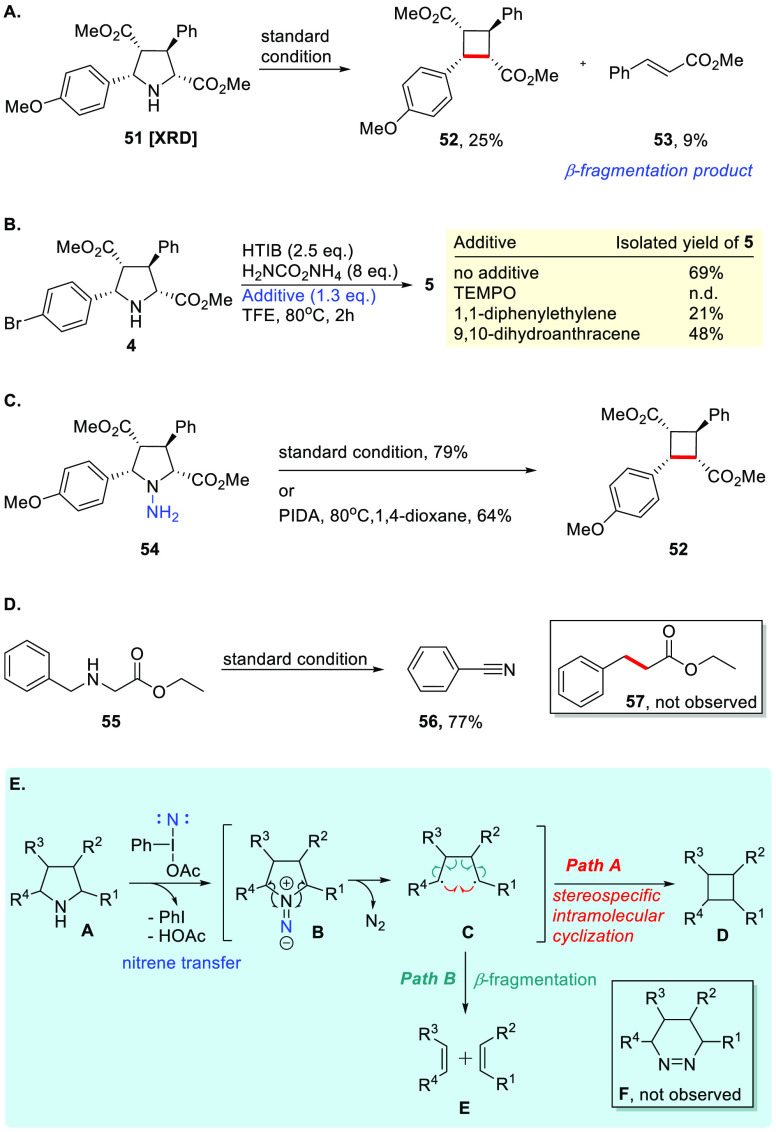
Further Investigation of Contractive Synthesis of Cyclobutanes: (A) Observation of Olefin Side Product Resulting from β-fragmentation; (B) Radical Trapping Experiments Using Various Scavengers; (C) Oxidative Ring Contraction of *N*-Aminopyrrolidine; (D) Attempts of Nitrogen Deletion from a Linear Secondary Amine; (E) Proposed Reaction Mechanism

The proposed reaction mechanism is illustrated in Scheme 2E. The observed stereospecificity of cyclobutane synthesis and the formation of a β-fragmentation product suggest the formation of a 1,4-biradical species, which results in rapid C–C bond formation. Treatment of pyrrolidine **A** with the *in situ* generated iodonitrene species leads to electrophilic amination, affording the 1,1-diazene **B** as a reactive intermediate. This reactive 1,1-diazene **B** proceeds further to give the 1,4-biradical **C**, via nitrogen extrusion, which undergoes intramolecular cyclization leading to C–C bond formation to give cyclobutane **D** (path A). We do not have any information on whether C–N bond breaking is occurring in a simultaneous or stepwise fashion, while 1,2-diazene **F** rearranged from 1,1-diazene **B** was not observed. The stereospecificity of the cyclobutane **D** from **B** reflects two simultaneous C–N bond cleavages with the formation of 1,4-biradical **C**, which rapidly undergoes cyclization. Meanwhile, olefinic side products **E** formed as an outcome of β-fragmentation of 1,4-biradical **C** through homolytic C–C bond cleavage (path B).

We envisioned that our method could directly forge the functionalized, unsymmetrical truxillate core of piperarborenine B (**3**)^67−69^ (Scheme 3). Pyrrolidine **60**, which was prepared from Ag/PPh_3_-catalyzed [3 + 2] cycloaddition of olefin **58** with imine **59**,^70^ was subjected to nitrogen extrusion/ring contraction to afford cyclobutane **61** in 30% yield. Treatment of cyclobutane **61** with TFA in dichloromethane led to the hydrolysis of the *tert*-butyl ester, in which the resulting carboxylic acid was converted to the corresponding acyl chloride followed by acylation with aniline **62** to give **63** in 67% yield in one pot. Cyclobutane **63** was subjected to a Krapcho dealkoxycarboxylation to give substituted cyclobutane precursor **64**, which was transformed into piperarborenine B (**3**) using Xie and Tang’s procedure.^69^ Our approach shows an alternative path to the synthesis of biologically important cyclobutanes, especially to those containing an unsymmetrical cyclobutane core.

**Scheme 3:**
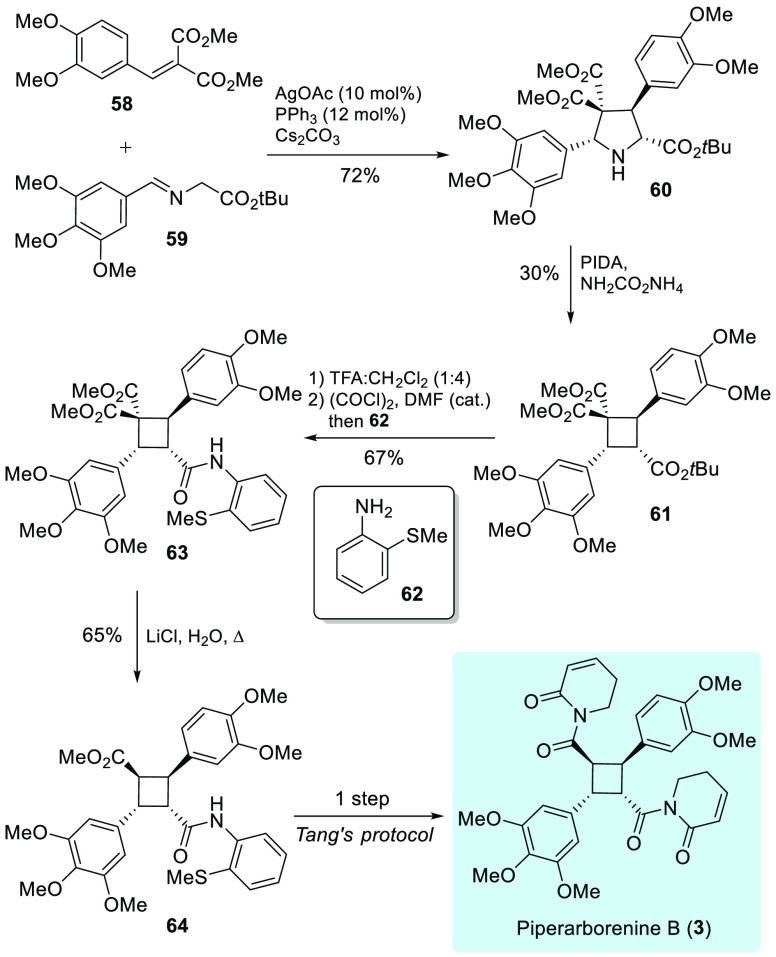
Formal Stereoselective Synthesis of Piperarborenine B (**3**)

In summary, we developed a novel and highly stereoselective synthesis of substituted cyclobutane derivatives from easily accessible pyrrolidines using iodonitrene chemistry, showing a good functional group compatibility. Studies on the reaction mechanism suggested the formation of a 1,4-biradical as a possible intermediate. A concise formal synthesis of piperarborenine B (**3**) was accomplished using the developed method.

## Supporting Information Available

Experimental procedures for all reactions and characterization data for all products, including ^1^H and ^13^C NMR spectra (PDF)

## Accession Codes

CCDC 2080159, 2080166, 2080174, 2080185, 2080194, 2080203,2080204, and 2105004 contain the supplementary crystallographic data for this paper. These data can be obtained free of charge via www.ccdc.cam.ac.uk/data_request/cif, or by emailing data_request@ccdc.cam.ac.uk, or by contacting The Cambridge Crystallographic Data Centre, 12 Union Road, Cambridge CB2 1EZ, UK; fax: +44 1223 336033.
