# Supplementary material for: Stereoselective Synthesis of Cyclobutanes by Contraction of Pyrrolidines
Source: J Am Chem Soc. 2021 Nov 8;143(45):18864–70. doi: 10.1021/jacs.1c10175 (PMC8603356; doi:10.1021/jacs.1c10175)

Supporting Information

Stereoselective synthesis of cyclobutanes by contraction of pyrrolidines

Chunngai Hui[1,2], Lukas Brieger[2], Carsten Strohmann[2], Andrey P. Antonchick[1,2,3]*

[1] Max Planck Institute of Molecular Physiology, Department of Chemical Biology, Otto-Hahn-Strasse 11, 44227 Dortmund, Germany

[2] Technical University Dortmund, Faculty of Chemistry and Chemical Biology, Otto-Hahn-Strasse 6, 44221 Dortmund, Germany

[3] Nottingham Trent University, School of Science and Technology, Department of Chemistry and Forensics, Clifton Lane, NG11 8NS Nottingham, United Kingdom

Contents

General S3
General procedure for the preparation of pyrrolidines S4
General procedure A S4
General procedure B S4
General procedure for the synthesis of cyclobutanes from pyrrolidines. S5
General procedure C S5
General procedure D S5
Visual demonstration of the synthesis of cyclobutanes from pyrrolidines S6
Optimization tables S8
Table S1. Screening of solvent S8
Table S2. Screening of oxidant. S9
Table S3. Screening of nitrogen source S10
Table S4. Screening of reaction temperature S11
Table S5. Screening of equivalence of reagents S12
Characterization data for pyrrolidines S13
Characterization data for cyclobutanes S25
Stereoselective synthesis of cyclobutanes from pyrrolidines S38
Ring contraction of *N*-aminopyrrolidine S47
Oxidation of pyrrolidine as side reaction S48
X-ray diffraction (XRD) analysis S52
Reported total synthesis of piperarborenine B S69
Formal synthesis of piperarborenine B S70
Synthesis and characterization of synthetic intermediates S71
Observation of olefinic side products using general procedure C S73
References S74
Spectra S75
NMR spectra of cyclobutanes S75
Spectra for formal synthesis of piperarborenine B S113
NMR spectra of pyrrolidines S118

General

Unless otherwise noted, all commercially available compounds were used as provided without further purification. Solvents for chromatography were technical grade.

Analytical thin-layer chromatography (TLC) was performed on Merck silica gel aluminium plates with F-254 indicator. Compounds were visualized by irradiation with UV light or potassium permanganate staining. Column chromatography was performed using silica gel Merck 60 (particle size 0.040-0.063 mm).

^{1}H-NMR, ^{13}C-NMR and ^{19}F-NMR were recorded on a Bruker DRX400 (400 MHz), Bruker DRX500 (500 MHz), INOVA500 (500 MHz) and Bruker DRX700 using CD_2Cl_2 or $CDCl_3$ as solvent. Data are reported in the following order: chemical shift (δ) values are reported in ppm with the solvent resonance as internal standard (standard (CD_2Cl_2: δ = 5.32 ppm for ^{1}H, δ = 53.84 ppm for ^{13}C; $CDCl_3$: δ = 7.26 ppm for ^{1}H, δ = 77.16 ppm for ^{13}C). Multiplicities are indicated s (singlet), d (doublet), t (triplet), q (quartet), m (multiplet); coupling constants (J) are given in Hertz (Hz).

High resolution mass spectra (HR-MS) were recorded on an *LTQ Orbitrap* mass spectrometer coupled to an Accela HPLC-System (HPLC column: Hypersyl GOLD, 50 mm x 1 mm, particle size 1.9 μm, ionization method: electron spray ionization). Fourier transform infrared spectroscopy (FT-IR) spectra were obtained with a Bruker Tensor 27 spectrometer (ATR, neat) and are reported in terms of frequency of absorption (cm-1). The enantiomeric excesses were determined by HPLC analysis using a chiral stationary phase column (CHIRALCEL IC, CHIRALCEL IA; eluent: (CH_2Cl_2/EtOH = 100/2) / iso-hexane, *i*-PrOH / iso-hexane; 4.6 mm x 250 mm, particle size 5 μm). The chiral HPLC methods were calibrated with the corresponding racemic mixtures.

General procedure for the preparation of pyrrolidines

General procedure A

The preparation of pyrrolidines as reaction substrate

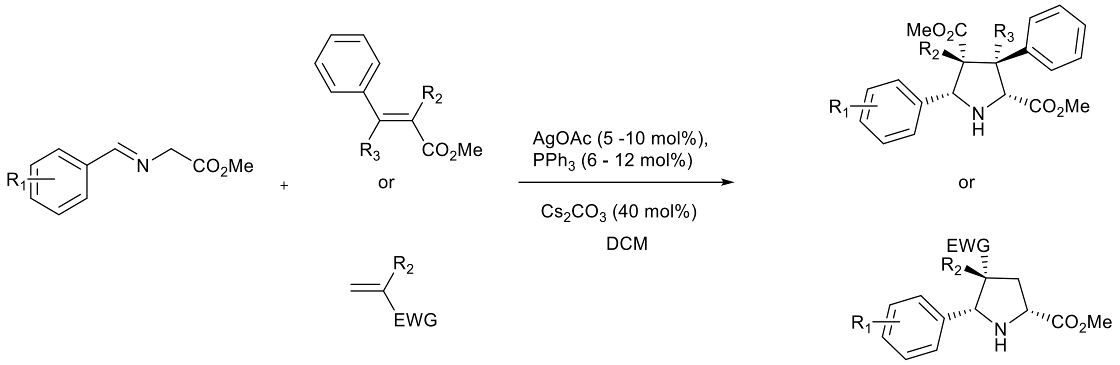

Under argon atmosphere, PPh_3 (6 - 12 mol%) and AgOAc (5 – 6 mol%) were dissolved in 20 mL DCM in a 50 ml round bottom flask, and stirred at room temperature for 1 hour. Then, imine substrate (1 mmol, 1 eq.), Cs_2CO_3 (40 mol%) and enone (1.1 mmol, 1.1 eq.) were added sequentially. Once starting material was consumed (monitored by TLC), the mixture was filtered through celite and the filtrate was concentrated. The crude product was purified by column chromatography (petroleum ether:acetone = 10:1 or petroleum ether:acetone = 5:1) to give the corresponding cycloaddition product. The iminoesters used for 1,3-dipolar cycloaddition were prepared according to the reported procedure.[1]

General procedure B

The preparation of pyrrolidines as reaction substrate with double [3+2] cycloaddition

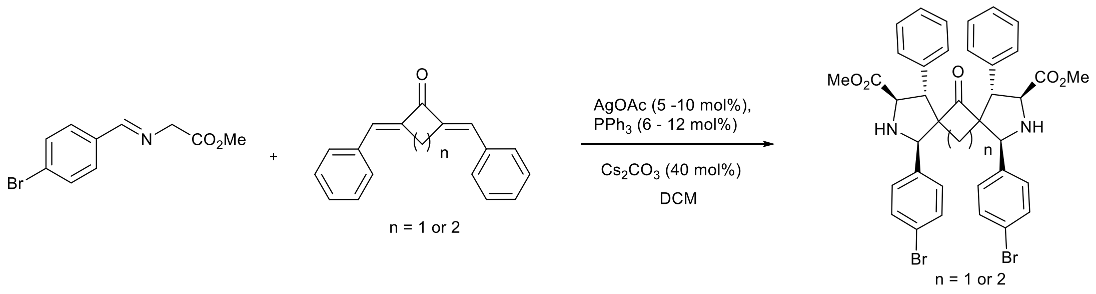

Under argon atmosphere, PPh_3(0.24 mmol, 15 mol%) and AgOAc (0.16 mmol, 10 mol%) were dissolved in 2 mL DCM, and stirred at room temperature for about 1h. Then, imine substrate (4.84 mmol, 3 eq.), Cs_2CO_3 (0.65 mmol, 40 mol%) and dienone or bis-α,β-unsaturated ester (1.61 mmol ,1 eq.) were added sequentially. Once starting material was consumed (monitored by TLC), the mixture was filtered through celite and the filtrate was concentrated to dryness. The crude product was purified by column chromatography (petroleum ether:acetone = 5:1) to give the corresponding cycloaddition product. The dienone were prepared according to the reported procedure.[2] The iminoesters used for 1,3-dipolar cycloaddition were prepared according to reference 1.

General procedure for the synthesis of cyclobutanes from pyrrolidines.

General procedure C

Under ambient atmosphere, HTIB (98 mg, 0.25 mmol, 2.5 eq.), ammonium carbamate (62 mg, 0.8 mmol, 8 eq.) and pyrrolidine (0.1 mmol, 1 eq.) were dissolved in 1 mL 2,2,2-trifluoroethanol and stirred at 80°C for two hours. The reaction vial was cooled down to room temperature and the vial cap was opened slowly. The reaction mixture was concentrated under vacuum. The crude mixture was directly charged onto silica gel and the product was isolated using petroleum ether / acetone as eluent.

General procedure D

Similar procedure to general procedure C except 5 eq. of HTIB is used instead of 2.5 eq. The reaction time is extended from 2h to 12h.

Visual demonstration of the synthesis of cyclobutanes from pyrrolidines

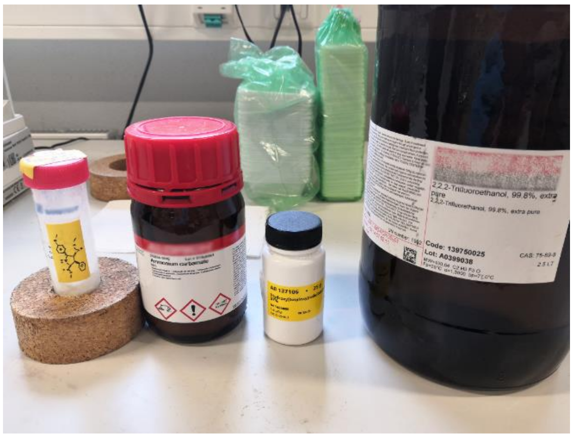

1. The reagents used are pyrrolidine, ammonium carbamate, HTIB and TFE.

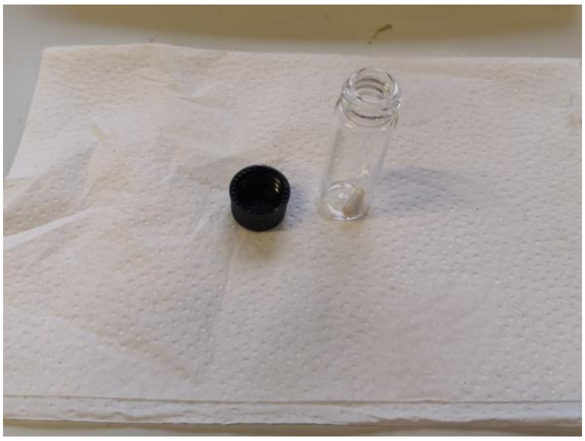

2. A magnetic stir bar was put into a reaction vial (with cap)

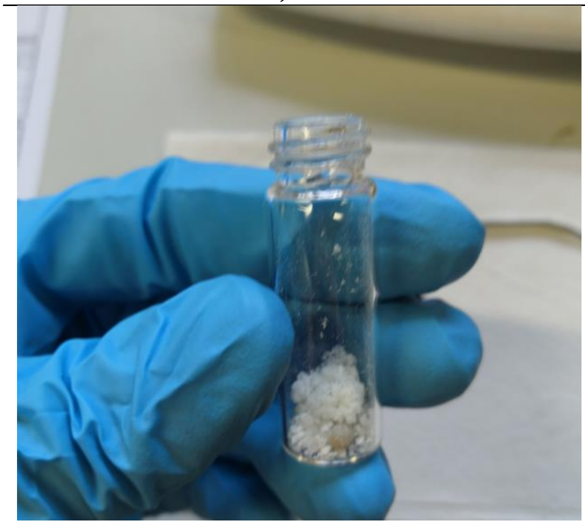

3. The sequential addition of pyrrolidine, ammonium carbamate, HTIB to the reaction tube is complete.

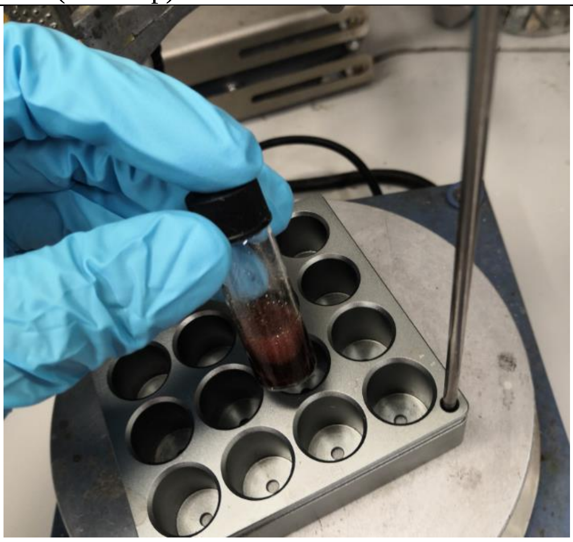

4. TFE (solvent) is added to the reaction mixture. Close the reaction tube tightly with the cap. The reaction is immediately stained dark brown and gas is gently released. Place in a preheated metal block (80°C) as soon as possible.

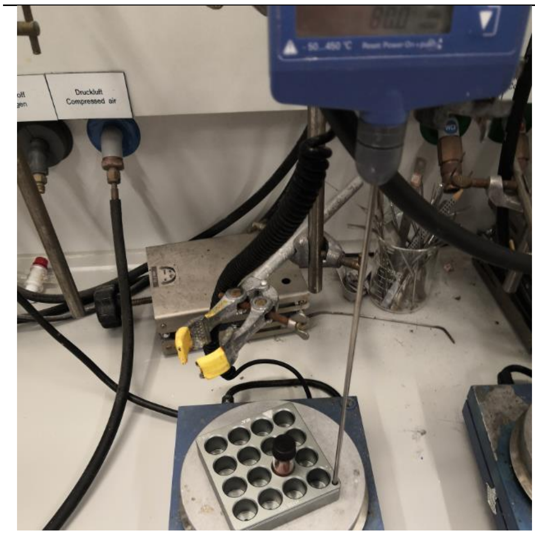

5. Heating at 80°C for 2h with vigorous stirring.

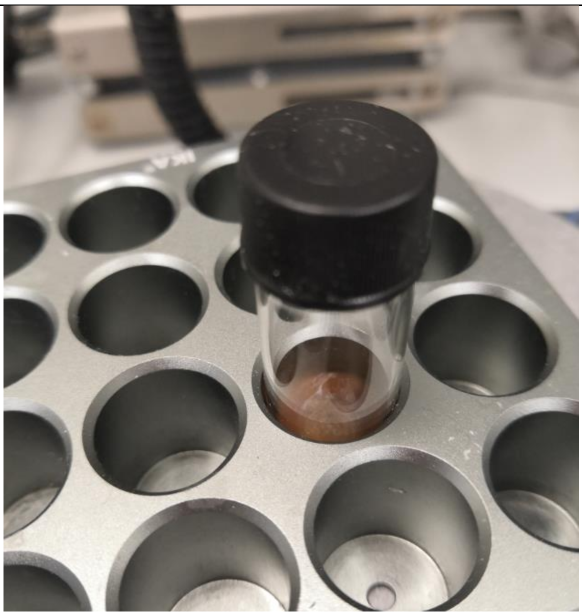

6. After 10 min, reaction changes from dark brown to light brown.

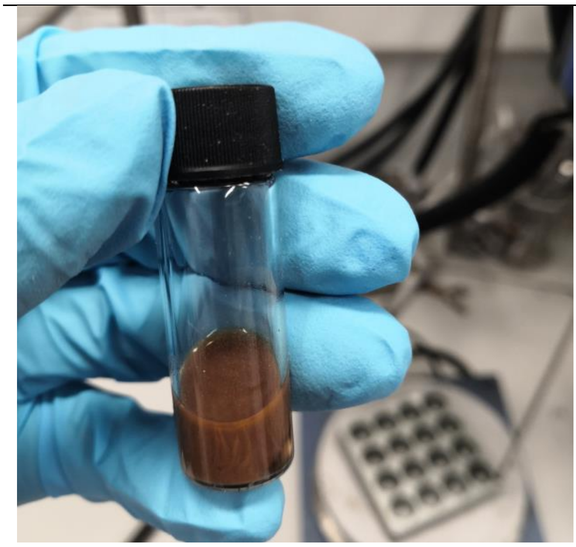

7. After 2h reaction, reaction is cooled down to room temperature (around 10 minutes). Then open the cap slowly.

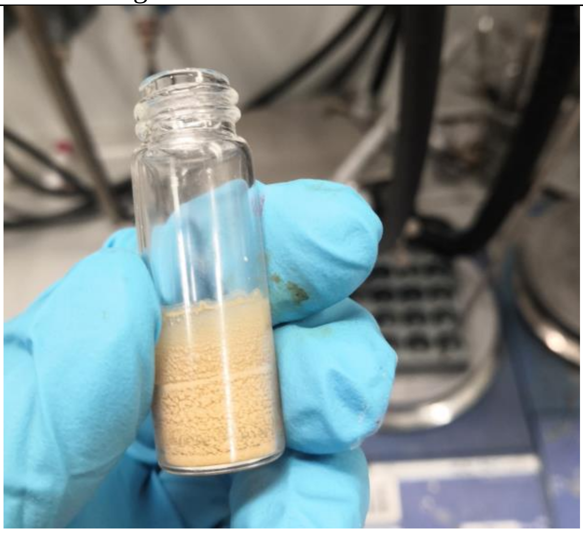

8. Remove the magnetic stir bar. TFE (solvent) is evaporated and is subjected to flash chromatography.

Optimization tables

Table S1. Screening of solvent

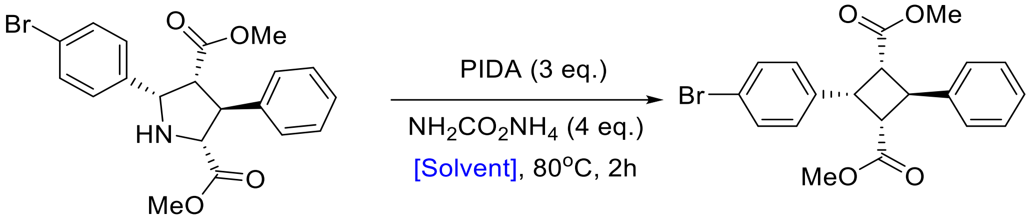

Entry	Variation from standard condition	NMR Yield (%) [a,b]
**1**	**2,2,2-trifluoroethanol (TFE)**	**54**
2	i-PrOH	24
3	*t*-BuOH	21
4	MeCN	36
5	EtOAc	15
6	AcOH	N.D.
7	Toluene	N.D.
8	DMF	3
9	$PhCF_3$	15
10	H_2O	N.D.
11	1,2-DCE	12
12	1,4-dioxane	15
13	MeOH	48 (44)
14	THF	N.D.
15	HFIP	36

[a]0.1 mmol of 1,3,5-trimethylbenzene as internal standard. [b]Isolated yield in parentheses. N.D. represents "not detected".

Table S2. Screening of oxidant.

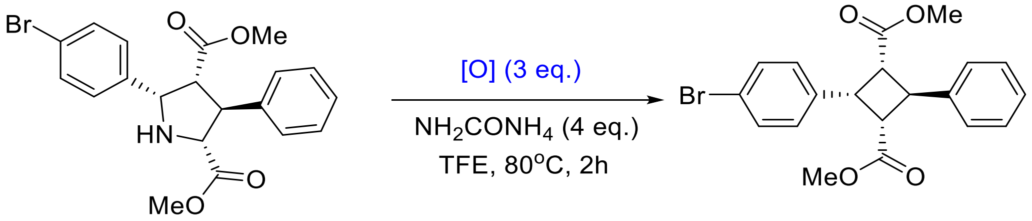

Entry	Variation from standard condition	NMR Yield (%) [a,b]
1	PIDA (**S1**)	54
2	PIFA (**S2**)	6 (37)
3	[bis-(Trifluoroacetoxy)iodo]pentafluorobenzene (**S3**)	33
4	bis(Tert-butylcarbonyloxy)iodobenzene (**S4**)	51
5	Iodosobenzene (**S5**)	51 (51)
6	Diphenyliodonium bromide (**S6**)	N.D. (0)
7	IBX (**S7**)	N.D.
**8**	**HTIB (S8)**	**63 (69)**
9	Iodine (**S9**)	N.D.
10	*N*-Iodosuccinimide (**S10**)	N.D.
11	1-Acetoxy-1,2-benziodoxol-3-(1*H*)-one (**S11**)	N.D.
12	(4-chlorophenyl)(hydroxy)-λ^3-iodaneyl 4-methylbenzenesulfonate (**S12**)	59
13	PhI=NTs (**S13**); without NH_2CONH_4	N.D.
14	1-hydroxy-1λ^3-benzo[d][1,2]iodaoxol-3(1*H*)-one (**S14**)	N.D.
15	(4-(*tert*-butyl)phenyl)(hydroxy)-λ^3-iodaneyl 4-methylbenzenesulfonate (**S15**)	48
16	(2-(methoxymethyl)phenyl)-λ^3-iodanediyl diacetate (**S16**)	37
17	PhI (20 mol%), *m*-CPBA (3 eq)	N.D.
18	[Hydroxy(methanesulfonyloxy)iodo]benzene (**S17**)	63 (63)
19	Iodosodilactone (**S18**)	N.D.

[a]0.1 mmol of 1,3,5-trimethylbenzene as internal standard. [b]Isolated yield in parentheses. N.D. represents “not detected”.

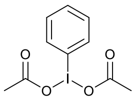
S1

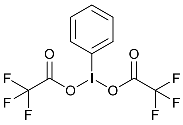
S2

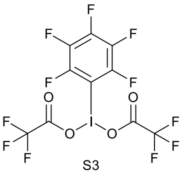
S3

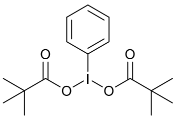
S4

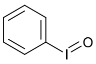
S5

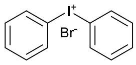
S6

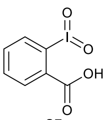
S7

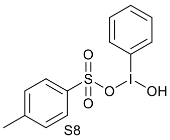
S8

I_2

S9

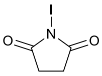
S10

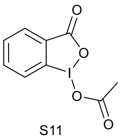
S11

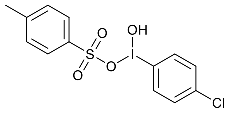
S12

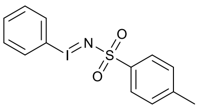
S13

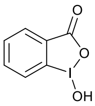
S14

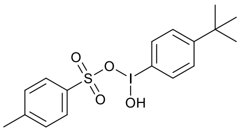
S15

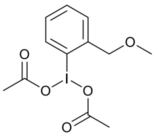
S16

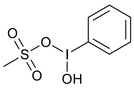
S17

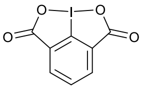
S18

Table S3. Screening of nitrogen source

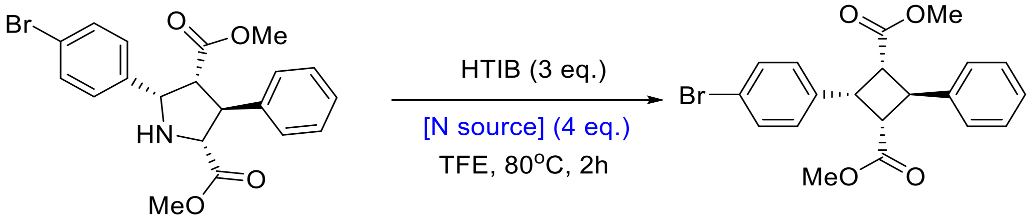

Entry	Variation from standard condition	NMR Yield (%) [a,b]
**1**	**$NH_2CO_2NH_4$**	**63 (69)**
2	O-(2,4-dinitrophenyl)hydroxylamine (DPH);<br>PIDA 3eq instead of HTIB 3eq.	N.D.
3	PhI=NTs; without HTIB	N.D.
4	NH_4Cl	N.D.
5	NH_4OAc	N.D.
6	NH_4PF_6	N.D.
7	NH_4OH (25% aq ammonia)	54
8	Hydrazine monohydrate (H_2NNH_2-H_2O)	N.D.
9	NH_4HCO_2	N.D.
10	NH_2-OH (50% wt aq. solution)	N.D.
11	Hydroxylamine-*O*-sulfonic acid	N.D.
12	$(NH_2)_2C=S$	N.D.
13	NH_3 (7M in MeOH)	55
14	*tert*-butyl carbamate	N.D.
15	Boc-ONH_2	N.D.
16	NH_4HCO_3	N.D.
27	*N*-aminophthalimide	N.D.

[a]0.1 mmol of 1,3,5-trimethylbenzene as internal standard. [b]Isolated yield in parentheses. N.D. represents "not detected".

Table S4. Screening of reaction temperature

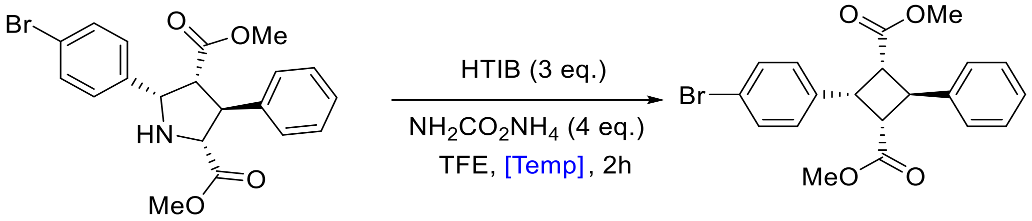

Entry	Variation from standard condition	NMR Yield (%) [a,b]
**1**	**80ºC**	**63 (69)**
2	20ºC	15
3	40ºC	29
4	60ºC	62
5	100ºC	42
6	120ºC	63
7	80ºC; In seal tube instead of reaction vial	(65)

[a]0.1 mmol of 1,3,5-trimethylbenzene as internal standard. [b]Isolated yield in parentheses.

Table S5. Screening of equivalence of reagents

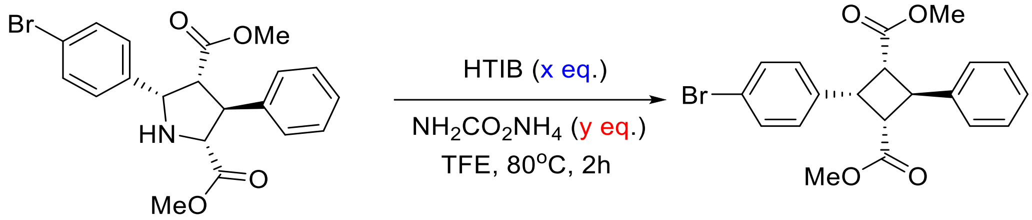

Entry	Variation from standard condition	NMR Yield (%) [a,b]
1	HTIB (3 eq), $NH_2CO_2NH_4$ (4 eq.)	63 (69)[c]
2	2 eq of HTIB instead of 3 eq	56
3	2.5 eq of HTIB instead of 3 eq	62 (54)
4	3.5 eq of HTIB instead of 3 eq	22
5	4 eq of HTIB instead of 3 eq	35
6	6 eq of HTIB instead of 3 eq	13
7	8 eq of HTIB instead of 3 eq	5
8	2 eq of $NH_2CO_2NH_4$	N.D.
9	3 eq of $NH_2CO_2NH_4$	15
10	6 eq of $NH_2CO_2NH_4$	50
11	8 eq of $NH_2CO_2NH_4$	58
**12**	**HTIB (2.5 eq), $NH_2CO_2NH_4$ (8 eq.)**	**(69)**

[a]0.1 mmol of 1,3,5-trimethylbenzene as internal standard. [b]Isolated yield in parentheses. N.D. represents "not detected". [c] Oxidation of pyrrolidine was observed when 4 eq. of ammonium carbamate was used. (see Scheme S3)

Characterization data for pyrrolidines

rac-Dimethyl (2*R*,3*S*,4*R*,5*S*)-5-(4-bromophenyl)-3-phenylpyrrolidine-2,4-dicarboxylate (Compound **4**)

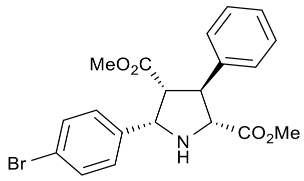

Compound 34 is prepared using procedure A.
Isolated yield: 74% (silica gel, 1:10 = acetone:petroleum ether)
^{1}H NMR (500 MHz, CDCl$_3$) δ 7.53 – 7.43 (m, 2H), 7.38 – 7.30 (m, 4H), 7.31 – 7.26 (m, 3H), 4.89 (d, *J* = 8.7 Hz, 1H), 4.18 (d, *J* = 8.9 Hz, 1H), 3.91 (t, *J* = 8.5 Hz, 1H), 3.73 (s, 3H), 3.58 – 3.54 (m, 1H), 3.24 ppm (s, 3H).
^{13}C NMR (126 MHz, CDCl$_3$) δ 172.63, 171.50, 139.46, 137.87, 131.68, 129.08, 128.95, 127.81, 127.68, 122.21, 67.24, 64.46, 58.37, 52.76, 51.84, 51.83 ppm.
HR-MS calculated for $C_{20}H_{21}{}^{79}BrNO_4$ = 418.0649 $[M+H]^+$, found: 418.0653; calculated for $C_{20}H_{21}{}^{81}BrNO_4$ = 420.0628 $[M+H]^+$, found: 420.0628.
IR ν_{max} (cm^{-1}) 1744, 1725, 1486, 1434, 1408, 1266, 1213, 1167, 1124, 1103, 1071, 1010, 969. 836.

rac-Dimethyl (2*R*,3*S*,4*R*,5*S*)-3,5-diphenylpyrrolidine-2,4-dicarboxylate (Compound **A1**)

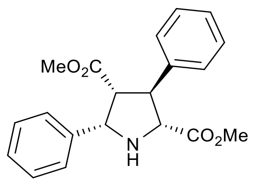

Compound A1 is prepared using procedure A.
Isolated yield: 44% (silica gel, 1:10 = acetone:petroleum ether)
^{1}H NMR (500 MHz, CDCl$_3$) δ 7.54 – 7.37 (m, 2H), 7.37 – 7.31 (m, 6H), 7.31 – 7.27 (m, 2H), 4.88 (d, *J* = 8.7, 1H), 4.14 – 4.08 (m, 1H), 3.91 (dd, *J* = 8.4, 8.4 Hz, 1H), 3.73 (s, 3H), 3.55 (dd, *J* = 8.4, 8.4 Hz, 1H), 3.16 ppm (s, 3H).
^{13}C NMR (126 MHz, CDCl$_3$) δ 172.95, 171.89, 140.01, 139.13, 128.86, 128.36, 127.93, 127.72, 127.34, 127.05, 67.72, 65.36, 58.88, 52.40, 52.27, 51.46 ppm.
HR-MS calculated for $C_{20}H_{22}NO_4$ = 340.1543 $[M+H]^+$, found: 340.1550
IR ν_{max} (cm^{-1}) 1732, 1603, 1495, 1454, 1434, 1376, 1264, 1215, 1166, 1029.

rac-Dimethyl (2*R*,3*S*,4*R*,5*S*)-3-phenyl-5-(*p*-tolyl)pyrrolidine-2,4-dicarboxylate (Compound **A2**)

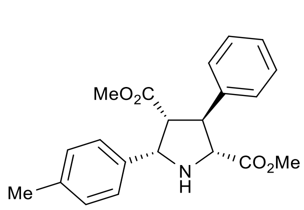

Compound A2 is prepared using procedure A.
Isolated yield: 48% (silica gel, 1:10 = acetone:petroleum ether)
^{1}H NMR (500 MHz, CDCl$_3$) δ 7.43 – 7.33 (m, 4H), 7.33 – 7.23 (m, 3H), 7.16 (d, *J* = 7.9 Hz, 2H), 4.89 (d, *J* = 8.7 Hz, 1H), 4.14 (d, *J* = 8.9 Hz, 1H), 3.91 (dd, *J* = 8.4, 8.4 Hz, 1H), 3.74 (s, 3H), 3.56 (dd, *J* = 8.4, 8.4 Hz, 1H), 3.22 (s, 3H), 2.34 ppm (s, 3H).
^{13}C NMR (126 MHz, CDCl$_3$) δ 172.82, 171.84, 139.90, 137.70, 135.72, 129.10, 128.87, 127.72, 127.37, 126.90, 67.54, 65.15, 58.77, 52.49, 52.26, 51.52, 21.16 ppm.
HR-MS calculated for $C_{21}H_{24}NO_4$ = 354.1700 $[M+H]^+$, found: 354.1700
IR ν_{max} (cm^{-1}) 1733, 1515, 1497, 1435, 1378, 1264, 1211, 1167, 1031, 822.

rac-Dimethyl (2*R*,3*S*,4*R*,5*S*)-5-(4-methoxyphenyl)-3-phenylpyrrolidine-2,4-dicarboxylate (Compound **51**)

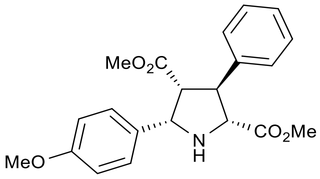

Compound 51 is prepared using procedure A.
Isolated yield: 67% (silica gel, 1:10 = acetone:petroleum ether)
^{1}H NMR (500 MHz, $CDCl_3$) δ 7.39 – 7.30 (m, 6H), 7.29 – 7.22 (m, 2H), 6.87 (d, *J* = 8.7 Hz, 2H), 4.82 (d, *J* = 8.7 Hz, 1H), 4.05 (d, *J* = 9.0 Hz, 1H), 3.88 (t, *J* = 8.6 Hz, 1H), 3.79 (s, 3H), 3.71 (s, 3H), 3.58 – 3.47 (m, 1H), 3.20 ppm (s, 3H).
^{13}C NMR (126 MHz, $CDCl_3$) δ 173.14, 172.01, 159.15, 140.09, 131.49, 128.81, 128.24, 127.73, 127.27, 113.64, 67.71, 64.89, 58.89, 55.25, 52.32, 52.28, 51.50 ppm.
HR-MS calculated for $C_{21}H_{24}NO_5$ = $[M+H]^+$, 370.1649, found: 370.1650.
IR ν_{max} (cm^{-1}) 1732, 1611, 1513, 1435, 1246, 1214, 1167, 1032, 835.

rac-Dimethyl (2*R*,3*R*,4*R*,5*S*)-5-(4-chlorophenyl)-3-phenylpyrrolidine-2,4-dicarboxylate (Compound **A3**)

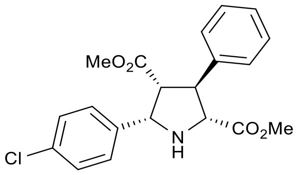

Compound A3 is prepared using procedure A.
Isolated yield: 45% (silica gel, 1:10 = acetone:petroleum ether)
^{1}H NMR (500 MHz, $CDCl_3$) δ 7.33 (m, 8H), 7.28 (d, *J* = 6.8 Hz, 1H), 4.92 – 4.81 (m, 1H), 4.17 – 4.07 (m, 1H), 3.90 (dd, *J* = 10.2, 6.7 Hz, 1H), 3.72 (s, 3H), 3.59 – 3.51 (m, 1H), 3.22 ppm (s, 3H).
^{13}C NMR (126 MHz, $CDCl_3$) δ 171.67, 129.02, 128.95, 128.67, 128.64, 127.91, 127.82, 127.58, 127.57, 67.47, 64.51, 58.55, 52.60, 51.95, 51.75 ppm.
HR-MS calculated for $C_{20}H_{21}{}^{35}ClNO_4$ = 374.1154 $[M+H]^+$, found: 374.1166; calculated for $C_{20}H_{21}{}^{37}ClNO_4$ = 376.1124 $[M+H]^+$, found: 376.1137;
IR ν_{max} (cm^{-1}) 1729, 1491, 1435, 1373, 1265, 1214, 1168, 1090, 1014, 836.

rac-Dimethyl (2*R*,3*S*,4*R*,5*S*)-5-(4-fluorophenyl)-3-phenylpyrrolidine-2,4-dicarboxylate (Compound **A4**)

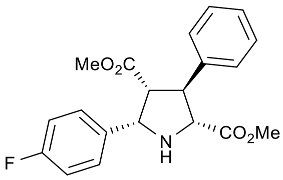

Compound A4 is prepared using procedure A.
Isolated yield: 41% (silica gel, 1:10 = acetone:petroleum ether)
^{1}H NMR (500 MHz, $CDCl_3$) δ 7.40 – 7.31 (m, 2H), 7.31 – 7.24 (m, 3H), 7.24 – 7.18 (m, 2H), 6.97 (t, *J* = 8.7 Hz, 2H), 4.80 (d, *J* = 8.8 Hz, 1H), 4.02 (d, *J* = 9.0 Hz, 1H), 3.84 (dd, *J* = 8.6, 8.6 Hz, 1H), 3.66 (s, 3H), 3.47 (t, *J* = 8.6, 8.6 Hz, 1H), 3.14 ppm (s, 3H).
^{13}C NMR (126 MHz, $CDCl_3$) δ 173.06, 171.75, 162.31 (d, J_{C-F} = 246.3 Hz), 139.84, 135.44 (d, J_{C-F} = 3.1 Hz), 128.85, 128.79, 127.72, 127.36, 115.16 (d, J_{C-F} = 21.4 Hz), 67.57, 64.45, 58.68, 52.35, 51.93, 51.51 ppm.
^{19}F NMR (470 MHz, $CDCl_3$) δ -114.50.
HR-MS calculated for $C_{20}H_{21}FNO_4$ = 358.1449 $[M+H]^+$, found: 358.1456.

IR ν_{max} (cm^{-1}) 1736, 1604, 1509, 1435, 1261, 1222, 1166, 841.

rac-Dimethyl (2*R*,3*S*,4*R*,5*S*)-5-(4-isopropylphenyl)-3-phenylpyrrolidine-2,4-dicarboxylate (Compound **A5**)

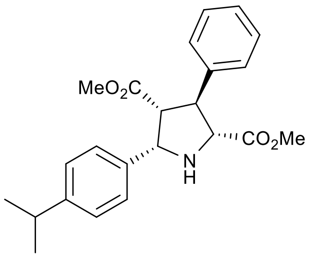

Compound A5 is prepared using procedure A.
Isolated yield: 58% (silica gel, 1:10 = acetone:petroleum ether)
^{1}H NMR (500 MHz, CDCl$_3$) δ 7.40 – 7.24 (m, 7H), 7.20 (d, *J* = 8.2 Hz, 2H), 4.86 (d, *J* = 8.7 Hz, 1H), 4.08 (d, *J* = 9.0 Hz, 1H), 3.98 – 3.87 (m, 1H), 3.72 (s, 3H), 3.60 – 3.46 (m, 1H), 3.14 (s, 3H), 2.90 (tt, *J* = 13.9, 6.9 Hz, 1H), 1.24 (s, 3H), 1.22 ppm (s, 3H).
^{13}C NMR (126 MHz, CDCl$_3$) δ 172.12, 171.40, 149.23, 129.03, 129.00, 127.74, 127.62, 126.99, 126.71, 126.66, 66.88, 64.90, 58.42, 52.87, 51.78, 51.69, 51.64, 33.80, 23.93 ppm.
HR-MS calculated $C_{23}H_{28}NO_4$ = 382.20123 [M+H]$^+$, found: 382.2022.
IR ν_{max} (cm^{-1}) 1735, 1436, 1265, 1216, 1168, 905, 835.

rac-Dimethyl (2*R*,3*S*,4*R*,5*S*)-5-(3-methoxyphenyl)-3-phenylpyrrolidine-2,4-dicarboxylate (Compound **A6**)

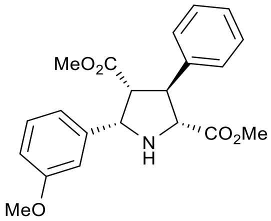

Compound A6 is prepared using procedure A.
Isolated yield: 32% (silica gel, 1:10 = acetone:petroleum ether)
^{1}H NMR (500 MHz, CDCl$_3$) δ 7.41 – 7.34 (m, 4H), 7.32 – 7.27 (m, 2H), 7.03 – 6.96 (m, 2H), 6.88 – 6.81 (m, 1H), 4.89 (d, *J* = 8.6 Hz, 1H), 4.13 (d, *J* = 8.9 Hz, 1H), 3.92 (t, *J* = 8.5 Hz, 1H), 3.84 (s, 3H), 3.75 (s, 3H), 3.63 – 3.56 (m, 1H), 3.25 ppm (s, 3H).
^{13}C NMR (126 MHz, CDCl$_3$) δ 173.05, 171.88, 159.71, 140.95, 140.02, 129.49, 128.96, 127.83, 127.47, 119.33, 113.65, 112.75, 67.71, 65.33, 58.82, 55.41, 52.53, 52.19, 51.66 ppm.
HR-MS calculated $C_{21}H_{24}NO_5$ = 370.1649 [M+H]$^+$, found: 370.1657.
IR ν_{max} (cm^{-1}) 1736, 1613, 1513, 1434, 1248, 1168, 1031, 834.

rac-Dimethyl (2*R*,3*S*,4*R*,5*S*)-5-(2-methoxyphenyl)-3-phenylpyrrolidine-2,4-dicarboxylate (Compound **A7**)

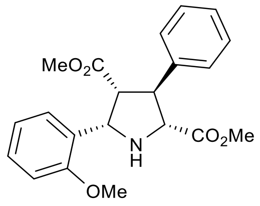

Compound A7 is prepared using procedure A.
Isolated yield: 38% (silica gel, 1:10 = acetone:petroleum ether)
^{1}H NMR (500 MHz, CDCl$_3$) δ 7.50 – 7.33 (m, 5H), 7.33 – 7.22 (m, 2H), 6.99 (td, *J* = 7.5, 1.1 Hz, 1H), 6.88 (dd, *J* = 8.2, 1.1 Hz, 1H), 5.08 (d, *J* = 8.1 Hz, 1H), 4.02 (d, *J* = 8.5 Hz, 1H), 3.91 – 3.87 (m, 1H), 3.87 (s, 3H), 3.76 (s, 3H), 3.64 (dd, *J* = 8.1, 5.7 Hz, 1H), 3.14 ppm (s, 3H).
^{13}C NMR (126 MHz, CDCl$_3$) δ 173.23, 172.78, 156.75, 141.52, 128.88, 128.68, 127.69, 127.14, 126.56, 126.47, 120.46, 110.00, 68.50, 61.16, 57.57, 55.46, 53.75, 52.29, 51.32 ppm.
HR-MS calculated $C_{21}H_{24}NO_5$ = 370.1649 $[M+H]^+$, found: 370.1657.
IR ν$_{max}$ (cm^{-1}) 1736, 1603, 1495, 1436, 1381, 1247, 1203, 1165, 1028, 912.

rac-Dimethyl (2*R*,3*S*,4*R*,5*S*)-5-(2-chlorophenyl)-3-phenylpyrrolidine-2,4-dicarboxylate (Compound **A8**)

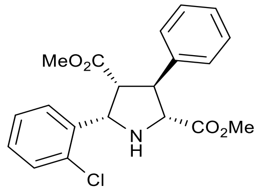

Compound A8 is prepared using procedure A.
Isolated yield: 50% (silica gel, 1:10 = acetone:petroleum ether)
^{1}H NMR (400 MHz, CDCl$_3$) δ 7.58 (dd, *J* = 7.8, 1.7 Hz, 1H), 7.46 – 7.31 (m, 5H), 7.29 (dd, *J* = 7.2, 1.5 Hz, 2H), 7.22 (td, *J* = 7.6, 1.7 Hz, 1H), 5.20 (d, *J* = 8.2 Hz, 1H), 4.04 (d, *J* = 8.3 Hz, 1H), 3.95 (dd, *J* = 8.3, 5.7 Hz, 1H), 3.76 (s, 3H), 3.69 (dd, *J* = 8.3, 5.7 Hz, 1H), 3.13 ppm (s, 3H).
^{13}C NMR (101 MHz, CDCl$_3$) δ 172.53, 140.98, 136.17, 133.41, 129.17, 128.92, 128.81, 127.64, 127.55, 127.28, 126.83, 67.91, 62.24, 56.46, 52.56, 52.32, 51.40 ppm.
HR-MS calculated $C_{20}H_{21}{}^{35}ClNO_4$ = 374.1154 $[M+H]^+$, found: 374.1158; calculated $C_{20}H_{21}{}^{37}ClNO_4$ = 376.1124 $[M+H]^+$, found: 376.1126.
IR ν$_{max}$ (cm^{-1}) 1737, 1650, 1438, 1264, 1203, 1175, 1035, 905.

*r*ac-Dimethyl (2*R*,3*S*,4*R*,5*S*)-5-(2-bromophenyl)-3-phenylpyrrolidine-2,4-dicarboxylate (Compound **A9**)

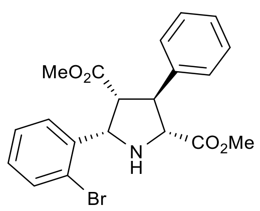

Compound A9 is prepared using procedure A.
Isolated yield: 67% (silica gel, 1:10 = acetone:petroleum ether)
^{1}H NMR (500 MHz, CDCl$_3$) δ 7.56 (d, *J* = 7.9 Hz, 2H), 7.41 – 7.32 (m, 5H), 7.31 – 7.26 (m, 1H), 7.16 (td, *J* = 7.6, 1.7 Hz, 1H), 5.20 (d, *J* = 8.2 Hz, 1H), 4.07 (d, *J* = 8.2 Hz, 1H), 3.97 (dd, *J* = 8.2, 5.7 Hz, 1H), 3.76 (s, 3H), 3.73 (dd, *J* = 8.2, 5.7 Hz, 1H), 3.14 ppm (s, 3H).

^{13}C NMR (126 MHz, CDCl$_3$) δ 172.43, 172.40, 140.89, 137.43, 132.57, 129.25, 128.97, 127.79, 127.67, 127.47, 127.34, 123.94, 67.78, 64.58, 56.20, 52.44, 52.31, 51.47 ppm.
HR-MS calculated $C_{20}H_{21}{}^{79}BrNO_4$ = 418.0649 [M+H]$^+$, found: 418.0653; calculated $C_{20}H_{21}{}^{81}BrNO_4$ = 420.0628 [M+H]$^+$, found: 420.0629.
IR ν$_{max}$ (cm^{-1}) 1736, 1496, 1435, 1379, 1205, 1168, 1121, 1026, 908,

rac-Dimethyl (2*R*,3*S*,4*R*,5*S*)-5-(2-bromophenyl)-3-phenylpyrrolidine-2,4-dicarboxylate (Compound **A10**)

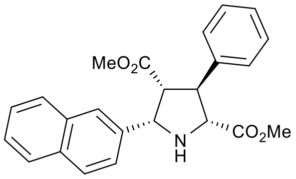

Compound A10 is prepared using procedure A.
Isolate yield: 65% (silica gel, 1:10 = acetone:petroleum ether)
^{1}H NMR (500 MHz, CDCl$_3$) δ 7.90 – 7.84 (m, 2H), 7.82 (d, *J* = 8.7 Hz, 2H), 7.53 – 7.45 (m, 3H), 7.37 (d, *J* = 4.5 Hz, 4H), 7.32 – 7.27 (m, 1H), 5.06 (d, *J* = 7.9 Hz, 1H), 4.18 (d, *J* = 7.9 Hz, 1H), 3.99 (t, *J* = 8.2 Hz, 1H), 3.76 (s, 3H), 3.64 (t, *J* = 8.1 Hz, 1H), 3.08 ppm (s, 3H).
^{13}C NMR (126 MHz, CDCl$_3$) δ 172.84, 171.92, 140.08, 140.04, 133.14, 132.98, 128.91, 128.11, 128.04, 127.74, 127.65, 127.41, 126.26, 126.12, 125.91, 125.05, 67.69, 65.47, 58.74, 52.51, 52.36, 51.52 ppm.
HR-MS calculated $C_{24}H_{24}NO_4$ = 390.1700 [M+H]$^+$, found: 390.1704.
IR ν$_{max}$ (cm^{-1}) 1736, 1601, 1497, 1434, 1376, 1270, 1221 1166, 859, 823.

rac-Dimethyl (2*R*,3*S*,4*R*,5*S*)-5-(perfluorophenyl)-3-phenylpyrrolidine-2,4-dicarboxylate (Compound **A11**)

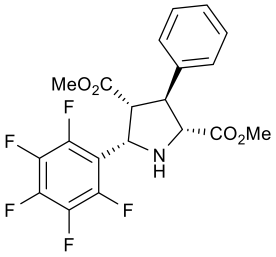

Compound A11 is prepared using procedure A.
Isolate yield: 41% (silica gel, 1:10 = acetone:petroleum ether)
^{1}H NMR (400 MHz, CDCl$_3$) δ 7.49 – 7.11 (m, 5H), 5.08 (d, *J* = 9.0 Hz, 1H), 4.04 – 3.79 (m, 2H), 3.64 (s, 3H), 3.53 (dd, *J* = 9.0, 7.4 Hz, 1H), 3.34 ppm (s, 3H).
^{13}C NMR (101 MHz, CDCl$_3$) δ 172.07, 171.51, 146.56, 144.09 (d, *J* = 2.9 Hz), 142.03, 139.77, 139.49, 138.86, 138.73, 136.47, 129.07, 127.71, 127.63, 112.91, 112.80, 112.65, 68.23, 57.93, 56.58, 52.70, 52.53, 52.14 ppm.
^{19}F NMR (377 MHz, CDCl$_3$) δ -140.73, -140.75, -140.79, -140.80, -153.77, -153.83, -153.89, -161.50, -161.52, -161.56, -161.57, -161.61, -161.63 ppm.
HR-MS calculated $C_{20}H_{17}F_5NO_4$ = 430.1072 [M+H]$^+$, found: 430.1078.
IR ν$_{max}$ (cm^{-1}) 1739, 1653, 1524, 1502, 1437, 1223, 1169, 1131, 1000, 970, 912.

rac-Dimethyl (2R,3S,4R,5S)-5-(furan-2-yl)-3-phenylpyrrolidine-2,4-dicarboxylate (Compound **A12**)

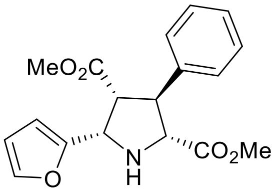

Compound A12 is prepared using procedure A.
Isolated yield: 49% (silica gel, 1:10 = acetone:petroleum ether)
^{1}H NMR (400 MHz, CDCl$_3$) δ 7.59 – 7.28 (m, 5H), 7.29 – 7.16 (m, 1H), 6.52 – 6.33 (m, 1H), 6.31 (ddd, *J* = 3.4, 1.8, 0.6 Hz, 1H), 4.86 (d, *J* = 8.0 Hz, 1H), 4.10 – 3.87 (m, 2H), 3.67 (s, 3H), 3.53 (t, *J* = 8.5 Hz, 1H), 3.41 (s, 3H).
^{13}C NMR (101 MHz, CDCl$_3$) δ 173.22, 171.05, 152.88, 142.30, 140.01, 128.76, 127.76, 127.25, 110.37, 107.60, 67.25, 59.03, 57.12, 52.34, 51.86, 50.95.
HR-MS calculated for $C_{18}H_{20}O_5N$ = 330.1336 [M+H]$^+$, found: 330.1340.
IR ν_{max} (cm^{-1}) 3002, 2944, 1632, 1442, 1375, 1039, 918.

rac-Dimethyl (2R,3S,4R,5S)-3-phenyl-5-(pyridin-3-yl)pyrrolidine-2,4-dicarboxylate (Compound **A13**)

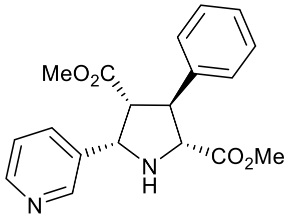

Compound A13 is prepared using procedure A.
Isolated yield: 41% (silica gel, 1:5 = acetone:petroleum ether)
^{1}H NMR (400 MHz, CDCl$_3$) δ 8.53 (s, 1H), 8.47 (d, *J* = 4.9 Hz, 1H), 7.84 (s, 1H), 7.45 – 7.09 (m, 6H), 4.85 (d, *J* = 9.0 Hz, 1H), 4.05 (d, *J* = 9.1 Hz, 1H), 3.96 – 3.82 (m, 1H), 3.66 (s, 3H), 3.62 – 3.45 (m, 1H), 3.15 (s, 3H).
^{13}C NMR (101 MHz, CDCl$_3$) δ 172.85, 171.33, 148.84, 148.69, 148.56, 139.37, 135.04, 128.85, 127.68, 127.43, 123.36, 67.40, 62.32, 58.26, 52.31, 51.58.
HR-MS calculated for $C_{19}H_{21}O_4N_2$ = 341.1496 [M+H]$^+$, found: 341.1501.
IR ν_{max} (cm^{-1}) 1740, 1631,1439, 1375, 1038, 918.

rac-Dimethyl (2R,3S,4R,5S)-3-phenyl-5-(thiophen-2-yl)pyrrolidine-2,4-dicarboxylate (Compound **A14**)

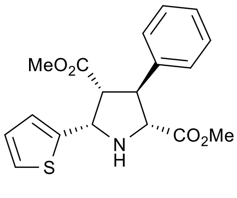

Compound A14 is prepared using procedure A.
Isolated yield: 37% (silica gel, 1:10 = acetone:petroleum ether)
^{1}H NMR (500 MHz, CDCl$_3$) δ 7.34 (dd, *J* = 6.0, 2.9 Hz, 4H), 7.30 – 7.27 (m, 1H), 7.23 (d, *J* = 5.2 Hz, 1H), 7.06 (d, *J* = 3.7 Hz, 1H), 6.98 (m, 1H), 5.11 (d, *J* = 8.1 Hz, 1H), 4.07 (d, *J* = 7.8 Hz, 1H), 3.93 (t, *J* = 8.8 Hz, 1H), 3.71 (s, 3H), 3.60 (t, *J* = 4.3 Hz, 1H), 3.36 ppm (s, 3H).
^{13}C NMR (126 MHz, CDCl$_3$) δ 171.11, 128.82, 128.02, 127.83, 127.38, 126.89, 126.87, 125.09, 124.96, 67.23, 60.65, 58.53, 52.43, 51.75, 51.10 ppm.
HR-MS calculated $C_{18}H_{20}NO_4S$ = 346.1108 [M+H]$^+$, found: 346.1117.
IR ν_{max} (cm^{-1}) 1735, 1603, 1497, 1435, 1376, 1348,1273, 1213, 1167, 1094, 1030, 925, 850.

rac-Dimethyl (2*R*,3*S*,4*R*,5*S*)-3-(pyridin-3-yl)-5-(thiophen-3-yl)pyrrolidine-2,4-dicarboxylate (Compound **A15**)

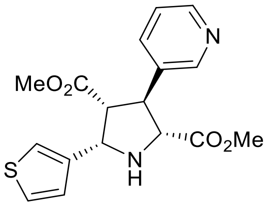

Compound A15 is prepared using procedure A.
Isolated yield: 37% (silica gel, 1:5 = acetone:petroleum ether)
^{1}H NMR (500 MHz, CD_2Cl_2) δ 8.54 (d, *J* = 1.6 Hz, 1H), 8.48 (dd, *J* = 4.8, 1.7 Hz, 1H), 7.65 (dt, *J* = 7.9, 0.6 Hz, 1H), 7.34 (ddd, *J* = 2.9, 1.4, 0.8 Hz, 1H), 7.31 (dd, *J* = 4.9, 3.0 Hz, 1H), 7.28 (ddd, *J* = 7.8, 4.8, 0.9 Hz, 1H), 7.05 (dd, *J* = 4.9, 1.4 Hz, 1H), 5.05 – 4.87 (m, 1H), 4.01 (d, *J* = 9.4 Hz, 1H), 3.82 (t, *J* = 9.1 Hz, 1H), 3.68 (s, 3H), 3.52 (t, *J* = 8.7 Hz, 1H), 3.29 (s, 3H).
^{13}C NMR (126 MHz, CD_2Cl_2) δ 173.39, 171.80, 150.23, 149.13, 142.03, 136.04, 135.53, 127.21, 126.24, 124.06, 122.68, 67.77, 61.57, 58.45, 52.77, 52.04, 49.58.
HR-MS calculated for $C_{17}H_{19}O_4N_2S$ = 347.1060 [M+H]$^+$, found: 347.1064.
IR ν_{max} (cm^{-1}) 1736, 1437, 1375, 1170, 1038, 918.

rac-Dimethyl (2*R*,4*R*,5*S*)-5-(4-bromophenyl)pyrrolidine-2,4-dicarboxylate (Compound **A16**)

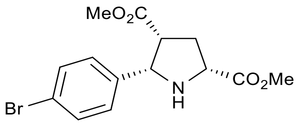

Compound A16 is prepared using procedure A.
Isolated yield: 64% (silica gel, 1:10 = acetone:petroleum ether)
^{1}H NMR (500 MHz, $CDCl_3$) δ 7.43 (d, *J* = 8.5 Hz, 2H), 7.24 – 7.19 (m, 2H), 4.50 (d, *J* = 7.8 Hz, 1H), 3.98 (t, *J* = 8.2 Hz, 1H), 3.82 (s, 3H), 3.31 (td, *J* = 7.5, 6.6 Hz, 1H), 3.27 (s, 3H), 2.41 ppm (ddd, *J* = 8.1, 7.0, 2.0 Hz, 2H).
^{13}C NMR (126 MHz, $CDCl_3$) δ 173.71, 172.77, 138.35, 131.32, 128.61, 121.53, 65.05, 59.79, 52.41, 51.48, 49.46, 33.09 ppm.
HR-MS calculated $C_{14}H_{17}{}^{79}BrNO_4$ = 342.0336 [M+H]$^+$, found: 342.0345**;** calculated $C_{14}H_{17}{}^{81}BrNO_4$ = 344.0315 [M+H]$^+$, found: 344.0322.
IR ν_{max} (cm^{-1}) 1734, 1653, 1559, 1436, 1203, 1010.

rac-Methyl (2*R*,4*R*,5*S*)-5-(4-bromophenyl)-4-cyanopyrrolidine-2-carboxylate (Compound **A17**)

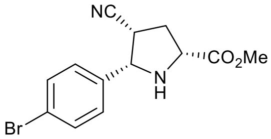

Compound A17 is prepared using the modified procedure A AgOAc (0.2 eq)/PPh_3 (0.22 eq)/Et_3N (0.5 eq)/CH_2Cl_2/rt.
Isolated yield: 16% (silica gel, 1:10 = acetone:petroleum ether)
^{1}H NMR (700 MHz, $CDCl_3$) δ 7.53 (d, *J* = 8.3 Hz, 2H), 7.38 (d, *J* = 8.3 Hz, 2H), 4.47 (dd, *J* = 4.6, 2.3 Hz, 1H), 4.14 – 4.00 (m, 1H), 3.84 (s, 3H), 3.43 – 3.18 (m, 1H), 2.72 – 2.58 (m, 1H), 2.54 (td, *J* = 8.9, 7.6, 4.1 Hz, 1H).
^{13}C NMR (176 MHz, $CDCl_3$) δ 172.50, 136.17, 131.93, 128.86, 122.81, 118.69, 64.01, 58.49, 52.86, 35.73, 33.82.
HR-MS calculated for $C_{13}H_{14}O_2N_2{}^{79}Br$ = 309.0233 [M+H]$^+$, found: 309.0241; calculated for $C_{13}H_{14}O_2N_2{}^{81}Br$ = 311.0213 [M+H]$^+$, found: 311.0214.
IR ν_{max} (cm^{-1}) 2253, 1631, 1444, 1375, 1039, 918.

rac-Methyl (2*R*,4*S*,5*S*)-5-(4-bromophenyl)-4-cyanopyrrolidine-2-carboxylate (Compound **A18**)

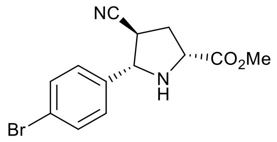

Compound A18 is prepared using the modified procedure A. AgOAc (0.2 eq)/PPh_3 (0.22 eq)/Et_3N (0.5 eq)/CH_2Cl_2/rt.
Isolated yield: 17% (silica gel, 1:10 = acetone:petroleum ether)
^{1}H NMR (700 MHz, $CDCl_3$) δ 7.51 (d, *J* = 8.4 Hz, 2H), 7.40 (d, *J* = 8.4 Hz, 2H), 4.37 (d, *J* = 9.3 Hz, 1H), 4.23 – 4.05 (m, 1H), 3.79 (s, 3H), 2.82 (q, *J* = 9.1 Hz, 1H), 2.72 – 2.54 (m, 1H), 2.55 – 2.46 (m, 1H).
^{13}C NMR (176 MHz, $CDCl_3$) δ 173.41, 137.62, 132.12, 128.42, 122.71, 119.29, 66.49, 58.36, 52.67, 36.37, 33.98.
HR-MS calculated for $C_{13}H_{14}O_2N_2\,^{79}Br$ = 309.0233 $[M+H]^+$, found: 309.0240; calculated for $C_{13}H_{14}O_2N_2\,^{81}Br$ = 311.0213 $[M+H]^+$, found: 311.0214.
IR ν_{max} (cm^{-1}) 2253, 1375, 1037, 918.

rac-Trimethyl (2*R*,3*R*,5*R*)-5-(4-bromophenyl)-3-phenylpyrrolidine-2,4,4-tricarboxylate (Compound **A19**)

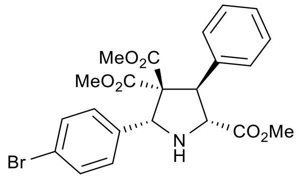

Compound A19 is prepared using procedure A.
Isolated yield: 78% (silica gel, 1:5 = acetone:petroleum ether)
^{1}H NMR (500 MHz, $CDCl_3$) δ 7.46 (d, *J* = 8.5 Hz, 2H), 7.37 (d, *J* = 8.4 Hz, 2H), 7.34 – 7.29 (m, 5H), 5.29 (s, 1H), 4.41 (d, *J* = 6.6 Hz, 1H), 4.23 (d, *J* = 6.6 Hz, 1H), 3.78 (s, 3H), 3.20 (s, 3H), 3.17 ppm (s, 3H).
^{13}C NMR (126 MHz, $CDCl_3$) δ 173.22, 169.73, 169.14, 138.52, 137.46, 131.47, 129.39, 128.90, 128.59, 127.90, 122.25, 71.02, 67.51, 66.12, 55.96, 52.70, 52.43, 52.12 ppm.
HR-MS calculated $C_{22}H_{23}\,^{79}BrNO_6$ = 476.0703 $[M+H]^+$, found: 476.0706; calculated $C_{22}H_{23}\,^{81}BrNO_6$ = 478.0682 $[M+H]^+$, found: 478.0682.
IR ν_{max} (cm^{-1}) 1729, 1434, 1275, 1267, 1211, 1009.

rac-Dimethyl (2*R*,4*R*,5*R*)-5-(4-bromophenyl)-4-methylpyrrolidine-2,4-dicarboxylate (Compound **A20**)

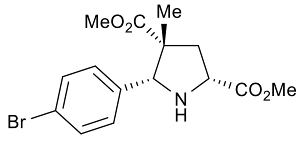

Compound A20 is prepared using procedure A.
Isolated yield: 55% (silica gel, 1:10 = acetone:petroleum ether)
^{1}H NMR (500 MHz, $CDCl_3$) δ 7.45 (d, *J* = 8.5 Hz, 2H), 7.20 (d, *J* = 8.3 Hz, 2H), 4.13 – 4.01 (m, 3H), 3.85 (s, 3H), 3.30 (s, 3H), 2.74 (dd, *J* = 13.3, 7.0 Hz, 1H), 2.13 (dd, *J* = 13.3, 9.0 Hz, 1H), 1.42 ppm (s, 3H).
^{13}C NMR (126 MHz, $CDCl_3$) δ 174.50, 174.20, 137.92, 131.30, 128.50, 121.80, 73.14, 58.82, 54.55, 52.36, 51.57, 41.12, 22.57 ppm.
HR-MS calculated $C_{15}H_{19}\,^{79}BrNO_4$ = 356.0492 $[M+H]^+$, found: 356.0502; calculated $C_{15}H_{19}\,^{81}BrNO_4$ = 358.0472 $[M+H]^+$, found: 358.0477.
IR ν_{max} (cm^{-1}) 1729, 1487, 1433, 1210, 1141, 1109, 1009, 826.

rac-Methyl (2*R*,4*R*,5*S*)-4-acetyl-2-methyl-5-phenylpyrrolidine-2-carboxylate (Compound **A21**)

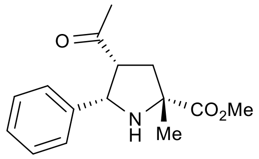

Compound A21 is prepared using procedure A.
Isolated yield: 51% (silica gel, 1:10 = acetone:petroleum ether)
^{1}H NMR (500 MHz, CDCl$_3$) δ 7.49 – 7.42 (m, 2H), 7.40 – 7.27 (m, 3H), 4.41 (d, *J* = 8.3 Hz, 1H), 3.76 (s, 3H), 3.12 (ddd, *J* = 9.9, 8.3, 6.8 Hz, 1H), 2.63 (dd, *J* = 13.3, 6.9 Hz, 1H), 2.13 (dd, *J* = 13.2, 9.9 Hz, 1H), 1.98 (s, 3H), 1.50 ppm (s, 3H).
^{13}C NMR (126 MHz, CDCl$_3$) δ 207.87, 177.39, 128.89, 128.55, 127.70, 127.13, 65.23, 64.15, 59.45, 52.59, 38.75, 30.19, 26.86 ppm.
HR-MS calculated $C_{15}H_{20}NO_3$ = 262.1438 [M+H]$^+$, found: 262.1442.
IR ν$_{max}$ (cm^{-1}) 1729, 1605, 1493, 1455, 1360, 1265, 1174, 1105, 1028, 982, 913, 818.

rac-Dimethyl (2'*R*,4'*R*,5'*S*)-5'-(4-bromophenyl)spiro[fluorene-9,3'-pyrrolidine]-2',4'-dicarboxylate (Compound **A22**)

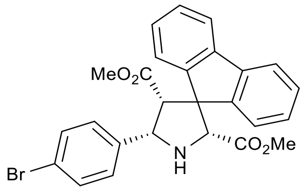

Compound A22 is prepared using procedure A.
Isolated yield: 67% (silica gel, 1:10 = acetone:petroleum ether)
^{1}H NMR (500 MHz, CDCl$_3$) δ 7.63 – 7.55 (m, 2H), 7.53 – 7.49 (m, 1H), 7.46 – 7.41 (m, 1H), 7.35 (dtd, *J* = 19.8, 7.4, 1.3 Hz, 2H), 7.21 – 7.13 (m, 2H), 7.01 – 6.92 (m, 2H), 6.73 – 6.55 (m, 2H), 4.91 (d, *J* = 8.0 Hz, 1H), 4.85 (s, 1H), 3.86 (s, 3H), 3.78 (d, *J* = 8.0 Hz, 1H), 3.27 ppm (s, 3H).
^{13}C NMR (126 MHz, CDCl$_3$) δ 171.88, 141.63, 130.26, 128.31, 128.06, 127.62, 127.40, 127.12, 125.45, 124.15, 119.95, 119.39, 70.84, 62.57, 61.01, 56.23, 52.59, 51.80 ppm.
HR-MS calculated $C_{26}H_{23}{}^{79}BrNO_4$ = 492.0805 [M+H]$^+$, found: 492.0807; calculated $C_{26}H_{23}{}^{81}BrNO_4$ = 494.0785 [M+H]$^+$, found: 494.0784.
IR ν$_{max}$ (cm^{-1}) 1733, 1487, 1449, 1436, 1207, 1176, 1010, 825.

rac-Methyl (1*R*,2*R*,2'*R*,4'*R*,5'*R*)-2'-(4-bromophenyl)-3-oxo-4'-phenylspiro[bicyclo[2.2.1]heptane-2,3'-pyrrolidine]-5'-carboxylate (Compound **A23**)

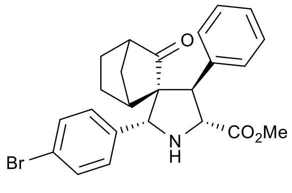

Compound A23 is prepared using procedure A.
Isolated yield: 73% (silica gel, 1:10 = acetone:petroleum ether)
^{1}H NMR (500 MHz, CDCl$_3$) δ 7.85 – 7.44 (m, 2H), 7.44 – 7.34 (m, 4H), 7.29 (dd, *J* = 8.5, 6.7 Hz, 3H), 4.62 (s, 1H), 3.93 (d, *J* = 3.8 Hz, 1H), 3.85 (s, 3H), 3.64 (d, *J* = 3.8 Hz, 1H), 2.38 (d, *J* = 2.4 Hz, 1H), 2.33 – 2.21 (m, 1H), 1.78 – 1.59 (m, 1H), 1.53 – 1.40 (m, 2H), 1.33 – 1.16 (m, 2H), 1.02 ppm (ddd, *J* = 10.7, 1.8, 0.9 Hz, 1H).
^{13}C NMR (126 MHz, CDCl$_3$) δ 173.33, 173.32, 142.44, 138.89, 131.99, 130.38, 128.87, 128.75, 127.19, 122.47, 71.87, 68.40, 67.39, 56.64, 52.58, 50.36, 43.07, 34.51, 26.54, 24.94 ppm.

HR-MS calculated $C_{24}H_{25}{}^{79}BrNO_3$ = 454.1012 [M+H]$^+$, found: 454.1014; calculated $C_{24}H_{25}{}^{81}BrNO_3$ = 456.0992 [M+H]$^+$, found: 456.0990;
IR ν_{max} (cm^{-1}) 1733, 1489, 1434, 1216, 1134, 1073, 1010, 912, 825.

rac-Methyl (2*R*,2'*R*,4'*R*,5'*R*)-2'-(4-bromophenyl)-1-oxo-4'-phenyl-1,3-dihydrospiro[indene-2,3'-pyrrolidine]-5'-carboxylate (Compound **A24**)

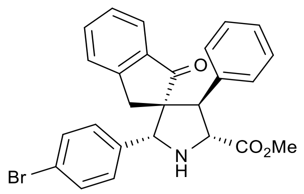

Compound A24 is prepared using procedure A.
Isolated yield: 53% (silica gel, 1:10 = acetone:petroleum ether)
^{1}H NMR (500 MHz, CDCl$_3$) δ 7.44 – 7.34 (m, 3H), 7.34 – 7.30 (m, 2H), 7.30 – 7.27 (m, 2H), 7.19 (d, *J* = 8.4 Hz, 2H), 7.16 – 7.10 (m, 2H), 7.04 (d, *J* = 8.5 Hz, 2H), 4.52 (s, 1H), 4.36 (d, *J* = 6.0 Hz, 1H), 3.98 (d, *J* = 6.1 Hz, 1H), 3.84 (s, 3H), 2.89 (d, *J* = 17.5 Hz, 1H), 2.77 ppm (d, *J* = 17.6 Hz, 1H).
^{13}C NMR (126 MHz, CDCl$_3$) δ 207.57, 173.18, 151.74, 140.91, 135.89, 135.59, 134.80, 131.17, 128.98, 128.78, 128.49, 127.46, 127.42, 125.56, 123.48, 121.86, 73.44, 67.38, 65.62, 55.89, 52.55, 35.58 ppm.
HR-MS calculated $C_{26}H_{23}{}^{79}BrNO_3$ = 476.0856 [M+H]$^+$, found: 476.0858; calculated $C_{26}H_{23}{}^{81}BrNO_3$ = 478.0835 [M+H]$^+$, found: 478.0833.
IR ν_{max} (cm^{-1}) 1734, 1700, 1653, 1616, 1559, 1436, 1275, 1261.

rac-Methyl (2'*R*,3*R*,4'*R*,5'*R*)-2'-(4-bromophenyl)-4-oxo-4'-phenylspiro[chromane-3,3'-pyrrolidine]-5'-carboxylate (Compound **A25**)

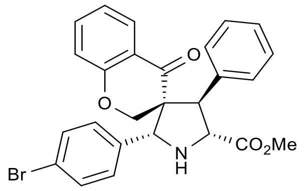

Compound A25 is prepared using procedure A.
Isolated yield: 46% (silica gel, 1:5 = acetone:petroleum ether)
^{1}H NMR (500 MHz, CDCl$_3$) δ 7.49 (dd, *J* = 7.9, 1.7 Hz, 1H), 7.35 (d, *J* = 4.3 Hz, 4H), 7.32 – 7.27 (m, 2H), 7.15 (d, *J* = 8.5 Hz, 2H), 7.03 (d, *J* = 8.5 Hz, 2H), 6.81 (ddd, *J* = 8.1, 7.2, 1.1 Hz, 1H), 6.67 (dd, *J* = 8.4, 1.0 Hz, 1H), 4.88 (s, 1H), 4.52 (d, *J* = 8.2 Hz, 1H), 4.47 (d, *J* = 8.1 Hz, 1H), 4.17 (dd, *J* = 12.0, 12.0 Hz, 1H), 3.84 (dd, *J* = 12.0 , 12.0 Hz, 1H), 3.80 ppm (s, 3H).
^{13}C NMR (126 MHz, CDCl$_3$) δ 192.36, 172.81, 160.34, 136.28, 135.95, 135.88, 131.00, 129.54, 128.91, 128.61, 127.91, 127.31, 122.16, 121.45, 120.97, 117.15, 72.77, 69.43, 64.08, 59.36, 52.87, 51.77 ppm.
HR-MS calculated $C_{26}H_{23}{}^{79}BrNO_4$ = 492.0805 (M+H+), found: 492.0808; calculated $C_{26}H_{23}{}^{81}BrNO_4$ = 494.0785 (M+H+), found: 494.0785.
IR ν_{max} (cm^{-1}) 1737, 1686, 1605, 1478, 1295, 1213, 1010, 913.

Methyl (1*S*,3*S*,5*R*,10*S*)-1-(4-bromophenyl)-7-methyl-6-oxo-10-(prop-1-en-2-yl)-2-azaspiro[4.5]dec-7-ene-3-carboxylate (Compound **A26**)

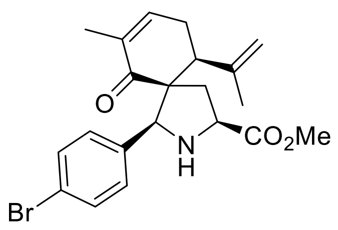

Compound A26 is prepared using procedure A.
Isolated yield: 41% (silica gel, 1:5 = acetone:petroleum ether)
^{1}H NMR (500 MHz, CDCl$_3$) δ 7.35 (d, *J* = 8.5 Hz, 2H), 7.08 (d, *J* = 8.5 Hz, 2H), 6.20 (ddt, *J* = 5.5, 2.7, 1.3 Hz, 1H), 4.75 (t, *J* = 1.7 Hz, 1H), 4.71 (d, *J* = 1.8 Hz, 1H), 4.33 (s, 1H), 3.94 (dd, *J* = 8.7, 7.5 Hz, 1H), 3.85 (s, 3H), 2.98 (d, *J* = 6.9 Hz, 1H), 2.74 – 2.62 (m, 1H), 2.17 (dd, *J* = 20.2, 5.6 Hz, 1H), 2.10 (dd, *J* = 13.5, 8.6 Hz, 1H), 1.53 (s, 3H), 1.52 – 1.40 ppm (m, 3H).
^{13}C NMR (126 MHz, CDCl$_3$) δ 199.64, 174.69, 146.84, 140.96, 138.88, 136.32, 131.67, 131.09, 129.49, 128.81, 122.00, 114.38, 72.47, 60.08, 58.62, 52.46, 51.15, 36.66, 28.93, 21.40, 16.23 ppm.
HR-MS calculated $C_{21}H_{25}{}^{79}BrNO_3$ = 418.1012 [M+H]$^+$, found: 418.1015; calculated $C_{21}H_{25}{}^{81}BrNO_3$ = 420.0992 [M+H]$^+$, found: 420.0993.
$[\alpha]_D^{20}\ -76\ (c\ 0.10, CHCl_3)$
IR ν$_{max}$ (cm^{-1}) 1737, 1665, 1488, 1434, 1376, 1261, 1206, 1073, 1009, 901, 816.

Methyl (1*S*,2'*R*,3a*R*,5a*S*,5'*R*,9a*S*,9b*R*)-2'-(4-bromophenyl)-3a,6,6,9a-tetramethyl-2-oxodecahydro-2*H*-spiro[naphtho[2,1-*b*]furan-1,3'-pyrrolidine]-5'-carboxylate (Compound **A27**)

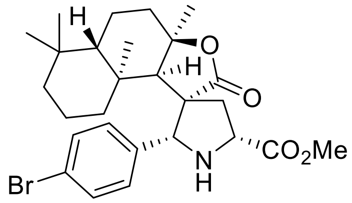

Compound A27 is prepared using procedure A.
Isolated yield: 70% (silica gel, 1:5 = acetone:petroleum ether)
^{1}H NMR (500 MHz, CDCl$_3$) ^{1}H NMR (500 MHz, CDCl3) δ 7.48 (d, *J* = 8.5 Hz, 2H), 7.30 (d, *J* = 8.5 Hz, 2H), 4.39 (s, 1H), 4.08 (dd, *J* = 10.0, 4.2 Hz, 1H), 3.81 (s, 3H), 2.84 (dd, *J* = 13.6, 10.1 Hz, 1H), 2.53 (dd, *J* = 13.5, 4.4 Hz, 1H), 2.16 (s, 1H), 1.92 (s, 2H), 1.86 – 1.76 (m, 2H), 1.76 – 1.67 (m, 1H), 1.57 (dt, *J* = 14.1, 3.5 Hz, 1H), 1.53 – 1.42 (m, 1H), 1.40 (s, 3H), 1.39 – 1.32 (m, 2H), 1.30 – 1.19 (m, 1H), 1.15 (s, 3H), 1.01 – 0.92 (m, 1H), 0.87 (s, 3H), 0.84 ppm (s, 3H).
^{13}C NMR (126 MHz, CDCl$_3$) δ 178.99, 172.76, 134.54, 131.84, 130.62, 122.80, 85.13, 70.94, 59.19, 58.24, 56.95, 52.70, 42.07, 40.73, 39.13, 38.91, 35.51, 33.61, 33.45, 31.08, 25.95, 21.22, 20.63, 18.22, 17.68 ppm.
HR-MS calculated $C_{27}H_{36}{}^{79}BrNO_4$ = 518.1901 [M+H]$^+$, found: 518.1914; calculated $C_{27}H_{36}{}^{81}BrNO_4$ = 520.1880 [M+H]$^+$, found: 520.1891.
$[\alpha]_D^{20}\ -112\ (c\ 0.10, CHCl_3)$
IR ν$_{max}$ (cm^{-1}) 1753, 1435, 1391, 1315, 1282, 1203, 1174, 1124, 1078, 1041, 1022, 1010, 942, 912, 819.

rac-Dimethyl (1*R*,3*R*,4*R*,5*S*,7*R*,8*S*,10*S*,11*R*)-1,8-bis(4-bromophenyl)-6-oxo-4,11-diphenyl-2,9-diazadispiro[4.1.4$^{7.15}$]dodecane-3,10-dicarboxylate (Compound **A28**)

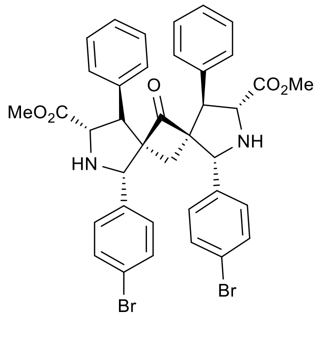

Compound A28 is prepared using procedure B.
Yield: 40% (silica gel, 1:5 = acetone:petroleum ether)
^{1}H NMR (500 MHz, CDCl$_3$) δ 7.56 (d, *J* = 8.4 Hz, 4H), 7.28 (d, *J* = 8.5 Hz, 6H), 7.25 – 7.21 (m, 4H), 6.81 – 6.77 (m, 4H), 4.25 (s, 2H), 3.93 (s, 1H), 3.83 (s, 6H), 3.22 (s, 1H), 2.57 (d, *J* = 2.7 Hz, 2H), 1.58 ppm (s, 2H).
^{13}C NMR (126 MHz, CDCl$_3$) δ 212.27, 172.53, 138.81, 132.09, 130.95, 128.71, 128.57, 127.72, 127.41, 123.00, 71.95, 69.64, 66.45, 52.66, 52.18, 22.42 ppm.

HR-MS calculated $C_{38}H_{35}{}^{79}Br^{81}BrN_2O_5$ = 759.0887 [M+H]$^+$, found: : 759.0902; calculated $C_{38}H_{35}{}^{81}Br^{81}BrN_2O_5$ = 761.0866 [M+H]$^+$, found : 761.0886.
IR ν$_{max}$ (cm^{-1}) 1739, 1493, 1434, 1222, 1011, 914.

rac-Dimethyl (1*R*,3*R*,4*R*,5*R*,7*S*,8*S*,10*S*,11*S*)-1,8-bis(4-bromophenyl)-6-oxo-4,11-diphenyl-2,9-diazadispiro[4.1.4$^{7.25}$]tridecane-3,10-dicarboxylate (Compound **A29**)

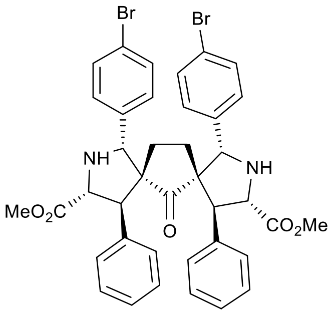

Compound A29 is prepared using procedure B.
Yield: 57% (silica gel, 1:5 = acetone:petroleum ether)
^{1}H NMR (700 MHz, CD_2Cl_2) δ 7.59 (d, J = 8.5 Hz, 4H), 7.40 – 7.20 (m, 9H), 7.20 – 7.11 (m, 2H), 6.56 (s, 3H), 4.18 (s, 2H), 3.89 (s, 6H), 3.85 (d, J = 3.4 Hz, 2H), 2.75 (d, J = 3.4 Hz, 2H), 1.16 – 0.97 ppm (m, 4H).
^{13}C NMR (176 MHz, CD_2Cl_2) δ 221.29, 174.02, 141.19, 138.07, 132.60, 130.94, 129.11, 128.89, 127.48, 123.28, 73.78, 67.85, 65.60, 56.27, 52.75, 29.07 ppm.
HR-MS calculated $C_{39}H_{37}{}^{79}Br^{79}BrN_2O_5$ = 771.1064 [M+H]$^+$, found: 771.1085; calculated $C_{39}H_{37}{}^{79}Br^{81}BrN_2O_5$ = 773.1043 [M+H]$^+$, found: 773.1066; calculated $C_{39}H_{37}{}^{81}Br^{81}BrN_2O_5$ = 775.1023 [M+H]$^+$, found: 775.1023.
IR ν$_{max}$ (cm^{-1}) 1733, 1491, 1454, 1434, 1219, 1145, 1075, 1009, 953, 901, 814.

Characterization data for cyclobutanes

Dimethyl (1*R*,2*s*,3*S*,4*s*)-2-(4-bromophenyl)-4-phenylcyclobutane-1,3-dicarboxylate (Compound **5**)

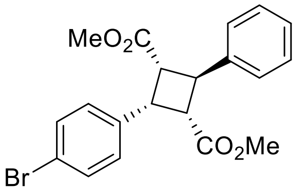

Compound 5 is prepared using procedure C.
Isolated yield: 69% (silica gel, 1:50 = acetone:petroleum ether)
^{1}H NMR (500 MHz, CDCl$_3$) δ 7.42 (d, J = 8.5 Hz, 2H), 7.38 – 7.30 (m, 4H), 7.28 – 7.23 (m, 1H), 7.16 (d, J = 8.5 Hz, 2H), 4.74 (dd, *J* = 10.7, 10.7 Hz, 1H), 4.16 (dd, *J* = 10.1, 10.1 Hz, 1H), 3.70 (dd, *J* = 10.4, 10.4 Hz, 2H), 3.40 ppm (s, 6H).
^{13}C NMR (126 MHz, CDCl$_3$) δ 171.47, 141.46, 136.57, 131.46, 129.91, 128.65, 126.97, 126.41, 121.31, 51.75, 46.23, 43.86, 41.74 ppm.
HR-MS calculated for $C_{20}H_{20}{}^{79}BrO_4$ = 403.0540 [M+H]$^+$, found: 403.0542; calculated for $C_{20}H_{20}{}^{81}BrO_4$ = 405.0519 [M+H]$^+$, found: 405.0520;
IR ν_{max} (cm^{-1}) 1739, 1723, 1490, 1433, 1337, 1225, 1204, 1180, 1161, 1107, 1075, 1045, 1008, 942, 837.

Dimethyl (1*R*,2*r*,3*S*,4*s*)-2,4-diphenylcyclobutane-1,3-dicarboxylate (Compound **8**)

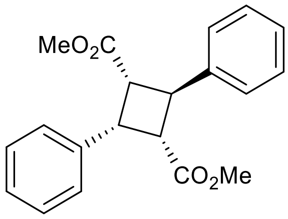

Compound 8 is prepared using procedure C.
Isolated yield: 67% (silica gel, 1:20 = acetone:petroleum ether)
^{1}H NMR (500 MHz, CDCl$_3$) δ 7.35 (d, *J* = 5.7 Hz, 4H), 7.31 – 7.26 (m, 4H), 7.26 – 7.20 (m, 2H), 4.79 (dd, *J* = 10.7, 10.7, 1H), 4.20 (m, 1H), 3.69 (dd, *J* = 10.4, 10.4 Hz, 2H), 3.36 ppm (s, 6H).
^{13}C NMR (126 MHz, CDCl$_3$) δ 171.75, 141.80, 137.46, 128.61, 128.33, 128.12, 127.26, 126.84, 126.45, 51.59, 46.43, 44.45, 41.76 ppm.
HR-MS calculated for $C_{20}H_{21}O_4$ = 325.1434 [M+H]$^+$, found: 325.1440.
IR ν_{max} (cm^{-1}) 1732, 1496, 1435, 1327, 1265, 1204.

Dimethyl (1*R*,2*s*,3*S*,4*s*)-2-phenyl-4-(p-tolyl)cyclobutane-1,3-dicarboxylate (Compound **9**)

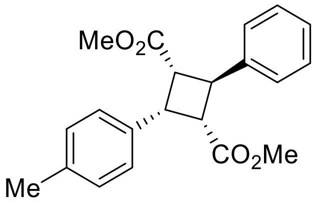

Compound 9 is prepared using procedure C.
Isolated yield: 51% (silica gel, 5% acetone in petroleum ether)
^{1}H NMR (500 MHz, CDCl$_3$) δ 7.34 (d, *J* = 6.0 Hz, 4H), 7.25 – 7.23 (m, 1H), 7.16 (d, *J* = 8.2 Hz, 2H), 7.08 (d, *J* = 8.1 Hz, 2H), 4.78 (dd, *J* = 10.7, 10.7 Hz, 1H), 4.17 (dd, *J* = 10.1, 10.1 Hz, 1H), 3.67 (dd, *J* = 10.4, 10.4 Hz, 2H), 3.38 (s, 6H), 2.30 ppm (s, 3H).

^{13}C NMR (126 MHz, CDCl$_3$) δ 171.81, 141.86, 136.76, 134.25, 129.02, 128.59, 127.99, 126.81, 126.46, 51.60, 46.47, 44.16, 41.70, 21.11 ppm.
HR-MS calculated for $C_{21}H_{23}O_4$ = 339.1591 [M+H]$^+$, found: 339.1596.
IR ν$_{max}$ (cm^{-1}) 1730, 1484, 1429, 1410, 1369, 1335, 1279, 1233, 1205, 1180, 1121, 1009, 938, 839.

Dimethyl (1*R*,2*s*,3*S*,4*s*)-2-(4-chlorophenyl)-4-phenylcyclobutane-1,3-dicarboxylate (Compound **10**)

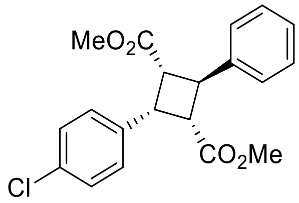

Compound 10 is prepared using procedure C.
Isolated yield: 51% (silica gel, 1:50 = acetone:petroleum ether)
1H NMR (500 MHz, CDCl$_3$) δ 7.35 – 7.23 (m, 4H), 7.22 – 7.12 (m, 5H), 4.68 (dd, *J* = 10.7, 10.7 Hz, 1H), 4.11 (dd, *J* = 10.1, 10.1 Hz, 1H), 3.64 (dd, *J* = 10.4, 10.4 Hz, 2H), 3.32 ppm (s, 6H).
13C NMR (126 MHz, CDCl$_3$) δ 171.60, 141.58, 136.16, 133.24, 129.67, 128.76, 128.61, 127.06, 126.51, 60.73, 51.83, 46.40, 43.89, 41.83 ppm.
HR-MS calculated for $C_{20}H_{20}{}^{35}ClO_4$ = 359.1044 [M+H]$^+$, found: 359.1052; calculated for $C_{20}H_{20}{}^{37}ClO_4$ = 361.1015 [M+H]$^+$, found: 361.1023;
IR ν$_{max}$ (cm^{-1}) 1731, 1495, 1435, 1329, 1205, 1094, 1045, 1013, 838.

Dimethyl (1*R*,2*s*,3*S*,4*s*)-2-(4-fluorophenyl)-4-phenylcyclobutane-1,3-dicarboxylate (Compound **11**)

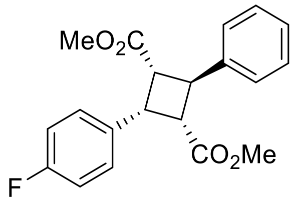

Compound 11 is prepared using procedure C.
Isolated yield: 65% (silica gel, 1:50 = acetone:petroleum ether)
^{1}H NMR (500 MHz, CDCl$_3$) δ 7.35 – 7.23 (m, 4H), 7.22 – 7.15 (m, 3H), 6.92 (m, 2H), 4.69 (dd, J = 10.7, 10.7 Hz, 1H), 4.13 (dd, J = 10.1, 10.1 Hz, 1H), 3.61 (dd, J = 10.4, 10.4 Hz, 2H), 3.32 ppm (s, 6H).
^{13}C NMR (126 MHz, CDCl$_3$) δ 171.59, 162.96, 161.00, 141.55, 133.29, 133.26, 129.85, 129.79, 128.64, 126.93, 126.42, 115.31, 115.14, 51.68, 46.42, 43.67, 41.65 ppm.
^{19}F NMR (470 MHz, CDCl$_3$) δ -115.18.
HR-MS calculated for $C_{20}H_{20}FO_4$ = 343.1340 [M+H]$^+$, found: 343.1346.
IR ν$_{max}$ (cm^{-1}) 1732, 1606, 1512, 1436, 1333, 1204, 1163, 1101, 1045, 940, 842.

Dimethyl (1*R*,2*s*,3*S*,4*s*)-2-(4-isopropylphenyl)-4-phenylcyclobutane-1,3-dicarboxylate (Compound **12**)

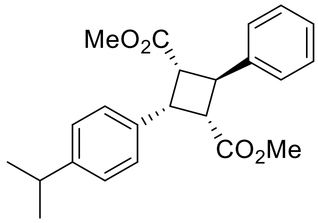

Compound 12 is prepared using procedure C.
Isolated yield: 51% (silica gel, 1:50 = acetone:petroleum ether)
^{1}H NMR (500 MHz, CDCl$_3$) δ 7.39 – 7.31 (m, 4H), 7.26 – 7.22 (m, 1H), 7.18 (d, *J* = 8.3 Hz, 2H), 7.13 (d, *J* = 8.3 Hz, 2H), 4.78 (dd, *J* = 10.7, 10.7 Hz, 1H), 4.18 (dd, *J* = 10.1, 10.1 Hz, 1H), 3.66 (dd, *J* = 10.4, 10.4 Hz, 2H), 3.35 (s, 6H), 2.86 (p, *J* = 6.9 Hz, 1H), 1.22 (s, 3H), 1.20 ppm (s, 3H).
^{13}C NMR (126 MHz, CDCl$_3$) δ 171.85, 147.73, 141.88, 134.58, 128.59, 128.02, 126.79, 126.46, 126.29, 51.55, 46.49, 44.16, 41.64, 33.66, 23.93 ppm.
HR-MS calculated for $C_{23}H_{27}O_4$ = 367.1904 [M+H]$^+$, found: 367.1908.
IR ν$_{max}$ (cm^{-1}) 1735, 1436, 1271, 1205, 835.

Dimethyl (1*R*,2*s*,3*S*,4*s*)-2-(3-methoxyphenyl)-4-phenylcyclobutane-1,3-dicarboxylate (Compound **13**)

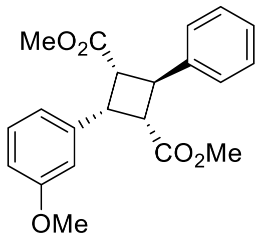

Compound 13 is prepared using procedure C.
Isolated yield: 42% (silica gel, 1:50 = acetone:petroleum ether to 1:20 = acetone:petroleum ether)
^{1}H NMR (500 MHz, CDCl$_3$) δ 7.38 – 7.30 (m, 4H), 7.26 (s, 1H), 7.20 (t, *J* = 7.9 Hz, 1H), 6.90 – 6.73 (m, 3H), 4.79 (dd, *J* = 10.7, 10.7 Hz, 1H), 4.17 (dd, *J* = 10.2, 10.2 Hz, 1H), 3.80 (s, 3H), 3.68 (dd, *J* = 10.4, 10.4 Hz, 2H), 3.39 ppm (s, 6H).
^{13}C NMR (126 MHz, CDCl$_3$) δ 171.87, 159.53, 141.90, 139.13, 129.44, 128.73, 126.97, 126.55, 120.43, 113.79, 113.18, 55.30, 51.79, 46.51, 44.64, 41.89 ppm.
HR-MS calculated for $C_{21}H_{23}O_5$ = 355.1540 [M+H]$^+$, found: 355.1544.
IR ν$_{max}$ (cm^{-1}) 1729, 1602, 1584, 1489, 1454, 1394, 1328, 1269, 1254, 1203, 1182, 1089, 1041, 874.

Dimethyl (1*R*,2*s*,3*S*,4*s*)-2-(2-methoxyphenyl)-4-phenylcyclobutane-1,3-dicarboxylate (Compound **14**)

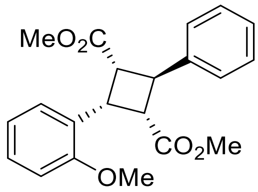

Compound 14 is prepared using procedure C.
Isolated yield: 44% (silica gel, 1:50 = acetone:petroleum ether to 1:20 = acetone:petroleum ether)
^{1}H NMR (500 MHz, CDCl$_3$) δ 7.34 – 7.23 (m, 4H), 7.20 – 7.13 (m, 2H), 7.10 (ddd, *J* = 8.2, 7.4, 1.7 Hz, 1H), 6.80 (td, *J* = 7.5, 1.2 Hz, 1H), 6.74 (dd, *J* = 8.2, 1.1 Hz, 1H), 4.68 (dd, *J* = 10.3, 10.3 Hz, 1H), 4.59 (dd, *J* = 10.6, 10.6 Hz, 1H), 3.70 (s, 3H), 3.57 (dd, *J* = 10.4, 10.4 Hz, 2H), 3.29 ppm (s, 6H).
^{13}C NMR (126 MHz, CDCl$_3$) δ 172.27, 157.37, 142.43, 128.43 (d, J = 32.9 Hz), 126.67, 126.51, 126.04, 120.46, 110.40, 55.30, 51.47, 45.87, 42.06 ppm.
HR-MS calculated for $C_{21}H_{23}O_5$ = 355.1540 [M+H]$^+$, found: 355.1544.
IR ν$_{max}$ (cm^{-1}) 1728, 1601, 1494, 1457, 1435, 1329, 1294, 1244, 1203, 1115, 1029.

Dimethyl (1*R*,2*s*,3*S*,4*s*)-2-(2-chlorophenyl)-4-phenylcyclobutane-1,3-dicarboxylate (Compound **15**)

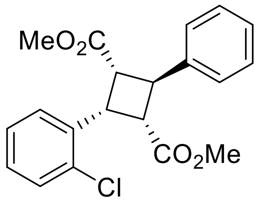

Compound 15 is prepared using procedure C.
Isolated yield: 88% (silica gel, 1:50 = acetone:petroleum ether)
^{1}H NMR (500 MHz, CDCl$_3$) δ 7.43 (dd, *J* = 7.9, 1.6 Hz, 1H), 7.38 (dd, *J* = 7.9, 1.4 Hz, 1H), 7.35 (d, *J* = 5.2 Hz, 4H), 7.27 (d, *J* = 3.1 Hz, 1H), 7.25 – 7.20 (m, 1H), 7.17 (td, *J* = 7.7, 1.7 Hz, 1H), 4.91 (dd, *J* = 10.3, 10.3 Hz, 1H), 4.75 (dd, *J* = 10.8, 10.8 Hz, 1H), 3.75 (dd, *J* = 10.5, 10.5 Hz, 2H), 3.39 ppm (s, 6H).
^{13}C NMR (126 MHz, CDCl$_3$) δ 171.70, 135.18, 134.93, 129.74, 128.66, 128.32, 127.50, 126.97, 126.74, 126.49, 51.67, 45.80, 41.90, 39.28 ppm.
HR-MS calculated for $C_{20}H_{20}{}^{35}ClO_4$ = 359.1045 [M+H]$^+$, found: 359.1052; calculated for $C_{20}H_{20}{}^{37}ClO_4$ = 361.1015 [M+H]$^+$, found: 361.1022.
IR ν$_{max}$ (cm^{-1}) 1730, 1497, 1478, 1436, 1328, 1202, 1091, 1062, 1040.

Dimethyl (1*R*,2*s*,3*S*,4*s*)-2-(2-bromophenyl)-4-phenylcyclobutane-1,3-dicarboxylate (Compound **16**)

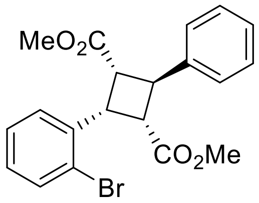

Compound 16 is prepared using procedure C.
Isolated yield: 68% (silica gel, 1:50 = acetone:petroleum ether)
^{1}H NMR (500 MHz, CDCl$_3$) δ 7.49 (dd, *J* = 8.0, 1.4 Hz, 1H), 7.34 (dd, *J* = 7.9, 1.7 Hz, 1H), 7.27 (d, *J* = 2.1 Hz, 1H), 7.22 – 7.15 (m, 4H), 7.00 (ddd, *J* = 8.0, 7.3, 1.6 Hz, 2H), 4.80 (td, *J* = 10.3, 10.27, 1.1 Hz, 1H), 4.66 (dd, *J* = 10.7, 10.7 Hz, 1H), 3.66 (dd, *J* = 10.5, 10.5 Hz, 2H), 3.31 ppm (s, 6H).
^{13}C NMR (126 MHz, CDCl$_3$) δ 171.67, 141.47, 136.81, 133.14, 128.66, 128.61, 127.50, 127.39, 126.97, 126.49, 125.93, 51.66, 45.86, 42.35, 41.82 ppm.
HR-MS calculated for $C_{20}H_{20}{}^{79}BrO_4$ = 403.0540 [M+H]$^+$, found: 403.0542; calculated for $C_{20}H_{20}{}^{81}BrO_4$ = 405.0519 [M+H]$^+$, found: 405.0519.
IR ν$_{max}$ (cm^{-1}) 1729, 1497, 1474, 1434, 1329, 1203, 1042, 1021, 940.

Dimethyl (1*R*,2*s*,3*S*,4*s*)-2-(naphthalen-2-yl)-4-phenylcyclobutane-1,3-dicarboxylate (Compound **17**)

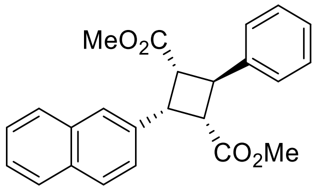

Compound 17 is prepared using procedure C.
Isolated yield: 51% (silica gel, 1:50 = acetone:petroleum ether)
^{1}H NMR (500 MHz, CDCl$_3$) δ 7.84 – 7.75 (m, 4H), 7.49 – 7.45 (m, 2H), 7.44 (dd, *J* = 8.4, 2.0 Hz, 1H), 7.41 – 7.35 (m, 4H), 7.30 – 7.26 (m, 1H), 4.93 (dd, *J* = 10.7, 10.7 Hz, 1H), 4.40 (dd, *J* = 10.1, 10.1 Hz, 1H), 3.77 (dd, *J* = 10.4, 10.4 Hz, 2H), 3.30 ppm (s, 6H).
^{13}C NMR (126 MHz, CDCl$_3$) δ 171.77, 141.80, 135.06, 133.30, 132.55, 128.65, 127.98, 127.95, 127.58, 127.46, 126.90, 126.49, 126.06, 125.92, 125.85, 51.67, 46.53, 44.64, 41.92 ppm.
HR-MS calculated for $C_{24}H_{23}O_4$ = 375.1591 [M+H]$^+$, found: 375.1595.
IR ν$_{max}$ (cm^{-1}) 1733, 1601, 1497, 1435, 1331, 1204, 1047, 860, 824.

Dimethyl (1*R*,2*s*,3*S*,4*s*)-2-(perfluorophenyl)-4-phenylcyclobutane-1,3-dicarboxylate (Compound **18**)

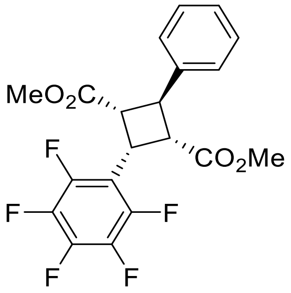

Compound 18 is prepared using procedure C.
Isolated yield: 70% (silica gel, 1:50 = acetone:petroleum ether)
^{1}H NMR (500 MHz, $CDCl_3$) δ 7.42 – 7.36 (m, 4H), 7.32 – 7.27 (m, 1H), 4.69 (dd, *J* = 10.2, 10.2 Hz, 1H), 4.62 (dd, *J* = 10.8, 10.8 Hz, 1H), 3.76 (dd, *J* = 10.5, 10.5 Hz, 2H), 3.57 ppm (s, 6H).
^{13}C NMR (126 MHz, $CDCl_3$) δ 171.37, 141.41, 128.72, 127.17, 126.52, 52.06, 44.54, 43.24, 31.69 ppm.
^{19}F NMR (470 MHz, $CDCl_3$) δ -139.20 (d, *J* = 16.0 Hz), -154.89, -162.36 ppm (d, *J* = 6.8 Hz).
HR-MS calculated for $C_{20}H_{16}F_5O_4$ = 415.0963 $[M+H]^+$, found: 415.0965.
IR ν_{max} (cm^{-1}) 1733, 1523, 1498, 1437, 1323, 1210, 1149, 1063, 1016, 982, 939, 867.

Dimethyl (1*R*,2*s*,3*S*,4*s*)-2-(furan-2-yl)-4-phenylcyclobutane-1,3-dicarboxylate (Compound **19**)

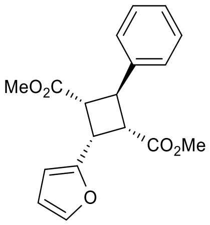

Compound 19 is prepared using procedure C.
Isolated yield: 24% (silica gel, 1:20 = acetone:petroleum ether to 1:10 = acetone:petroleum ether)
^{1}H NMR (500 MHz, $CDCl_3$) δ 7.28 (s, 2H), 7.27 (d, *J* = 1.3 Hz, 2H), 7.26 (dd, *J* = 2.0, 0.8 Hz, 1H), 6.23 (dd, *J* = 3.3, 1.8 Hz, 1H), 6.16 (d, *J* = 3.2 Hz, 1H), 4.67 (t, *J* = 10.7 Hz, 1H), 4.18 (t, *J* = 9.9 Hz, 1H), 3.55 – 3.47 (m, 2H), 3.45 (s, 6H).
^{13}C NMR (126 MHz, $CDCl_3$) δ 171.53, 151.39, 141.91, 141.42, 128.58, 126.90, 126.55, 110.52, 107.92, 51.89, 45.39, 42.15, 37.50.
HR-MS calculated for $C_{18}H_{19}O_5$ = 315.1227 $[M+H]^+$, found: 315.1226.
IR ν_{max} (cm^{-1}) 2159, 2026, 1737, 1439, 1375, 1171, 1039, 919.

Dimethyl (1*R*,2*s*,3*S*,4*s*)-2-phenyl-4-(pyridin-3-yl)cyclobutane-1,3-dicarboxylate (Compound **20**)

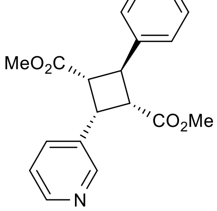

Compound 20 is prepared using procedure C.
Isolated yield: 30% (silica gel, 1:20 = acetone:petroleum ether to 1:10 = acetone:petroleum ether)
^{1}H NMR (500 MHz, $CDCl_3$) δ 8.60 – 8.23 (m, 2H), 7.66 (ddd, *J* = 8.0, 2.4, 1.5 Hz, 1H), 7.35 – 7.24 (m, 4H), 7.24 – 7.06 (m, 2H), 4.68 (t, *J* = 10.7 Hz, 1H), 4.16 (t, *J* = 10.0 Hz, 1H), 3.67 (t, *J* = 10.4 Hz, 2H), 3.32 (s, 3H).
^{13}C NMR (126 MHz, $CDCl_3$) δ 171.25, 150.23, 148.62, 141.15, 134.80, 133.29, 128.71, 127.09, 126.40, 123.14, 51.80, 46.06, 41.92, 41.57.
HR-MS calculated for $C_{19}H_{20}NO_4$ = 326.1387 $[M+H]^+$, found: 326.1389.
IR ν_{max} (cm^{-1}) 2026 ,2012, 1977, 1728, 1437, 1265, 1207

Dimethyl (1*R*,2*s*,3*S*,4*s*)-2-phenyl-4-(thiophen-2-yl)cyclobutane-1,3-dicarboxylate (Compound **21**)

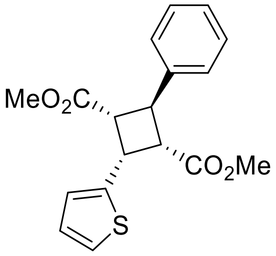

Compound 21 is prepared using procedure C.
Isolated yield: 42% (silica gel, 1:20 = acetone:petroleum ether to 1:10 = acetone:petroleum ether)
^{1}H NMR (500 MHz, $CDCl_3$) δ 7.27 (d, *J* = 6.3 Hz, 4H), 7.18 (d, *J* = 4.9 Hz, 1H), 7.08 (dd, *J* = 5.2, 1.1 Hz, 1H), 6.93 (dd, *J* = 3.6, 1.2 Hz, 1H), 6.88 (dd, *J* = 5.1, 3.6 Hz, 1H), 4.69 (dd, *J* = 10.9, 10.9 Hz, 1H), 4.38 (dd, *J* = 9.8, 9.8 Hz, 1H), 3.59 (dd, *J* = 10.9, 9.6 Hz, 2H), 3.40 ppm (s, 6H).
^{13}C NMR (126 MHz, $CDCl_3$) δ 171.21, 141.17, 139.19, 128.62, 127.20, 126.96, 126.50, 125.48, 124.15, 51.78, 47.13, 41.46, 39.29 ppm.
HR-MS calculated for $C_{18}H_{19}O_4S$ = 331.0999 $[M+H]^+$, found: 331.1004; calculated for $C_{18}H_{18}O_4SNa$ = 353.0818 $[M+Na]^+$, found: 353.0818.
IR ν_{max} (cm^{-1}) 1730, 1497, 1435, 1331, 1204, 1041, 850.

Dimethyl (1*R*,2*s*,3*S*,4*s*)-2-(pyridin-3-yl)-4-(thiophen-3-yl)cyclobutane-1,3-dicarboxylate (Compound **22**)

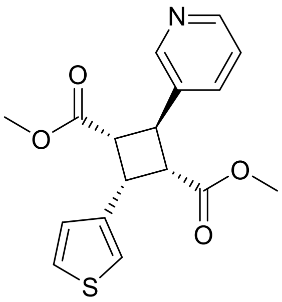

Compound 22 is prepared using procedure C.
Isolated yield: 40% (silica gel, 1:10 = acetone:petroleum ether to 1:5= acetone:petroleum ether)
^{1}H NMR (600 MHz, CD_2Cl_2) δ 8.57 (s, 1H), 8.48 (d, *J* = 3.8 Hz, 1H), 7.65 (d, *J* = 7.9 Hz, 1H), 7.28 (m, 2H), 7.18 (dd, *J* = 2.9, 1.3 Hz, 1H), 7.01 (dd, *J* = 5.0, 1.3 Hz, 1H), 4.69 (t, *J* = 10.8 Hz, 1H), 4.38 (t, *J* = 9.7 Hz, 1H), 3.63 (dd, *J* = 10.8, 9.7 Hz, 2H), 3.42 (s, 6H).
^{13}C NMR (151 MHz, CD_2Cl_2) δ 171.22, 148.34, 148.31, 137.47, 136.68, 133.99, 127.57, 125.42, 123.31, 122.41, 51.60, 45.83, 39.70, 39.48.
HR-MS calculated for C17H18O4NS= = 332.0951, $[M+H]^+$, found: 332.0955
IR ν_{max} (cm^{-1}) 1633, 1445, 1375, 1038, 918.

Dimethyl (1*R*,2*r*,3*S*,4*r*)-2-cyclopropyl-4-phenylcyclobutane-1,3-dicarboxylate (Compound **23**)

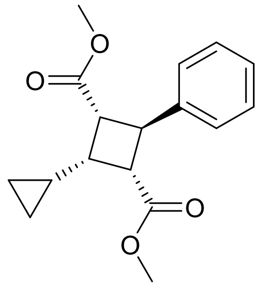

Compound 23 is prepared using procedure C.
Isolated yield: 39% (silica gel, 1:50 = acetone:petroleum ether)
^{1}H NMR (500 MHz, CDCl$_3$) δ 7.34 – 7.28 (m, 2H), 7.24 – 7.19 (m, 3H), 4.51 (t, *J* = 10.6 Hz, 1H), 3.73 (s, 6H), 3.31 (dd, *J* = 10.6, 8.9 Hz, 2H), 2.12 (dtd, *J* = 11.2, 8.9, 1.0 Hz, 1H), 1.10 (dddd, *J* = 11.2, 7.9, 4.8, 3.1 Hz, 1H), 0.55 – 0.39 (m, 2H), 0.08 (dt, *J* = 5.9, 4.6 Hz, 2H).
^{13}C NMR (126 MHz, CDCl$_3$) δ 172.10, 141.40, 128.52, 126.76, 126.45, 51.65, 45.22, 44.10, 40.48, 8.59, 3.14.
HR-MS calculated for $C_{17}H_{21}O_4$ = 289.1434 [M+H]$^+$, found: 289.1437; calculated for $C_{17}H_{20}O_4Na$ = 311.1254 [M+H]$^+$, found: 311.1257.
IR ν$_{max}$ (cm^{-1}) 3001, 2943, 1632, 1442, 1375, 1039, 918.

Dimethyl (1*R*,2*s*,3*S*)-2-(4-bromophenyl)cyclobutane-1,3-dicarboxylate (Compound **24**)

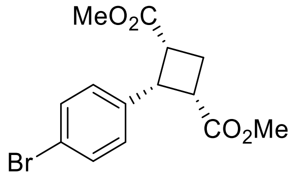

Compound 24 is prepared using procedure C.
Isolated yield: 50% yield (silica gel, 1:50 = acetone:petroleum ether)
^{1}H NMR (500 MHz, CDCl$_3$) δ 7.37 (d, *J* = 8.5 Hz, 2H), 7.06 (d, *J* = 8.5 Hz, 2H), 4.18 – 4.08 (m, 1H), 3.73 – 3.54 (m, 2H), 3.34 (s, 6H), 3.21 (tdd, *J* = 12.4, 11.3, 10.6, 1.0 Hz, 1H), 2.39 ppm (dtd, *J* = 12.7, 8.8, 8.8, 2.4 Hz, 1H).
^{13}C NMR (126 MHz, CDCl$_3$) δ 172.09, 137.10, 131.41, 129.75, 121.24, 53.58, 51.66, 47.29, 39.49, 24.51 ppm.
HR-MS calculated for $C_{14}H_{16}{}^{79}BrO_4$ = 327.0227 [M+H]$^+$, found: 327.0232; calculated for $C_{14}H_{16}{}^{81}BrO_4$ = 329.0206 [M+H]$^+$, found: 329.0210.
IR ν$_{max}$ (cm^{-1}) 1731, 1489, 1435, 1410, 1335, 1243, 1199, 1179, 1075, 1031, 1010, 836.

Methyl (1*S*,2*R*,3*R*)-2-(4-bromophenyl)-3-cyanocyclobutane-1-carboxylate (Compound **25**)

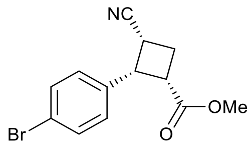

Compound 25 is prepared using procedure C.
Isolated yield: 44% (silica gel, 1:50 = acetone:petroleum ether)
^{1}H NMR (700 MHz, CDCl$_3$) δ 7.49 (d, *J* = 8.5 Hz, 2H), 7.22 (d, *J* = 8.5 Hz, 2H), 4.10 (t, *J* = 9.6 Hz, 1H), 3.66 (p, *J* = 9.7 Hz, 2H), 3.38 (s, 3H), 3.11 (dt, *J* = 12.6, 9.8 Hz, 1H), 2.69 (dtd, *J* = 11.1, 9.0, 2.1 Hz, 1H).
^{13}C NMR (176 MHz, CDCl$_3$) δ 171.01, 134.98, 131.77, 129.88, 122.25, 118.86, 51.83, 45.38, 41.09, 26.80, 24.91.
HR-MS calculated for $C_{13}H_{13}O_2N$ ^{79}Br = 294.0124, [M+H]$^+$, found: 294.0124; calculated for $C_{13}H_{13}O_2N$ ^{81}Br = 296.0104 [M+H]$^+$, found: 296.0103.
IR ν$_{max}$ (cm^{-1}) 1636, 1442, 1375, 1038, 918.

(Z)-3-(4-bromophenyl)acrylonitrile was isolated in 48% yield; *1H NMR and 13C NMR are consistent to the reported data.*[3]

Methyl (1*S*,2*R*,3*S*)-2-(4-bromophenyl)-3-cyanocyclobutane-1-carboxylate (Compound **26**)

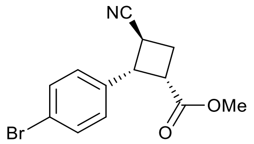

Compound 26 is prepared using procedure C.
Isolated yield: 48% (silica gel, 1:50 = acetone:petroleum ether)
^{1}H NMR (400 MHz, CD_2Cl_2) δ 7.49 (d, *J* = 8.2 Hz, 2H), 7.07 (d, *J* = 8.7 Hz, 2H), 4.20 (t, *J* = 10.0 Hz, 1H), 3.79 (q, *J* = 9.4 Hz, 1H), 3.62 – 3.46 (m, 1H), 3.37 (s, 3H), 2.69 (ddd, *J* = 11.9, 8.8, 2.5 Hz, 1H), 2.63 – 2.49 (m, 1H).
^{13}C NMR (101 MHz, CD_2Cl_2) δ 172.29, 136.08, 131.55, 128.33, 121.50, 120.54, 51.53, 45.88, 43.00, 25.49, 25.05.
HR-MS calculated for $C_{13}H_{13}O_2N$ ^{79}Br = 294.0124, $[M+H]^+$, found: 294.0124; calculated for $C_{13}H_{13}O_2N$ ^{81}Br = 296.0104 $[M+H]^+$, found: 296.0109.
IR ν_{max} (cm^{-1}) 2161, 1632, 1444, 1375, 1039, 918.

(*E*)-3-(4-bromophenyl)acrylonitrile was isolated in 23% yield*; 1H NMR and 13C NMR are consistent to the reported data.*[3]

rac-Trimethyl (2*R*,3*S*,4*R*)-2-(4-bromophenyl)-4-phenylcyclobutane-1,1,3-tricarboxylate (Compound **27**)

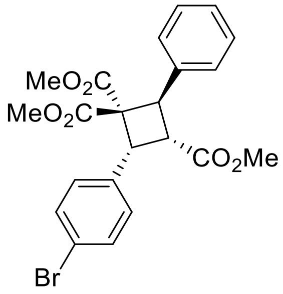

Compound 27 is prepared using procedure C.
Isolated yield: 40% (silica gel, 1:20 = acetone:petroleum ether to 1:10 = acetone:petroleum ether)
^{1}H NMR (500 MHz, $CDCl_3$) δ 7.42 (d, *J* = 8.5 Hz, 2H), 7.32 (d, *J* = 4.3 Hz, 4H), 7.25 – 7.23 (m, 1H), 7.16 (d, *J* = 8.5 Hz, 2H), 5.11 (d, *J* = 11.6, 1H), 4.52 (d, *J* = 10.5, 1H), 4.21 (dd, *J* = 11.6, 10.5 Hz, 1H), 3.40 (s, 3H), 3.32 (s, 3H), 3.24 ppm (s, 3H).
^{13}C NMR (126 MHz, $CDCl_3$) δ 171.27, 169.19, 136.74, 135.77, 131.66, 130.19, 128.43, 127.73, 127.48, 121.80, 62.37, 52.62, 52.45, 51.96, 46.55, 45.49, 41.63 ppm.
HR-MS calculated for $C_{22}H_{22}{}^{79}BrO_6$ = 461.0594 $[M+H]^+$, found: 461.0594; calculated for $C_{22}H_{22}{}^{81}Br\ O_6$ = 463.0574 $[M+H]^+$, found: 463.0573.
IR ν_{max} (cm^{-1}) 1728, 1490, 1434, 1248, 1211, 1173, 1143, 1106, 1075, 1047, 1010, 942, 838.

rac-(1*R*,2*R*,3*S*)-2-(4-bromophenyl)-1-methylcyclobutane-1,3-dicarboxylate (Compound **28**)

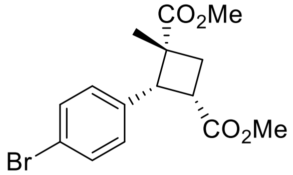

Compound 28 is prepared using procedure C.
Isolated yield: 76% yield (silica gel, 1:50 = acetone:petroleum ether to 1:20 = acetone:petroleum ether)

^{1}H NMR (500 MHz, CDCl$_3$) δ 7.38 (d, *J* = 8.5 Hz, 2H), 7.03 (d, *J* = 8.5 Hz, 2H), 3.68 (td, *J* = 10.3, 8.7 Hz, 1H), 3.60 (dd, *J* = 10.0, 2.8 Hz, 1H), 3.38 (dd, *J* = 12.6, 10.5 Hz, 1H), 3.35 (s, 3H), 3.30 (s, 3H), 2.07 (ddd, *J* = 12.6, 8.7, 2.8 Hz, 1H), 1.65 ppm (s, 3H).
^{13}C NMR (126 MHz, CDCl$_3$) δ 174.63, 172.56, 137.65, 131.20, 129.45, 120.90, 54.62, 51.56, 51.54, 45.97, 37.09, 30.71, 25.77 ppm.
HR-MS calculated for $C_{15}H_{18}{}^{79}BrO_4$ = 341.0383 [M+H]$^+$, found: 341.0392; calculated for $C_{15}H_{18}{}^{81}BrO_4$ = 343.0363 [M+H]$^+$, found: 343.0368.
IR ν$_{max}$ (cm^{-1}) 1730, 1489, 1434, 1358, 1305, 1291, 1246, 1196, 1160, 1138, 1074, 1043, 1010, 960, 835.

rac-Methyl (1*S*,2*S*,3*R*)-3-acetyl-1-methyl-2-phenylcyclobutane-1-carboxylate (Compound **29**)

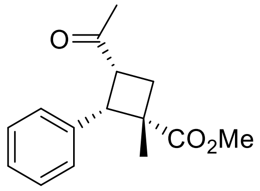

Compound 29 is prepared using procedure C.
Isolated yield: 48% yield (silica gel, 1:50 = acetone:petroleum ether to 1:20 = acetone:petroleum ether)
^{1}H NMR (500 MHz, CDCl$_3$) δ 7.37 – 7.32 (m, 3H), 7.28 – 7.21 (m, 2H), 4.10 (d, *J* = 10.3 Hz, 1H), 3.75 (s, 3H), 3.55 (q, *J* = 9.6 Hz, 1H), 2.66 (dd, *J* = 11.4, 9.6 Hz, 1H), 2.12 (s, 3H), 1.99 (ddd, *J* = 11.3, 8.9, 0.8 Hz, 1H), 1.05 ppm (s, 3H).
^{13}C NMR (126 MHz, CDCl$_3$) δ 208.40, 177.00, 137.74, 128.49, 127.87, 127.09, 52.29, 47.89, 43.73, 43.50, 31.38, 28.28, 19.16 ppm.
HR-MS calculated for $C_{15}H_{19}O_3$ = 247.1329 [M+H]$^+$, found: 247.1331.
IR ν$_{max}$ (cm^{-1}) 1726, 1709, 1604, 1498, 1452, 1356, 1312, 1224, 1202, 1161, 1105, 1033, 992, 881

Dimethyl (2*R*,3*r*,4*S*)-3-(4-bromophenyl)spiro[cyclobutane-1,9'-fluorene]-2,4-dicarboxylate (Compound **30**)

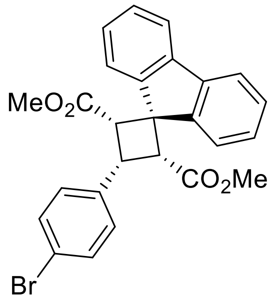

Compound 30 is prepared using procedure C.
Isolated yield: 62% yield (silica gel, 1:20 = acetone:petroleum ether)
^{1}H NMR (500 MHz, CDCl$_3$) δ 8.04 (dt, *J* = 7.8, 0.9 Hz, 1H), 7.82 – 7.73 (m, 2H), 7.70 – 7.63 (m, 1H), 7.48 – 7.37 (m, 2H), 7.33 (td, *J* = 7.5, 1.1 Hz, 1H), 7.15 (td, *J* = 7.6, 1.2 Hz, 1H), 7.12 – 7.08 (m, 2H), 6.34 – 6.29 (m, 2H), 4.72 (d, *J* = 10.0 Hz, 1H), 4.28 (t, *J* = 10.2 Hz, 1H), 4.20 (d, *J* = 10.4 Hz, 1H), 3.83 (s, 3H), 3.18 ppm (s, 3H).
^{13}C NMR (126 MHz, CDCl$_3$) δ 172.13, 169.34, 150.53, 142.80, 140.87, 139.99, 135.99, 130.72, 128.78, 128.49, 128.32, 127.88, 127.40, 122.12, 120.03, 119.92, 119.61, 58.14, 53.59, 52.17, 51.51, 47.01, 46.12, 41.03 ppm.
HR-MS calculated for $C_{26}H_{21}{}^{79}BrO_4$ = 477.0696 [M+H]$^+$, found: 477.0696; calculated for $C_{26}H_{21}{}^{81}BrO_4$ = 479.0676 [M+H]$^+$, found: 479.0675.
IR ν$_{max}$ (cm^{-1}) 1736, 1688, 1586, 1489, 1450, 1437, 1398, 1377, 1197, 1171, 1100, 1072, 1011, 838.

rac-Methyl (1*R*,2*R*,2'*R*,3'*S*,4'*R*)-2'-(4-bromophenyl)-3-oxo-4'-phenylspiro[bicyclo[2.2.1]heptane-2,1'-cyclobutane]-3'-carboxylate (Compound **31**)

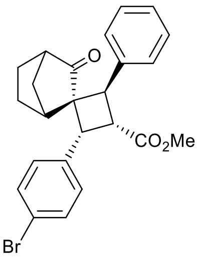

Compound 31 is prepared using procedure C.
Isolated yield: 65% (silica gel, 1:20 = acetone:petroleum ether to 1:10 = acetone:petroleum ether)
^{1}H NMR (500 MHz, CDCl$_3$) δ 7.35 – 7.30 (m, 2H), 7.28 – 7.22 (m, 4H), 7.19 (s, 1H), 7.16 – 7.10 (m, 2H), 4.65 (d, *J* = 11.3 Hz, 1H), 3.95 (t, *J* = 11.2 Hz, 1H), 3.71 (d, *J* = 11.0 Hz, 1H), 3.32 (s, 3H), 2.73 (dd, *J* = 4.5, 2.0 Hz, 1H), 2.35 – 2.25 (m, 1H), 1.70 (dt, *J* = 10.5, 1.8 Hz, 1H), 1.58 – 1.46 (m, 1H), 1.38 (ddd, *J* = 10.5, 2.1, 1.0 Hz, 1H), 1.12 (dddd, *J* = 13.0, 9.0, 4.7, 2.0 Hz, 1H), 1.03 (ddt, *J* = 13.3, 12.4, 4.6 Hz, 1H), 0.39 ppm (dddd, *J* = 13.6, 9.1, 4.9, 2.2 Hz, 1H).
^{13}C NMR (126 MHz, CDCl$_3$) δ 214.85, 171.60, 138.37, 136.10, 131.24, 130.63, 128.79, 127.96, 127.38, 121.40, 62.12, 53.22, 51.68, 49.67, 46.43, 42.86, 41.72, 34.28, 27.39, 21.59 ppm.
HR-MS calculated for $C_{24}H_{24}{}^{79}BrO_3$ = 439.0903 [M+H]$^+$, found: 439.0907; calculated for $C_{24}H_{24}{}^{81}BrO_3$ = 441.0883 [M+H]$^+$, found: 441.0885;
IR ν_{max} (cm^{-1}) 1733, 1489, 1435, 1198, 1179, 1076, 1010, 834.

rac-Methyl (1*R*,2*R*,3*S*,4*R*)-2-(4-bromophenyl)-1'-oxo-4-phenyl-1',3'-dihydrospiro[cyclobutane-1,2'-indene]-3-carboxylate (Compound **32**)

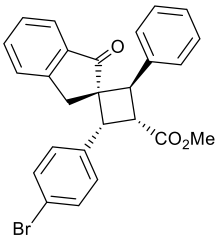

Compound 32 is prepared using procedure C.
Isolated yield: 47% yield (silica gel, 1:20 = acetone:petroleum ether to 1:10 = acetone:petroleum ether)
^{1}H NMR (500 MHz, CDCl$_3$) δ 7.58 – 7.51 (m, 2H), 7.42 – 7.35 (m, 3H), 7.35 – 7.28 (m, 3H), 7.27 – 7.22 (m, 1H), 7.17 – 7.12 (m, 2H), 7.12 – 7.08 (m, 2H), 5.13 (d, *J* = 11.8 Hz, 1H), 4.16 (dd, *J* = 11.9, 10.1 Hz, 1H), 3.87 (d, *J* = 10.2 Hz, 1H), 3.54 (s, 3H), 3.27 – 3.00 ppm (m, 2H).
^{13}C NMR (126 MHz, CDCl$_3$) δ 202.55, 171.85, 151.05, 138.66, 136.00, 135.18, 134.76, 131.10, 130.63, 128.74, 127.78, 127.03, 126.96, 126.33, 124.07, 121.48, 57.73, 55.66, 51.86, 43.05, 41.63, 38.21 ppm.
HR-MS calculated for $C_{26}H_{22}{}^{79}BrO_3$ = 461.0747 [M+H]$^+$, found: 461.0749; calculated for $C_{26}H_{22}{}^{81}BrO_3$ = 463.0726 [M+H]$^+$, found: 463.0726.
IR ν_{max} (cm^{-1}) 1730, 1702, 1603, 1490, 1465, 1435, 1412, 1325, 1282, 1207, 1152, 1124, 1075, 1009, 927, 870, 834.

rac-Methyl (2'*R*,3*R*,3'*S*,4'*R*)-2'-(4-bromophenyl)-4-oxo-4'-phenylspiro[chromane-3,1'-cyclobutane]-3'-carboxylate (Compound **33**)

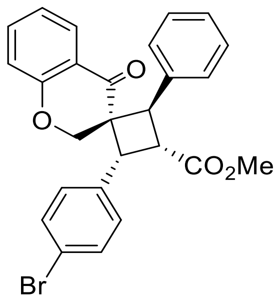

Compound 33 is prepared using procedure C.
Isolated yield: 42% yield (silica gel, 1:20 = acetone:petroleum ether to 1:10 = acetone:petroleum ether)
^{1}H NMR (500 MHz, CDCl$_3$) δ 7.46 (dd, *J* = 7.9, 1.7 Hz, 1H), 7.37 (ddd, *J* = 8.8, 7.2, 1.8 Hz, 1H), 7.32 (ddd, *J* = 7.6, 6.2, 1.3 Hz, 2H), 7.25 – 7.18 (m, 5H), 6.97 – 6.91 (m, 2H), 6.89 (dd, *J* = 8.5, 1.0 Hz, 1H), 6.84 (ddd, *J* = 8.1, 7.2, 1.1 Hz, 1H), 5.45 (d, *J* = 11.3 Hz, 1H), 4.29 (d, *J* = 12.2 Hz, 1H), 4.24 (d, *J* = 10.5 Hz, 1H), 4.21 – 4.13 (m, 1H), 3.99 (d, *J* = 12.1 Hz, 1H), 3.48 ppm (s, 3H).
^{13}C NMR (126 MHz, CDCl$_3$) δ ^{13}C NMR (126 MHz, CDCl3) δ 190.73, 171.71, 160.44, 135.92, 135.85, 134.88, 131.14, 130.17, 128.66, 127.37, 127.23, 126.98, 121.51, 121.31, 120.47, 117.19, 71.88, 52.19, 51.93, 46.89, 40.36, 38.25 ppm.
HR-MS calculated for $C_{26}H_{22}{}^{79}BrO_4$ = 477.0696 $[M+H]^+$, found: 477.0695; calculated for $C_{26}H_{22}{}^{79}BrO_4$ = 479.0676 $[M+H]^+$, found: 479.0674.
IR ν$_{max}$ (cm^{-1}) 1733, 1687, 1605, 1490, 1475, 1465, 1315, 1208, 1143, 1034, 1010.

Methyl (1*S*,2*R*,4*R*,9S)-1-(4-bromophenyl)-6-methyl-5-oxo-9-(prop-1-en-2-yl)spiro[3.5]non-6-ene-2-carboxylate (Compound **34**)

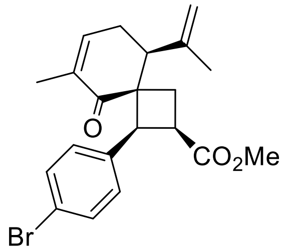

Compound 34 is prepared using procedure C.
Isolated yield: 44% (silica gel, 1:20 = acetone:petroleum ether to 1:10 = acetone:petroleum ether)
^{1}H NMR (500 MHz, CDCl$_3$) δ 7.29 (d, *J* = 8.5 Hz, 2H), 6.88 (d, *J* = 8.5 Hz, 2H), 6.19 (ddt, *J* = 4.1, 2.7, 1.4 Hz, 1H), 4.86 – 4.74 (m, 1H), 4.62 – 4.47 (m, 1H), 3.93 (dd, *J* = 9.5, 2.3 Hz, 1H), 3.69 – 3.57 (m, 2H), 3.40 (s, 3H), 3.21 – 3.08 (m, 1H), 3.01 – 2.90 (m, 1H), 2.35 (ddt, *J* = 19.8, 5.6, 1.5 Hz, 1H), 2.02 – 1.94 (m, 1H), 1.66 – 1.52 (m, 3H), 1.36 – 1.13 ppm (m, 3H).
^{13}C NMR (126 MHz, CDCl$_3$) δ 198.10, 172.90, 143.68, 139.09, 136.07, 135.50, 130.87, 130.48, 120.95, 114.56, 52.83, 51.79, 51.75, 51.57, 36.88, 27.05, 23.74, 22.00, 15.70 ppm.
HR-MS calculated for $C_{21}H_{24}{}^{79}BrO_3$ = 403.0903 $[M+H]^+$, found: 403.0908; calculated for $C_{21}H_{24}{}^{81}BrO_3$ = 405.0883 $[M+H]^+$, found: 405.0883.
$[\alpha]_D^{20}\ -99\ (c\ 0.10, CHCl_3)$
IR ν$_{max}$ (cm^{-1}) 1730, 1663, 1489, 1435, 1353, 1201, 1156, 1076, 1010, 900, 847, 825.

Methyl (1*R*,2*R*,3*S*,3a'*R*,5a'*S*,9a'*S*,9b'*R*)-2-(4-bromophenyl)-3a',6',6',9a'-tetramethyl-2'-oxodecahydro-2'*H*-spiro[cyclobutane-1,1'-naphtho[2,1-*b*]furan]-3-carboxylate (Compound **35**)

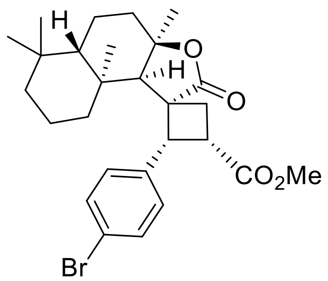

Compound 35 is prepared using procedure C.

Isolated yield: 40% (silica gel, 1:20 = acetone:petroleum ether to 1:10 = acetone:petroleum ether)

^{1}H NMR (500 MHz, CDCl$_3$) δ 7.32 (d, *J* = 8.5 Hz, 2H), 7.07 (d, *J* = 8.4 Hz, 2H), 4.36 (d, *J* = 11.3 Hz, 1H), 3.56 (s, 3H), 3.47 (ddd, *J* = 11.3, 8.9, 5.1 Hz, 1H), 2.94 – 2.73 (m, 2H), 2.05 – 1.98 (m, 1H), 1.94 (dt, *J* = 12.0, 3.4 Hz, 1H), 1.80 (m, 2H), 1.73 – 1.62 (m, 1H), 1.60 – 1.46 (m, 3H), 1.42 – 1.33 (m, 2H), 1.31 (s, 3H), 1.22 – 1.15 (m, 1H), 1.12 (s, 3H), 0.95 (dd, *J* = 12.5, 2.7 Hz, 1H), 0.80 (s, 3H), 0.78 ppm (s, 3H).

^{13}C NMR (126 MHz, CDCl$_3$) δ 178.70, 172.14, 134.94, 131.17, 130.78, 121.07, 84.57, 61.39, 57.40, 53.72, 51.70, 48.72, 41.65, 40.30, 40.20, 39.27, 38.71, 33.52, 33.29, 28.73, 25.25, 21.04, 20.39, 18.13, 17.41 ppm.

HR-MS calculated for $C_{27}H_{36}{}^{79}BrO_4$ = 503.1792 $[M+H]^+$, found: 503.1793; calculated for $C_{27}H_{36}{}^{81}BrO_4$ = 505.1771 $[M+H]^+$, found: 505.1771;

$[\alpha]_D^{20}$ -167 (c 0.10, $CHCl_3$)

IR ν$_{max}$ (cm^{-1}) 1762, 1733, 1489, 1459, 1390, 1352, 1284, 1257, 1222, 1172, 1148, 1131, 1119, 1064, 1039, 1010, 971, 941, 914, 893, 878, 859, 830.

rac-Dimethyl (1*S*,2*R*,3*S*,4*S*,6*S*,7*S*,8*R*,9*S*)-1,7-bis(4-bromophenyl)-5-oxo-3,9-diphenyldispiro[3.1.3^{6}.1^{4}]decane-2,8-dicarboxylate (Compound **36**)

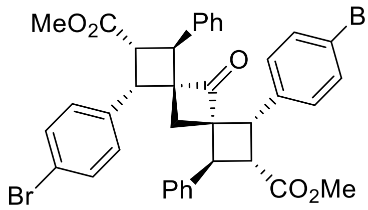

Compound 36 is prepared using procedure D.

Isolated yield: 36% (silica gel, 1:20 = acetone:petroleum ether to 1:10 = acetone:petroleum ether)

^{1}H NMR (500 MHz, CDCl$_3$) δ 7.52 – 7.42 (m, 4H), 7.34 – 7.28 (m, 6H), 7.20 – 7.16 (m, 4H), 6.87 – 6.84 (m, 4H), 4.60 (d, *J* = 10.7 Hz, 2H), 3.64 (t, *J* = 10.7 Hz, 2H), 3.47 (d, *J* = 10.7 Hz, 2H), 3.31 (s, 6H), 1.94 ppm (s, 2H).

^{13}C NMR (126 MHz, CDCl$_3$) δ 208.79, 170.92, 137.70, 135.10, 131.20, 130.64, 128.85, 127.49, 121.53, 64.74, 51.79, 51.69, 45.94, 41.89, 33.96 ppm.

HR-MS calculated for $C_{38}H_{33}{}^{79}Br^{79}BrO_5$ = 727.0689 $[M+H]^+$, found: 727.0693; calculated for $C_{38}H_{33}{}^{79}Br^{81}BrO_5$ = 729.06689 $[M+H]^+$, found: 729.0671; calculated for $C_{38}H_{33}{}^{81}Br^{81}BrO_5$ = 731.0648 $[M+H]^+$, found: 731.0643.

IR ν$_{max}$ (cm^{-1}) 1757, 1731, 1489, 1436, 1410, 1274, 1209, 1174, 1132, 1075, 1010, 963, 919, 853.

rac-Dimethyl (1*R*,2*S*,3*R*,4*R*,6*R*,7*R*,8*S*,9*R*)-1,7-bis(4-bromophenyl)-5-oxo-3,9-diphenyldispiro[3.1.3^{6}.2^{4}]undecane-2,8-dicarboxylate (Compound **37**)

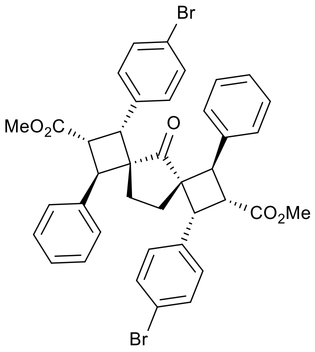

Compound 37 is prepared using procedure D.
Isolated yield: 59% (silica gel, 1:20 = acetone:petroleum ether to 1:10 = acetone:petroleum ether)
^{1}H NMR (500 MHz, $CDCl_3$) δ 7.56 – 7.39 (m, 4H), 7.23 – 7.17 (m, 6H), 6.98 (d, *J* = 8.5 Hz, 4H), 6.54 (dd, *J* = 7.2, 2.3 Hz, 4H), 4.54 (d, *J* = 11.6 Hz, 2H), 3.92 (dd, *J* = 11.1, 11.1 Hz, 2H), 3.48 (d, *J* = 10.6 Hz, 2H), 3.40 (s, 6H), 1.87 (d, *J* = 8.1 Hz, 2H), 1.57 ppm (d, *J* = 8.2 Hz, 2H).
^{13}C NMR (126 MHz, $CDCl_3$) δ 211.22, 171.39, 137.20, 135.16, 131.24, 130.90, 128.44, 127.26, 126.95, 121.44, 57.46, 55.43, 51.72, 42.74, 41.38, 30.76 ppm.
HR-MS calculated for $C_{39}H_{35}{}^{79}Br^{79}BrO_5$ = 741.0846 $[M+H]^+$,found:741.0849;calculated for $C_{39}H_{35}{}^{79}Br^{81}Br\ O_5$ = 743.0825 $[M+H]^+$, found: 743.0831; calculated for $C_{39}H_{35}{}^{81}Br^{81}Br\ O_5$ = 745.0805 $[M+H]^+$, found: 745.0804.
IR ν_{max} (cm^{-1}) 1730, 1489, 1435, 1412, 1312, 1203, 1160, 1075, 1010, 843.

Diethyl (1*R*,2*S*)-cyclobutane-1,2-dicarboxylate (Compound ***cis*-46**)

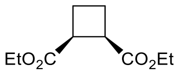

Compound *cis*-46 is prepared using procedure C.
Isolated yield: 39% (> 20:1 d.r.) (silica gel, 1:20 = acetone:petroleum ether)
^{1}H NMR (700 MHz, $CDCl_3$) δ 4.13 (qd, *J* = 7.1, 0.8 Hz, 4H), 3.47 – 3.28 (m, 2H), 2.46 – 2.32 (m, 2H), 2.26 – 2.12 (m, 2H), 1.25 (t, *J* = 7.1 Hz, 6H).
^{13}C NMR (176 MHz, $CDCl_3$) δ 173.28, 60.52, 40.64, 22.08, 14.18.
HR-MS calculated for $C_{10}H_{17}O_4$ = 201.1121 $[M+H]^+$, found: 201.1121.
IR ν_{max} (cm^{-1}) 1633, 1442, 1375, 1039, 918.

Starting material ***cis*-45** is prepared using the reported method.[4]

Stereoselective synthesis of cyclobutanes from pyrrolidines

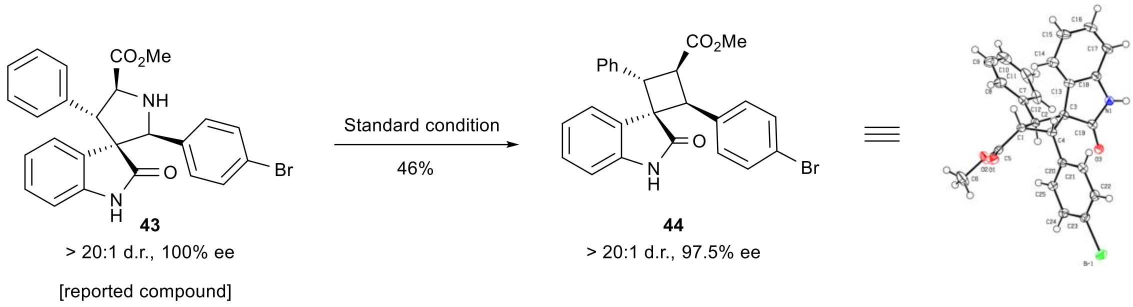

Scheme S1. Retention of stereochemistry in ring contraction. Enantioenriched pyrrolidine was prepared according to ref. 1.

Methyl (1*R*,2*R*,3*S*,4*R*)-2-(4-bromophenyl)-2'-oxo-4-phenylspiro[cyclobutane-1,3'-indoline]-3-carboxylate (Compound **44**)

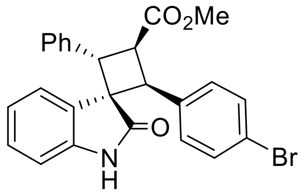

Compound 44 is prepared using procedure C.
Isolated yield: 46%, 97.7% *ee* (silica gel, 1:20 = acetone:petroleum ether to 1:10 = acetone:petroleum ether)
^{1}H NMR (700 MHz, CDCl$_3$) δ 7.42 (d, *J* = 8.5 Hz, 2H), 7.27 (s, 2H), 7.16 (t, *J* = 7.6 Hz, 2H), 7.13 – 7.08 (m, 2H), 7.02 (d, *J* = 7.5 Hz, 1H), 6.90 (d, *J* = 8.1 Hz, 2H), 6.87 (t, *J* = 7.6 Hz, 1H), 6.75 (d, *J* = 7.7 Hz, 1H), 5jj 10.8 Hz, 1H), 3.57 ppm (s, 3H).
^{13}C NMR (126 MHz, CDCl$_3$) δ 178.70, 172.14, 134.94, 131.17, 130.78, 121.07, 84.57, 61.39, 57.40, 53.72, 51.70, 48.72, 41.65, 40.30, 40.20, 39.27, 38.71, 33.52, 33.29, 28.73, 25.25, 21.04, 20.39, 18.13, 17.41 ppm.
HR-MS calculated for $C_{25}H_{21}{}^{79}BrNO_3$ = 462.0699 [M+H]$^+$, found: 462.0702; calculated for $C_{25}H_{21}{}^{81}BrNO_3$ = 464.0679 [M+H]$^+$, found: 464.0680;
IR ν_{max} (cm^{-1}) 1706, 1617, 1489, 1470, 1329, 1262, 1210, 1109, 1074, 1010, 830.
$[\alpha]_D^{20}$ -62 (c 0.10, $CHCl_3$)
HPLC conditions: CHIRALPAK IA column, *iso*-propanol/ *iso*-hexane = 10/90, flow rate = 0.5 mL min, minor enantiomer: t_R = 30.81 min; major enantiomer: t_R = 49.61 min; (97.6% *ee*);

HPLC traces (compound **43**)

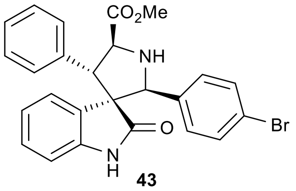

43

> 20:1 d.r., 100% ee

racemic

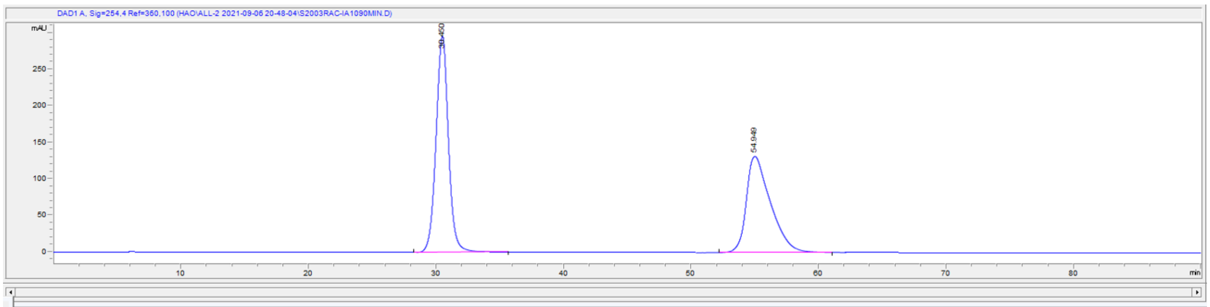

#	Time	Area	Height	Width	Area%	Symmetry
1	30.45	20249.4	297.6	1.0359	53.821	0.973
2	54.949	17374.1	132.1	1.832	46.179	0.524

#	Time	Area	Height	Width	Area%	Symmetry
1	30.45	20249.4	297.6	1.0359	53.821	0.973
2	54.949	17374.1	132.1	1.832	46.179	0.524

chiral

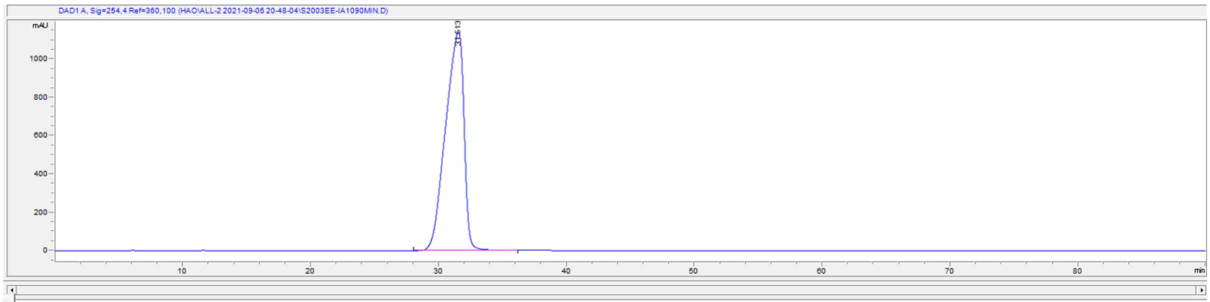

#	Time	Area	Height	Width	Area%	Symmetry
1	31.513	114088.5	1139.8	1.4938	100.000	2.138

#	Time	Area	Height	Width	Area%	Symmetry
1	31.513	114088.5	1139.8	1.4938	100.000	2.138

Method IA 10C: CHIRALPAK IA column, *iso*-propanol/ *iso*-hexane = 10/90, flow rate = 0.5 mL min

HPLC traces (compound **44**)

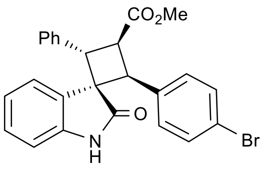

44

> 20:1 d.r., 97.5% ee

racemic

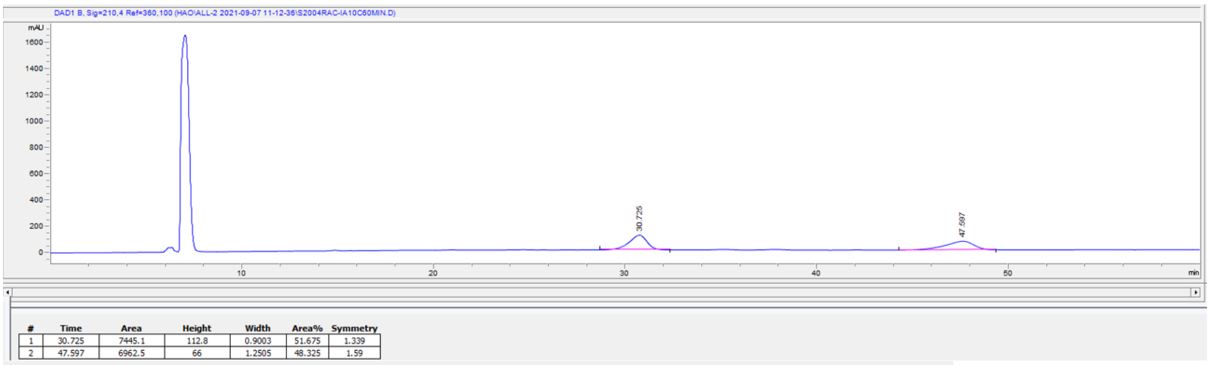

#	Time	Area	Height	Width	Area%	Symmetry
1	30.725	7445.1	112.8	0.9003	51.675	1.339
2	47.597	6962.5	66	1.2505	48.325	1.59

chiral

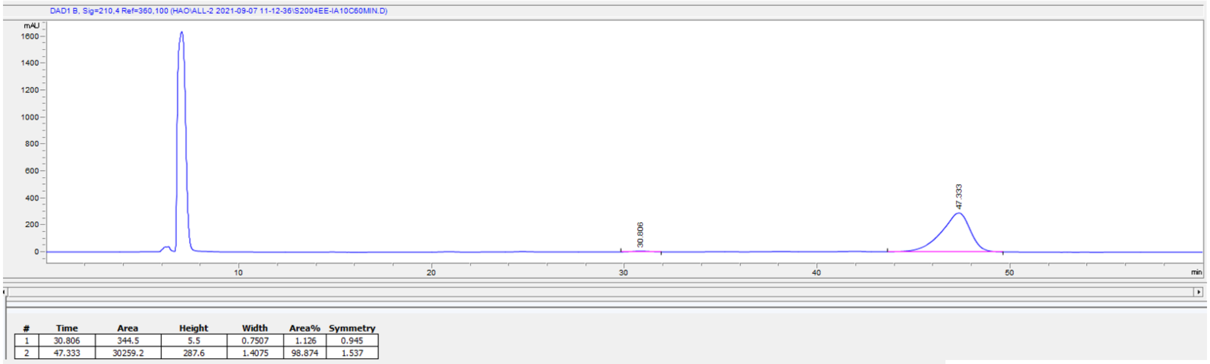

#	Time	Area	Height	Width	Area%	Symmetry
1	30.806	344.5	5.5	0.7507	1.126	0.945
2	47.333	30259.2	287.6	1.4075	98.874	1.537

Method IA 10C: CHIRALPAK IA column, *iso*-propanol/ *iso*-hexane = 10/90, flow rate = 0.5 mL min

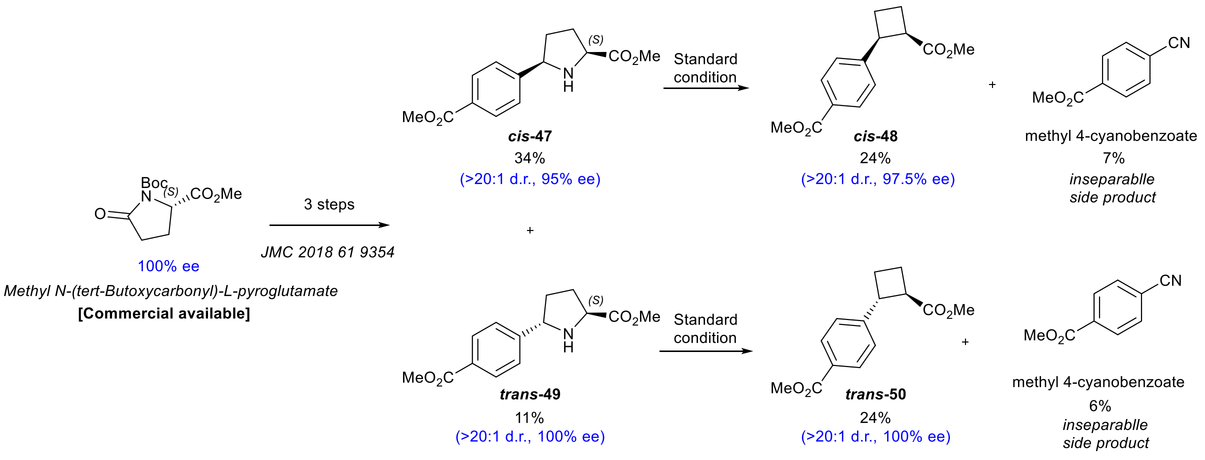

Scheme S2. Investigation of stereospecificity of cyclobutane synthesis from pyroglutamate derived pyrrolidine.

Methyl (2*S*,5*R*)-5-(4-(methoxycarbonyl)phenyl)pyrrolidine-2-carboxylate (Compound ***cis*-47**)

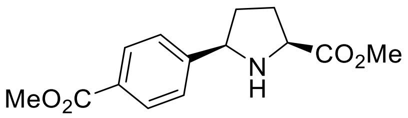

Compound *cis*-47 is prepared using the reported procedure.[5]
Isolated yield: 34%, (>20:1 d.r. 95% *ee*)
^{1}H NMR (400 MHz, $CDCl_3$) δ 8.00 (d, *J* = 8.0 Hz, 2H), 7.52 (d, *J* = 8.1 Hz, 2H), 4.32 (dd, *J* = 9.2, 5.8 Hz, 1H), 4.10 – 3.97 (m, 1H), 3.91 (s, 3H), 3.78 (s, 3H), 2.30 – 2.10 (m, 3H), 1.82 – 1.67 (m, 1H).
^{13}C NMR (126 MHz, $CDCl_3$) δ 175.01, 166.95, 147.95, 129.92, 129.28, 126.84, 63.15, 59.87, 52.46, 52.13, 34.04, 30.25.
HR-MS calculated for $C_{14}H_{18}O_4N$ = 264.1230 $[M+H]^+$, found: 264.1231.
IR ν_{max} (cm^{-1}) 2952, 2160, 2028, 1720, 1610, 1435, 1278, 1205, 1112, 1018.
$[\alpha]_D^{20}$ + 34 (c 0.10, $CHCl_3$)
HPLC conditions: CHIRALPAK IA column, *iso*-propanol/ *iso*-hexane = 10/90, flow rate = 0.5 mL min, minor enantiomer: t_R = 32.43 min; major enantiomer: t_R = 25.94 min; (95.3% *ee*);

Methyl (2*S*,5*S*)-5-(4-(methoxycarbonyl)phenyl)pyrrolidine-2-carboxylate (Compound ***trans*-49**)

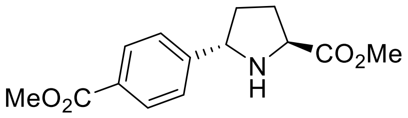

Compound *trans*-49 is prepared using the reported procedure.[5]
Isolated yield: 11% (>20:1 d.r. 100% *ee*)
^{1}H NMR (400 MHz, CD_2Cl_2) δ 7.97 (d, *J* = 8.1 Hz, 2H), 7.48 (d, *J* = 8.7 Hz, 2H), 4.49 – 4.33 (m, 1H), 4.03 (dd, *J* = 8.2, 5.8 Hz, 1H), 3.88 (s, 3H), 3.75 (s, 3H), 2.45 – 2.15 (m, 2H), 2.07 – 1.91 (m, 1H), 1.67 (dtd, *J* = 12.2, 8.2, 7.6 Hz, 1H).
^{13}C NMR (101 MHz, CD_2Cl_2) δ 176.14, 166.81, 150.59, 129.36, 128.69, 126.38, 61.31, 59.63, 51.91, 51.76, 34.85, 29.77.
HR-MS calculated for $C_{14}H_{18}O_4N$ = 264.1230 $[M+H]^+$, found: 264.1232.
IR ν_{max} (cm^{-1}) 2951, 2158, 2015, 1721, 1610, 1436, 1278, 1214, 1112, 1018.
$[\alpha]_D^{20}$ − 23 (c 0.10, $CHCl_3$)
HPLC conditions: CHIRALPAK IA column, *iso*-propanol/ *iso*-hexane = 10/90, flow rate = 0.5 mL min, enantiomer: t_R = 24.02 min; (100% *ee*);

Methyl 4-((1*S*,2*R*)-2-(methoxycarbonyl)cyclobutyl)benzoate (Compound ***cis*-48**)

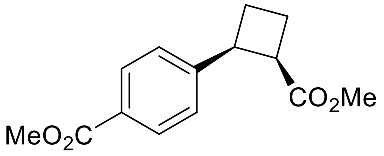

Compound *cis*-48 is prepared using procedure C.
Isolated yield: 24% (> 20:1 d.r., 97.5% ee) + 7% Methyl 4-cyanobenzoate as an inseparable mixture
^{1}H NMR (500 MHz, CDCl$_3$) δ 7.96 (d, *J* = 8.4 Hz, 2H), 7.26 (d, *J* = 8.1 Hz, 2H), 4.01 (q, *J* = 8.8 Hz, 1H), 3.90 (s, 3H), 3.61 – 3.53 (m, 1H), 3.26 (s, 3H), 2.71 – 2.59 (m, 1H), 2.45 – 2.30 (m, 2H), 2.30 – 2.20 (m, 1H).
^{13}C NMR (126 MHz, CDCl$_3$) δ 173.60, 167.10, 146.47, 129.43, 128.31, 127.20, 52.06, 51.17, 44.94, 42.40, 24.38, 20.37.
HR-MS calculated for $C_{14}H_{17}O_4$ = 249.1121 $[M+H]^+$, found: 249.1124; calculated for $C_{14}H_{16}O_4Na$ = 271.0941 $[M+H]^+$, found: 271.0939.
IR ν_{max} (cm^{-1}) 1723, 1610, 1436, 1279, 1180, 1107.
$[\alpha]_D^{20} + 15\ (c\ 0.10, CHCl_3)$
HPLC conditions: CHIRALPAK IC column, *iso*-propanol/ *iso*-hexane = 3/97, flow rate = 0.5 mL min, minor enantiomer: t_R = 62.74 min; major enantiomer: t_R = 68.79 min; (97.4% *ee*);
The stereochemistry of cis-48 is in analogy to the previously reported compound.[6]

Methyl 4-((1*R*,2*R*)-2-(methoxycarbonyl)cyclobutyl)benzoate (Compound ***trans*-50**)

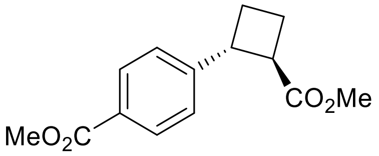

Compound *trans*-50 is prepared using procedure C.
Isolated yield: 24% (> 20:1 d.r., 100% ee) + 6% Methyl 4-cyanobenzoate as an inseparable mixture
^{1}H NMR (500 MHz, CDCl$_3$) δ 7.98 (d, *J* = 8.2 Hz, 2H), 7.30 (d, *J* = 8.2 Hz, 2H), 3.90 (s, 3H), 3.84 (q, *J* = 9.5 Hz, 1H), 3.71 (s, 3H), 3.27 – 3.16 (m, 1H), 2.41 – 2.26 (m, 2H), 2.23 – 2.11 (m, 2H).
^{13}C NMR (126 MHz, CDCl$_3$) δ 174.60, 167.05, 148.83, 129.76, 128.25, 126.41, 52.08, 51.86, 44.94, 42.96, 25.21, 21.79.
HR-MS calculated for $C_{14}H_{17}O_4$ = 249.1121 $[M+H]^+$, found: 249.1121.
IR ν_{max} (cm^{-1}) 1723, 1610, 1435, 1278,1179, 1108.
$[\alpha]_D^{20}\ - 30\ (c\ 0.10, CHCl_3)$
HPLC conditions: CHIRALPAK IA column, *iso*-propanol/ *iso*-hexane = 1/99, flow rate = 0.5 mL min, enantiomer: t_R = 32.16 min; (100% *ee*);
The stereochemistry of cis-48 is in analogy to the previously reported compound.[6,7]

HPLC trace (compound ***cis*-47**)

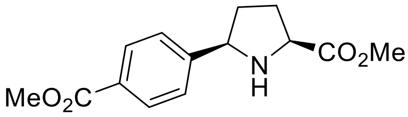

95% ee

racemic

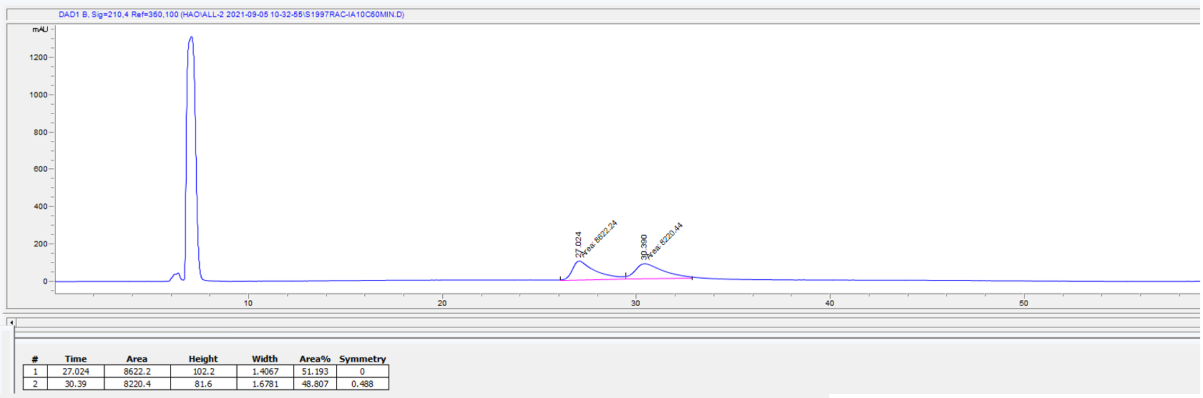

#	Time	Area	Height	Width	Area%	Symmetry
1	27.024	8622.2	102.2	1.4067	51.193	0
2	30.39	8220.4	81.6	1.6781	48.807	0.488

chiral

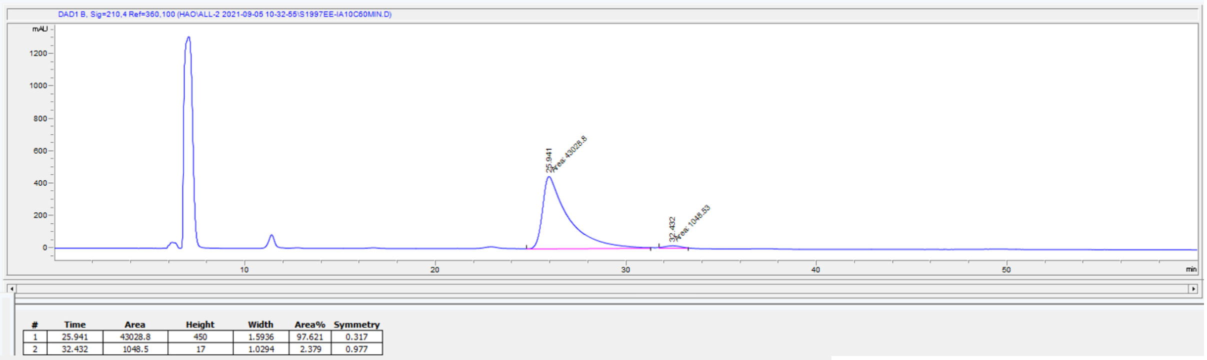

#	Time	Area	Height	Width	Area%	Symmetry
1	25.941	43028.8	450	1.5936	97.621	0.317
2	32.432	1048.5	17	1.0294	2.379	0.977

Condition: DAD1 B, Sig=254.4, Ref=360; Column IA, 10% iPrOH in *iso*-hexane, 60min

HPLC trace (compound ***cis*-49**)

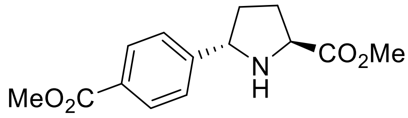

100% ee

racemic

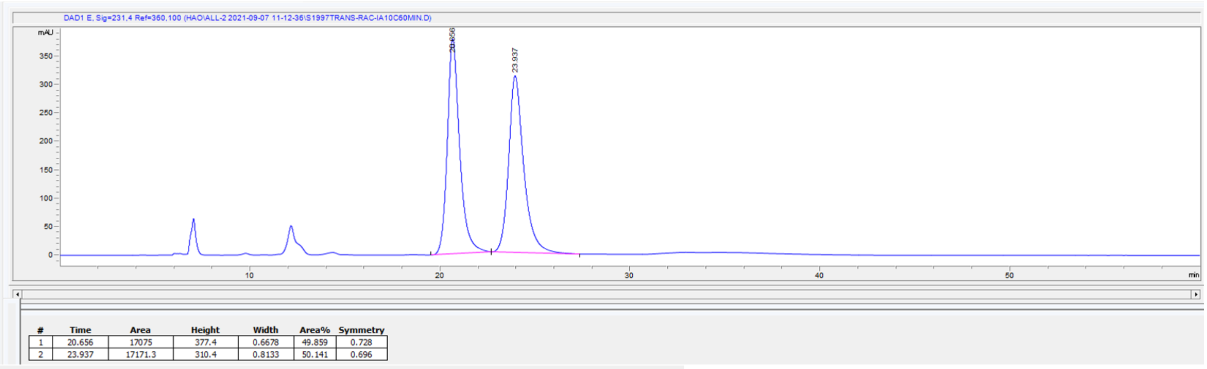

#	Time	Area	Height	Width	Area%	Symmetry
1	20.656	17075	377.4	0.6678	49.859	0.728
2	23.937	17171.3	310.4	0.8133	50.141	0.696

chiral

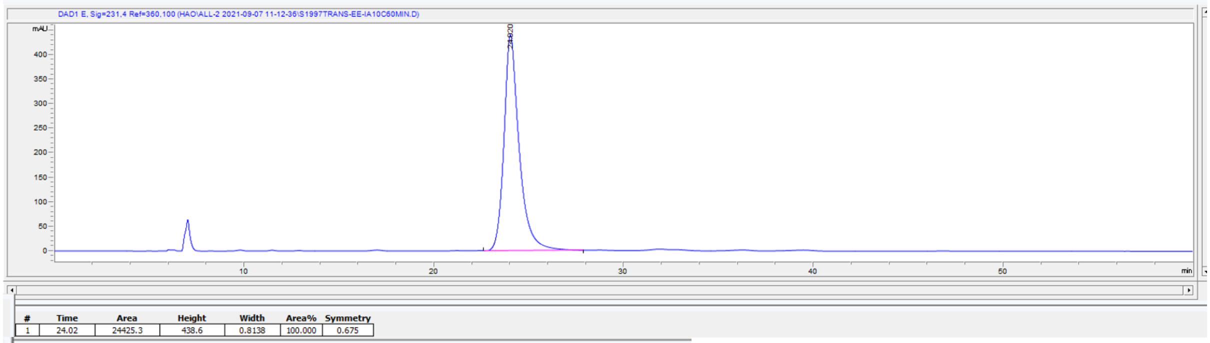

#	Time	Area	Height	Width	Area%	Symmetry
1	24.02	24425.3	438.6	0.8138	100.000	0.675

Condition: DAD1 E, Sig=231.4, Ref=360; Column IA, 10% iPrOH in *iso*-hexane, 60min

HPLC trace (compound ***trans*-48**)

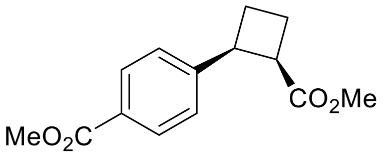

97.5% ee

racemic

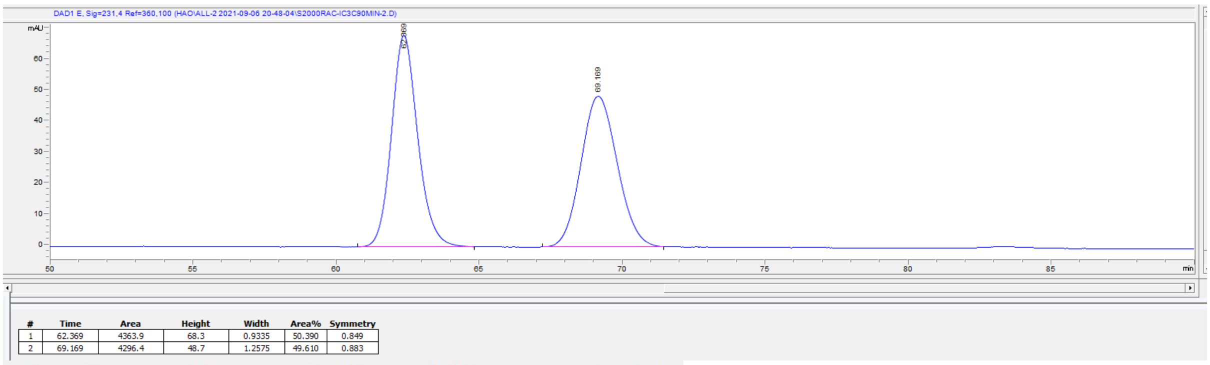

#	Time	Area	Height	Width	Area%	Symmetry
1	62.369	4363.9	68.3	0.9335	50.390	0.849
2	69.169	4296.4	48.7	1.2575	49.610	0.883

chiral

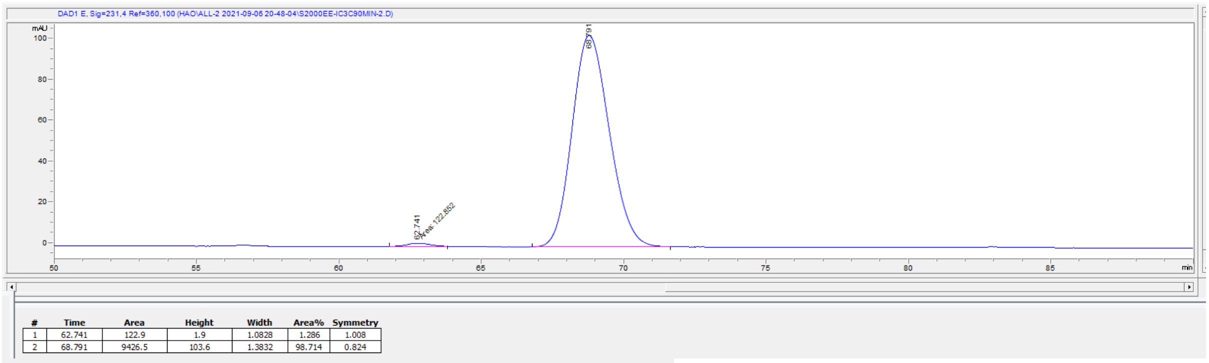

#	Time	Area	Height	Width	Area%	Symmetry
1	62.741	122.9	1.9	1.0828	1.286	1.008
2	68.791	9426.5	103.6	1.3832	98.714	0.824

Condition: DAD1 E, Sig=231.4, Ref=360; Column IA, 10% iPrOH in *iso*-hexane, 60min

HPLC trace (compound ***trans*-50**)

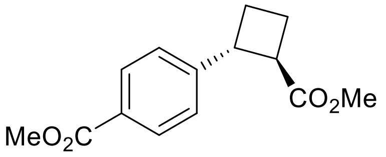

racemic

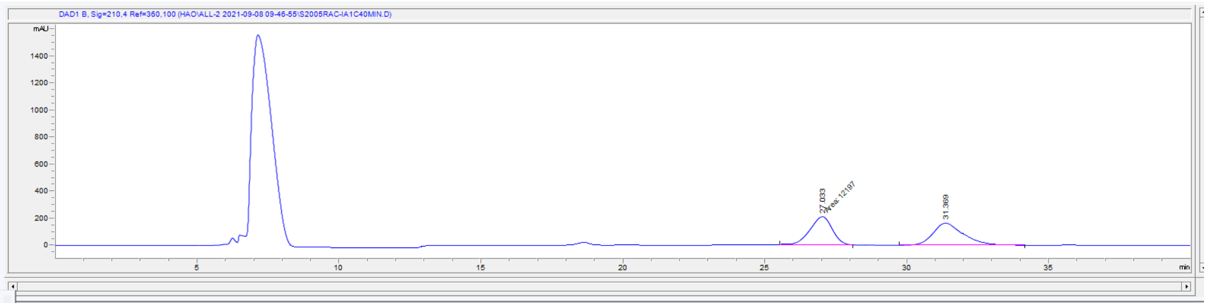

#	Time	Area	Height	Width	Area%	Symmetry
1	27.033	12197	213.2	0.9534	50.519	1.236
2	31.369	11946.4	163.1	1.0388	49.481	0.683

#	Time	Area	Height	Width	Area%	Symmetry
1	27.033	12197	213.2	0.9534	50.519	1.236
2	31.369	11946.4	163.1	1.0388	49.481	0.683

chiral

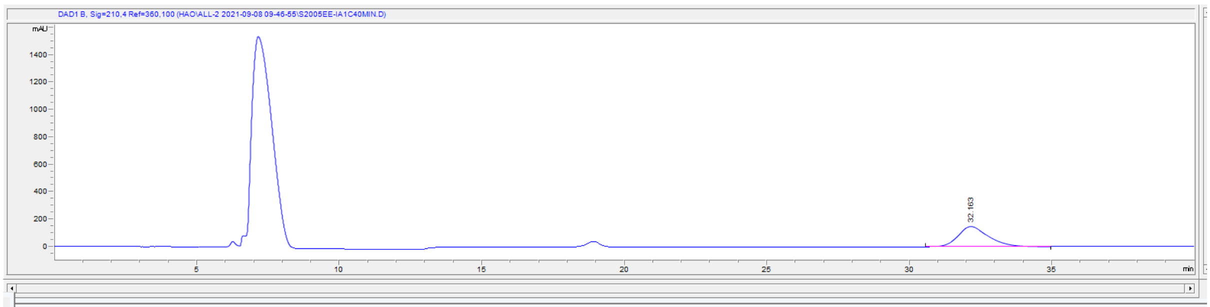

#	Time	Area	Height	Width	Area%	Symmetry
1	32.163	11051.6	149.1	1.0326	100.000	0.694

#	Time	Area	Height	Width	Area%	Symmetry
1	32.163	11051.6	149.1	1.0326	100.000	0.694

Condition: DAD1 B, Sig=210, Ref=360; Column IA, 10% iPrOH in *iso*-hexane, 60min

Ring contraction of *N*-aminopyrrolidine

Synthesis of *N*-aminopyrrolidine (compound **54**)

Under ambient condition, the pyrrolidine **31** (0.5 g, 1.35 mmol), $NaNO_2$ (0.187 g., 2.71 mmol, 2 eq.) and were dissolved in 100 ml DCM at 0°C. Then acetic acid (0.116 ml, 2.03 mmol, 1.5 eq.) and water (0.122 ml, 6.77 mmol, 5 eq.) were added to the reaction mixture. Ice-water bath was removed and the reaction was stirred for 5 hours in room temperature, in which the complete consumption of starting material can be monitored by TLC. The crude reaction mixture was transferred to the separation funnel and was diluted with DCM. Saturated aqueous sodium bicarbonate was added. Repeat extraction of the aqueous phase using DCM (100 ml) for three times. Combined organic phase was then dried by $MgSO_4$. Solvent were evaporated under vacuum to give the *N*-nitrosylation product as a yellow solid. Flash chromatography of this crude product gave the *N*-nitrosylated pyrrolidine in 68% yield (367 mg, 0.92 mmol) in the form of yellow solid (eluent: from 10:1 to 2:1 petroleum ether: ethyl acetate). *N*-nitrosylated pyrrolidine (367 mg, 0.92 mmol) was dissolved in MeOH and was cooled down to 0°C. Zinc (0.602 g, 9.21 mmol, 10 eq.) and NH_4Cl (0.739 g, 13.82 mmol, 15 eq.) were added to the reaction. Reaction mixture was stirred at 0°C for 1h. The crude reaction mixture was filtered through a pad of celite. The filtrate was evaporated and the crugde mixture was purified by flash chromatography to give the *N*-aminopyrrolidine **36** as a white solid. (210 mg, 0.55 mmol 59% yield)

rac-Dimethyl (2*R*,3*S*,4*R*,5*S*)-1-amino-5-(4-methoxyphenyl)-3-phenylpyrrolidine-2,4-dicarboxylate (compound **54**)

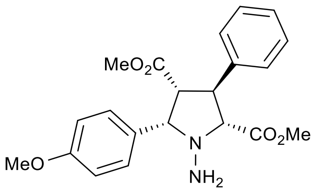

Isolated yield: 59% (white solid) (silica gel, 1:10 = ethyl acetate:petroleum ether to 1:5 = ethyl acetate:petroleum ether)
^{1}H NMR (700 MHz, CD_2Cl_2) δ 7.50 – 7.30 (m, 4H), 7.30 – 7.22 (m, 3H), 6.88 (d, *J* = 8.7 Hz, 2H), 4.06 (d, *J* = 10.8 Hz, 1H), 4.04 – 4.00 (m, 1H), 3.79 (s, 3H), 3.67 (s, 3H), 3.55 (d, *J* = 10.4 Hz, 1H), 3.44 (dd, *J* = 10.8, 8.6 Hz, 1H), 3.09 ppm (s, 3H).
^{13}C NMR (176 MHz, CD_2Cl_2) δ 172.21, 171.91, 160.06, 140.01, 130.63, 130.04, 129.30, 128.36, 127.90, 114.05, 76.89, 75.11, 56.08, 55.78, 52.51, 51.92, 48.34 ppm.
HR-MS calculated for $C_{21}H_{25}N_2O_5$ = 385.1758 $[M+H]^+$, found: 385.1767.
IR ν_{max} (cm^{-1}) 1735, 1610, 1511, 1435, 1384, 1245, 1210, 1168, 1106, 1030, 929, 843, 817.

Dimethyl (1*R*,2*s*,3*S*,4*s*)-2-(4-methoxyphenyl)-4-phenylcyclobutane-1,3-dicarboxylate (compound **52**)

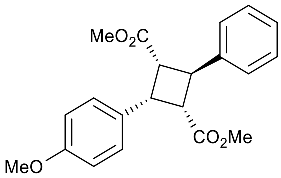

Compound 52 is prepared using procedure C.
Isolated yield: 25% (white solid) (silica gel, 1:50 = acetone:petroleum ether to 1:20 = acetone:petroleum ether)
^{1}H NMR (500 MHz, $CDCl_3$) δ 7.39 – 7.30 (m, 4H), 7.25 – 7.19 (m, 3H), 6.85 – 6.78 (m, 2H), 4.77 (dd, *J* = 10.7, 10.7 Hz, 1H), 4.16 (dd, *J* = 10.0, 10.0 Hz, 1H), 3.79 (s, 3H), 3.66 (dd, *J* = 10.4, 10.4 Hz, 2H), 3.39 ppm (s, 6H).
^{13}C NMR (126 MHz, $CDCl_3$) δ 171.82, 158.61, 141.82, 129.41, 129.29, 128.59, 126.81, 126.45, 113.63, 55.15, 51.65, 43.78, 41.59 ppm.
HR-MS calculated for $C_{21}H_{24}O_5$ = 355.1540 $[M+H]^+$, found: 355.1543.
IR ν_{max} (cm^{-1}) 1728, 1611, 1514, 1435, 1249, 1201, 1181, 1031, 836.

Oxidation of pyrrolidine as side reaction

Synthesis of compound **7**

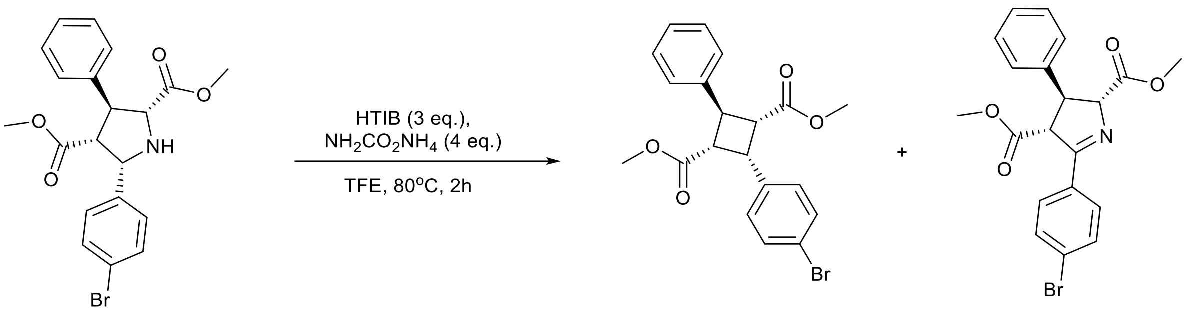

4 8 7

32% 38%

Scheme S3. Oxidation of pyrrolidine was observed when 4 eq. of ammonium carbamate was used.

Under ambient atmosphere, HTIB (98 mg, 0.25 mmol, 2.5 eq.), ammonium carbamate (62 mg, 0.8 mmol, 4 eq.) and pyrrolidine (0.1 mmol, 1 eq.) were dissolved in 1 mL 2,2,2-trifluoroethanol and stirred at 80°C for two hours. The reaction vial was cooled down to room temperature and the vial cap was opened slowly. The reaction mixture was filtered through a cotton wool and the filtrate was concentrated under vacuum. The crude mixture was directly charged onto silica gel and the imine was isolated using petroleum ether / acetone (10:1 v:v) as eluent.

rac-Dimethyl (2*R*,3*S*,4*R*)-5-(4-bromophenyl)-3-phenyl-3,4-dihydro-2H-pyrrole-2,4-dicarboxylate (compound **7**)

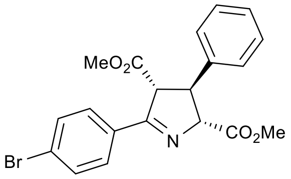

Isolated yield: 38% (white solid) (silica gel, 1:50 = acetone:petroleum ether)
^{1}H NMR (500 MHz, CD_2Cl_2) δ 7.86 – 7.70 (m, 2H), 7.59 (d, *J* = 8.7 Hz, 2H), 7.39 – 7.30 (m, 4H), 7.27 – 7.19 (m, 1H), 4.97 (dd, *J* = 6.1, 1.6 Hz, 1H), 4.41 – 4.28 (m, 1H), 4.14 (t, *J* = 6.3 Hz, 1H), 3.78 (s, 3H), 3.65 ppm (s, 3H).
^{13}C NMR (126 MHz, CD_2Cl_2) δ 171.16, 170.98, 169.81, 141.11, 131.85, 131.77, 131.12, 129.67, 129.08, 127.57, 127.03, 81.58, 62.45, 52.69, 52.47, 52.31 ppm.
HR-MS calculated for $C_{20}H_{19}{}^{79}BrNO_4$ = 416.0492, $[M+H]^+$, found: 416.0498; calculated for $C_{20}H_{19}{}^{81}BrNO_4$ = 418.0472, $[M+H]^+$, found: 418.0474;
IR ν_{max} (cm^{-1}) 1735, 1617, 1589, 1489, 1434, 1396, 1262, 1197, 1161, 1072, 1009, 827.

Radical trap experiment

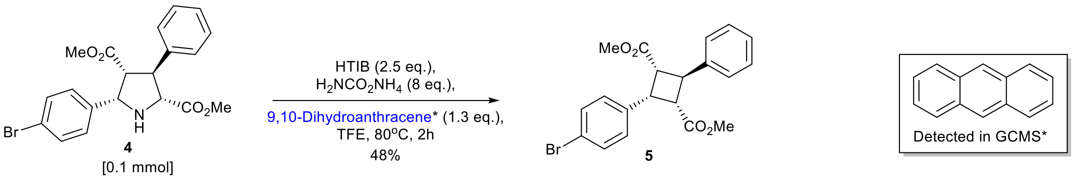

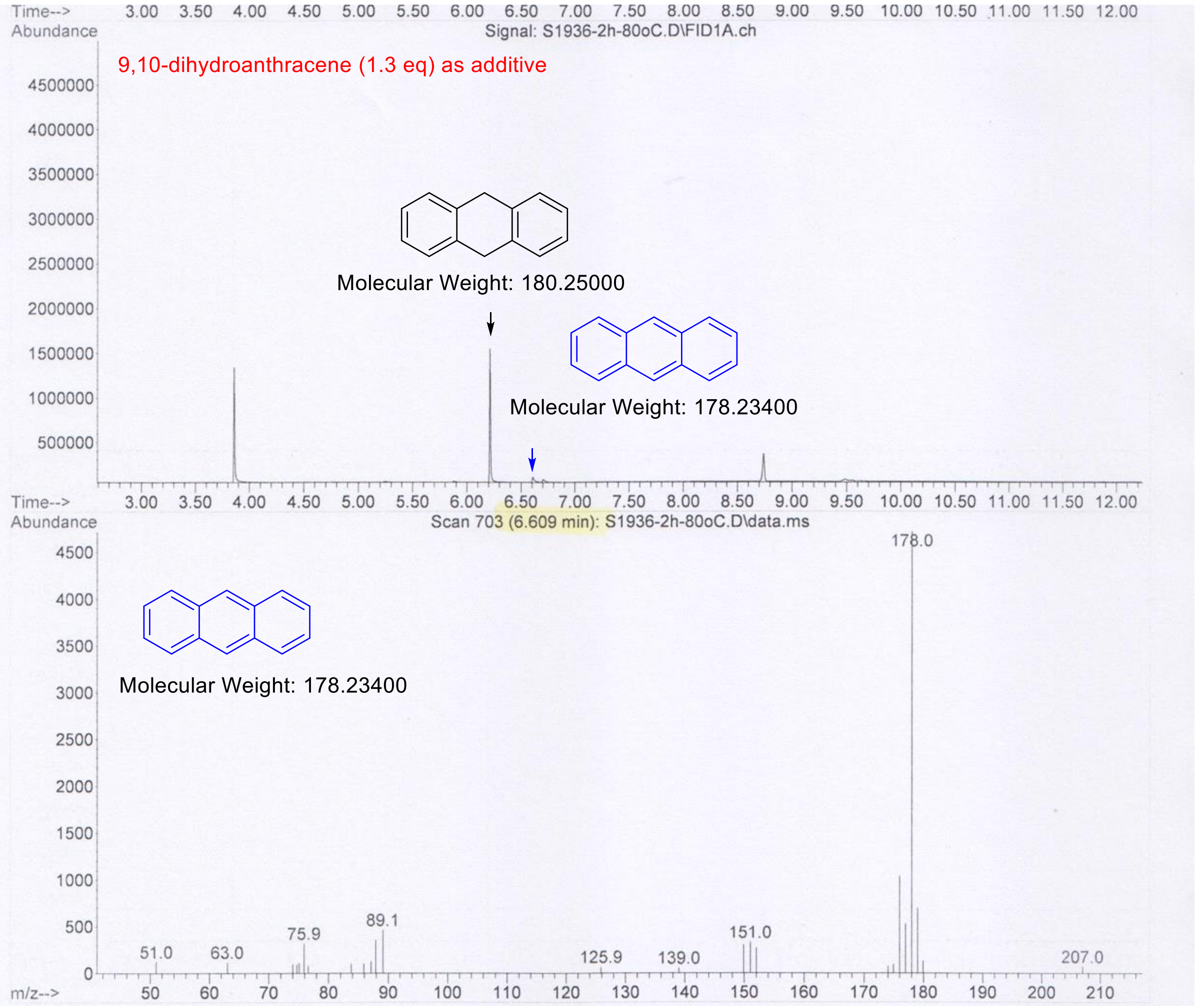

GCMS result: Standard condition with 0.1 mmol of substrate and 1.3 eq. of 9,10-dihydroanthracene as additive, anthracene was detected at 6.609 min.

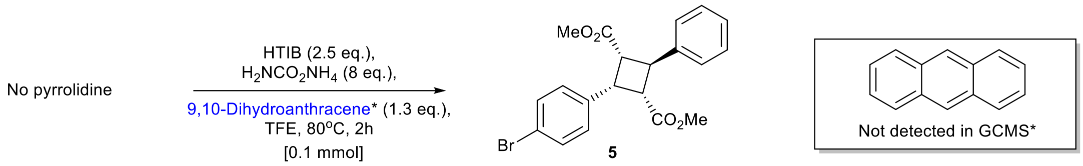

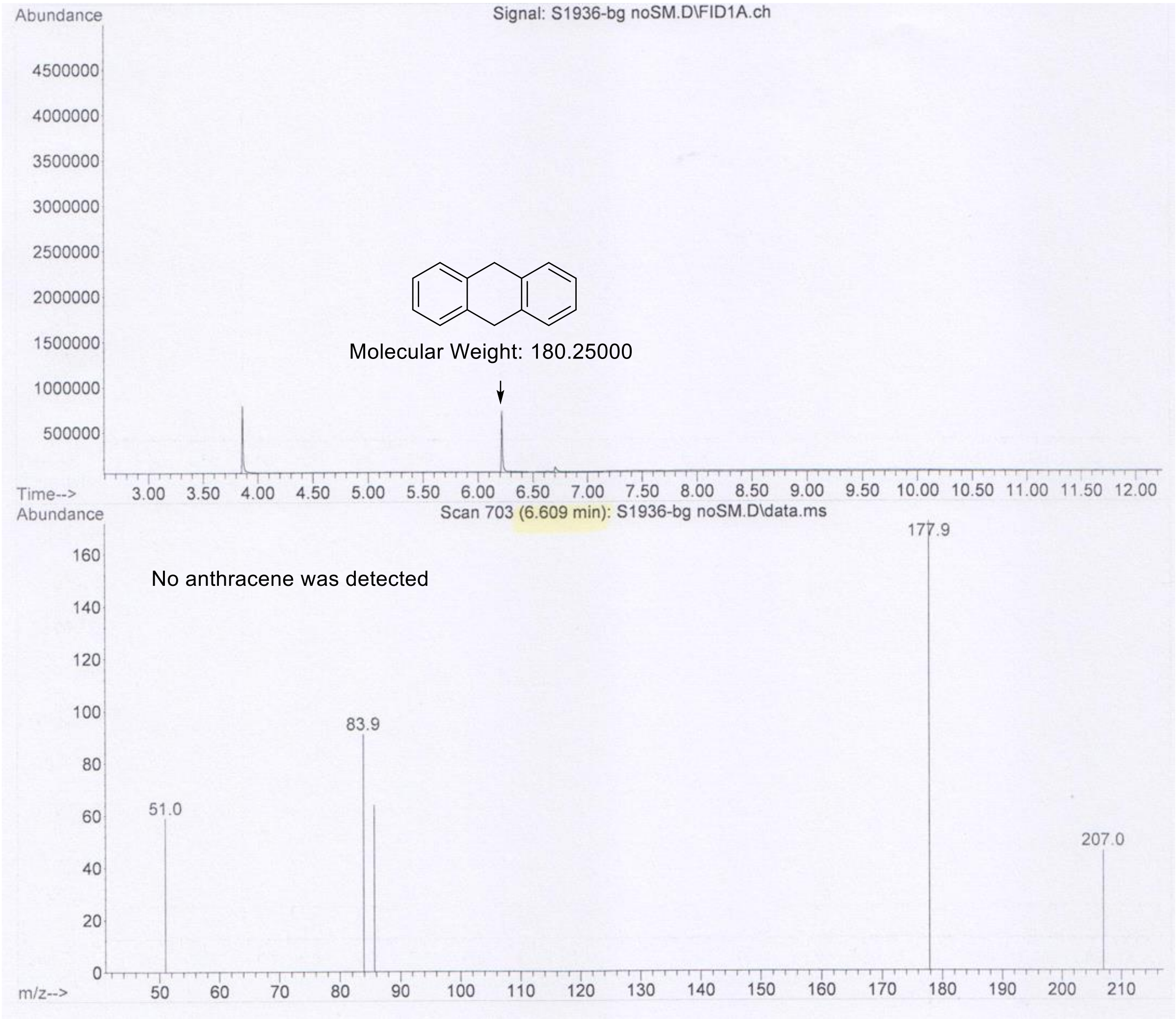

GCMS result: Control experiment without adding starting material, anthracene was not detected at 6.609 min.

GCMS spectrum of commercial available anthracene

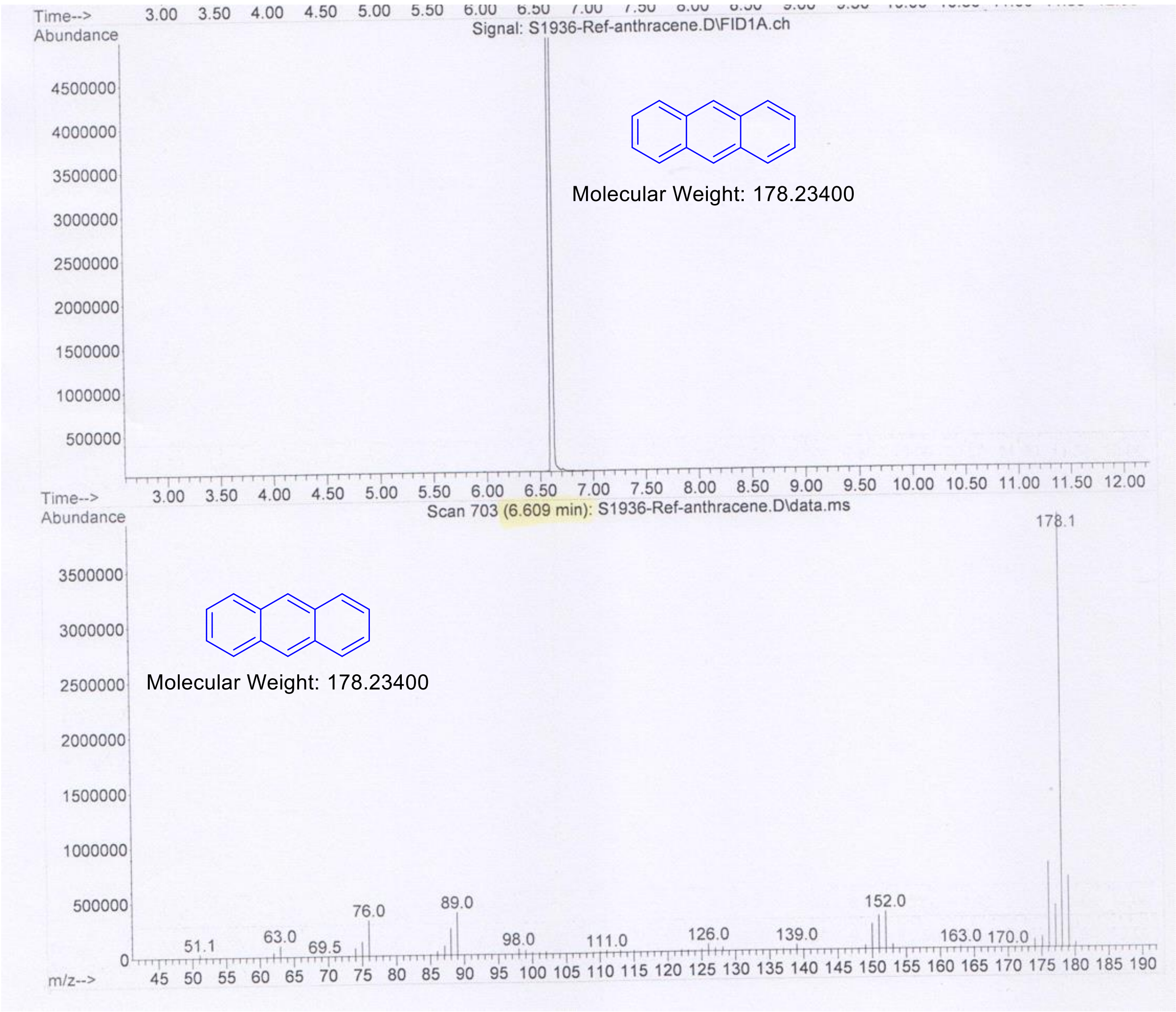

GCMS spectrum of anthracene (peak at 6.609 min)

X-ray diffraction (XRD) analysis

The crystal structures of compounds of **5**, **7**, **25**, **31**, **34**, **37, 44** and **51** was determined using the *Bruker D8 Venture* four-circle diffractometer equipped with a *PHOTON II* CPAD detector by *Bruker AXS GmbH*. The X-ray radiation was generated by the *IμS* microfocus source Mo (□ = 0.71073 Å) from *Incoatec GmbH* equipped with HELIOS mirror optics and a single-hole collimator by *Bruker AXS GmbH*. The selected single crystal of **5**, **7**, **25**, **31**, **34**, **37, 44** and **51** were covered with an inert oil (perfluoropolyalkyl ether) and mounted on the *MicroMount* from *MiTeGen*. The APEX 3 Suite (v.2019.1-0) software integrated with SAINT (integration) and SADABS (adsorption correction) programs by *Bruker AXS GmbH* were used for data collection. The processing and finalization of the crystal structure were performed using the Olex2 program.[8] The crystal structures were solved by the ShelXT[9] structure solution program using the Intrinsic Phasing option, which were further refined by the ShelXL[10] refinement package using Least Squares minimization. The non-hydrogen atoms were anisotropically refined. The C-bound H atoms were placed in geometrically calculated positions, and a fixed isotropic displacement parameter was assigned to each atom according to the riding-model: C–H = 0.95–1.00 Å with *U*iso(H) = 1.5*U*eq(CH3) and 1.2*U*eq(CH_2, CH) for other hydrogen atoms. The N-bound hydrogen atoms were located on the Difference-Fourier-Map and refined independently in every structure. The crystallographic data for the structures of **5**, **7**, **25**, **31**, **34**, **37, 44** and **51** has been published as supplementary publication number 2080159 (**B1484**), 2080166 (**B1681**), 2080174 (**B1692**), 2080185 (**B1808**), 2080194 (**B1832**), 2080203 (**B1956**), 2080204 (**B2074**) and 2105004 (**B2296**) in the Cambridge Crystallographic Data Centre. A copy of these data can be obtained for free by applying to CCDC, 12 Union Road, Cambridge CB2 IEZ, UK, fax: 144-(0)1223-336033 or e-mail: deposit@ccdc.cam.ac.uk.

X-ray structure of compound **51** (CCDC: 2080159)

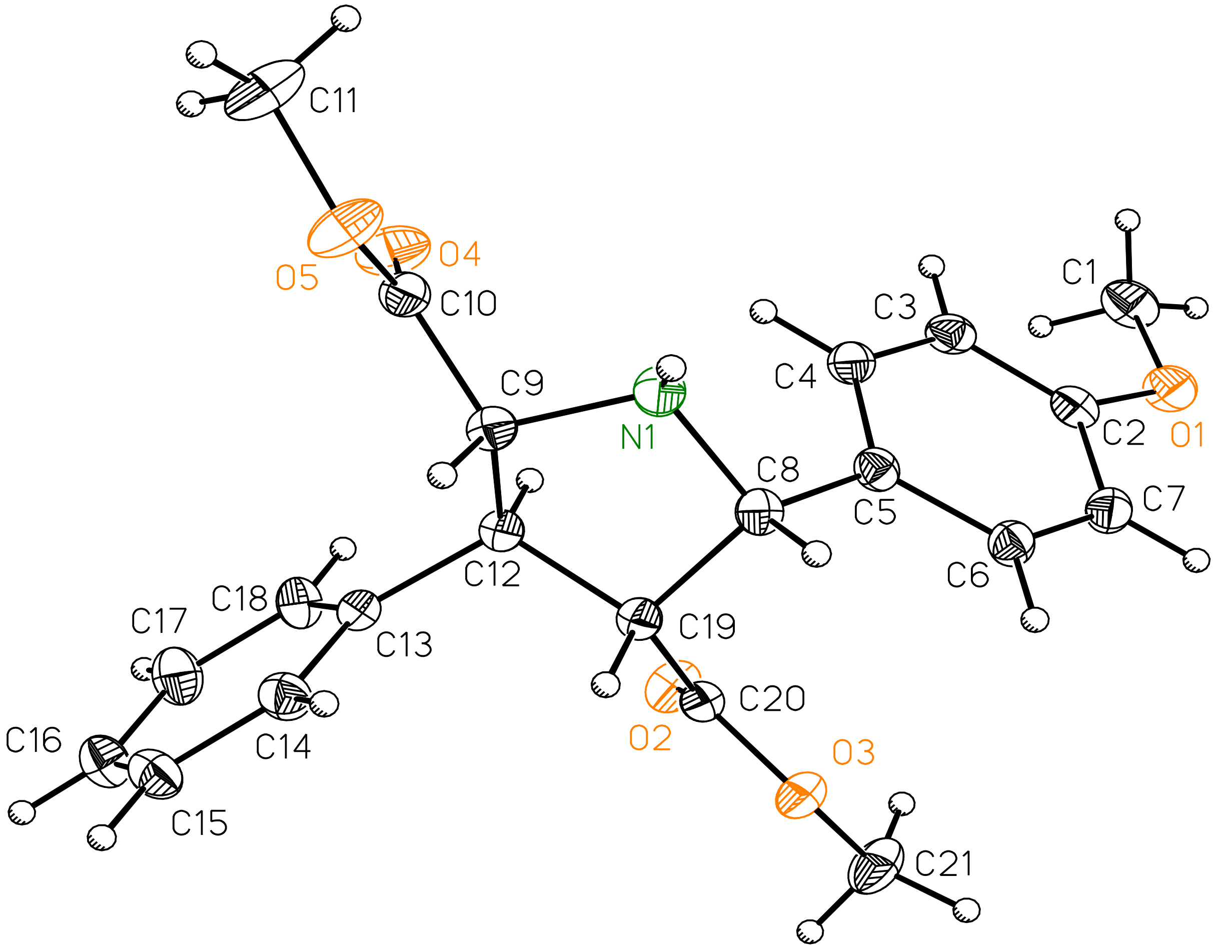

Figure S1 Ortep plot of the molecular structure in the crystal of compound **51 (B1484)**.[11] The displacement ellipsoids are drawn at the 50% probability level. Co-crystallized solvent has been omitted for clarity.

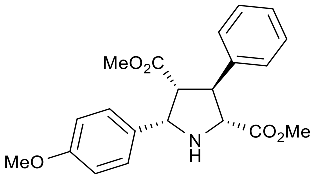

Compound **51**

Table Crystallographic data of compound **51 (B1484)**.

Compound	**51 (B1484)**
Empirical formula	$C_{21}H_{23}NO_5$
Formula weight	369.40
Temperature/K	100.0
Crystal system	monoclinic
Space group	$P2_1/n$
a/Å	5.7105(3)
b/Å	36.9090(18)
c/Å	8.9359(4)
α/°	90
β/°	90.225(2)
γ/°	90
Volume/Å^3	1883.39(16)
Z	4
ρ_{calc}g/cm^3	1.303
μ/mm^{-1}	0.093
F(000)	784.0
Crystal size/mm^3	0.782 × 0.347 × 0.245
Radiation	MoKα (λ = 0.71073)
2Θ range for data collection/°	5.064 to 61.098
Index ranges	$-8 \le h \le 8$, $-52 \le k \le 52$, $-12 \le l \le 12$
Reflections collected	52374
Independent reflections	5742 [R_{int} = 0.0389, R_{sigma} = 0.0230]
Data/restraints/parameters	5742/0/251
Goodness-of-fit on F^2	1.189
Final *R* indexes [I>=2σ (I)]	R_1 = 0.0558, wR_2 = 0.1377
Final *R* indexes [all data]	R_1 = 0.0571, wR_2 = 0.1383
Largest diff. peak/hole / e Å^{-3}	0.53/–0.21
Flack parameter	–

X-ray structure of compound **5** (CCDC: 2080174)

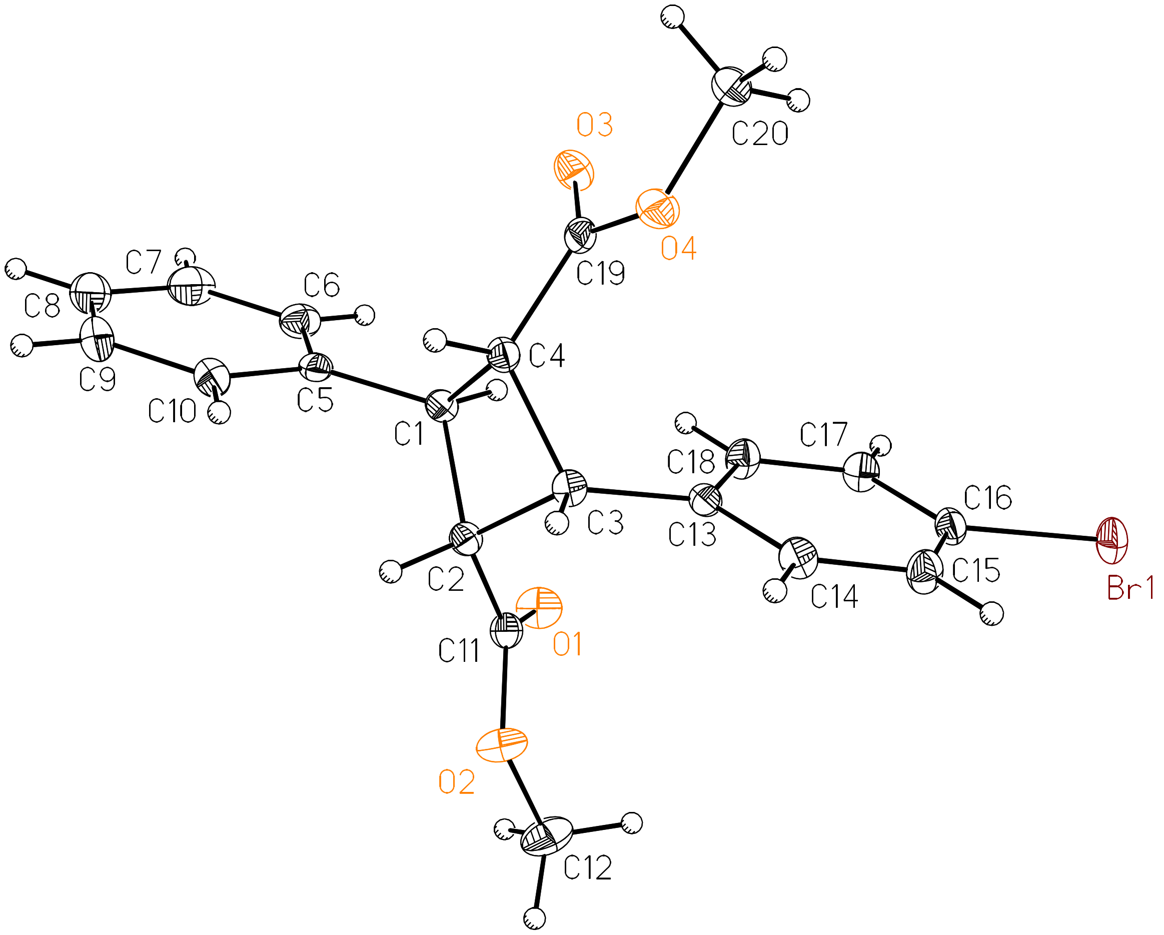

Figure S1 Ortep plot of the molecular structure in the crystal of compound **5 (B1692)**.[11] The displacement ellipsoids are drawn at the 50% probability level.

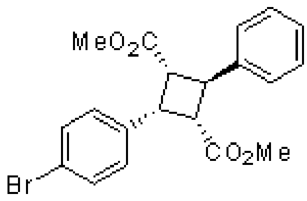

Compound **5**

Table Crystallographic data of compound **5 (B1692)**.

Compound	**5 (B1692)**
Empirical formula	$C_{20}H_{19}BrO_4$
Formula weight	403.26
Temperature/K	100.0
Crystal system	monoclinic
Space group	$P2_1/n$
a/Å	5.683(3)
b/Å	21.449(9)
c/Å	14.809(5)
α/°	90
β/°	97.565(16)
γ/°	90
Volume/Å^3	1789.4(14)
Z	4
ρ_{calc}g/cm^3	1.497
μ/mm^{-1}	2.319
F(000)	824.0
Crystal size/mm^3	0.336 × 0.082 × 0.066
Radiation	MoKα (λ = 0.71073)
2Θ range for data collection/°	5.55 to 59.996
Index ranges	$-7 \leq h \leq 7$, $-30 \leq k \leq 30$, $-20 \leq l \leq 20$
Reflections collected	52874
Independent reflections	5196 [R_{int} = 0.0397, R_{sigma} = 0.0205]
Data/restraints/parameters	5196/0/228
Goodness-of-fit on F^2	1.037
Final *R* indexes [I>=2σ (I)]	R_1 = 0.0255, wR_2 = 0.0579
Final *R* indexes [all data]	R_1 = 0.0316, wR_2 = 0.0604
Largest diff. peak/hole / e Å^{-3}	0.48/–0.32
Flack parameter	–

X-ray structure of compound **31** (CCDC: 2080166)

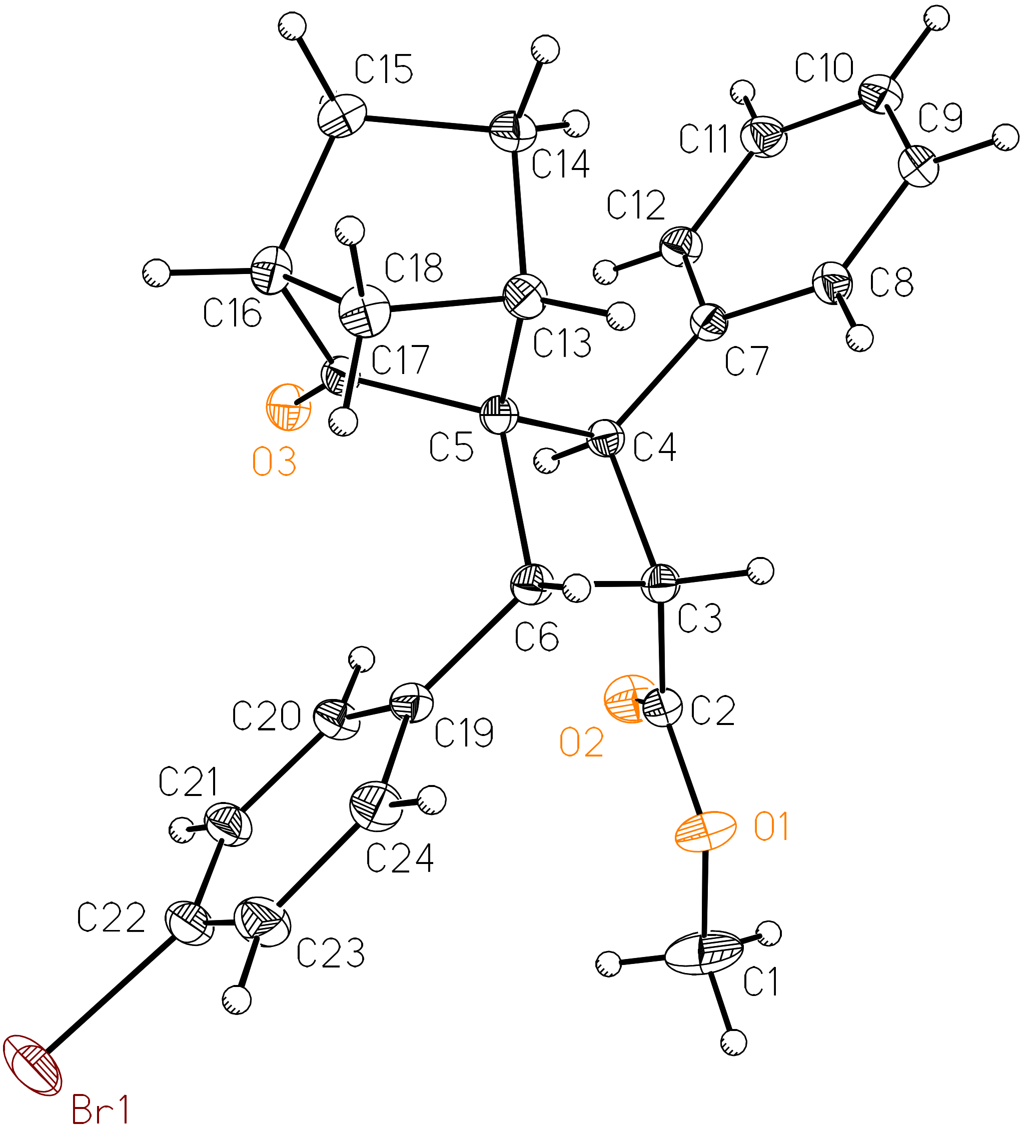

Figure S2 Ortep plot of the molecular structure in the crystal of compound **31 (B1681)**. [11]The displacement ellipsoids are drawn at the 50% probability level.

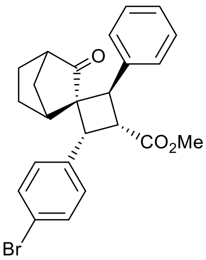

Compound **31**

Table Crystallographic data of compound **31 (B1681)**.

Compound	**31 (B1681)**
Empirical formula	$C_{24}H_{23}BrO_3$
Formula weight	439.33
Temperature/K	100.0
Crystal system	triclinic
Space group	*P*-1
a/Å	6.1786(4)
b/Å	7.7074(5)
c/Å	22.9571(12)
α/°	84.833(2)
β/°	85.296(2)
γ/°	70.319(2)
Volume/Å^3	1023.63(11)
Z	2
ρ_{calc}g/cm^3	1.425
μ/mm^{-1}	2.030
F(000)	452.0
Crystal size/mm^3	0.433 × 0.294 × 0.092
Radiation	MoKα (λ = 0.71073)
2Θ range for data collection/°	5.354 to 69.998
Index ranges	$-9 \le h \le 9$, $-12 \le k \le 12$, $-37 \le l \le 37$
Reflections collected	106331
Independent reflections	9005 [R_{int} = 0.0361, R_{sigma} = 0.0134]
Data/restraints/parameters	9005/0/254
Goodness-of-fit on F^2	1.031
Final *R* indexes [I>=2σ (I)]	R_1 = 0.0292, wR_2 = 0.0783
Final *R* indexes [all data]	R_1 = 0.0323, wR_2 = 0.0801
Largest diff. peak/hole / e Å^{-3}	0.92/–0.84
Flack parameter	–

X-ray structure of compound **34** (CCDC: 2080203)

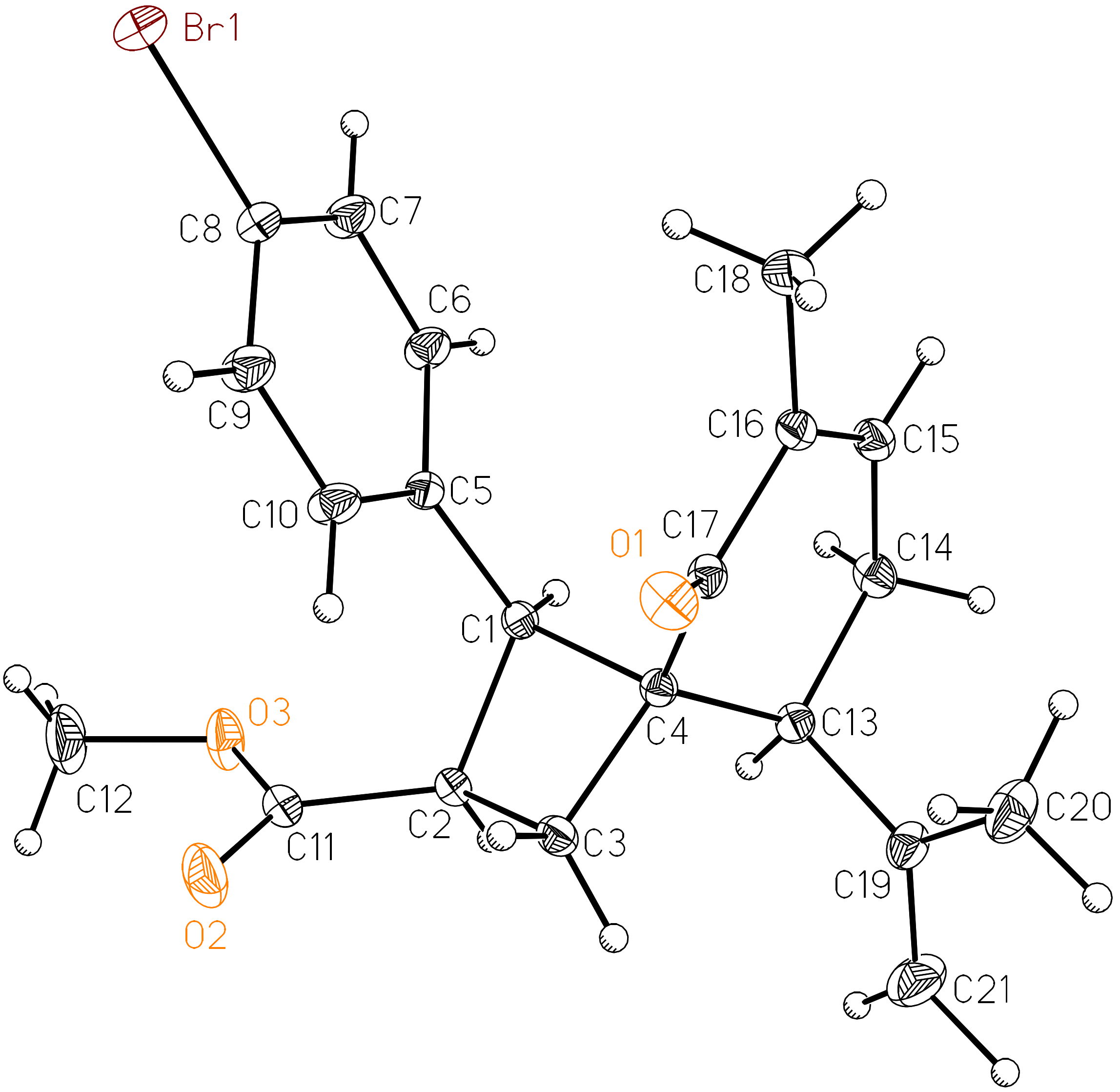

Figure S3 Ortep plot of the molecular structure in the crystal of compound **34 (B1956)**. [11] The displacement ellipsoids are drawn at the 50% probability level. Co-crystallized solvent has been omitted for clarity.

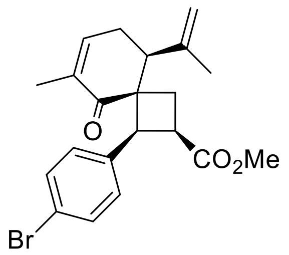

Compound **34**

Table Crystallographic data of compound **34 (B1956)**.

Compound	**34 (B1956)**
Empirical formula	$C_{21}H_{23}BrO_3$
Formula weight	403.30
Temperature/K	100.0
Crystal system	trigonal
Space group	$P3_1$
a/Å	10.3555(5)
b/Å	10.3555(5)
c/Å	15.2451(9)
α/°	90
β/°	90
γ/°	120
Volume/Å^3	1415.80(16)
Z	3
ρ_{calc}g/cm^3	1.419
μ/mm^{-1}	2.194
F(000)	624.0
Crystal size/mm^3	0.334 × 0.396 × 0.312
Radiation	MoKα (λ = 0.71073)
2Θ range for data collection/°	4.542 to 72.674
Index ranges	$-17 \le h \le 17$, $-17 \le k \le 17$, $-25 \le l \le 25$
Reflections collected	257036
Independent reflections	9159 [R_{int} = 0.0515 R_{sigma} = 0.0146]
Data/restraints/parameters	9159/1/238
Goodness-of-fit on F^2	1.038
Final *R* indexes [I>=2σ (I)]	R_1 = 0.0175, wR_2 = 0.0468
Final *R* indexes [all data]	R_1 = 0.0184, wR_2 = 0.0472
Largest diff. peak/hole / e Å^{-3}	0.34/–0.23
Flack parameter	–0.0029(13)

X-ray structure of compound **37** (CCDC: 2080185)

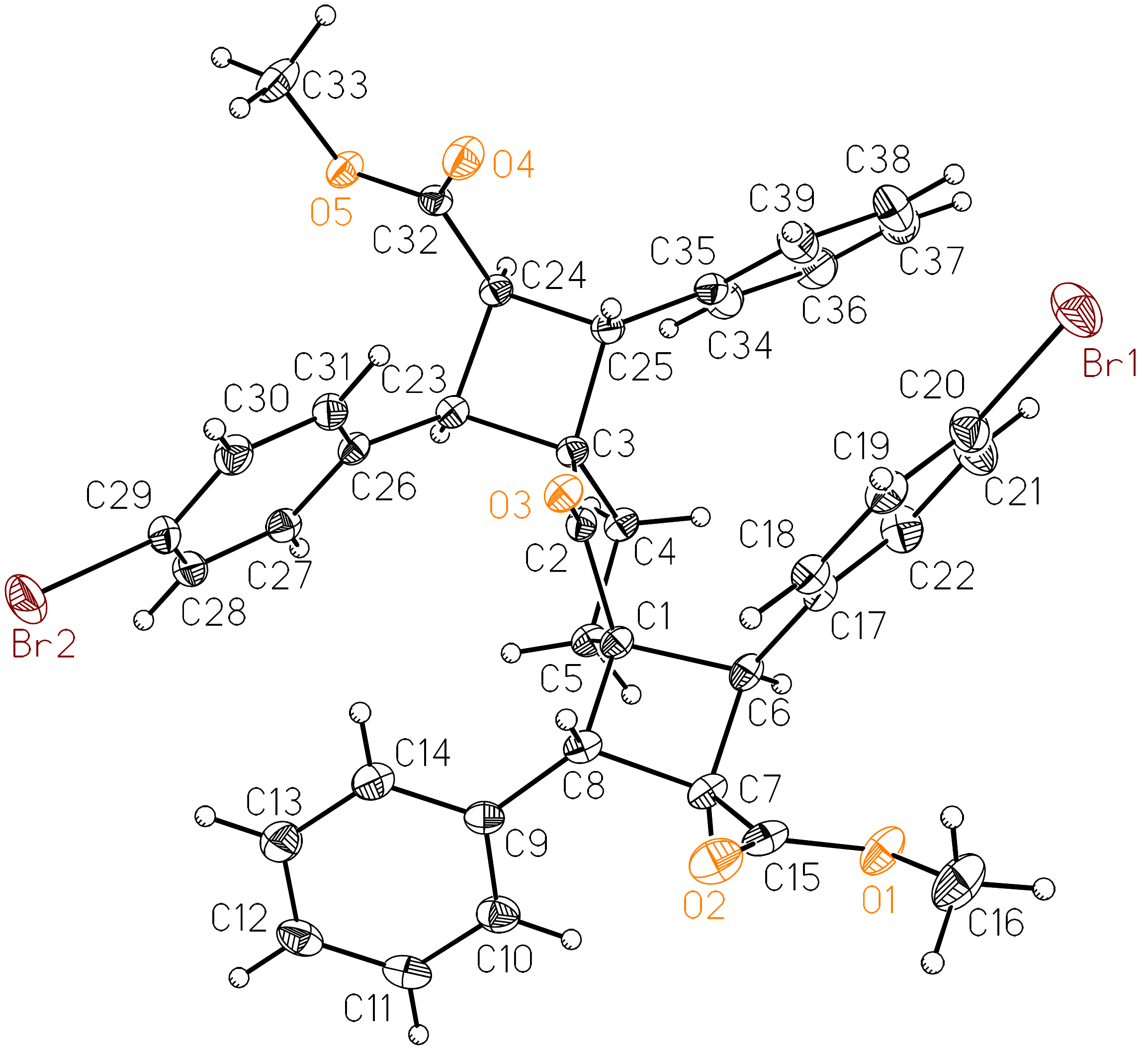

Figure S4 Ortep plot of the molecular structure in the crystal of compound **37 (B1808).** [11] The displacement ellipsoids are drawn at the 50% probability level.

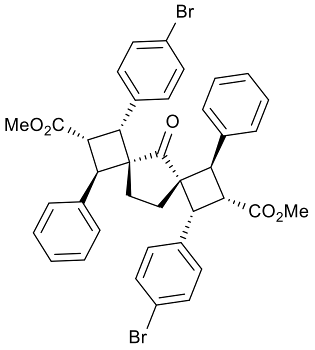

Compound **37**

Table Crystallographic data of compound **37 (B1808)**.

Compound	**37 (B1808)**
Empirical formula	$C_{39}H_{34}Br_2O_5$
Formula weight	742.48
Temperature/K	100.0
Crystal system	monoclinic
Space group	$P2_1/c$
a/Å	11.456(16)
b/Å	23.373(18)
c/Å	13.367(11)
α/°	90
β/°	112.38(4)
γ/°	90
Volume/Å^3	3310(6)
Z	4
ρ_{calc}g/cm^3	1.490
μ/mm^{-1}	2.493
F(000)	1512.0
Crystal size/mm^3	0.514 × 0.129 × 0.035
Radiation	MoKα (λ = 0.71073)
2Θ range for data collection/°	4.222 to 61.16
Index ranges	$-16 \le h \le 16$, $-32 \le k \le 33$, $-19 \le l \le 19$
Reflections collected	105668
Independent reflections	10155 [R_{int} = 0.0461, R_{sigma} = 0.0247]
Data/restraints/parameters	10155/0/417
Goodness-of-fit on F^2	1.023
Final R indexes [I>=2σ (I)]	R_1 = 0.0307, wR_2 = 0.0691
Final R indexes [all data]	R_1 = 0.0456, wR_2 = 0.0747
Largest diff. peak/hole / e Å^{-3}	0.73/–1.04
Flack parameter	–

X-ray structure of compound **44** (CCDC: 2080204)

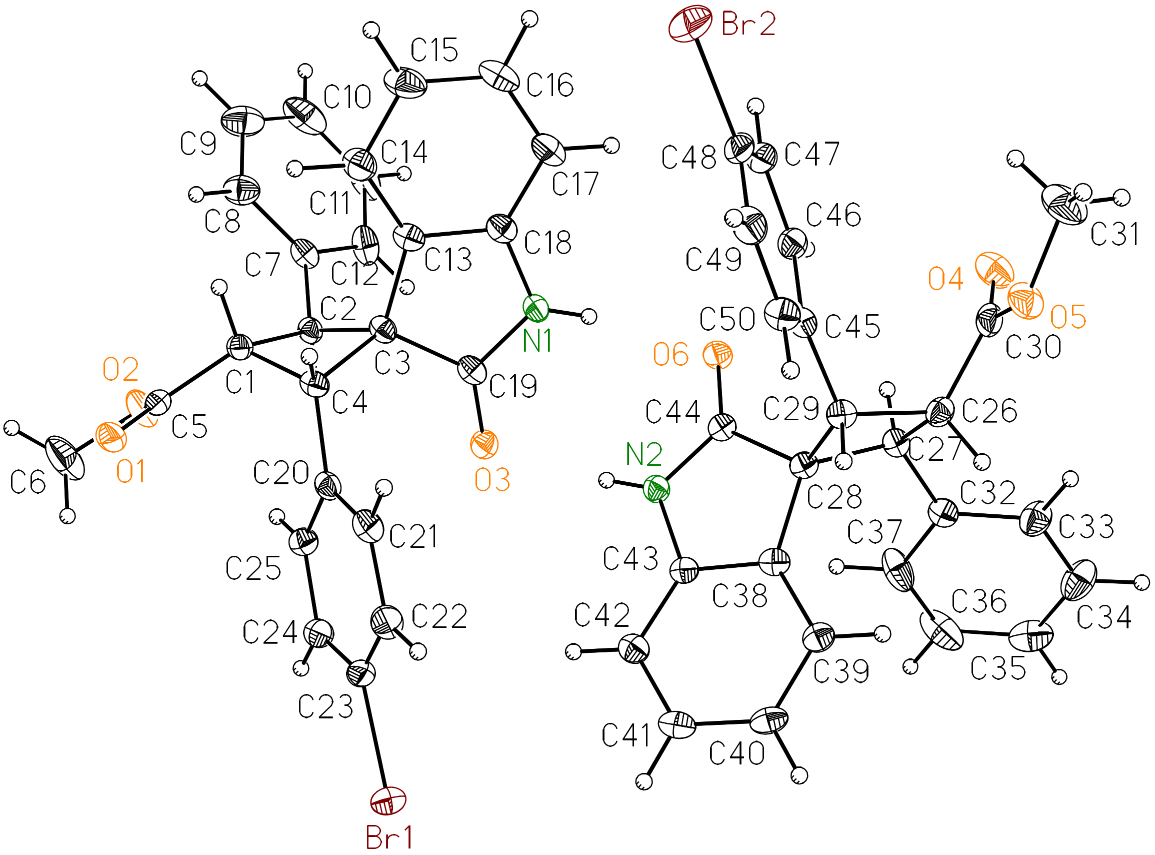

Figure S5 Ortep plot of the molecular structure in the crystal of compound **44 (B2074)**. [11]The displacement ellipsoids are drawn at the 50% probability level. Co-crystallized solvent has been omitted for clarity.

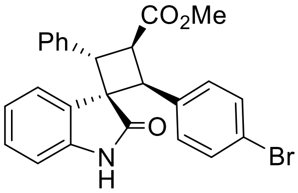

Compound **44**

Table Crystallographic data of compound **44 (B2074)**.

Compound	**44 (B2074)**
Empirical formula	$C_{25}H_{20}BrNO_3$
Formula weight	462.33
Temperature/K	100.0
Crystal system	orthorhombic
Space group	$P2_12_12_1$
a/Å	11.150(3)
b/Å	16.942(8)
c/Å	22.754(5)
α/°	90
β/°	90
γ/°	90
Volume/Å^3	4298(2)
Z	8
ρ_{calc}g/cm^3	1.429
μ/mm^{-1}	1.939
F(000)	1888.0
Crystal size/mm^3	0.845 × 0.053 × 0.027
Radiation	MoKα (λ = 0.71073)
2Θ range for data collection/°	4.068 to 60.992
Index ranges	–15 ≤ h ≤ 15, –24 ≤ k ≤ 24, –32 ≤ l ≤ 32
Reflections collected	56317
Independent reflections	13094 [R_{int} = 0.0406, R_{sigma} = 0.0374]
Data/restraints/parameters	13094/0/551
Goodness-of-fit on F^2	1.023
Final *R* indexes [I>=2σ (I)]	R_1 = 0.0322, wR_2 = 0.0654
Final *R* indexes [all data]	R_1 = 0.0440, wR_2 = 0.0695
Largest diff. peak/hole / e Å^{-3}	0.69/–0.59
Flack parameter	–0.007(2)

X-ray structure of compound **7** (CCDC: 2080194)

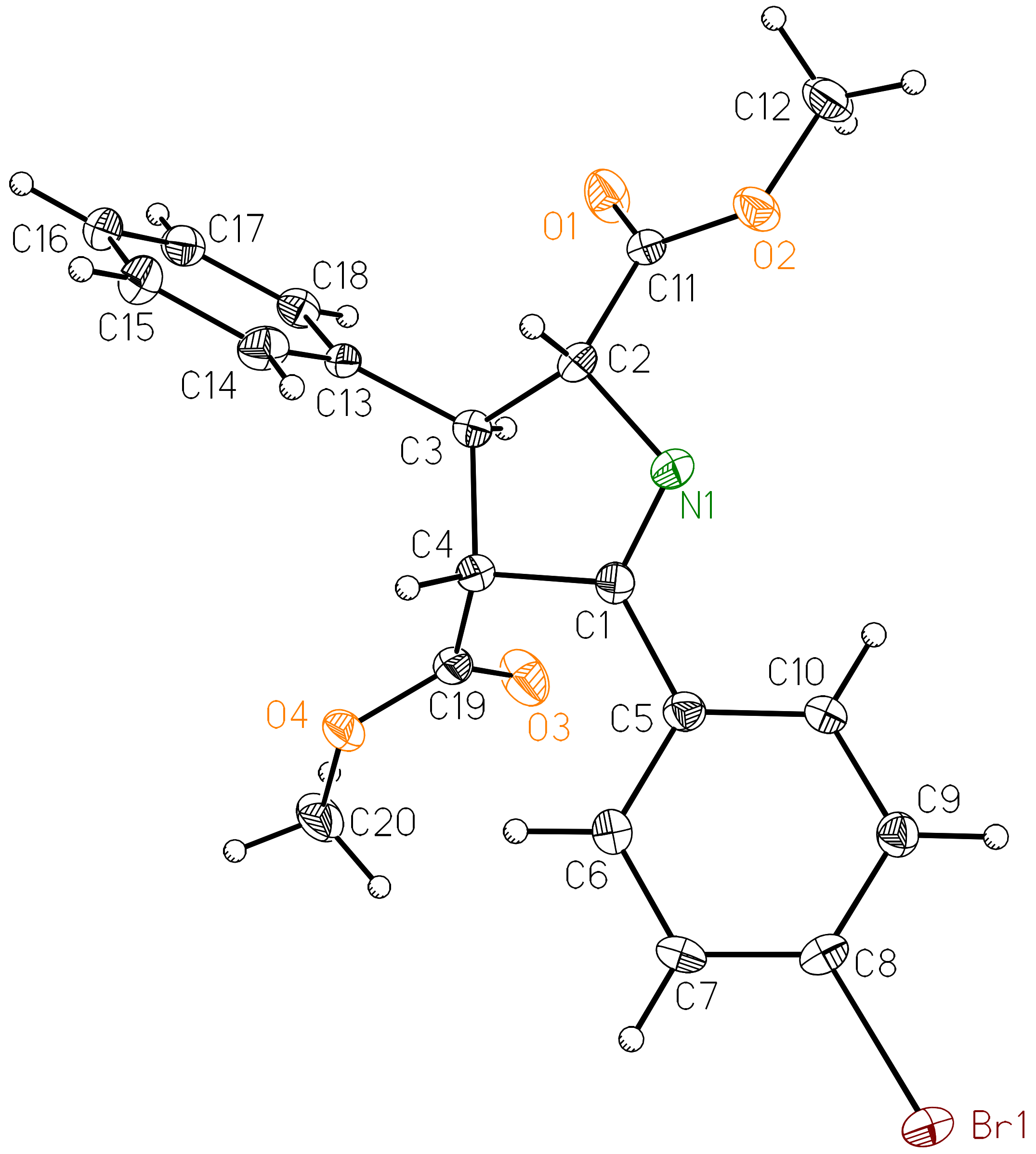

Figure S6 Ortep plot of the molecular structure in the crystal of compound **7 (B1832)**. [11] The displacement ellipsoids are drawn at the 50% probability level. Co-crystallized solvent has been omitted for clarity.

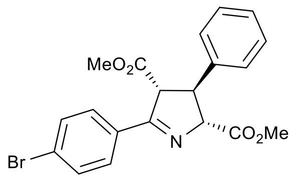

Compound **7**

Table Crystallographic data of compound **7 (B1832)**.

Compound	**7 (B1832)**
Empirical formula	$C_{20}H_{18}BrNO_4$
Formula weight	416.26
Temperature/K	100.0
Crystal system	monoclinic
Space group	*Pn*
a/Å	5.8234(8)
b/Å	9.4036(19)
c/Å	16.355(3)
α/°	90
β/°	96.446(6)
γ/°	90
Volume/Å^3	890.0(3)
Z	2
ρ_{calc}g/cm^3	1.553
μ/mm^{-1}	2.335
F(000)	424.0
Crystal size/mm^3	0.519 × 0.057 × 0.048
Radiation	MoKα (λ = 0.71073)
2Θ range for data collection/°	4.332 to 55.012
Index ranges	$-7 \leq h \leq 7$,<br>$-12 \leq k \leq 10$,<br>$-21 \leq l \leq 21$
Reflections collected	11658
Independent reflections	4093 [R_{int} = 0.0408,<br>R_{sigma} = 0.0485]
Data/restraints/parameters	4093/2/237
Goodness-of-fit on F^2	1.039
Final *R* indexes [I>=2σ (I)]	R_1 = 0.0324,<br>wR_2 = 0.0598
Final *R* indexes [all data]	R_1 = 0.0392,<br>wR_2 = 0.0627
Largest diff. peak/hole / e Å^{-3}	0.32/–0.33
Flack parameter	0.009(5)

X-ray structure of compound **25** (CCDC: 2105004)

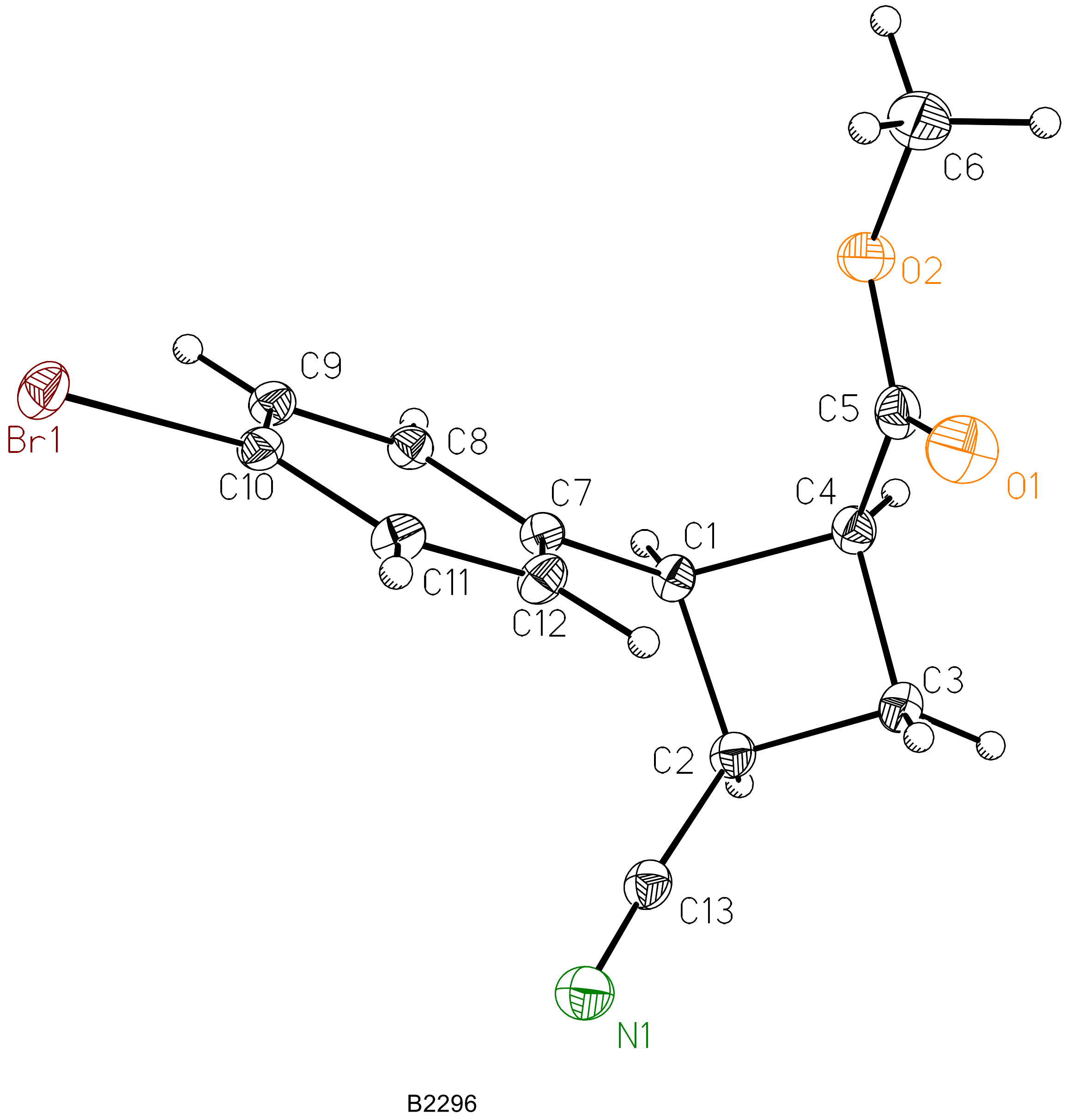

Figure S7 Ortep plot of the molecular structure in the crystal of compound **25** (**B2296**). [11] The displacement ellipsoids are drawn at the 50% probability level. Co-crystallized solvent has been omitted for clarity.

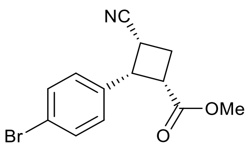

Compound **25**

Table Crystallographic data of compound **25 (B2296)**.

Compound	**25 (B2296)**
Empirical formula	$C_{10}H_{10}NOBr$
Formula weight	160.19
Temperature/K	100.00
Crystal system	monoclinic
Space group	$P2_1/n$
a/Å	12.9281(4)
b/Å	5.8766(2)
c/Å	16.0567(5)
α/°	90
β/°	96.7320(10)
γ/°	90
Volume/Å^3	1211.47(7)
Z	6
ρ_{calc}g/cm^3	1.317
μ/mm^{-1}	0.086
F(000)	510.0
Crystal size/mm^3	1.243 × 0.266 × 0.157
Radiation	MoKα (λ = 0.71073)
2Θ range for data collection/°	3.832 to 61.102
Index ranges	$-18 \le h \le 15$,<br>$-8 \le k \le 8$,<br>$-22 \le l \le 22$
Reflections collected	24890
Independent reflections	3719 [R_{int} = 0.0583,<br>R_{sigma} = 0.0326]
Data/restraints/parameters	3719/0/202
Goodness–of–fit on F^2	1.074
Final *R* indexes [I>=2σ (I)]	R_1 = 0.0245,<br>wR_2 = 0.0600
Final *R* indexes [all data]	R_1 = 0.0266,<br>wR_2 = 0.0613
Largest diff. peak/hole / e Å^{-3}	0.73/–0.28
Flack parameter	–

Reported total synthesis of piperarborenine B

The total synthesis of piperarborenine B was reported firstly by Baran and co-workers in 2011.[12] In 2016, Fox and co-workers reported the enantioselective synthesis of piperarborenin B.[13] Later in the same year, Tang and Xie reported the enantioselective synthesis of (+)-piperarborenin B.[14] (Scheme S4)

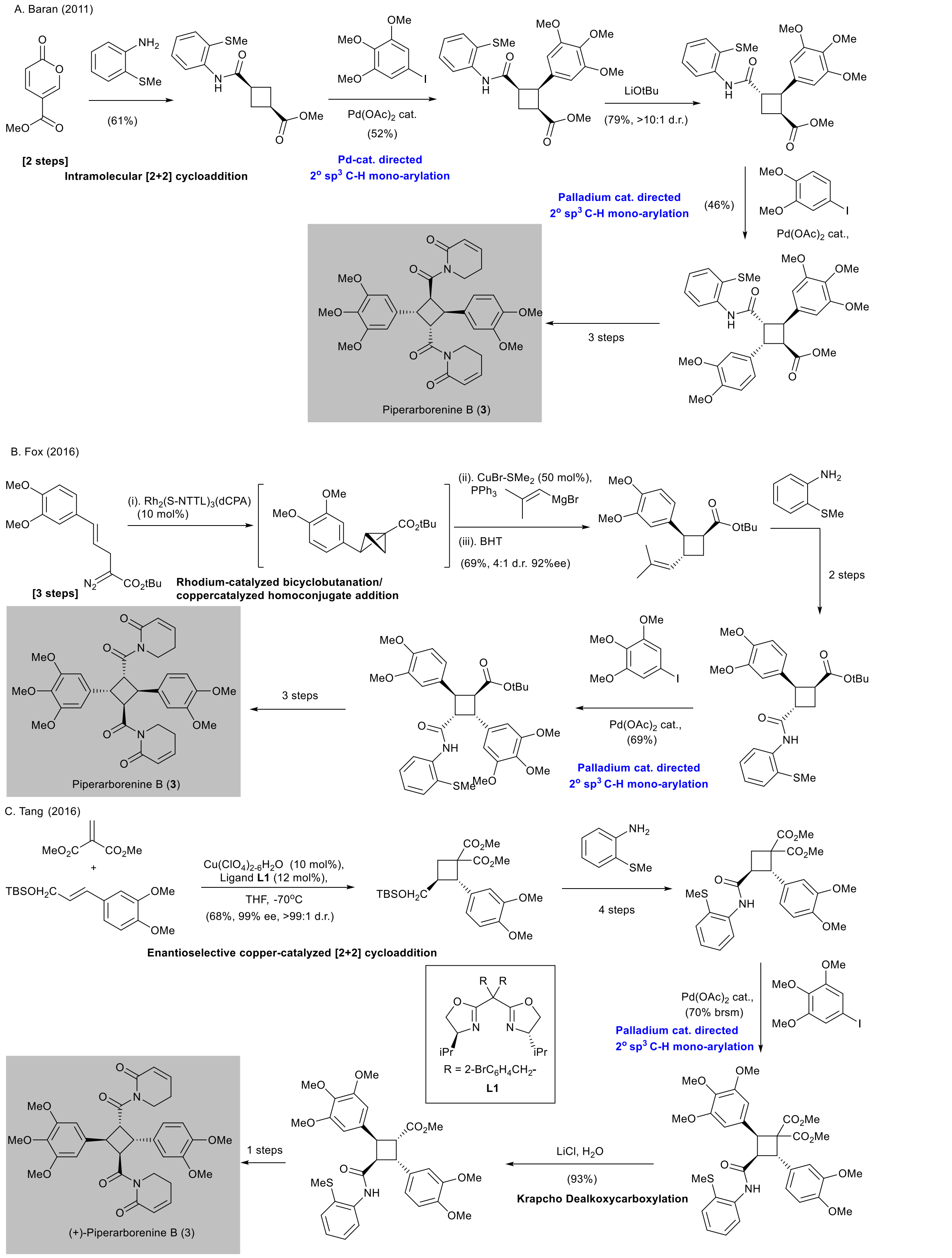

Scheme S4. Reported total synthesis of piperarborenin B.(A) Baran (2011)[12]; (B) Fox (2016)[13] and (C) Tang and Xie (2016)[14].

Formal synthesis of piperarborenine B

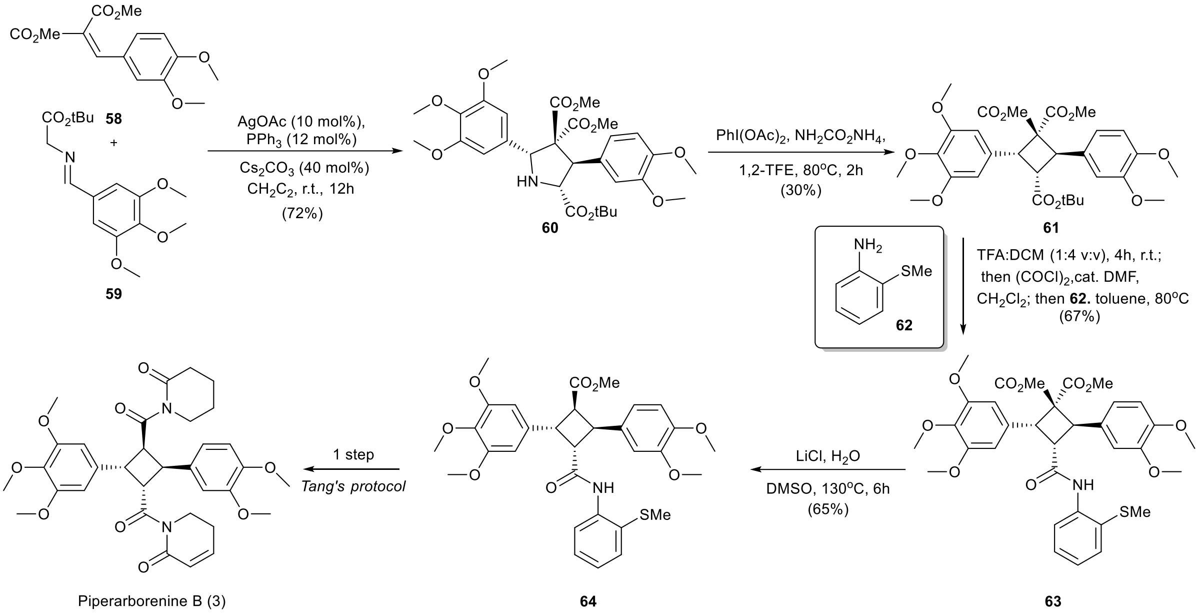

Scheme S5. Formal synthesis of piperarborenine B (*this work*)

Synthesis and characterization of synthetic intermediates

rac-2-(tert-butyl) 4,4-dimethyl (2*R*,3*R*,5*R*)-3-(3,4-dimethoxyphenyl)-5-(3,4,5-trimethoxyphenyl)pyrrolidine-2,4,4-tricarboxylate (Compound **60**) [15]

A solution of AgOAc (0.02 mmol, 0.1 equiv.) and PPh_3 (0.03 mmol, 0.12 equiv.) in DCM (0.5 mL) was stirred for 30 minutes at room temperature. Cs_2CO_3 (0.04 mmol, 0.2 equiv.), imine **59** and dimethyl benzylidenemalonate **58**[16] were added sequentially into the solution and stirred overnight. The solvent was removed under reduced pressure. Compound **39** were purified by silica gel chromatography using petroleum ether and acetone from 10:1 (v:v) to 5:1 (v:v).

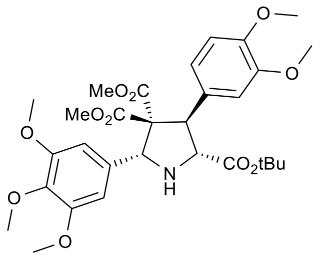

Isolated yield: 72% (white solid)
^{1}H NMR (400 MHz, CDCl$_3$) δ 6.94 – 6.77 (m, 3H), 6.71 (s, 2H), 5.21 (s, 1H), 4.25 (d, *J* = 7.9 Hz, 1H), 4.08 (d, *J* = 7.6 Hz, 1H), 4.04 (m, 3H), 3.90 – 3.85 (m, 9H), 3.82 (d, *J* = 1.3 Hz, 3H), 3.26 (d, *J* = 1.4 Hz, 3H), 3.21 (s, 3H), 1.37 ppm (s, 9H).
^{13}C NMR (101 MHz, CDCl$_3$) δ 171.57, 169.74, 152.97, 148.51, 148.35, 137.69, 134.28, 130.64, 120.97, 112.25, 110.75, 104.68, 81.76, 71.43, 68.45, 66.29, 60.85, 56.33, 56.20, 55.99, 55.86, 52.21, 52.12, 27.94 ppm.
HR-MS calculated for $C_{30}H_{40}NO_{11}$ = 590.2596 $[M+H]^+$, found: 590.2599.
IR ν_{max} (cm^{-1}) 2950, 2839, 2349, 2326, 1726, 1591, 1517, 1463, 1423, 1367, 1328, 1249, 1157, 1126, 1027, 1008, 848.

rac-3-(tert-butyl) 1,1-dimethyl (2*R*,3*S*,4*R*)-2-(3,4-dimethoxyphenyl)-4-(3,4,5-trimethoxyphenyl)cyclobutane-1,1,3-tricarboxylate (Compound **61**)

Under ambient atmosphere, PIDA (2.5 eq.), ammonium carbamate (8 eq.), pyrrolidine **60** (0.1 mmol, 1 eq.) were dissolved in 1 mL TFE and stirred at 80°C for two hours. The reaction vial was cooled down to room temperature and the vial cap was opened slowly. The reaction mixture was filtered through a cotton wool and the filtrate was concentrated under vacuum. The crude product **61** was purified by column chromatography using petroleum ether and acetone from 20:1 (v:v) to 10:1 (v:v). to give the cyclobutene product.

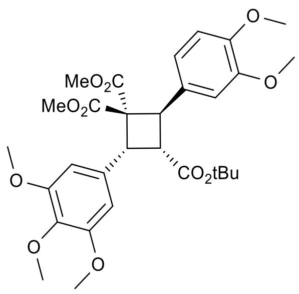

Isolated yield: 30% (yellow solid)
^{1}H NMR (400 MHz, CDCl$_3$) δ 6.94 (d, *J* = 1.8 Hz, 1H), 6.88 – 6.76 (m, 2H), 6.52 (s, 2H), 5.00 (d, *J* = 11.4 Hz, 1H), 4.41 (d, *J* = 10.7 Hz, 1H), 3.98 (dd, *J* = 11.0, 11.0 Hz, 1H), 3.89 (s, 3H), 3.85 (s, 9H), 3.78 (s, 3H), 3.36 (s, 3H), 3.30 (s, 3H), 1.08 ppm (s, 9H).
^{13}C NMR (101 MHz, CDCl$_3$) δ 170.31, 169.89, 169.64, 152.96, 148.78, 148.49, 137.37, 132.78, 129.98, 119.30, 111.32, 110.83, 105.80, 80.90, 62.43, 60.96, 56.23, 55.98, 52.68, 52.41, 47.52, 45.41, 43.26, 27.77 ppm.
HR-MS calculated for $C_{30}H_{39}O_{11}$ = 575.2487 $[M+H]^+$, found: 575.2487.
IR ν_{max} (cm^{-1}) 2349, 1726, 1589, 1516, 1462, 1424, 1367, 1345, 1247, 1156, 1124, 1060, 1027, 909, 846.

rac-Dimethyl (2R,3S,4R)-2-(3,4-dimethoxyphenyl)-3-((2-(methylthio)phenyl)carbamoyl)-4-(3,4,5-trimethoxyphenyl)cyclobutane-1,1-dicarboxylate (Compound **63**)

Cyclobutane **61** (0.03 mmol, 0.12 equiv.) was dissolved in DCM (1 mL). Trifluoroacetic acid (0.25ml) was added slowly to the solution at room temperature and stirred for 4 – 6h at that temperature. Solvents and acid were removed under vacuum and the residue was purified by column chromatography using petroleum ether/acetone (silica gel, 10:1 to 5:1 v:v). Carboxylic acid was collected and the solvent were evaporated. This acid was dissolved in DCM. Oxalyl chloride was added slowly to this solution at room temperature. Catalytic amount of DMF (0.1 eq.) was added into the reaction mixture and stirred for 1h. Solvent was removed under vacuum. To the dried reaction mixture, toluene, aniline **62** and molecular sieve were added sequentially. Reaction was put into a pre-heated oil bath at 80°C and stirred for 12h. The reaction was cooled to room temperature and was filtered through a filter paper to remove the molecular sieve. Filtrate was dried under reduced pressure. Compound **63** were purified by silica gel chromatography using petroleum ether and acetone (silica gel, 10:1 to 5:1 v:v).

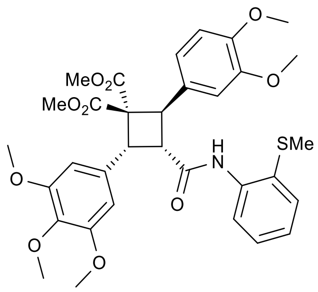

Isolated yield: 89% (yellow solid)
^{1}H NMR (500 MHz, CDCl$_3$) δ 8.27 – 8.06 (m, 2H), 7.37 (d, *J* = 7.3 Hz, 1H), 7.23 – 7.13 (m, 1H), 7.03 – 6.94 (m, 1H), 6.91 (dd, *J* = 8.3, 1.9 Hz, 1H), 6.83 (d, *J* = 8.4 Hz, 1H), 6.57 (s, 2H), 5.21 (d, *J* = 11.6 Hz, 1H), 4.60 (d, *J* = 10.4 Hz, 1H), 4.28 – 4.21 (m, 1H), 3.92 (s, 3H), 3.87 (s, 3H), 3.78 (s, 6H), 3.70 (s, 3H), 3.34 (s, 3H), 3.32 (s, 3H), 2.02 ppm (s, 3H).
^{13}C NMR (126 MHz, CDCl$_3$) δ 169.64, 169.18, 152.93, 148.77, 148.54, 137.32, 133.53, 131.51, 129.45, 129.18, 124.45, 124.22, 119.84, 119.27, 111.15, 110.76, 105.79, 62.42, 60.79, 56.13, 55.92, 55.90, 52.74, 52.32, 47.38, 45.11, 44.03, 19.02 ppm.
HR-MS calculated for $C_{33}H_{38}NO_{10}S$ = 640.2211 $[M+H]^+$, found: 640.2212.
IR ν_{max} (cm^{-1}) 2397, 2344, 2310, 2172, 2154, 2141, 2023, 2007, 1978, 1725, 1685, 1587, 1509, 1429, 1236, 1120, 1026.

rac-Methyl (1*S*,2*S*,3*S*,4*S*)-2-(3,4-dimethoxyphenyl)-3-((2-(methylthio)phenyl)carbamoyl)-4-(3,4,5-trimethoxyphenyl)cyclobutane-1-carboxylate (Compound **64**)

Compound **63** (13mg, 0.02mmol, 1 eq.) in DMSO (1 mL) was added lithium chloride (9 mg, 0.21 mmol, 10 eq.) and water (4ml, 0.021 mmol, 10 eq.). The reaction vial was purged with argon gas for 10 seconds before closing tightly with the cap. The reaction vial was put into the pre-heated metal block at 130°C for 6h with vigorous stirring. After 6h, the reaction vial was cooled down to the room temperature. Reaction mixture was added 2ml of water and was extracted with 3 ml of ethyl acetate (3 ml x 3 times). The combined organic phase was dried by sodium sulfate and the solvent was evaporated to give a reaction mixture as yellow, crude oil. This mixture was subjected to flash chromatography (silica gel, petroleum ether: acetone from 10:1 to 3:2 v:v) and gave compound **64** in 65% yield.

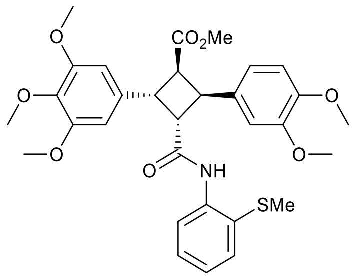

Isolated yield: 65% (yellow foam)

^{1}H NMR (700 MHz, CD_2Cl_2) δ 8.17 – 8.03 (m, 1H), 7.37 (dd, *J* = 7.7, 1.5 Hz, 1H), 7.19 (d, *J* = 1.3 Hz, 1H), 7.00 (t, *J* = 7.5 Hz, 1H), 6.97 – 6.93 (m, 1H), 7.02 – 6.70 (m, 2H), 6.58 (s, 2H), 4.53 (dd, *J* = 10.8, 6.3 Hz, 1H), 4.42 (t, *J* = 9.1, 9.1 Hz, 1H), 3.94 (dd, *J* = 10.9, 7.6 Hz, 1H), 3.91 – 3.88 (m, 1H), 3.87 (s, 3H), 3.83 (s, 3H), 3.75 (s, 6H), 3.59 (s, 3H), 3.38 (s, 3H), 2.15 ppm (s, 3H).

^{13}C NMR (176 MHz, CD_2Cl_2) δ 172.89, 169.93 (d, *J* = 15.0 Hz), 153.84, 149.60, 148.98, 137.89, 134.89, 133.38, 132.19, 129.09, 125.75, 125.65, 124.70, 120.47, 120.39, 119.93, 112.22, 111.84, 105.56, 60.85, 56.57, 56.46, 56.32, 52.00, 50.12, 47.95, 43.19, 41.57 (d, *J* = 2.7 Hz), 19.20 ppm.

HR-MS calculated for $C_{31}H_{35}NO_8S$ = 582.2156 $[M+H]^+$, found: 582.2170.

IR ν_{max} (cm^{-1}) 1729, 1681, 1587, 1511, 1462, 1334, 1250, 1124, 1026.

Observation of olefinic side products using general procedure C

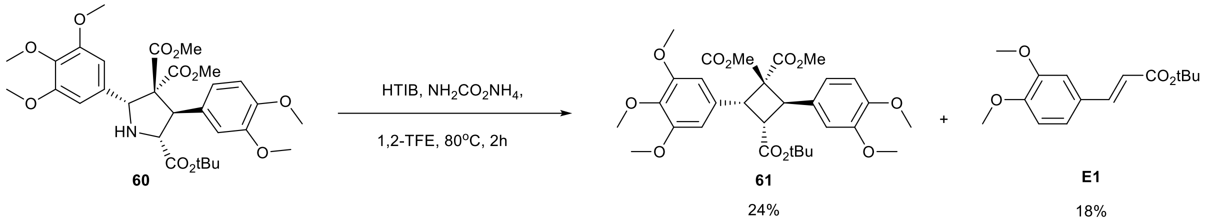

Scheme S6. Olefinic side product observed when standard condition using HTIB was applied.

tert-Butyl (*E*)-3-(3,4-dimethoxyphenyl)acrylate (Compound **E1**)

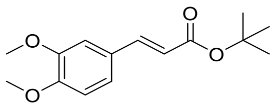

Using general procedure C

Isolated yield: 18% (colorless oil)

(silica gel, 1:100 acetone:petroleum ether to 1:50 = acetone:petroleum ether)

^{1}H NMR (500 MHz, $CDCl_3$) δ 7.53 (d, *J* = 15.9 Hz, 1H), 7.16 – 6.99 (m, 2H), 6.85 (d, *J* = 8.3 Hz, 1H), 6.24 (d, *J* = 15.9 Hz, 1H), 3.91 (s, 6H), 1.53 ppm (s, 9H).

^{13}C NMR (126 MHz, $CDCl_3$) δ 166.61, 150.82, 149.13, 143.50, 127.64, 122.47, 117.90, 110.95, 109.38, 80.35, 55.96, 55.87, 28.25 ppm.

HR-MS calculated for $C_{15}H_{21}O_4$ = 265.1434 $[M+H]^+$, found: 265.1429.

IR ν_{max} (cm^{-1}) 1702, 1632, 1599, 1513, 1465, 1421, 1367, 1308, 1264, 1138, 1025, 980, 912, 847.

References

1. Antonchick, A. P.; Gerding-Reimers, C.; Catarinella, M.; Schürmann, M.; Preut, H.; Ziegler, S.; Rauh, D.; Waldmann, H., Highly enantioselective synthesis and cellular evaluation of spirooxindoles inspired by natural products. *Nat Chem* **2010,** *2* (9), 735-740. https://doi.org/10.1038/nchem.730
2. Zhou, J. F.; Xu, J. M.; Wei, H.; Zhang, L. L., Solid phase synthesis of alpha, alpha '-bis(substituted benzylidene) cycloalkanones. *Chinese Journal of Organic Chemistry* **2001,** *21* (4), 322-324.
3. Mu, Y.; Nguyen, T. T.; Koh, M. J.; Schrock, R. R.; Hoveyda, A. H., E- and Z-, di- and tri-substituted alkenyl nitriles through catalytic cross-metathesis. *Nature Chemistry* **2019,** *11* (5), 478-487. https://doi.org/10.1038/s41557-019-0233-x
4. Fraenkel, G.; Duncan, J. H.; Wang, J., Restricted Stereochemistry of Solvation of Allylic Lithium Compounds: Structural and Dynamic Consequences. *Journal of the American Chemical Society* **1999,** *121* (2), 432-443. https://doi.org/10.1021/ja983047h
5. Hansen, A. H.; Sergeev, E.; Bolognini, D.; Sprenger, R. R.; Ekberg, J. H.; Ejsing, C. S.; McKenzie, C. J.; Rexen Ulven, E.; Milligan, G.; Ulven, T., Discovery of a Potent Thiazolidine Free Fatty Acid Receptor 2 Agonist with Favorable Pharmacokinetic Properties. *Journal of Medicinal Chemistry* **2018,** *61* (21), 9534-9550. https://doi.org/10.1021/acs.jmedchem.8b00855
6. Wu, Q.-F.; Wang, X.-B.; Shen, P.-X.; Yu, J.-Q., Enantioselective C–H Arylation and Vinylation of Cyclobutyl Carboxylic Amides. *ACS Catalysis* **2018,** *8* (3), 2577-2581. https://doi.org/10.1021/acscatal.8b00069
7. Nguyen, K.; Clement, H. A.; Bernier, L.; Coe, J. W.; Farrell, W.; Helal, C. J.; Reese, M. R.; Sach, N. W.; Lee, J. C.; Hall, D. G., Catalytic Enantioselective Synthesis of a cis-β-Boronyl Cyclobutylcarboxyester Scaffold and Its Highly Diastereoselective Nickel/Photoredox Dual-Catalyzed Csp3–Csp2 Cross-Coupling to Access Elusive trans-β-Aryl/Heteroaryl Cyclobutylcarboxyesters. *ACS Catalysis* **2021,** *11* (1), 404-413. https://doi.org/10.1021/acscatal.0c04520
8. Dolomanov, O. V.; Bourhis, L. J.; Gildea, R. J.; Howard, J. A. K.; Puschmann, H., OLEX2: a complete structure solution, refinement and analysis program. *Journal of Applied Crystallography* **2009,** *42* (2), 339-341. https://doi.org/doi:10.1107/S0021889808042726
9. Sheldrick, G., SHELXT - Integrated space-group and crystal-structure determination. *Acta Crystallographica Section A* **2015,** *71* (1), 3-8. https://doi.org/doi:10.1107/S2053273314026370
10. Sheldrick, G., Crystal structure refinement with SHELXL. *Acta Crystallographica Section C* **2015,** *71* (1), 3-8. https://doi.org/doi:10.1107/S2053229614024218
11. Farrugia, L., ORTEP-3 for Windows - a version of ORTEP-III with a Graphical User Interface (GUI). *Journal of Applied Crystallography* **1997,** *30* (5 Part 1), 565. https://doi.org/doi:10.1107/S0021889897003117
12. Gutekunst, W. R.; Baran, P. S., Total Synthesis and Structural Revision of the Piperarborenines via Sequential Cyclobutane C–H Arylation. *J Am Chem Soc* **2011,** *133* (47), 19076-19079. https://doi.org/10.1021/ja209205x
13. Panish, R. A.; Chintala, S. R.; Fox, J. M., A Mixed-Ligand Chiral Rhodium(II) Catalyst Enables the Enantioselective Total Synthesis of Piperarborenine B. *Angewandte Chemie International Edition* **2016,** *55* (16), 4983-4987. https://doi.org/https://doi.org/10.1002/anie.201600766
14. Hu, J.-L.; Feng, L.-W.; Wang, L.; Xie, Z.; Tang, Y.; Li, X., Enantioselective Construction of Cyclobutanes: A New and Concise Approach to the Total Synthesis of (+)-Piperarborenine B. *Journal of the American Chemical Society* **2016,** *138* (40), 13151-13154. https://doi.org/10.1021/jacs.6b08279
15. Xue, Z.-Y.; Liu, T.-L.; Lu, Z.; Huang, H.; Tao, H.-Y.; Wang, C.-J., exo-Selective asymmetric 1,3-dipolar cycloaddition of azomethine ylides with alkylidene malonates catalyzed by AgOAc/TF-BiphamPhos. *Chemical Communications* **2010,** *46* (10), 1727-1729. https://doi.org/10.1039/B919625K
16. Sanders, S. D.; Ruiz-Olalla, A.; Johnson, J. S., Total synthesis of (+)-virgatusinviaAlCl3-catalyzed [3+2] cycloaddition. *Chemical Communications* **2009,** (34), 5135-5137. https://doi.org/10.1039/B911765B

Spectra

NMR spectra of cyclobutanes

^{1}H NMR of compound 5

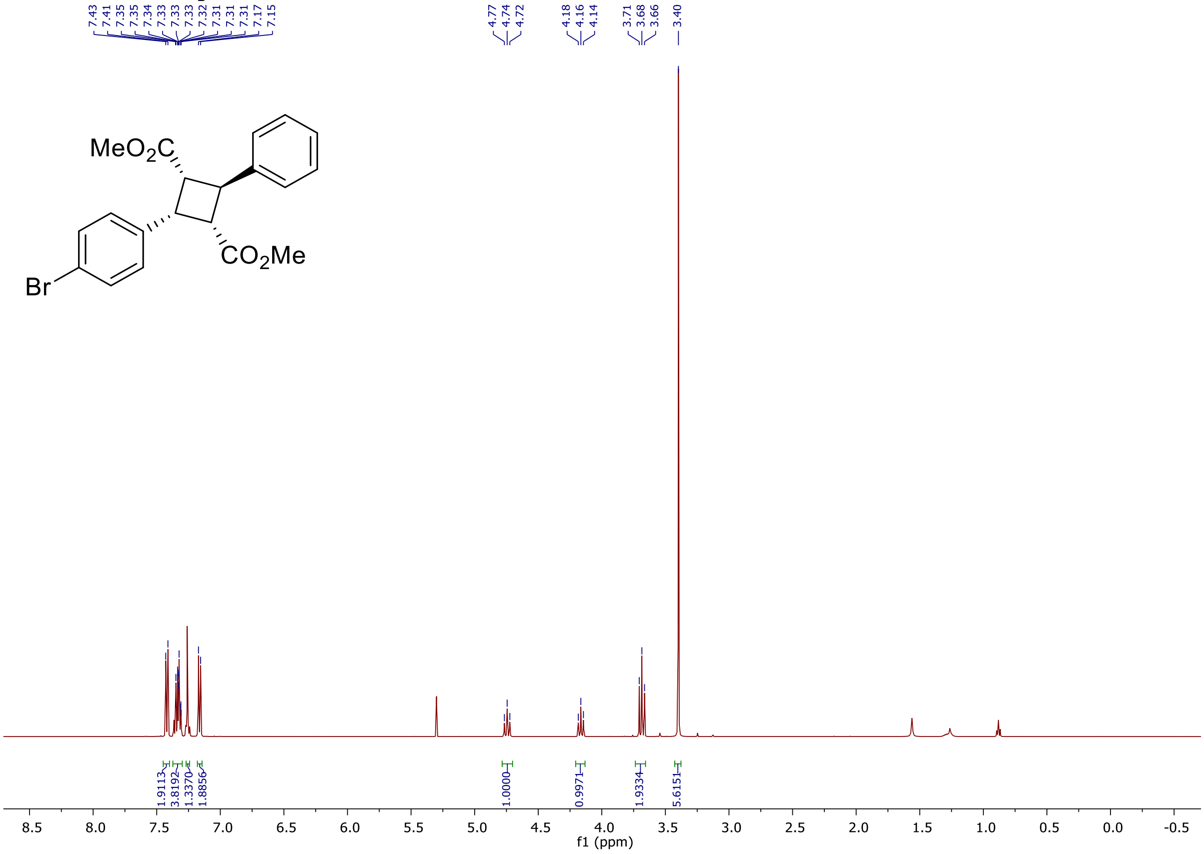

^{13}C NMR of compound 5

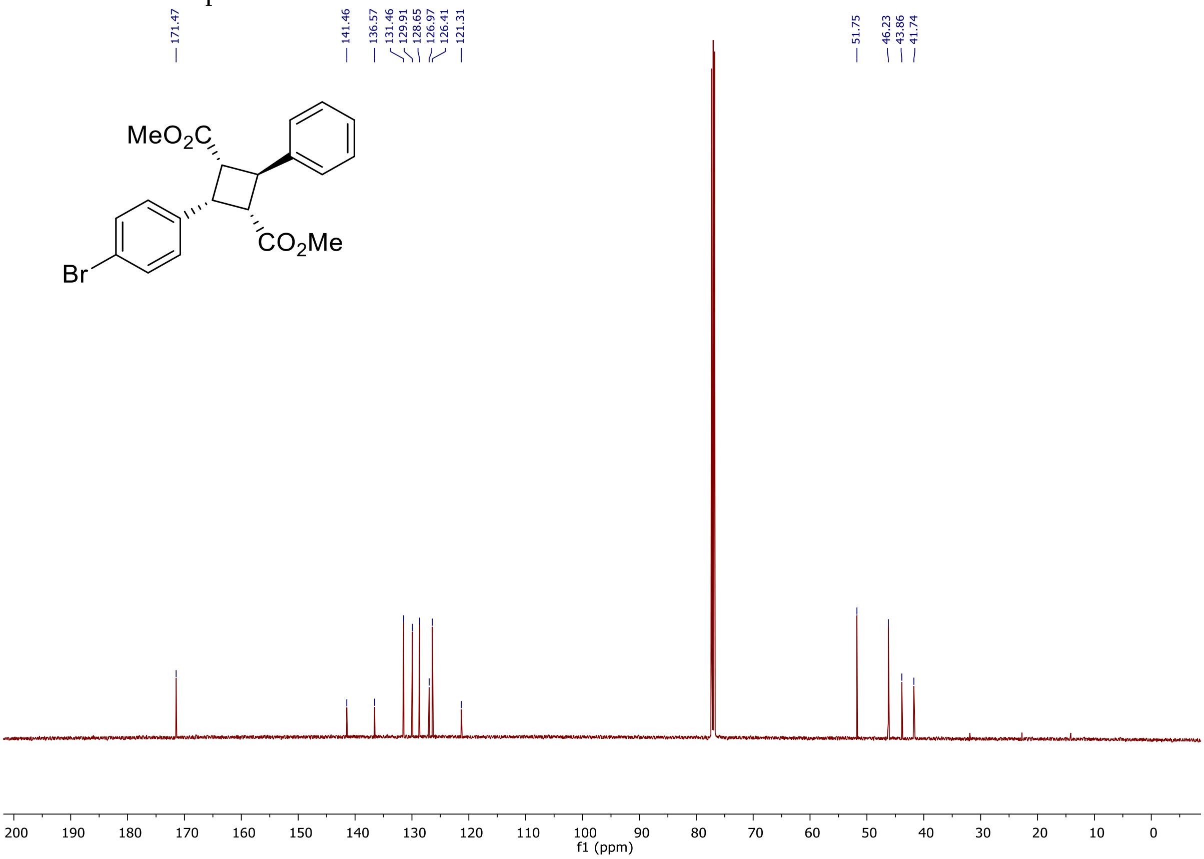

^{1}H NMR of compound 8

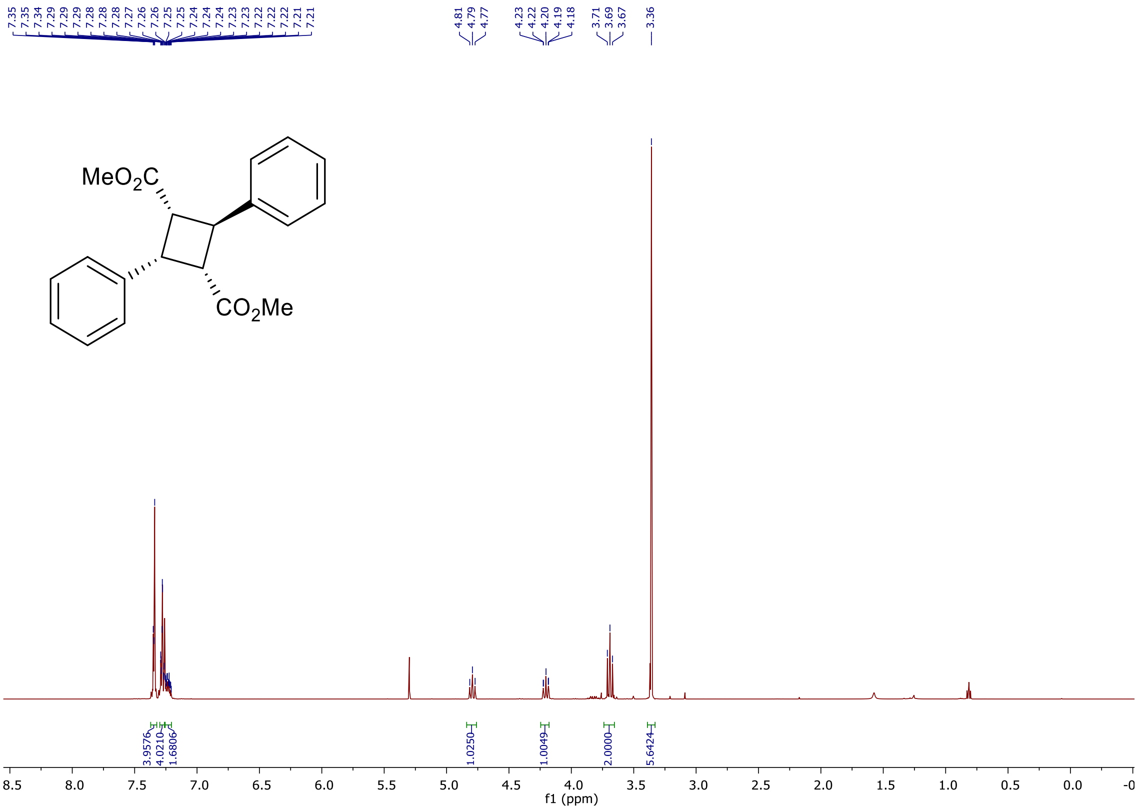

^{13}C NMR of compound 8

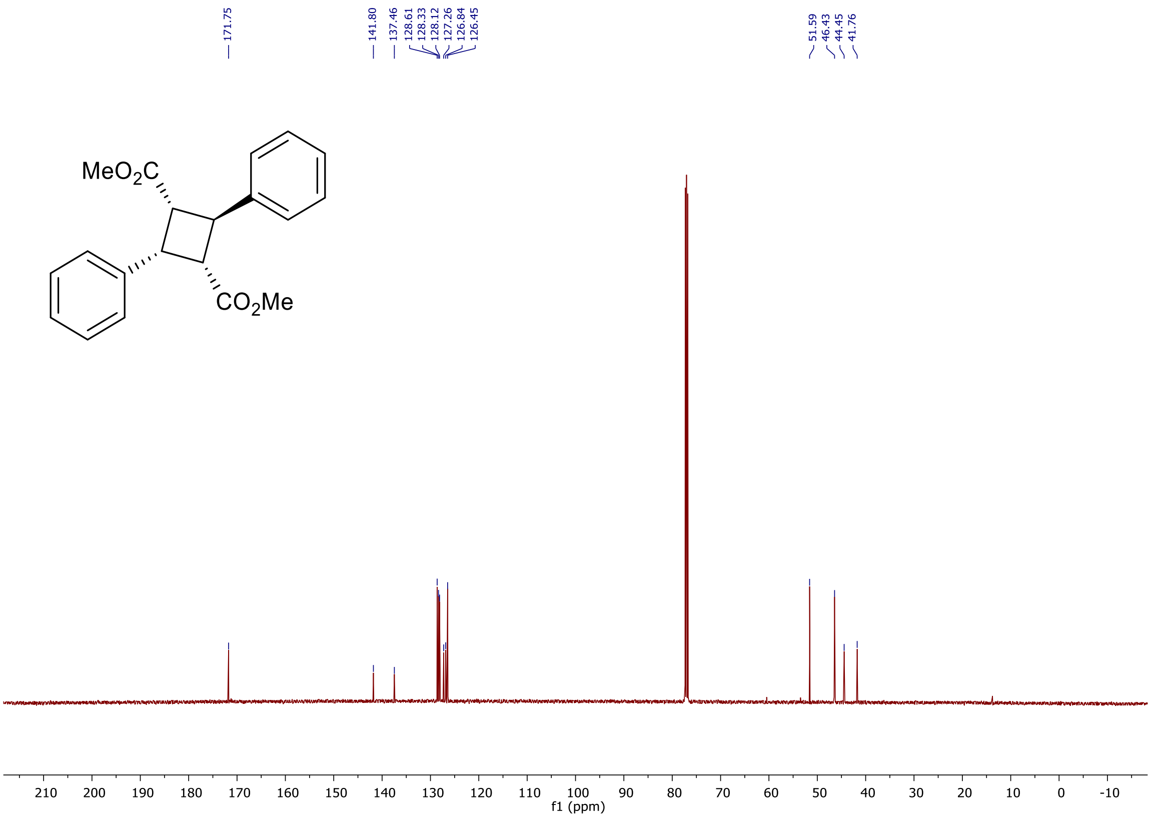

^{1}H NMR of compound 9

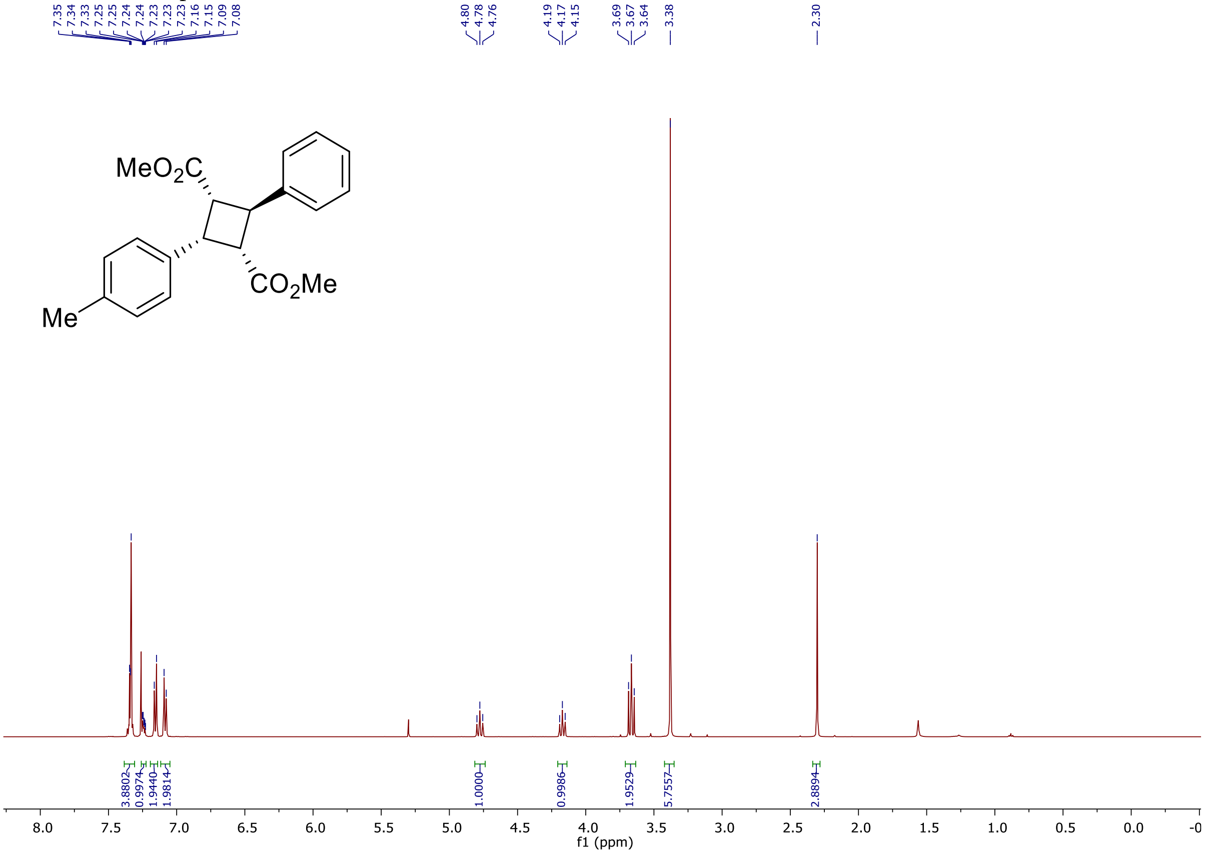

^{13}C NMR of compound 9

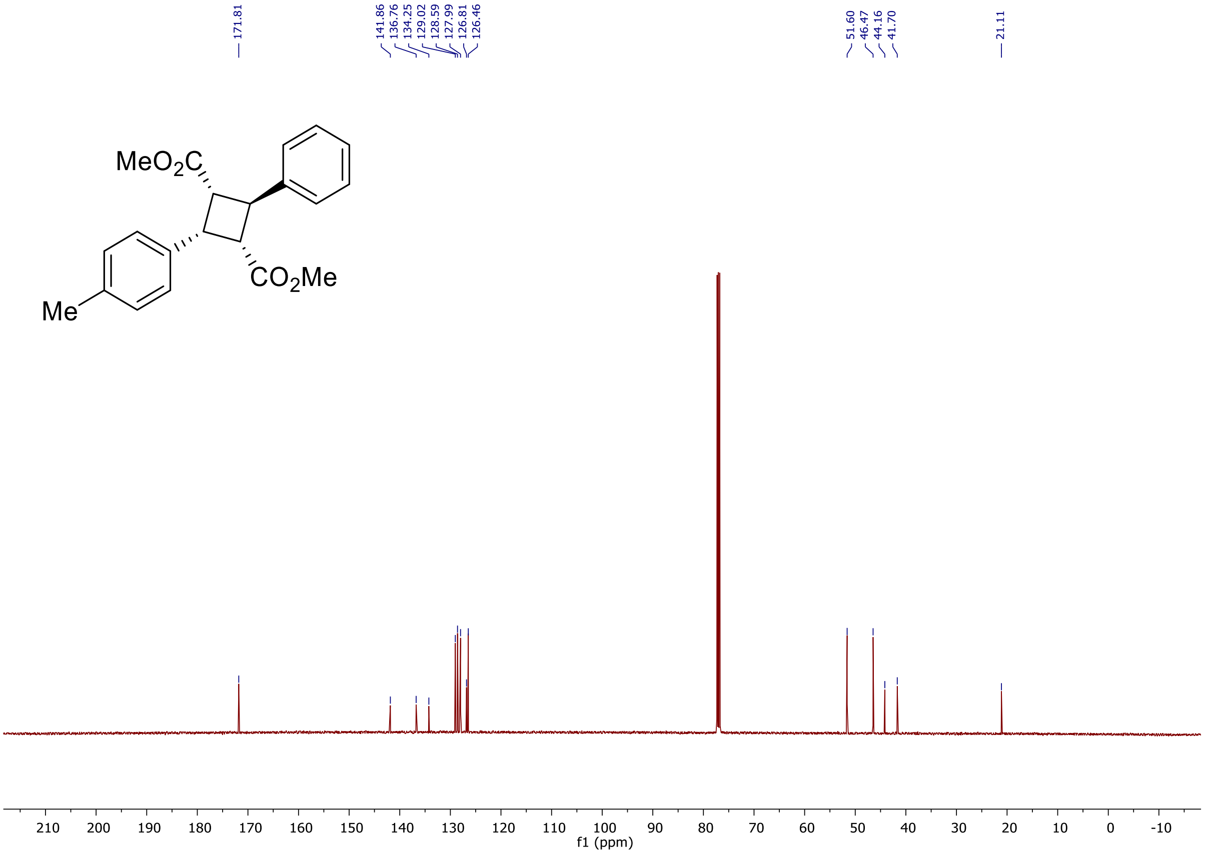

^{1}H NMR of compound 10

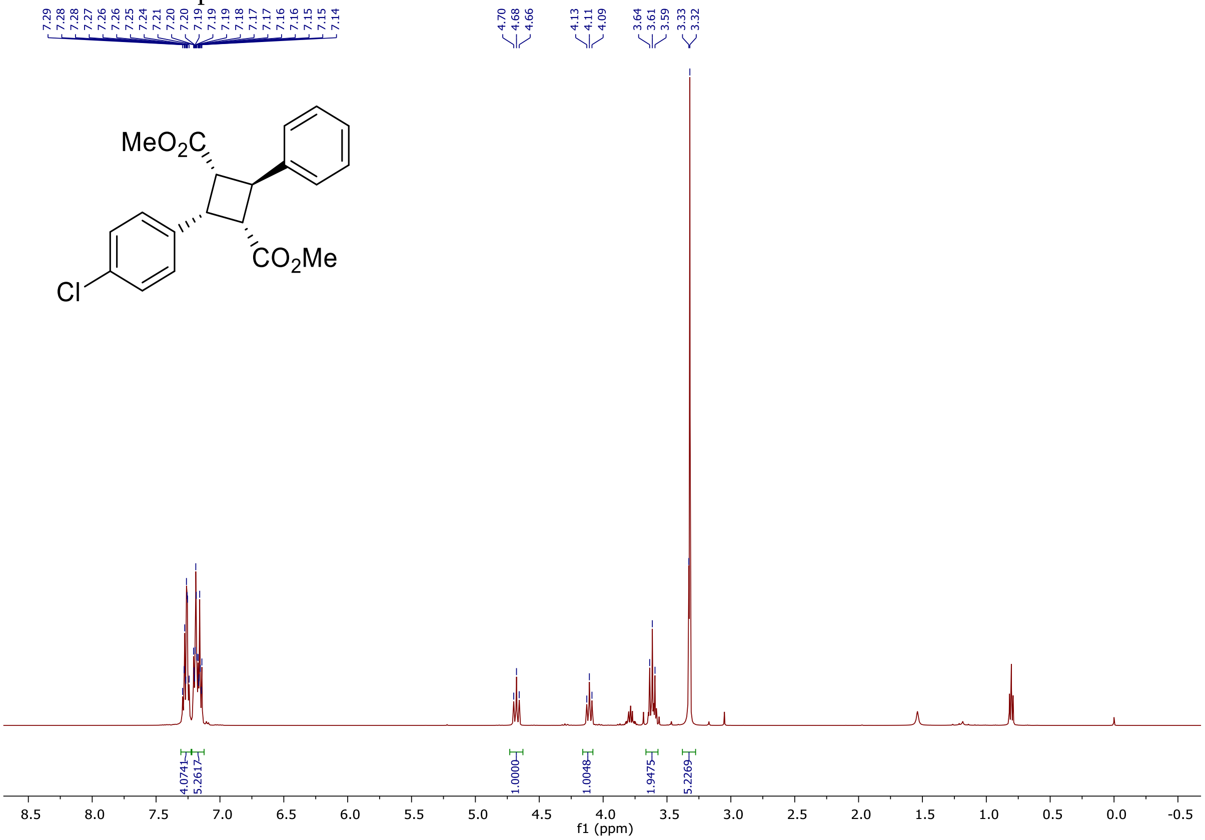

^{13}C NMR of compound 10

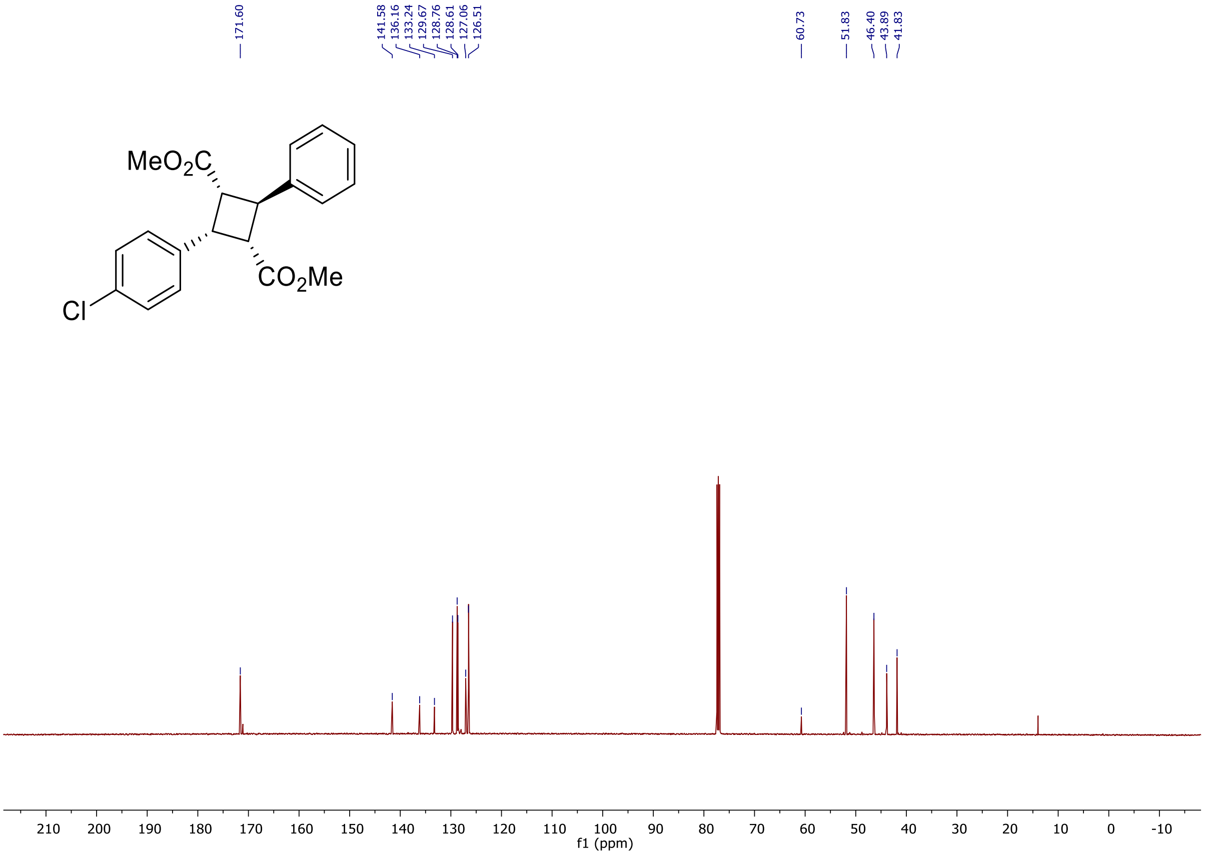

^{1}H NMR of compound 11

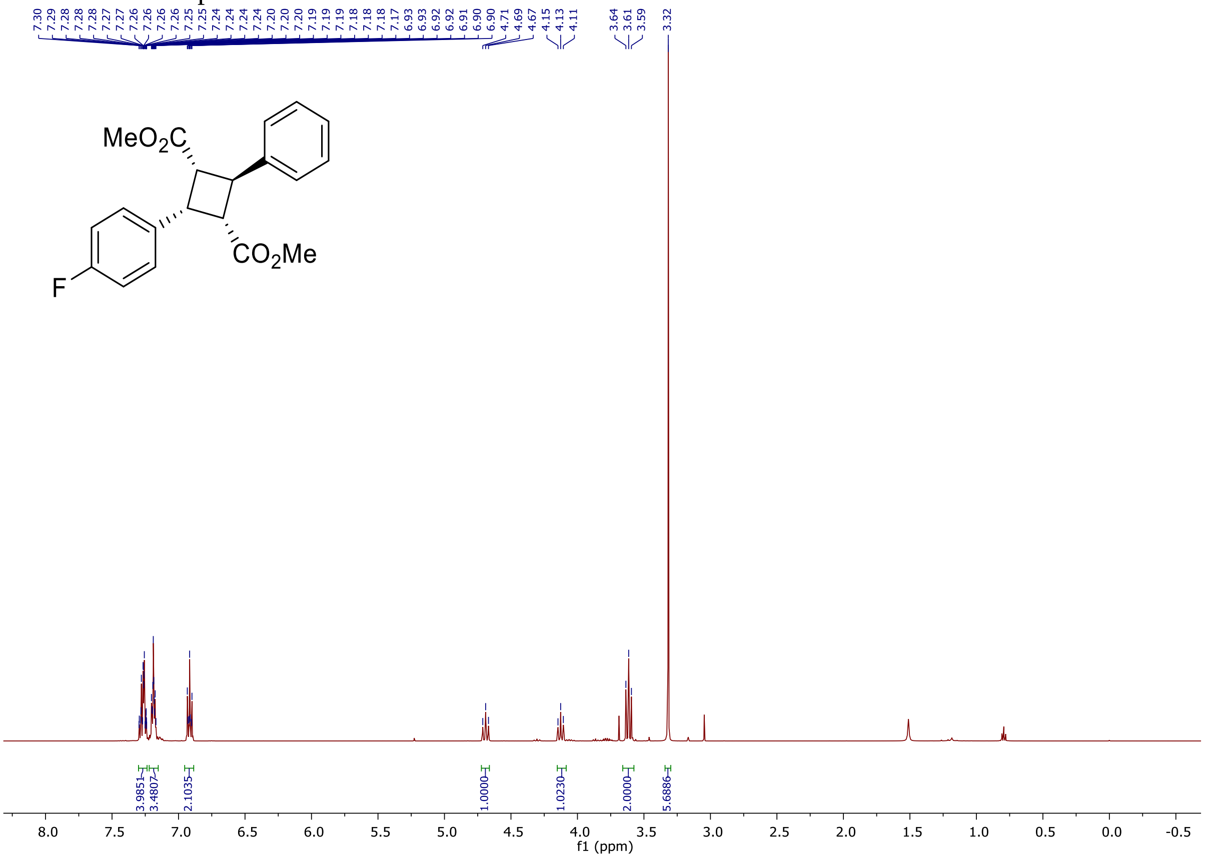

^{13}C NMR of compound 11

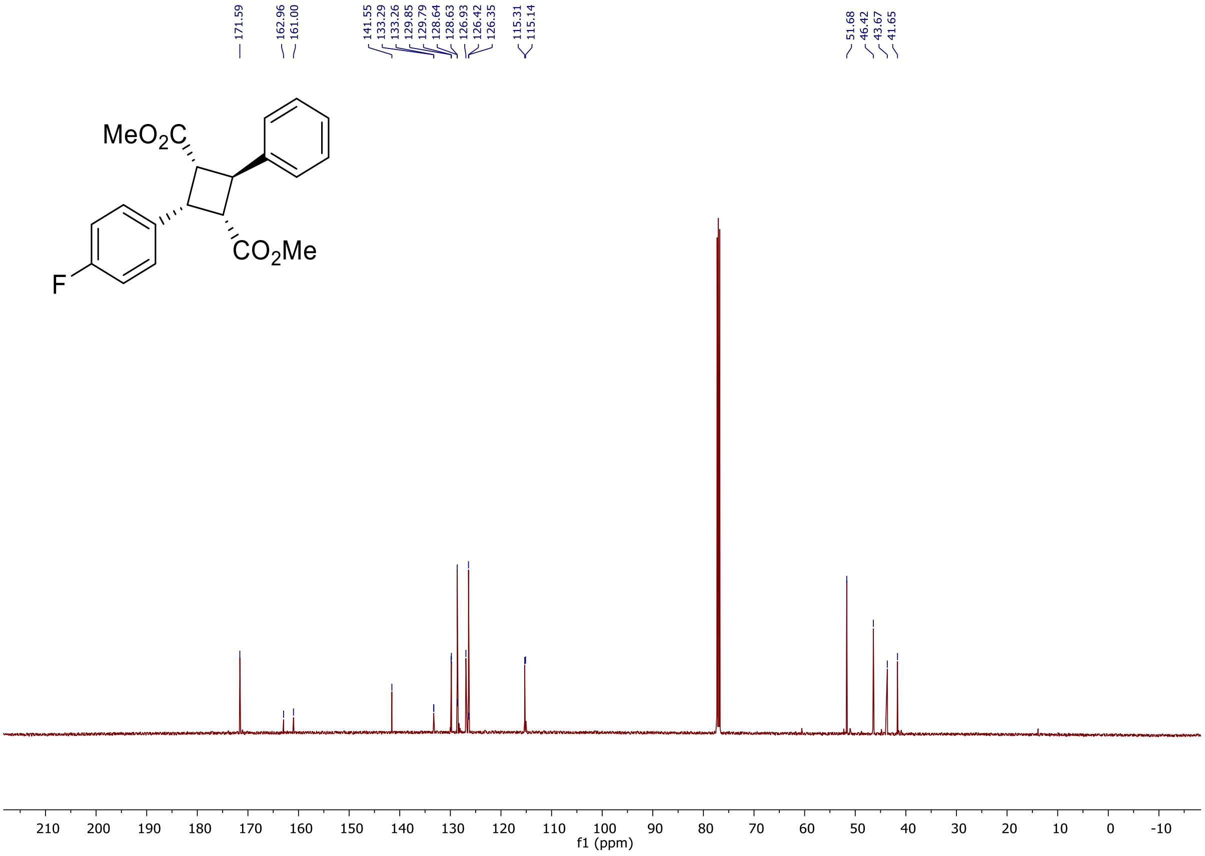

^{19}F NMR of compound 11

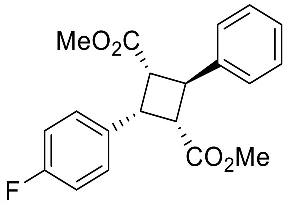

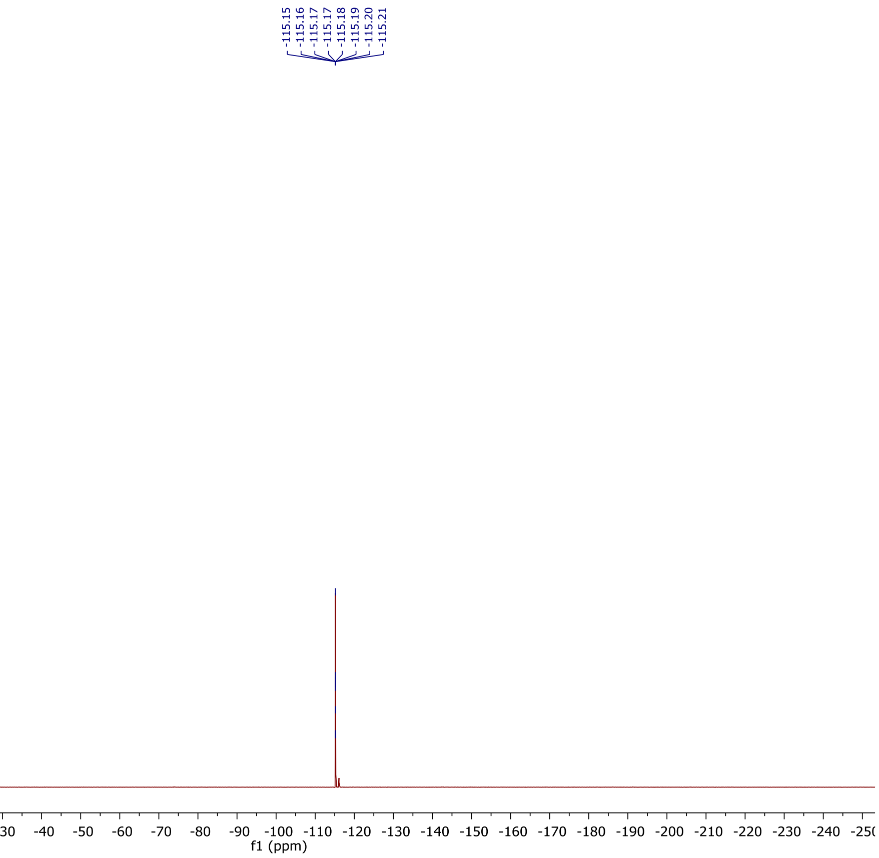

^{1}H NMR of Compound 12

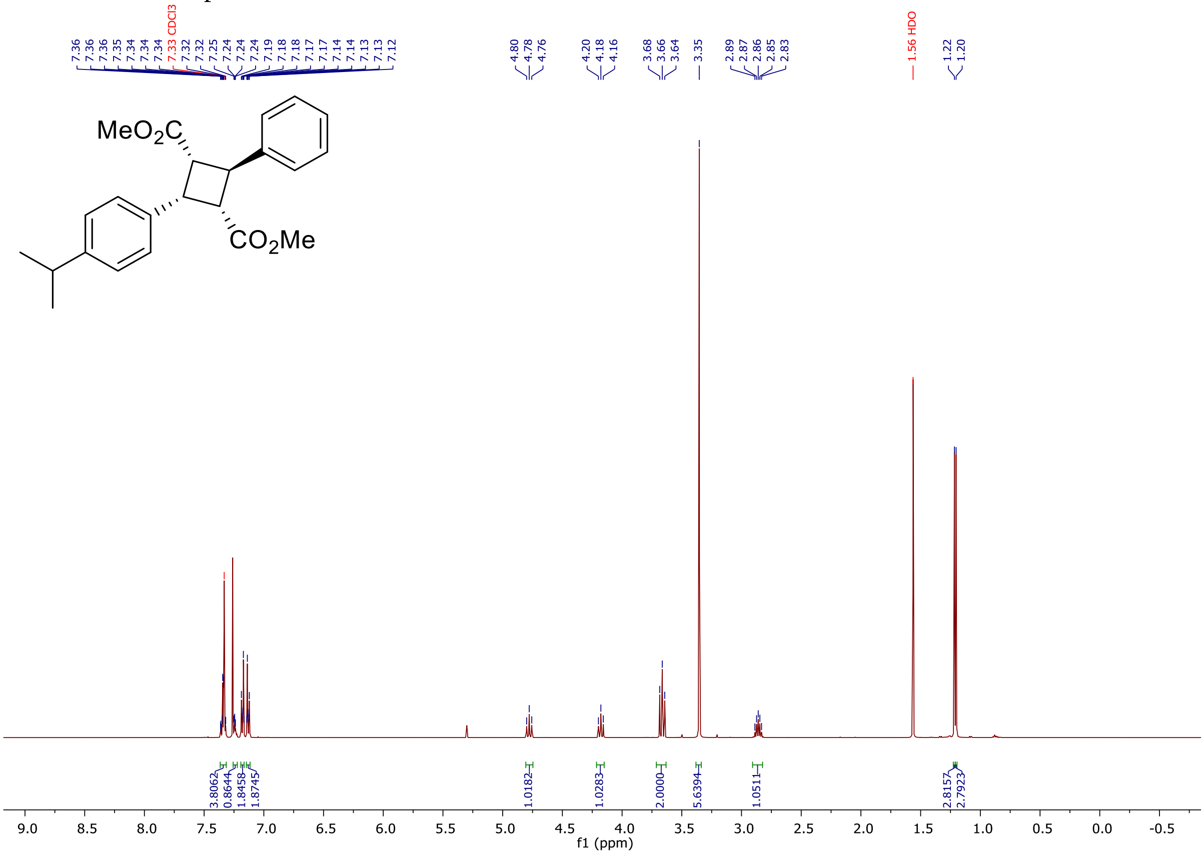

^{13}C NMR of compound 12

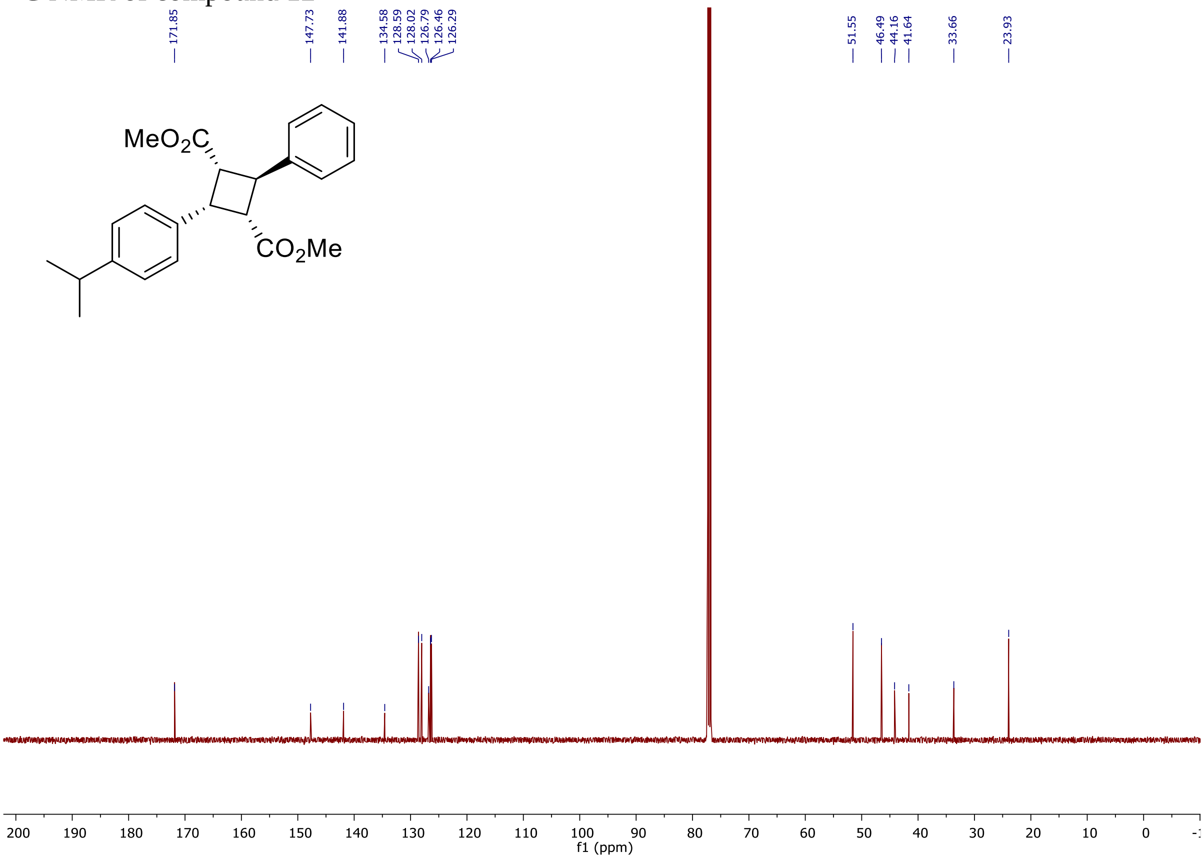

^{1}H NMR of compound 13

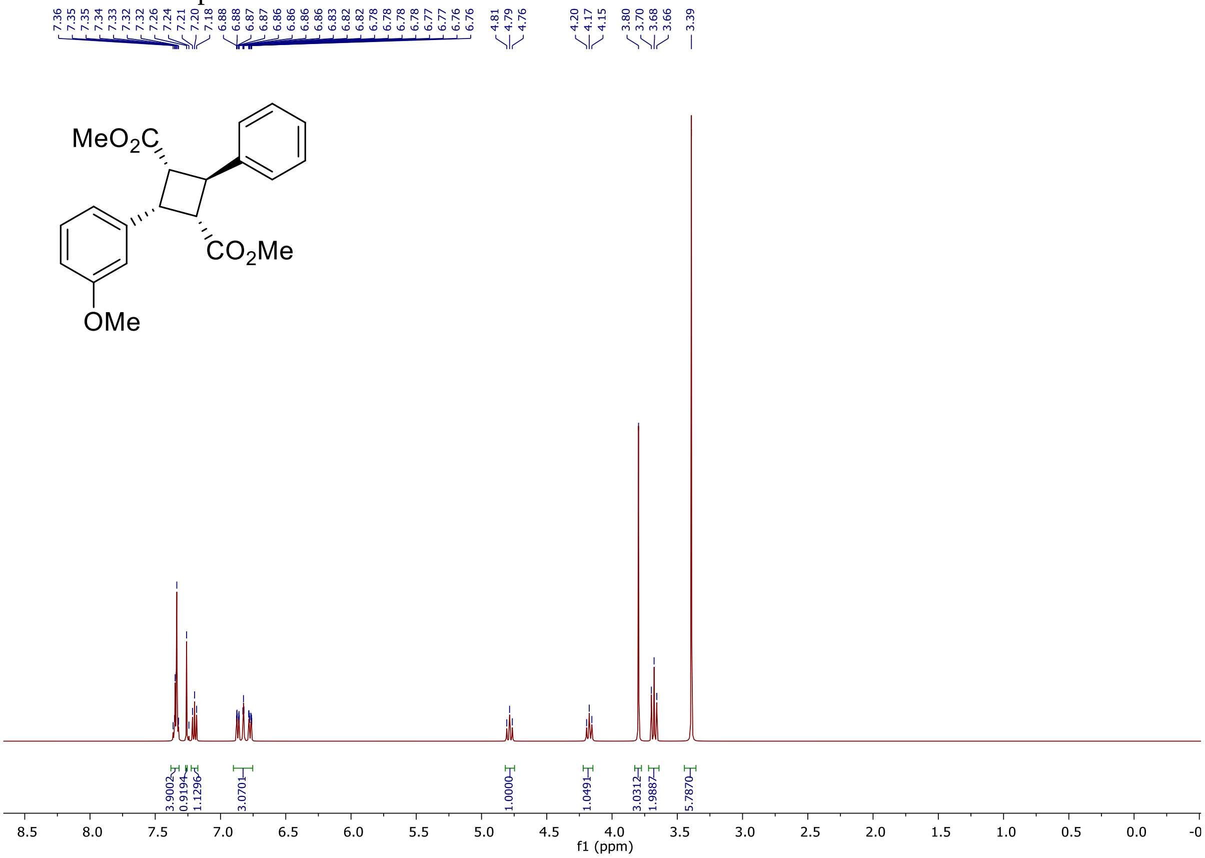

^{13}C NMR of compound 13

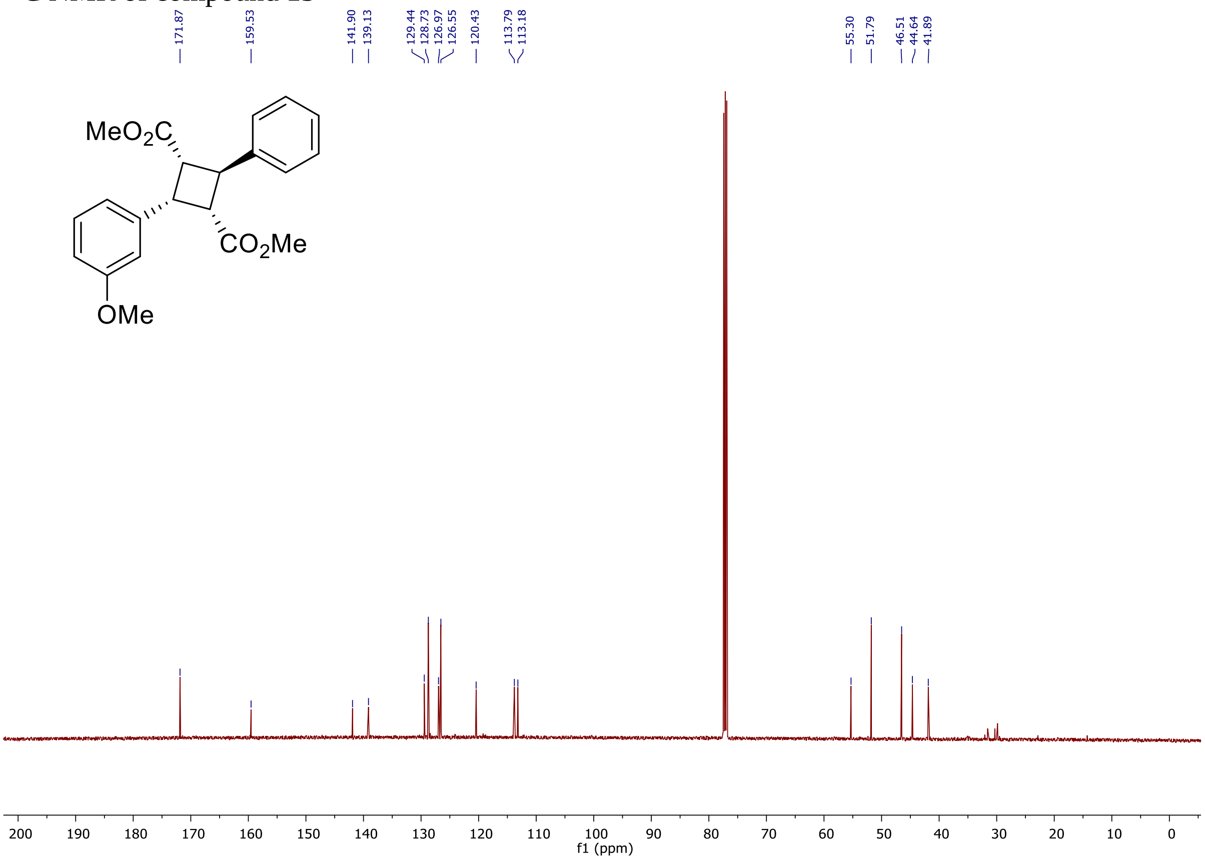

^{1}H NMR of Compound 14

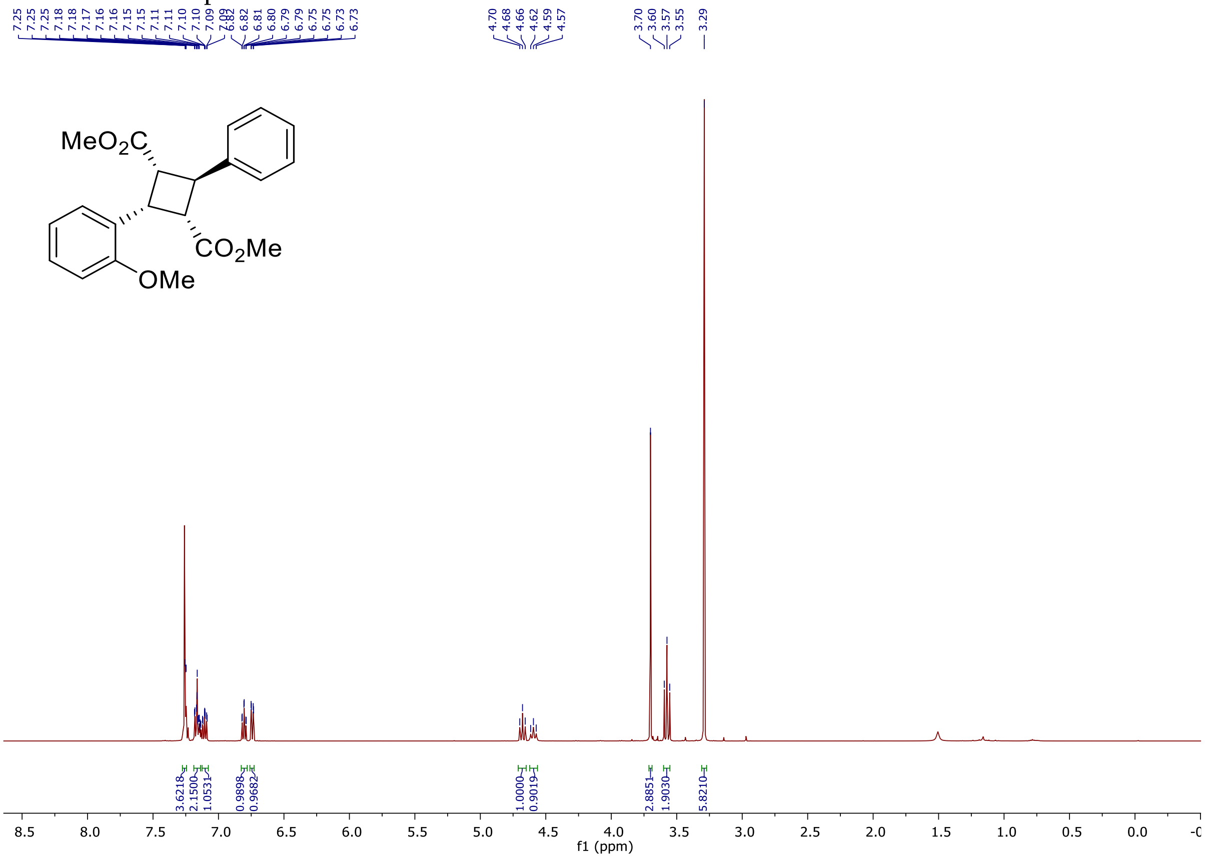

^{13}C NMR of compound 14

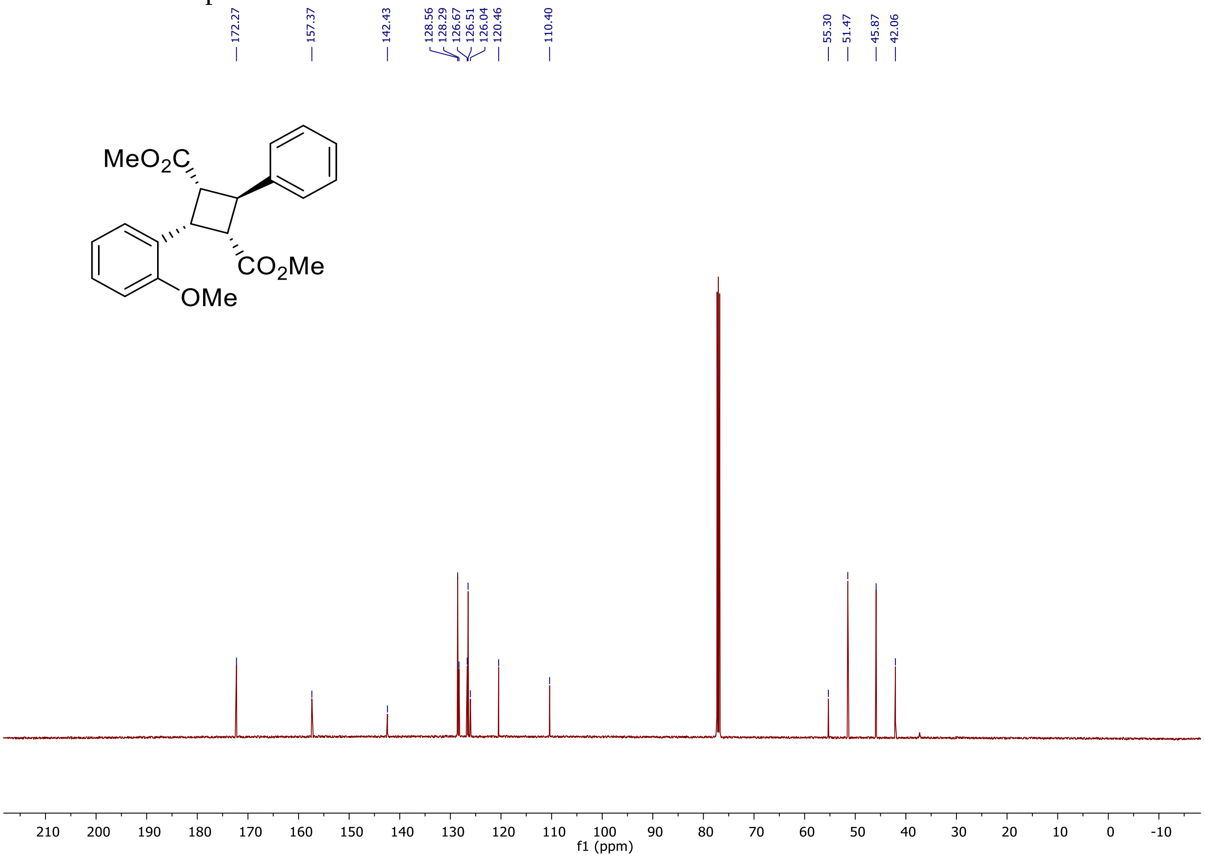

^{1}H NMR of compound 15

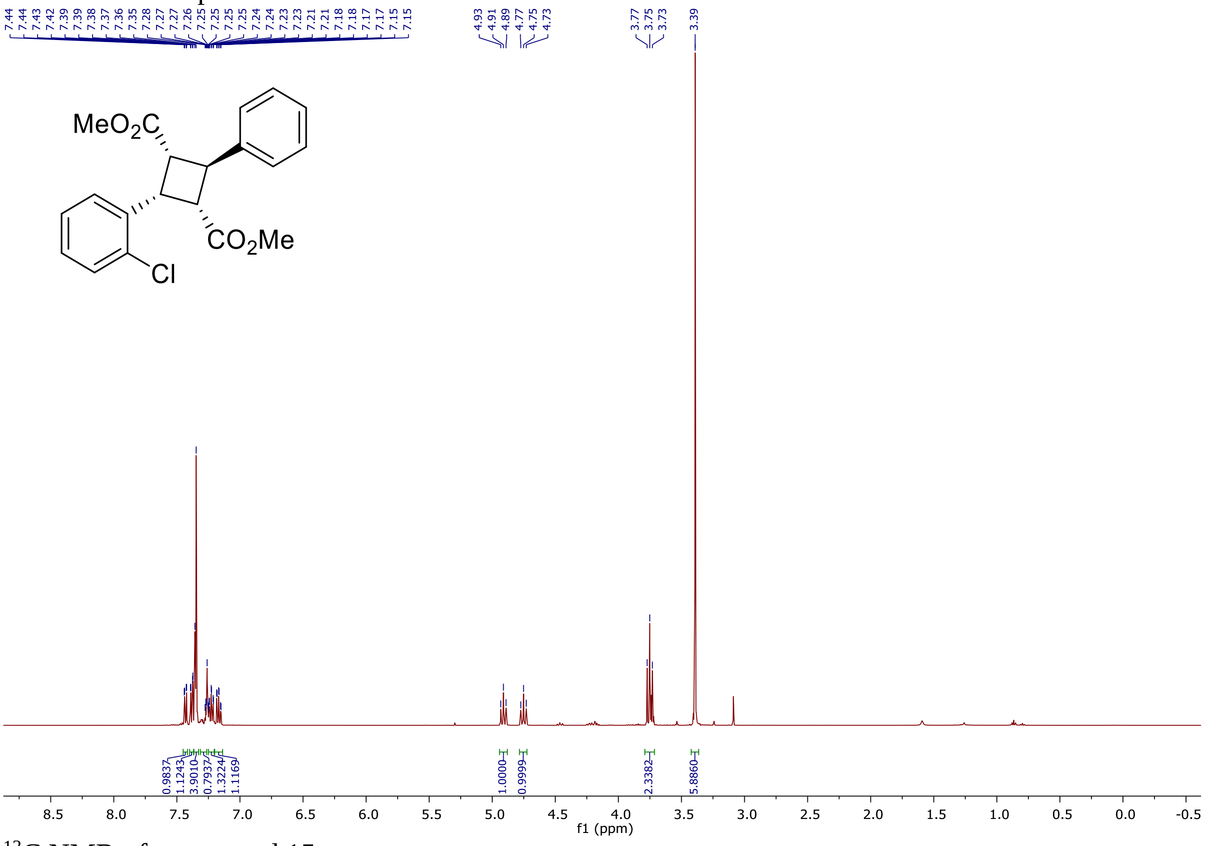

^{13}C NMR of compound 15

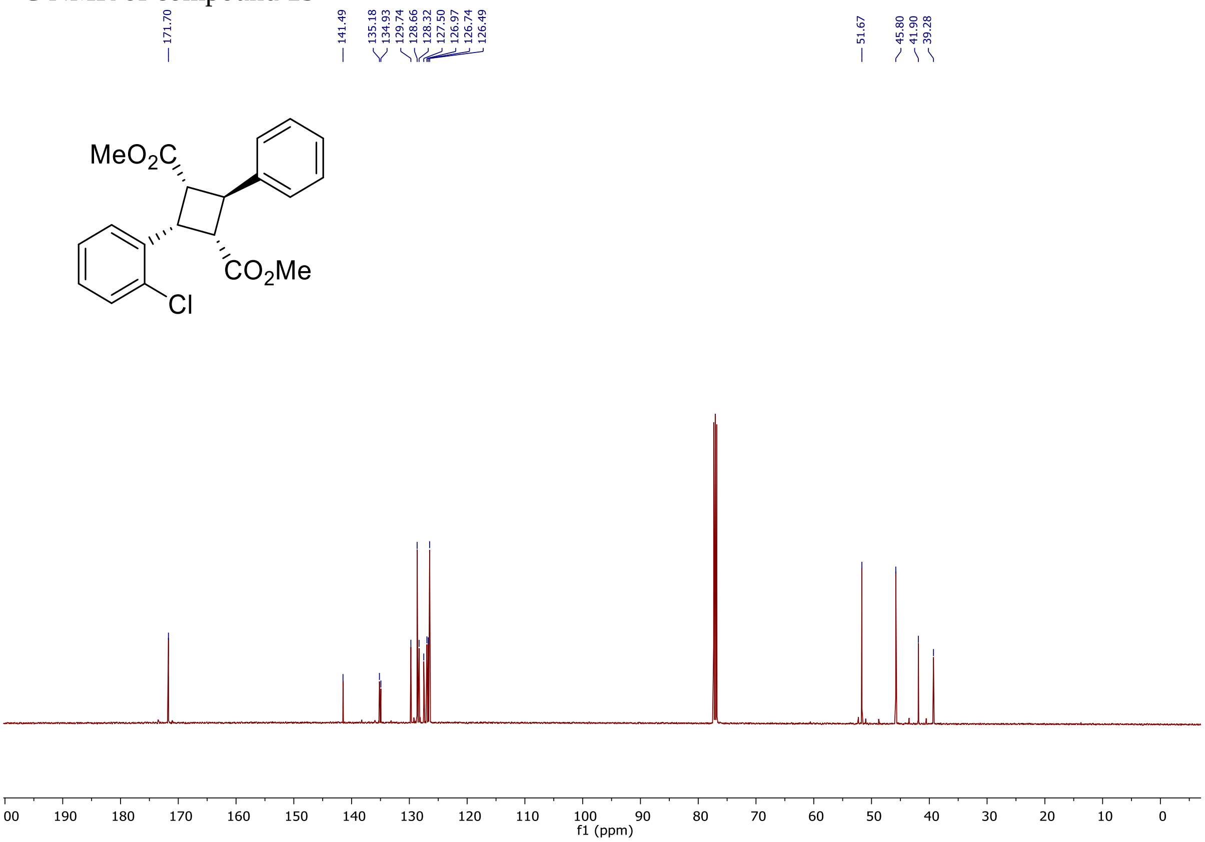

^{1}H NMR of compound 16

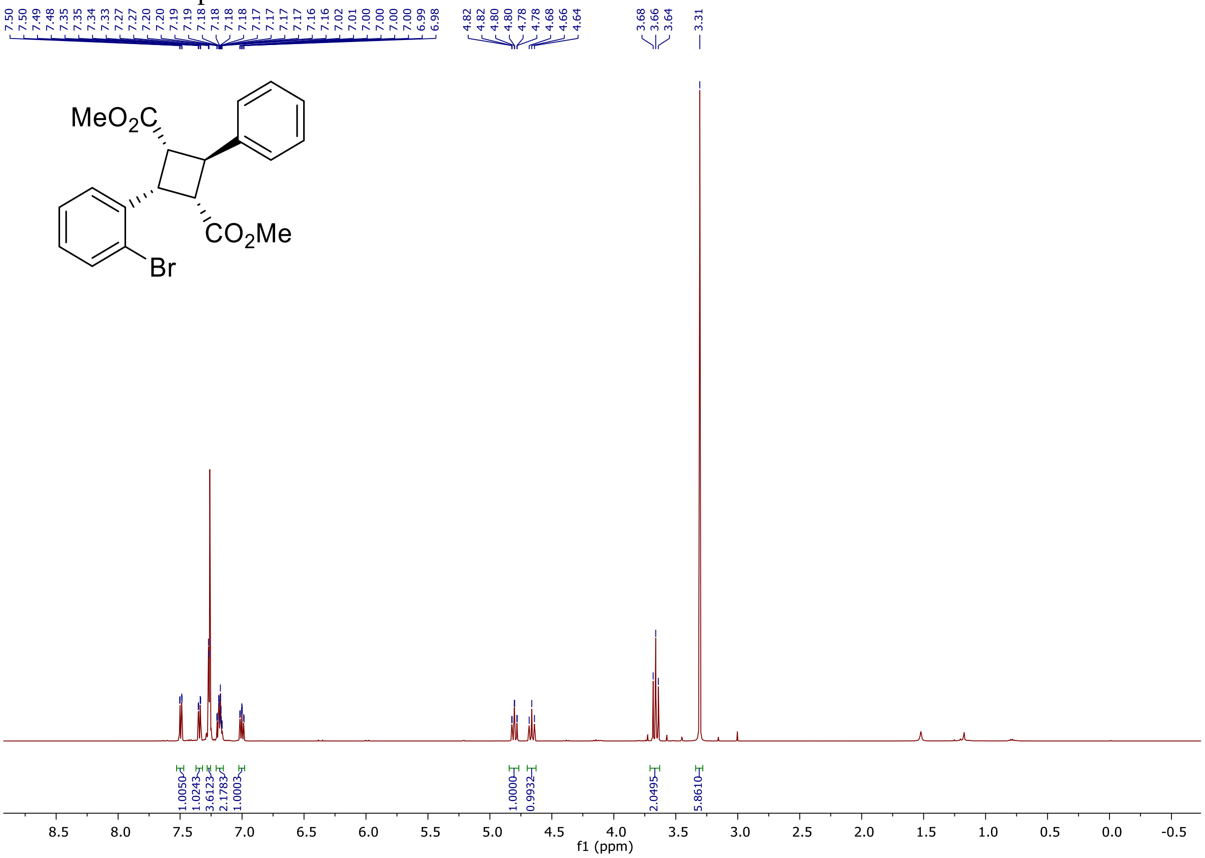

^{13}C NMR of compound 16

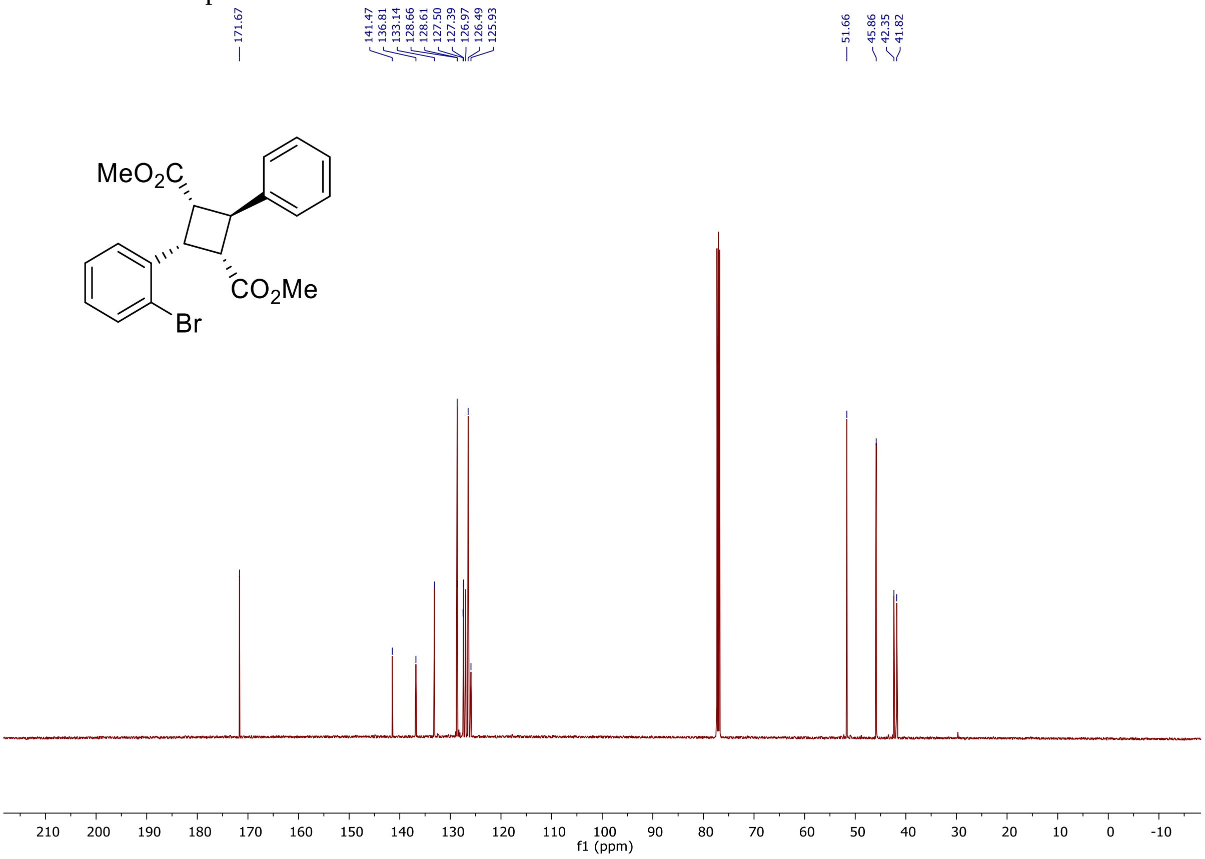

^{1}H NMR of compound 17

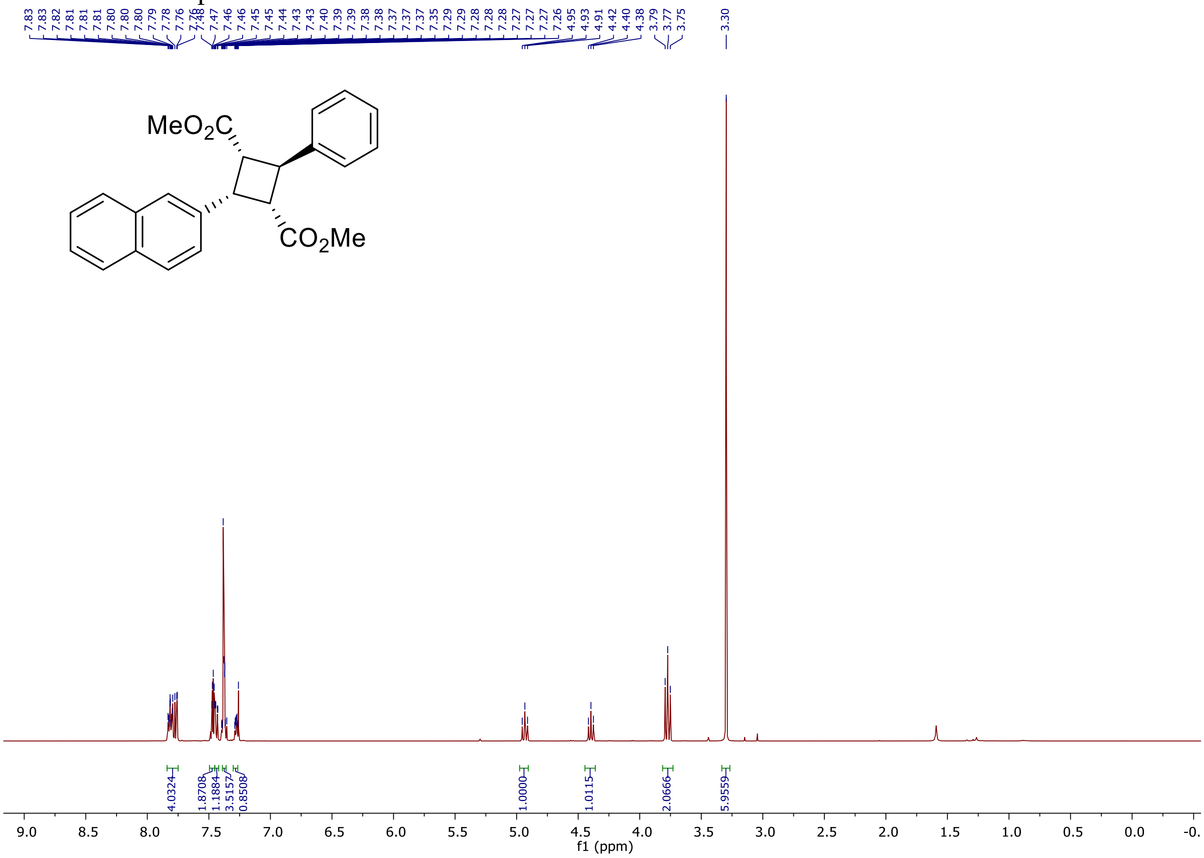

^{13}C NMR of compound 17

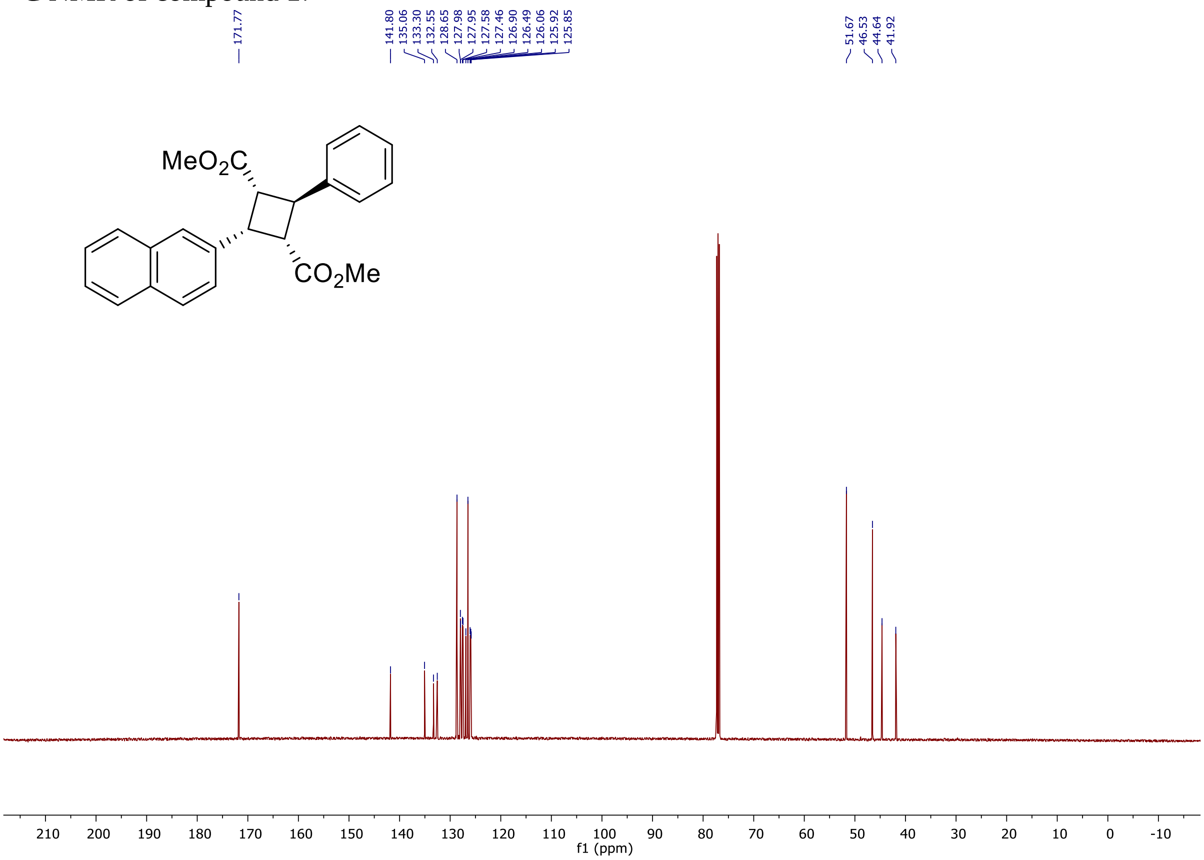

^{1}H NMR of compound 18

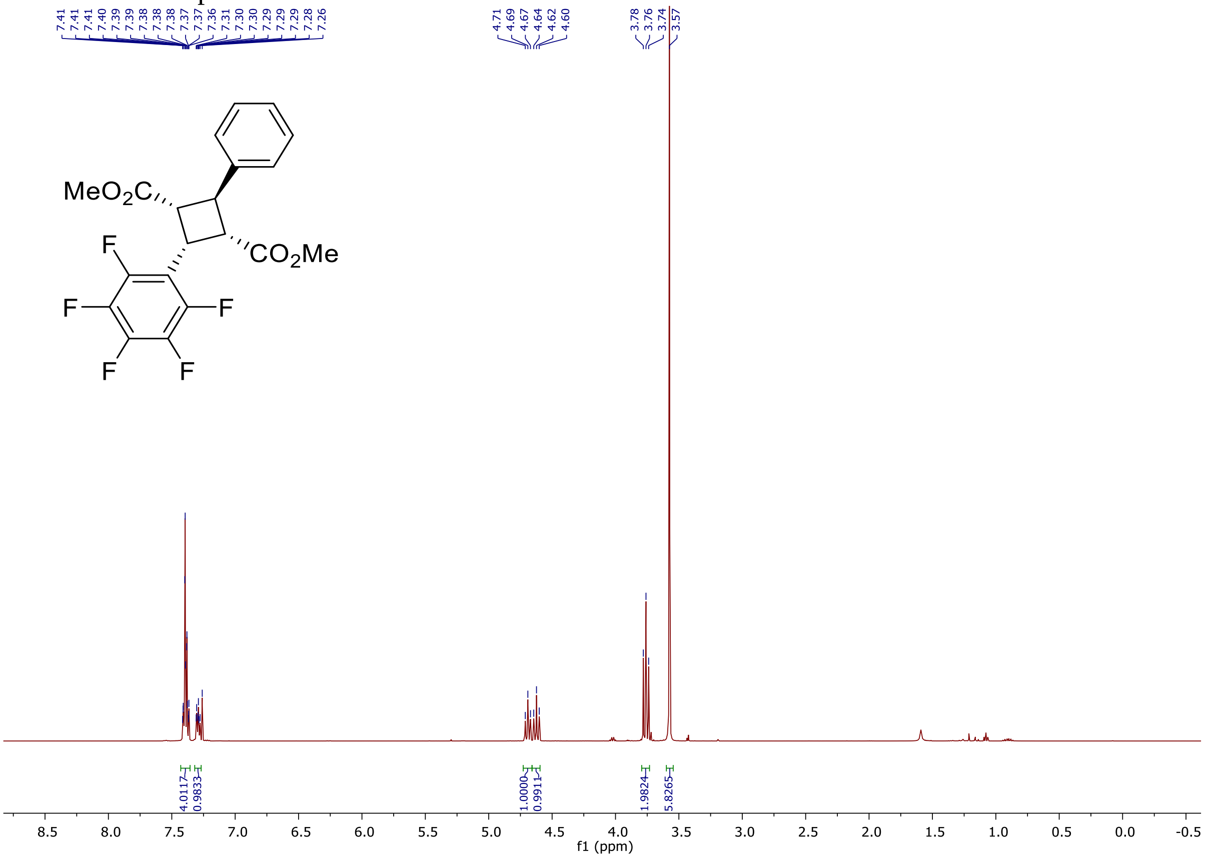

^{13}C NMR of compound 18

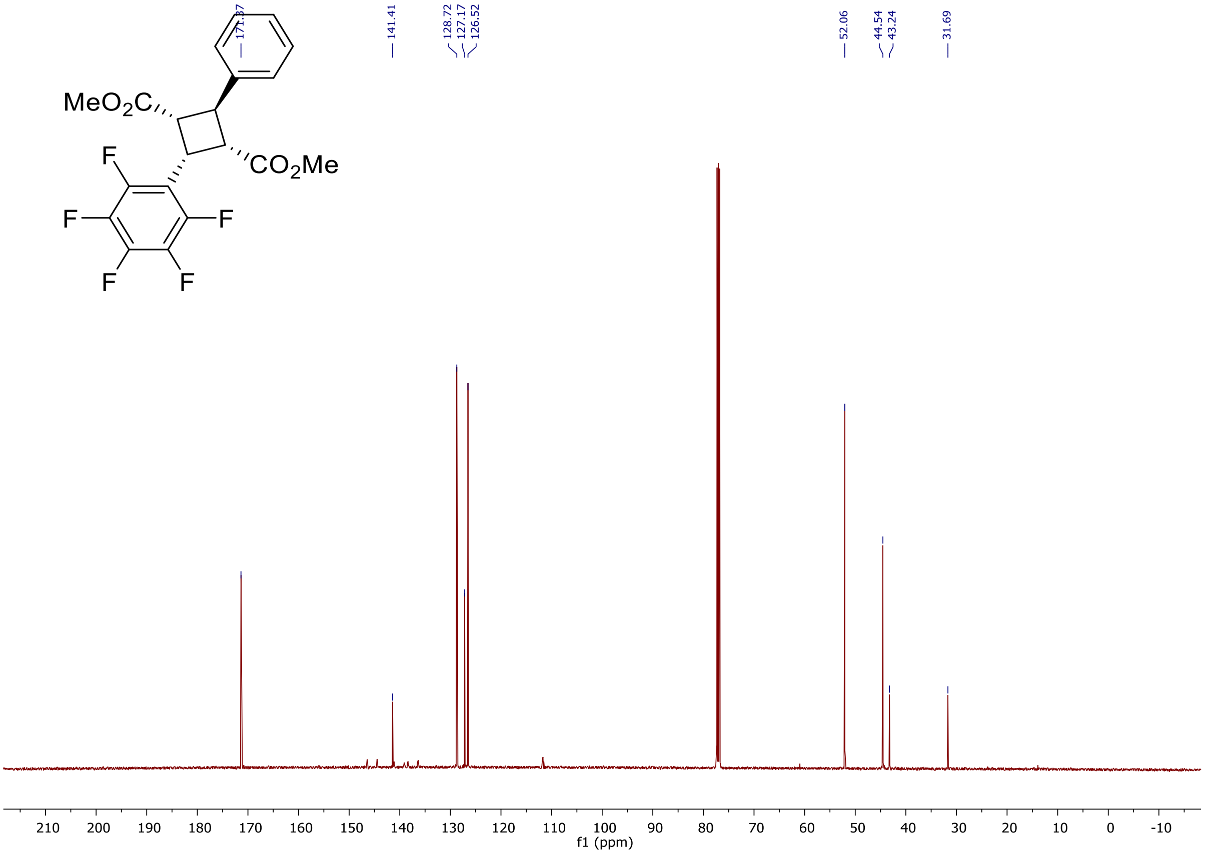

^{19}F NMR of compound 18

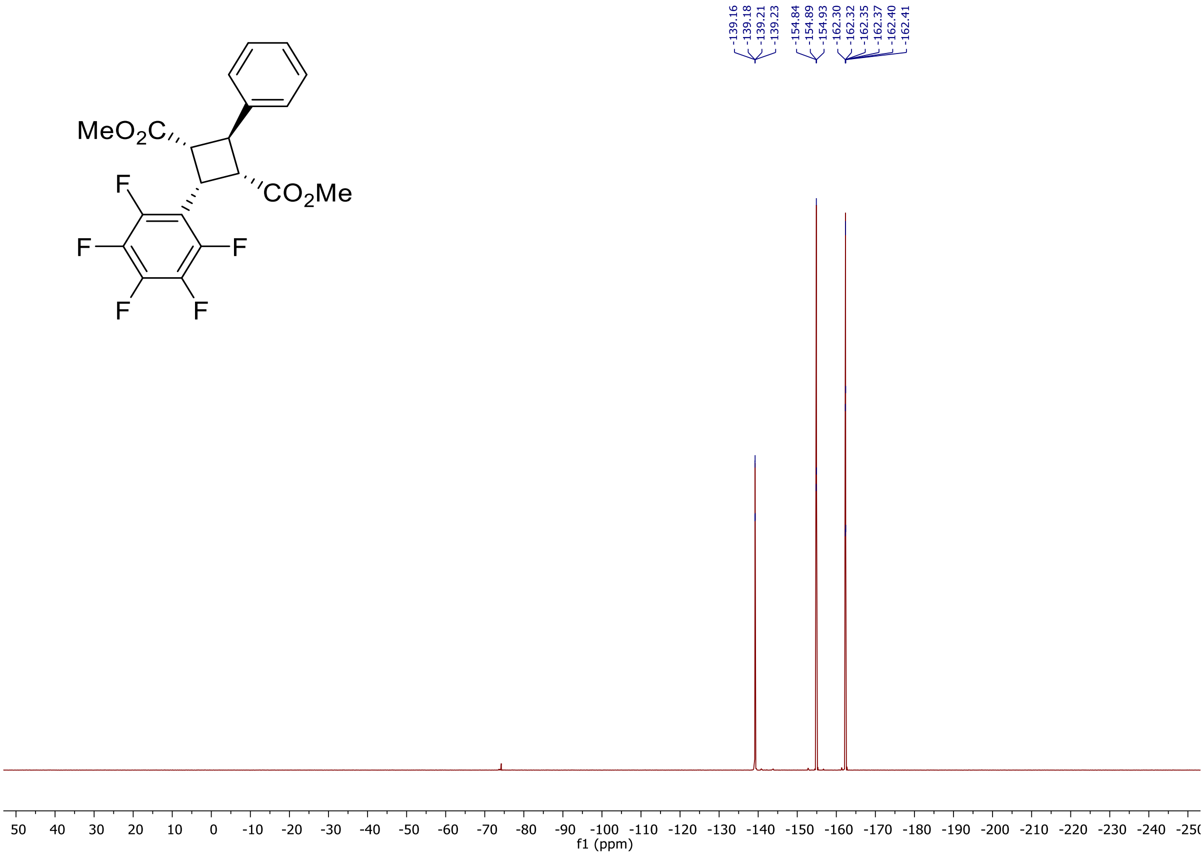

^{1}H NMR of Compound 19

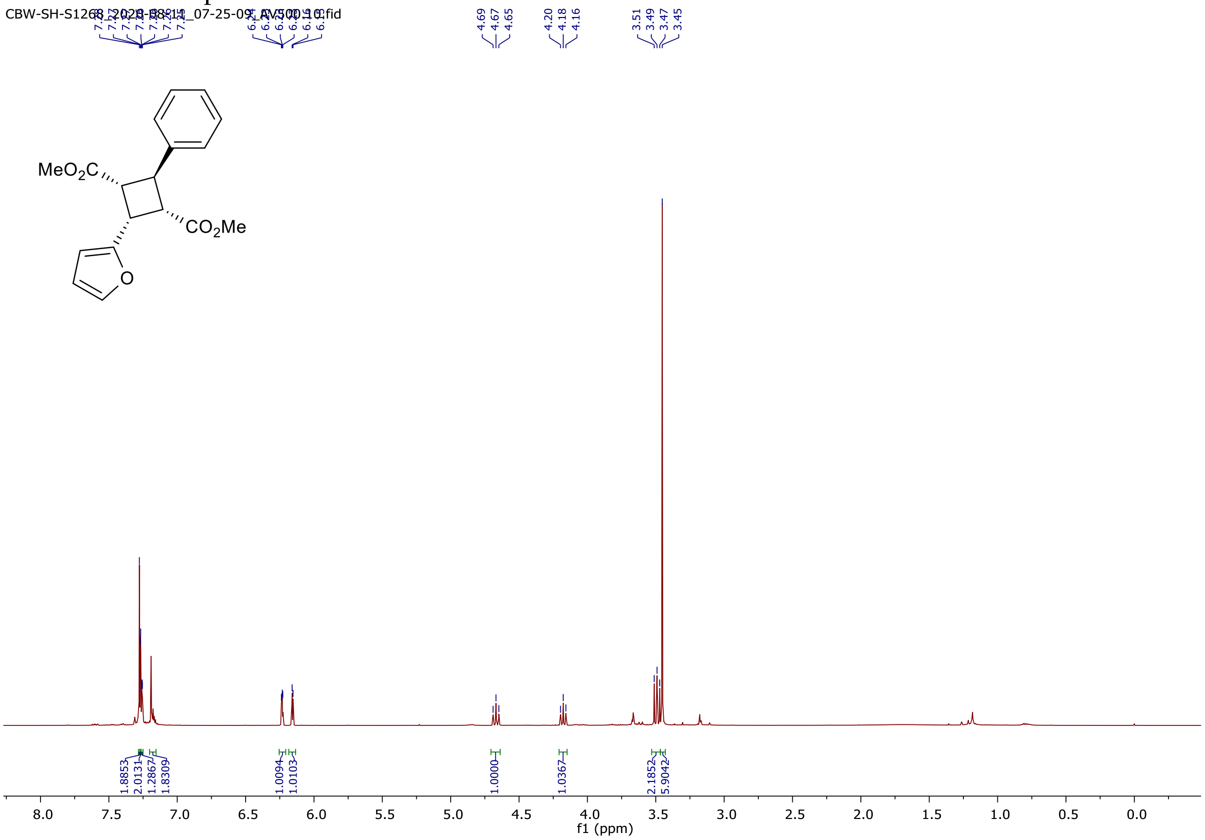

^{13}C NMR of Compound 19

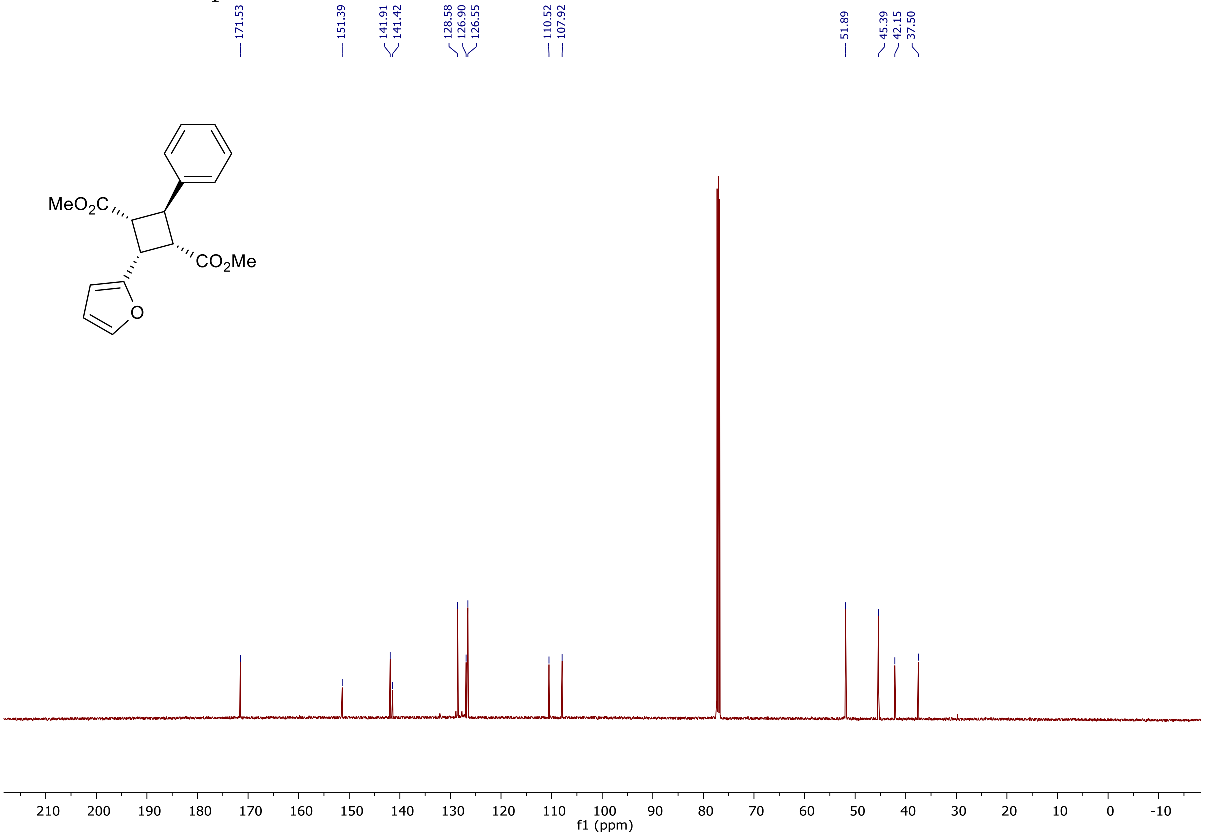

^{1}H NMR of Compound 20

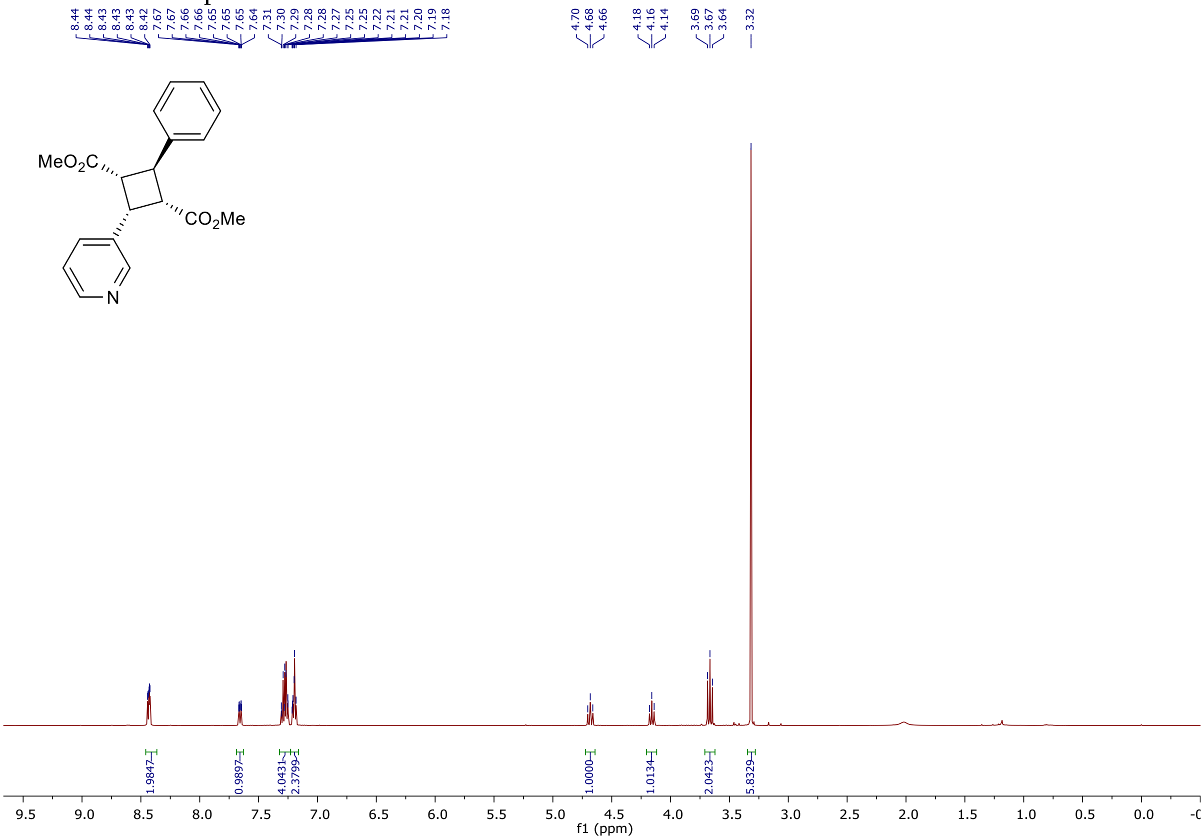

^{1C} NMR of Compound 20

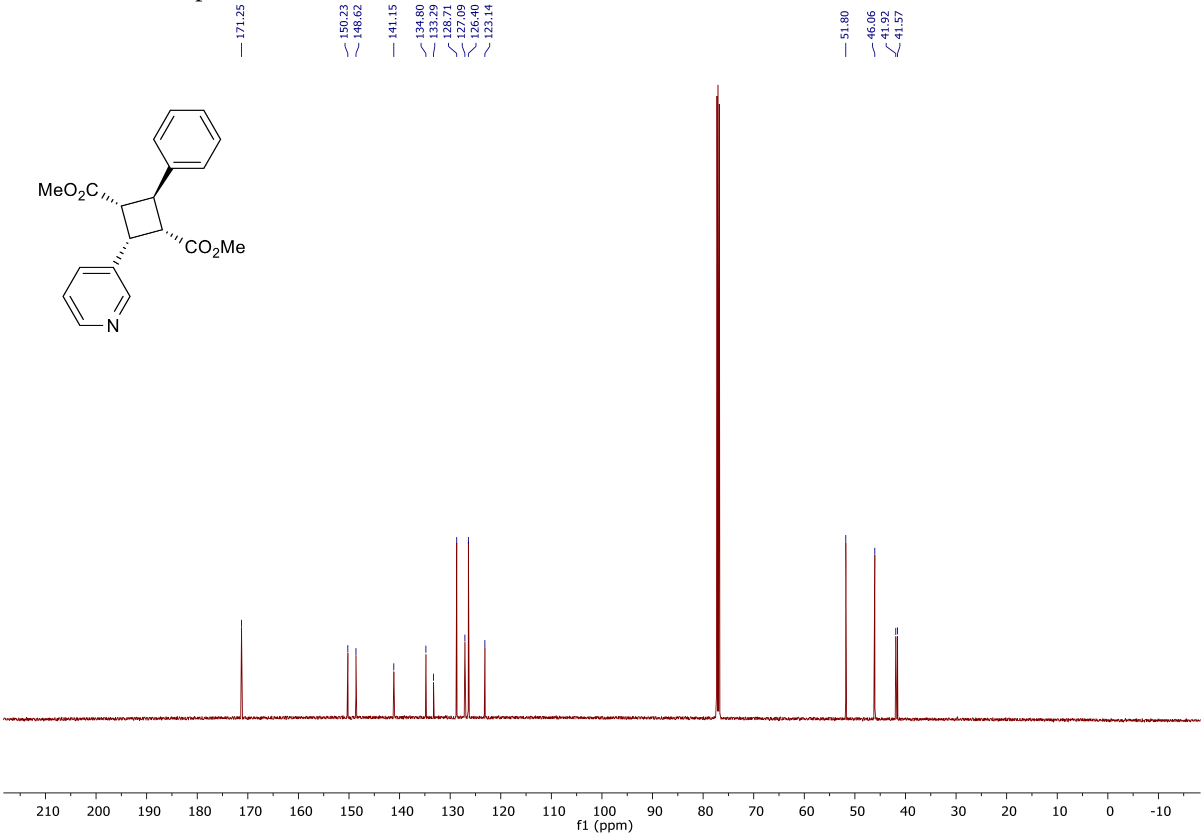

^{1}H NMR of compound 21

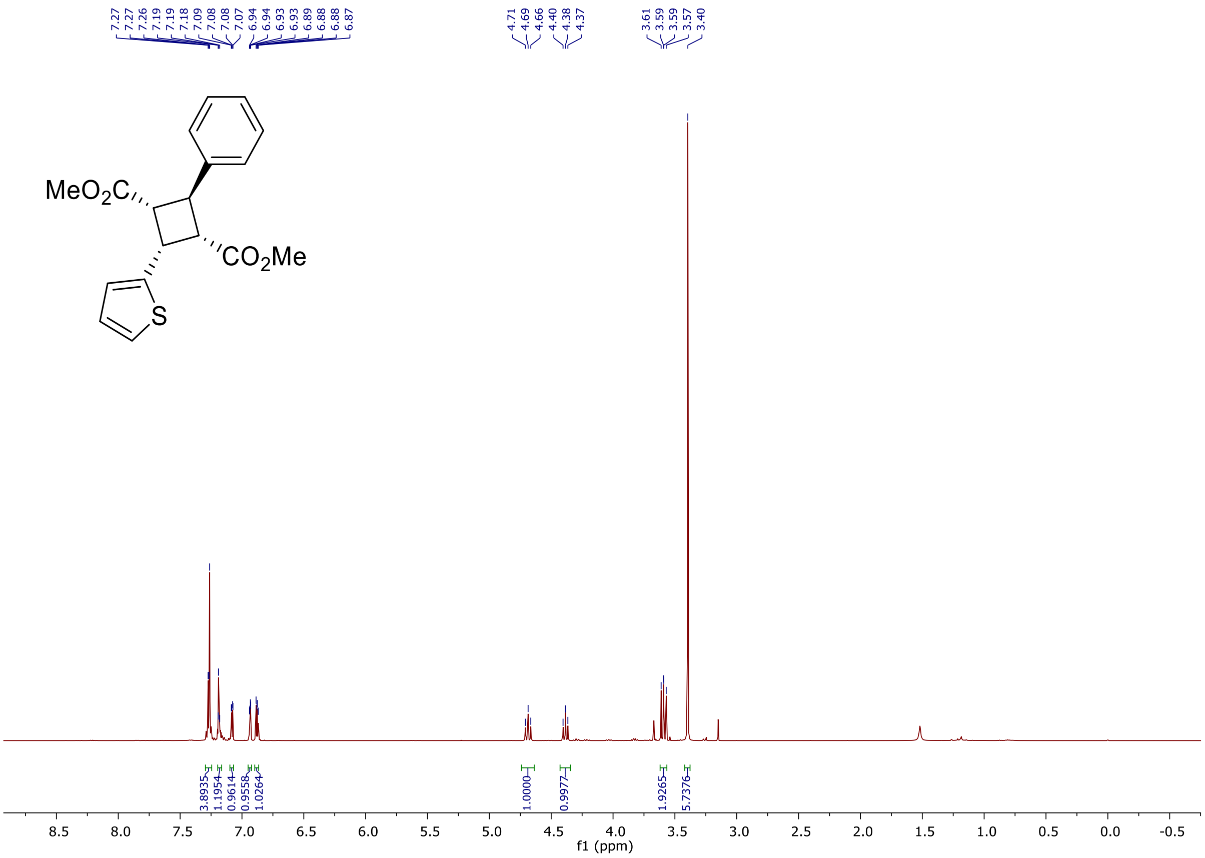

^{13}C NMR of compound 21

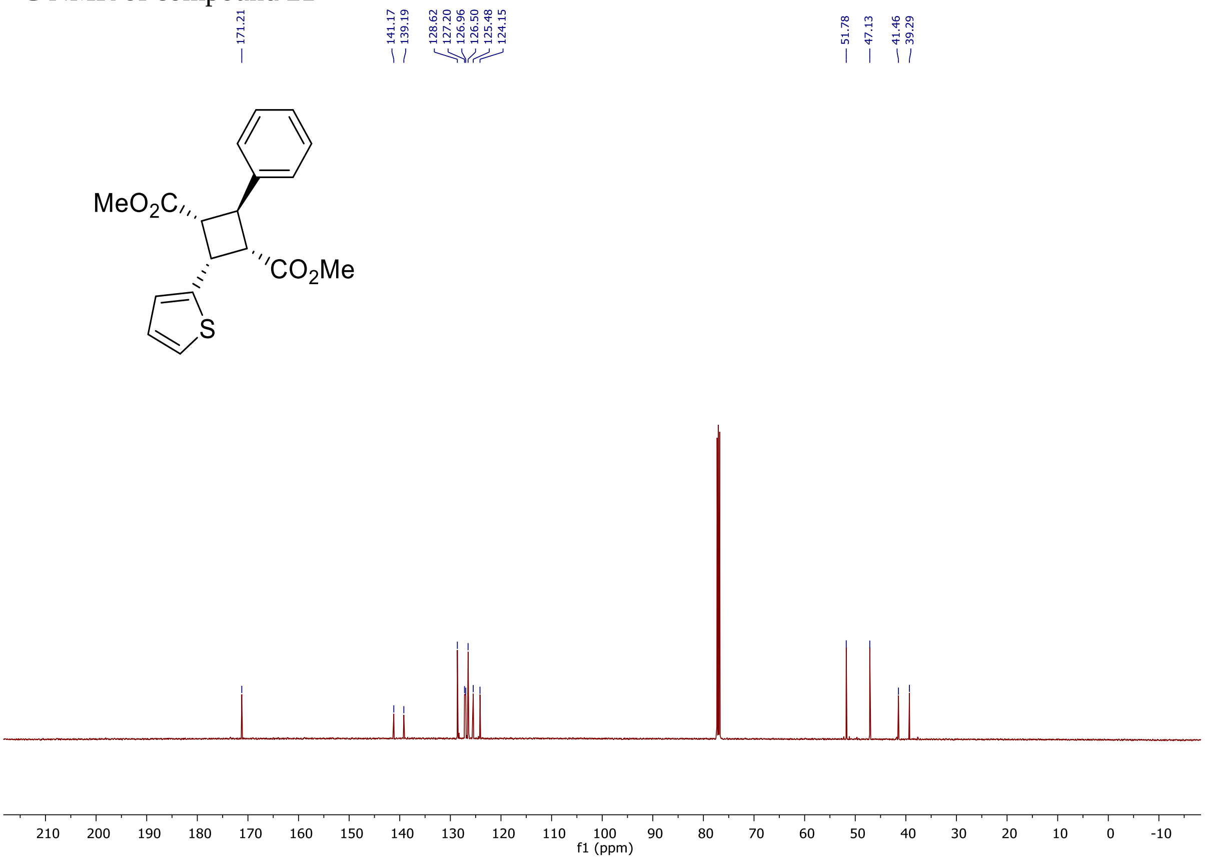

^{1}H NMR of compound 22

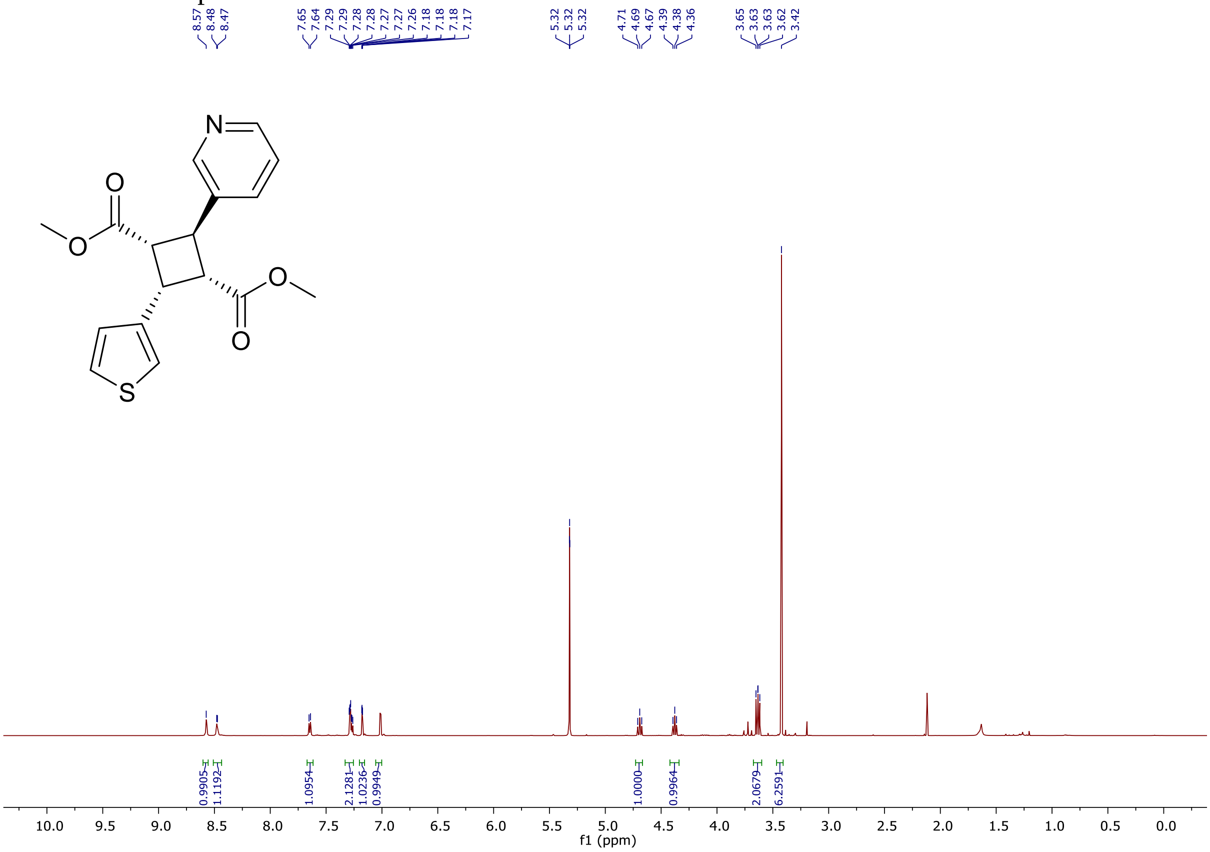

13C NMR of compound 22

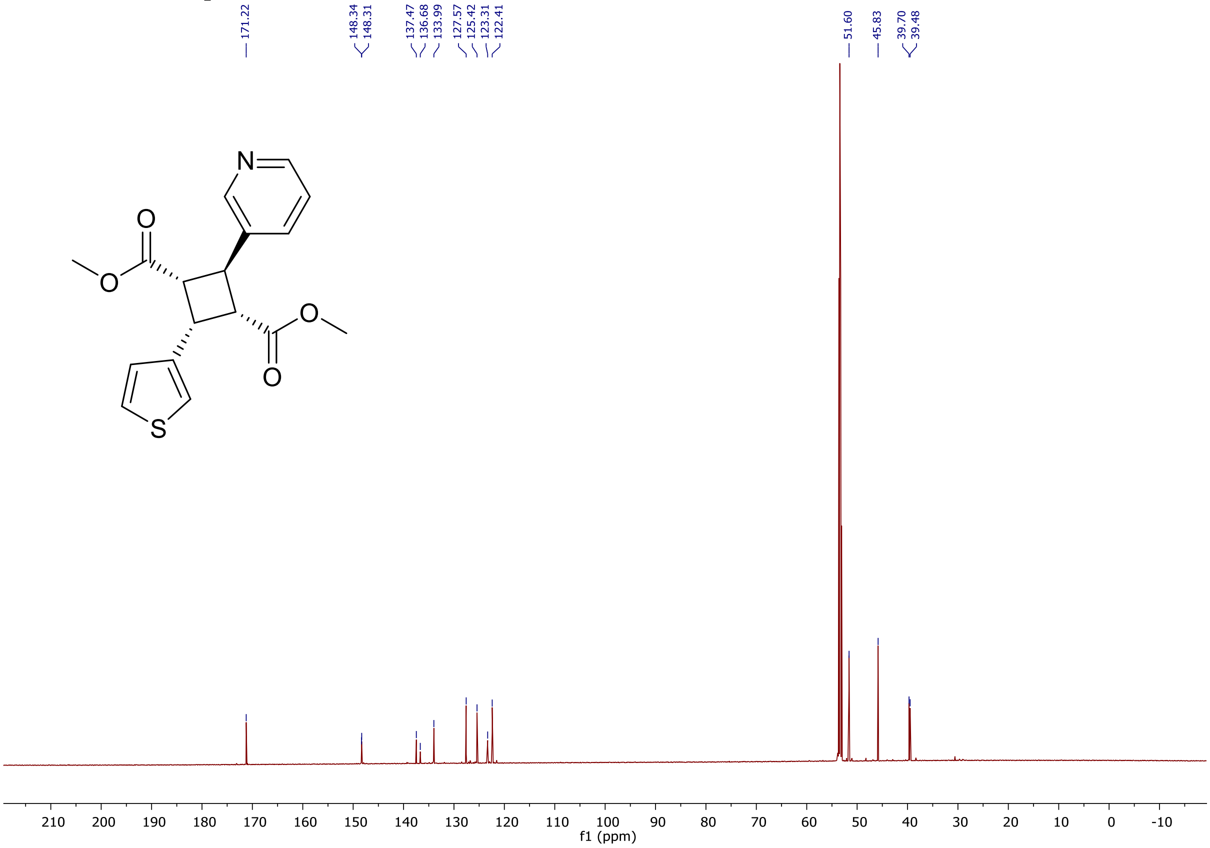

1H NMR of compound 23

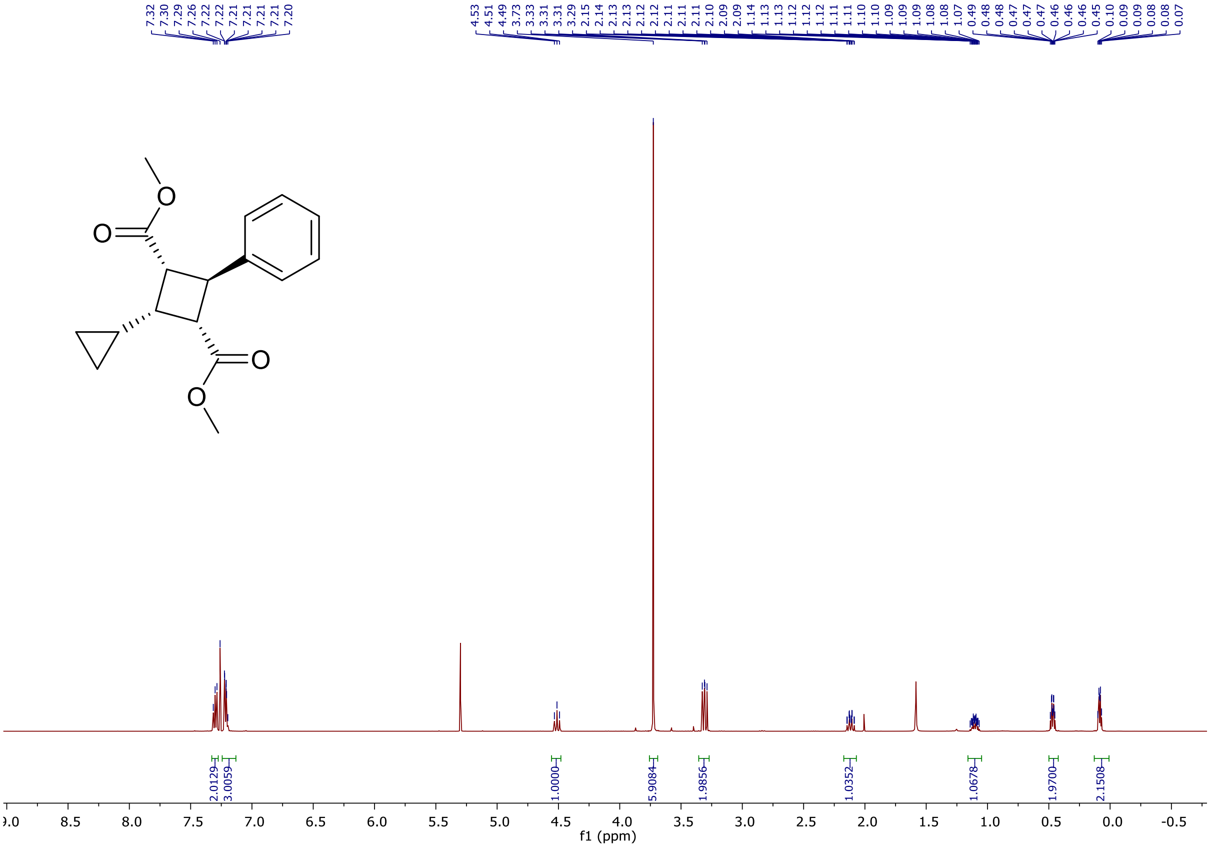

13C NMR of compound 23

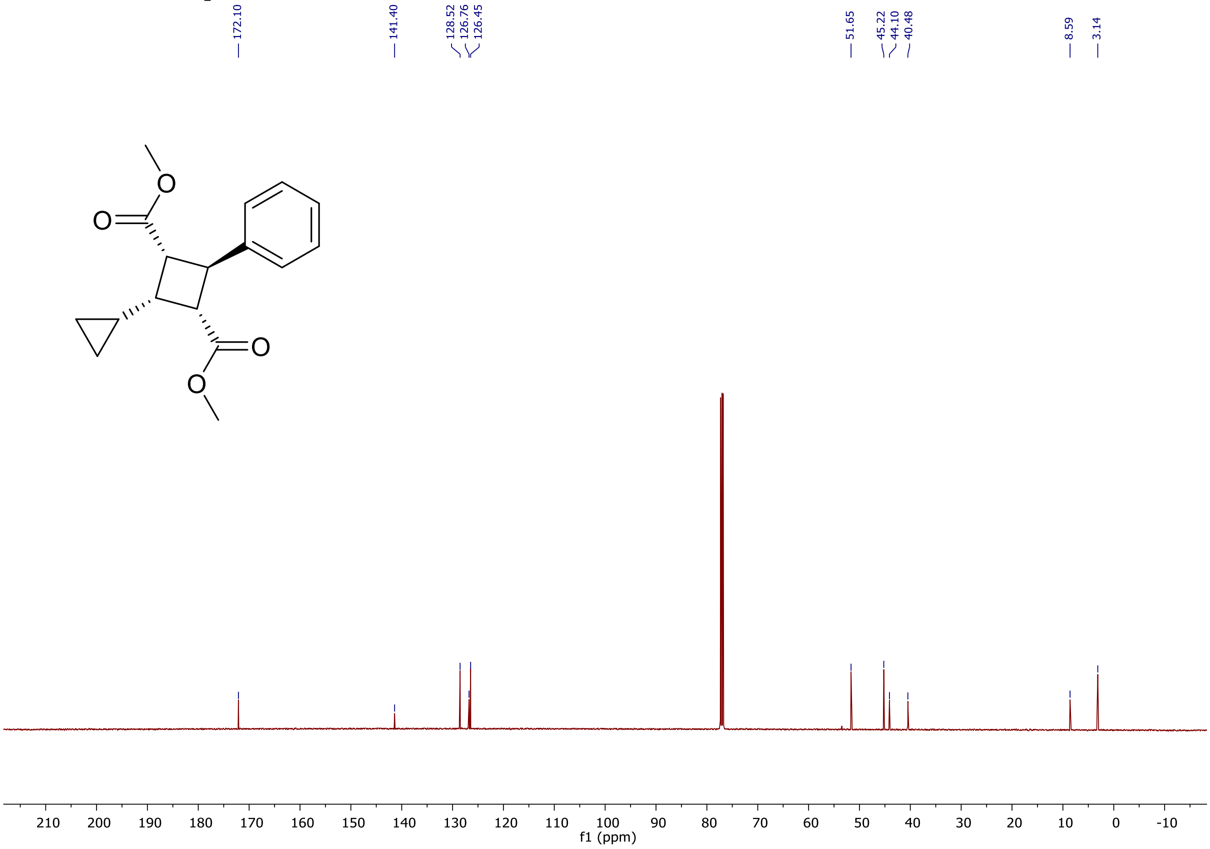

^{1}H NMR of compound 24

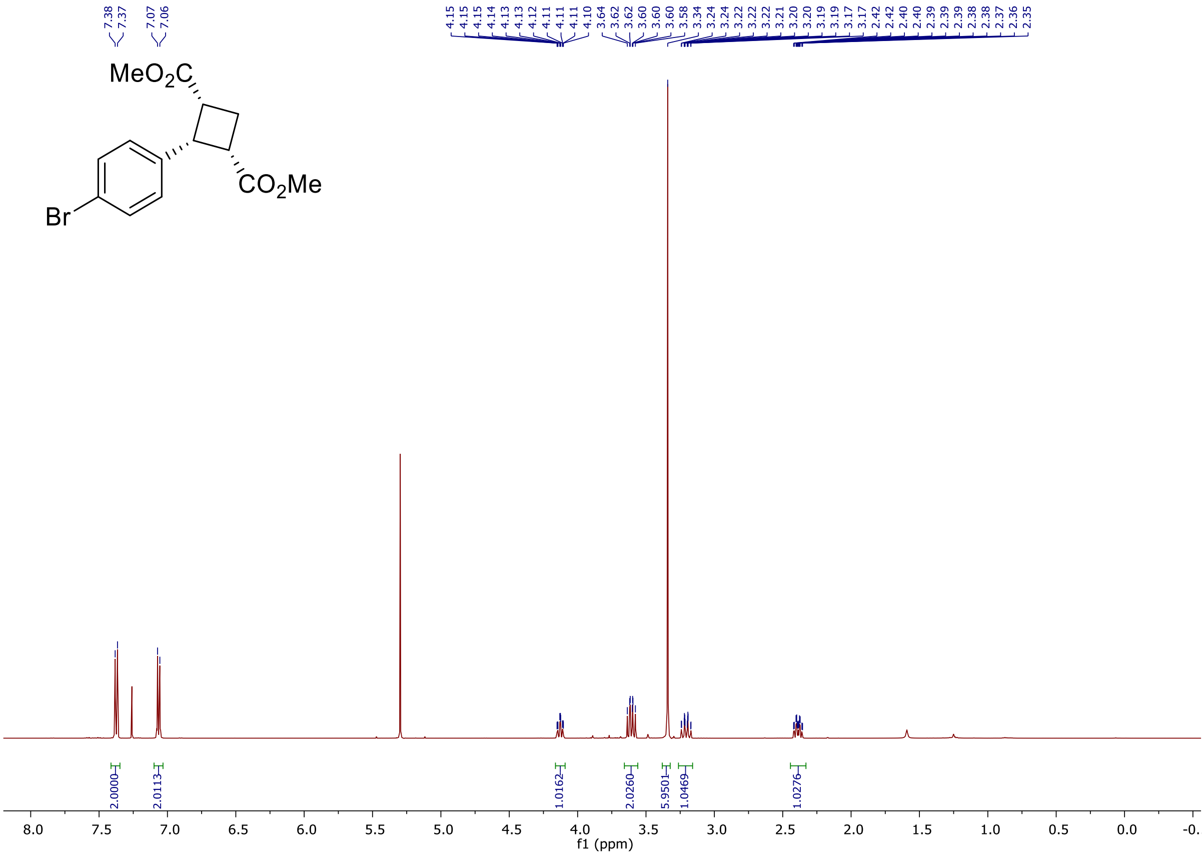

^{13}C NMR of compound 24

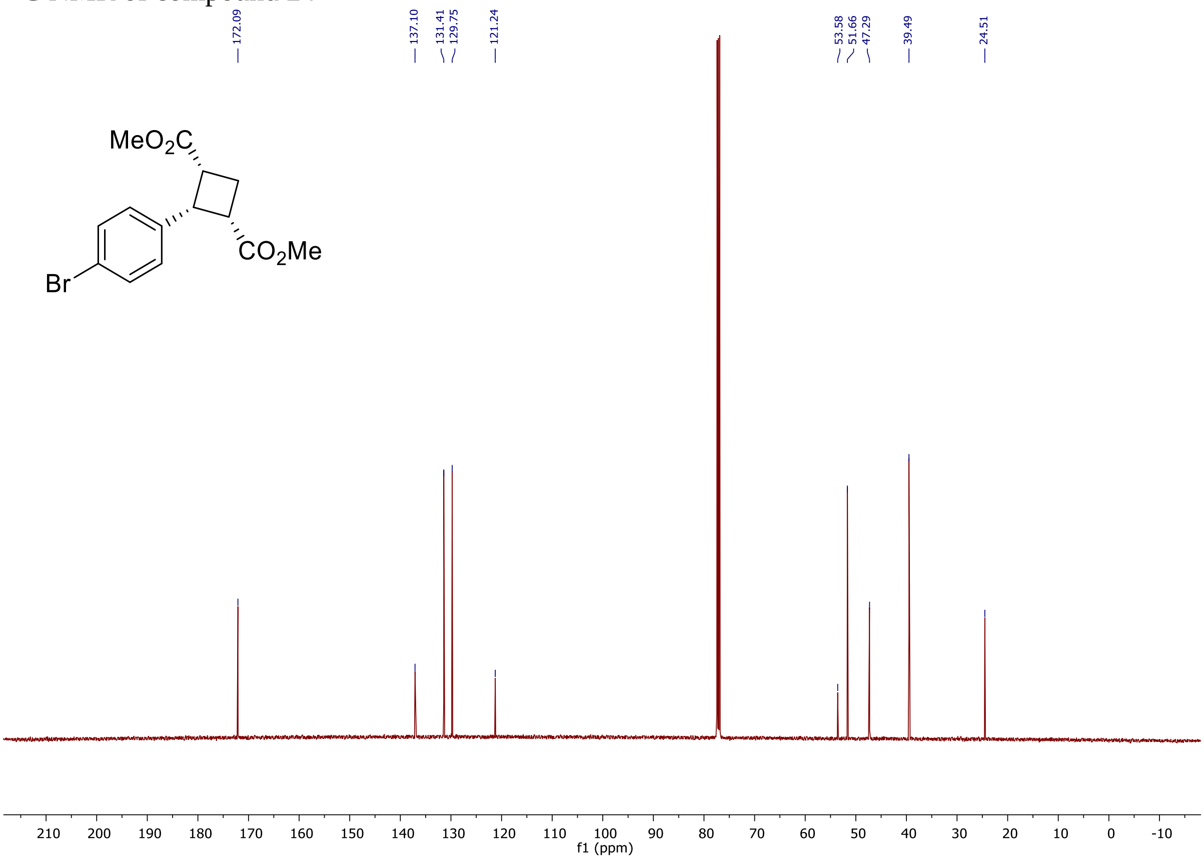

1H NMR of compound 25

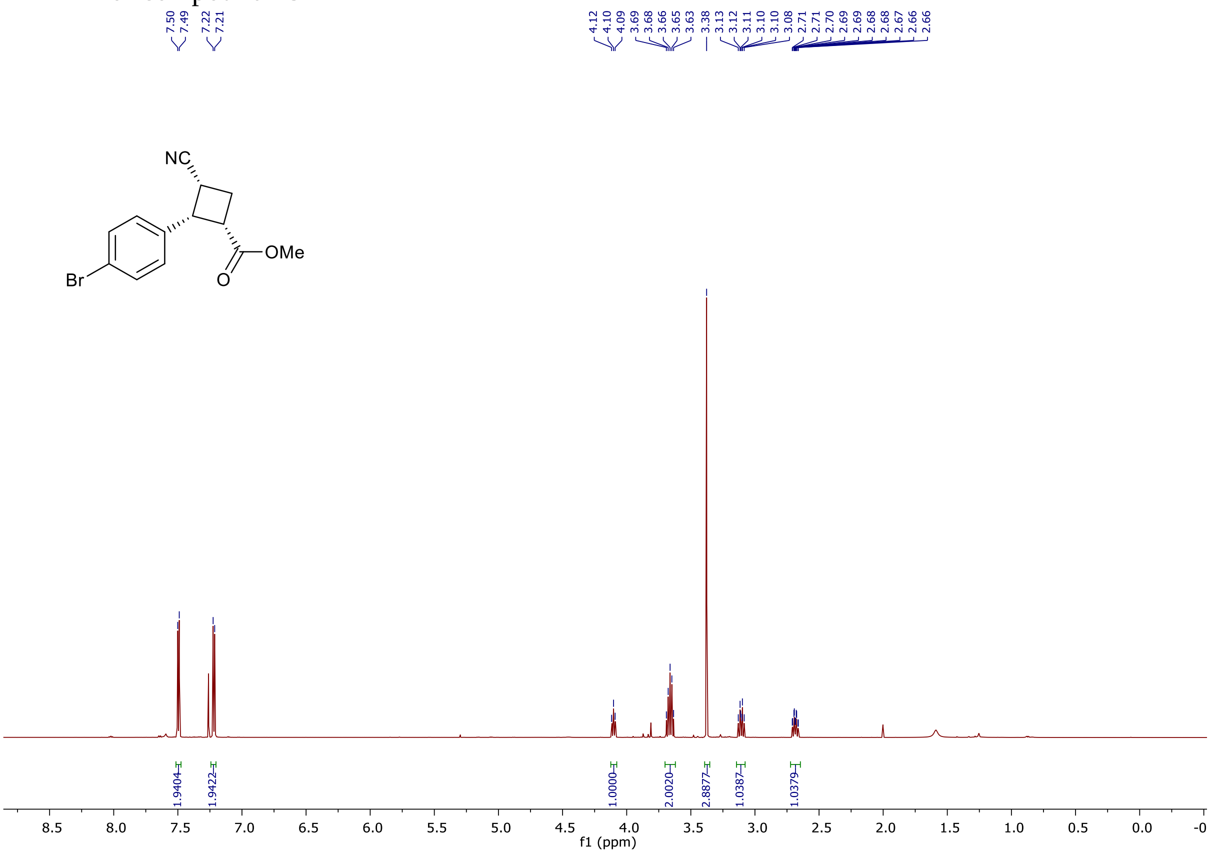

13C NMR of compound 25

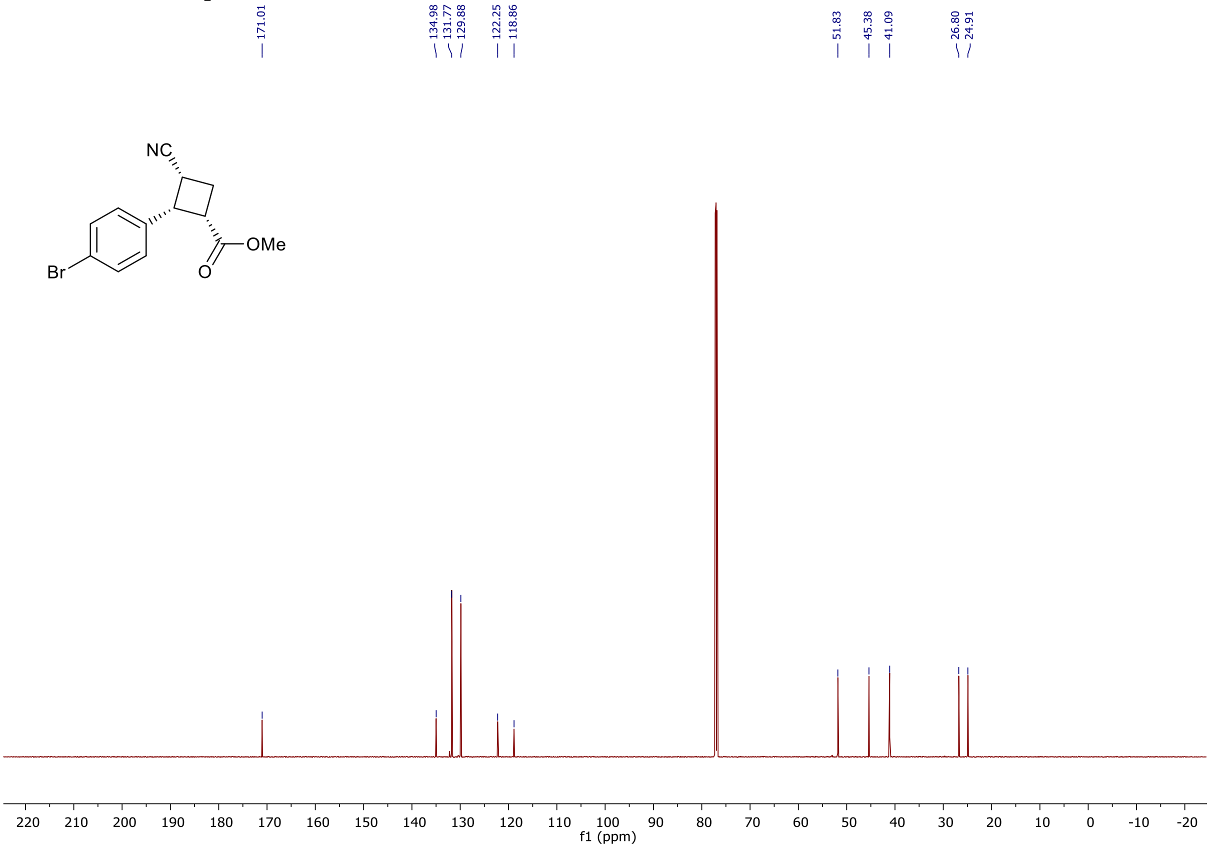

1H NMR of compound 26

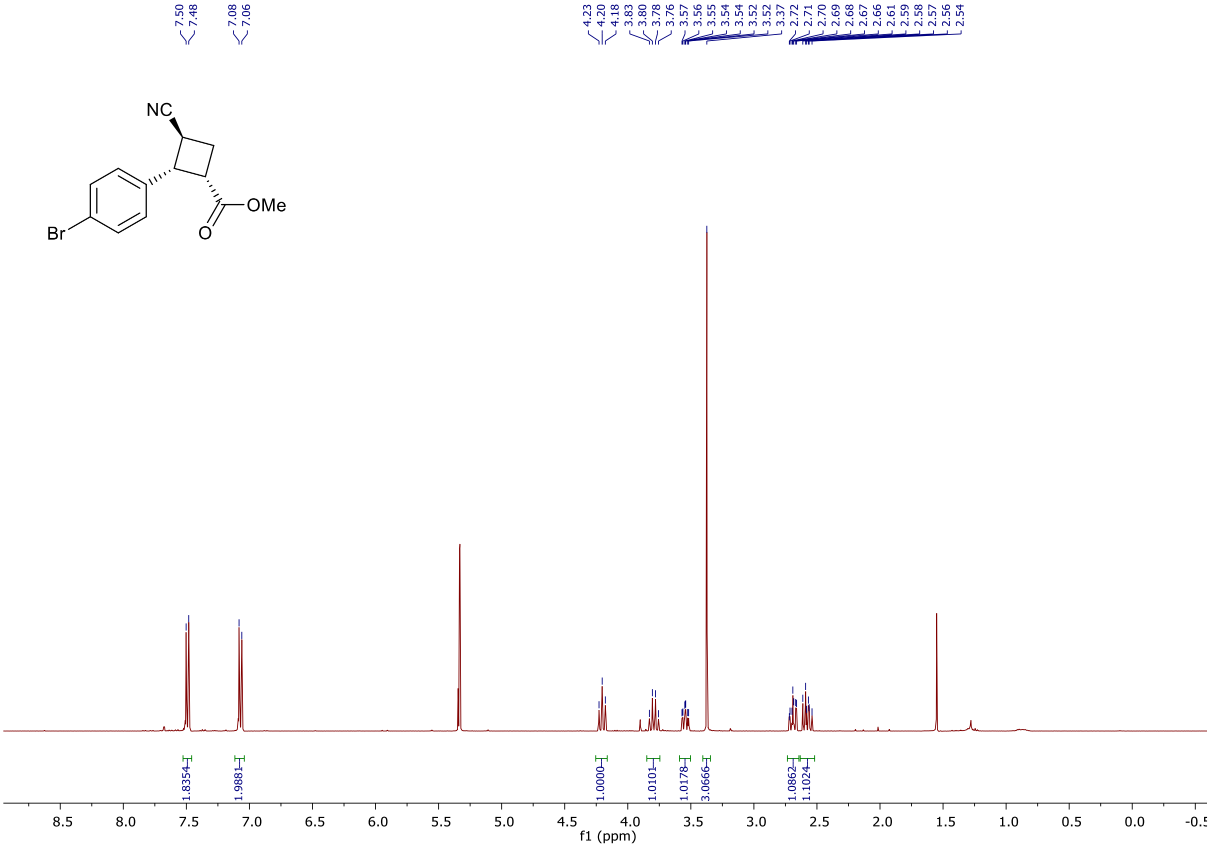

13C NMR of compound 26

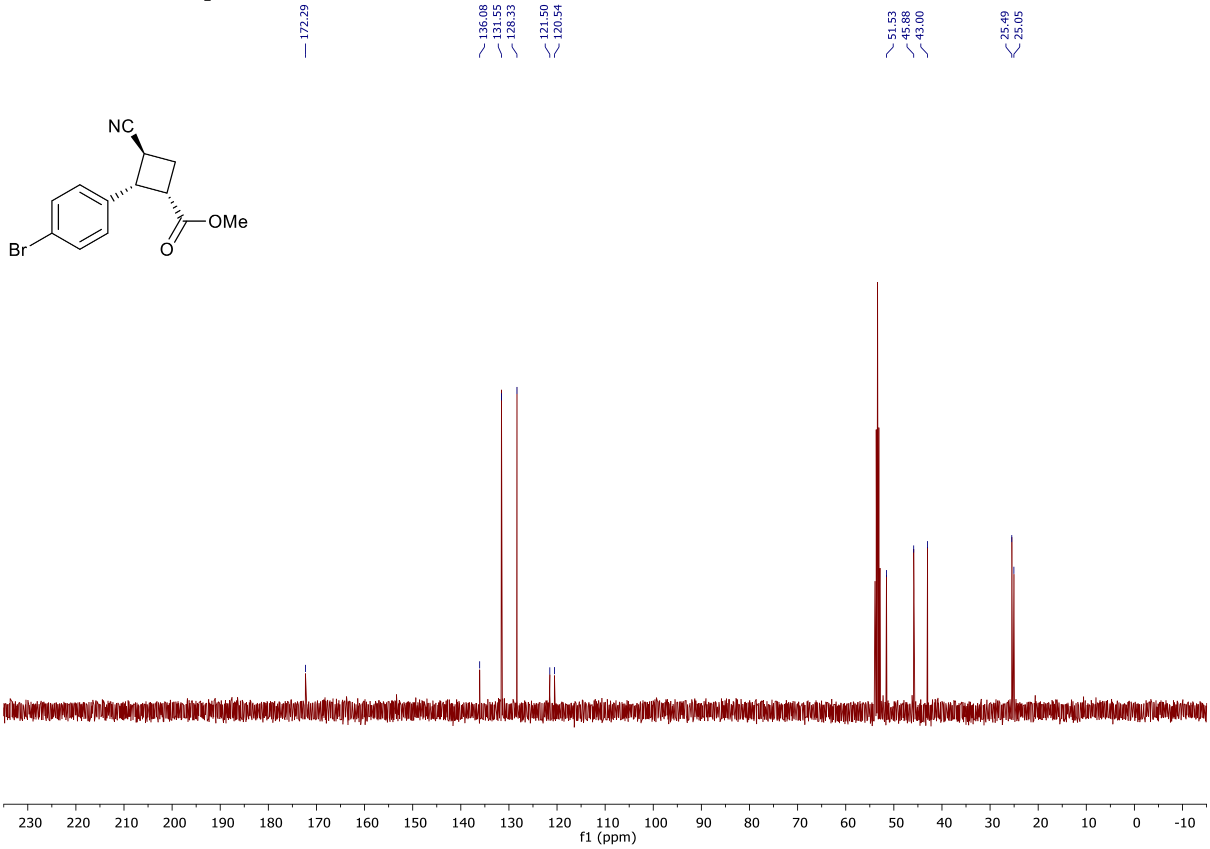

^{1}H NMR of compound 27

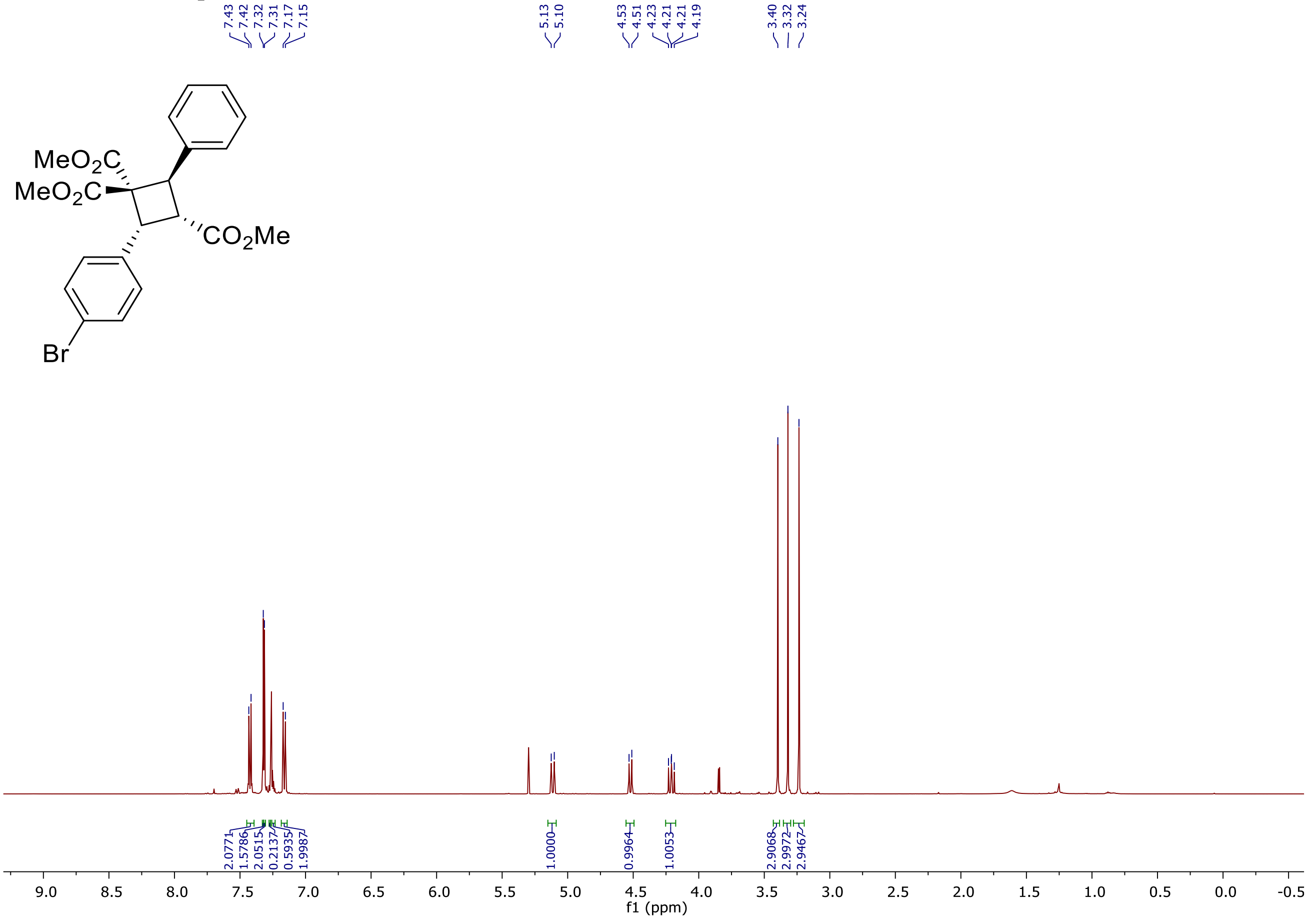

^{13}C NMR of compound 27

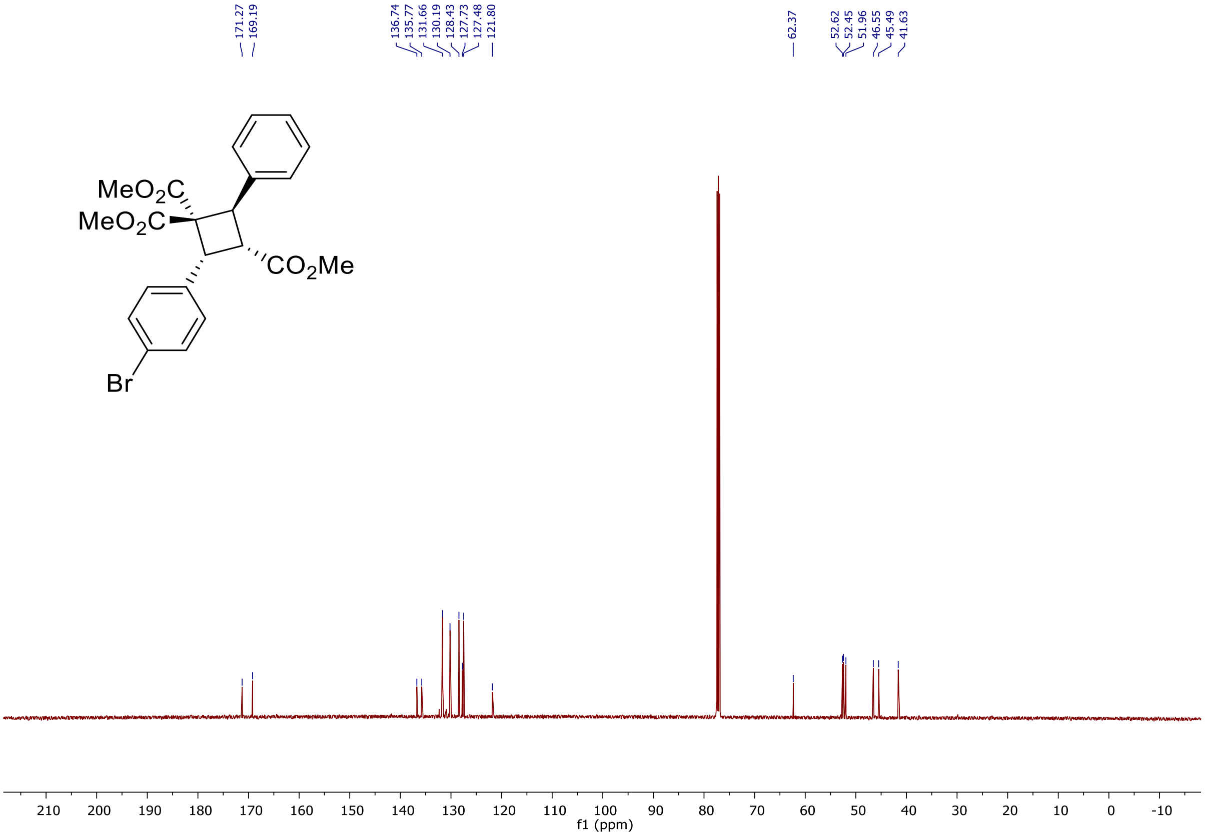

^{1}H NMR of compound 28

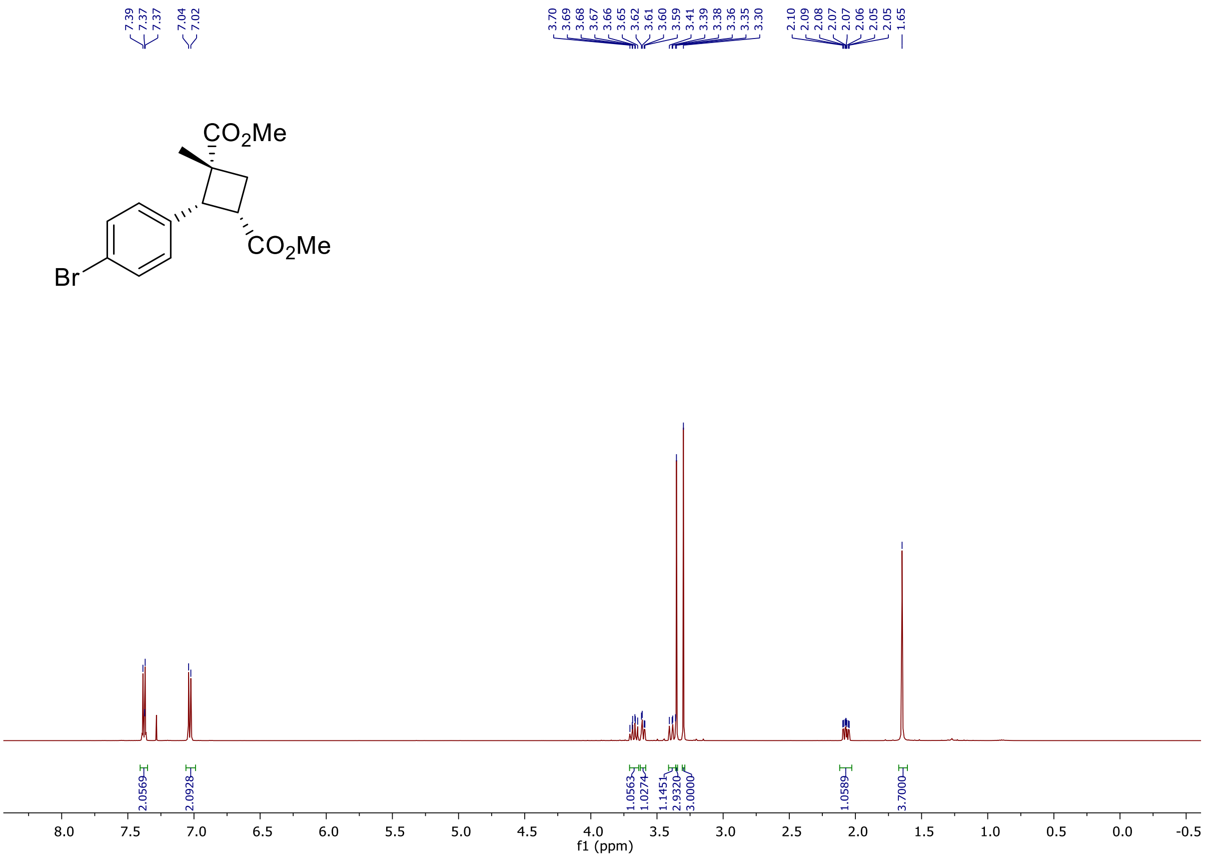

^{13}C NMR of compound 28

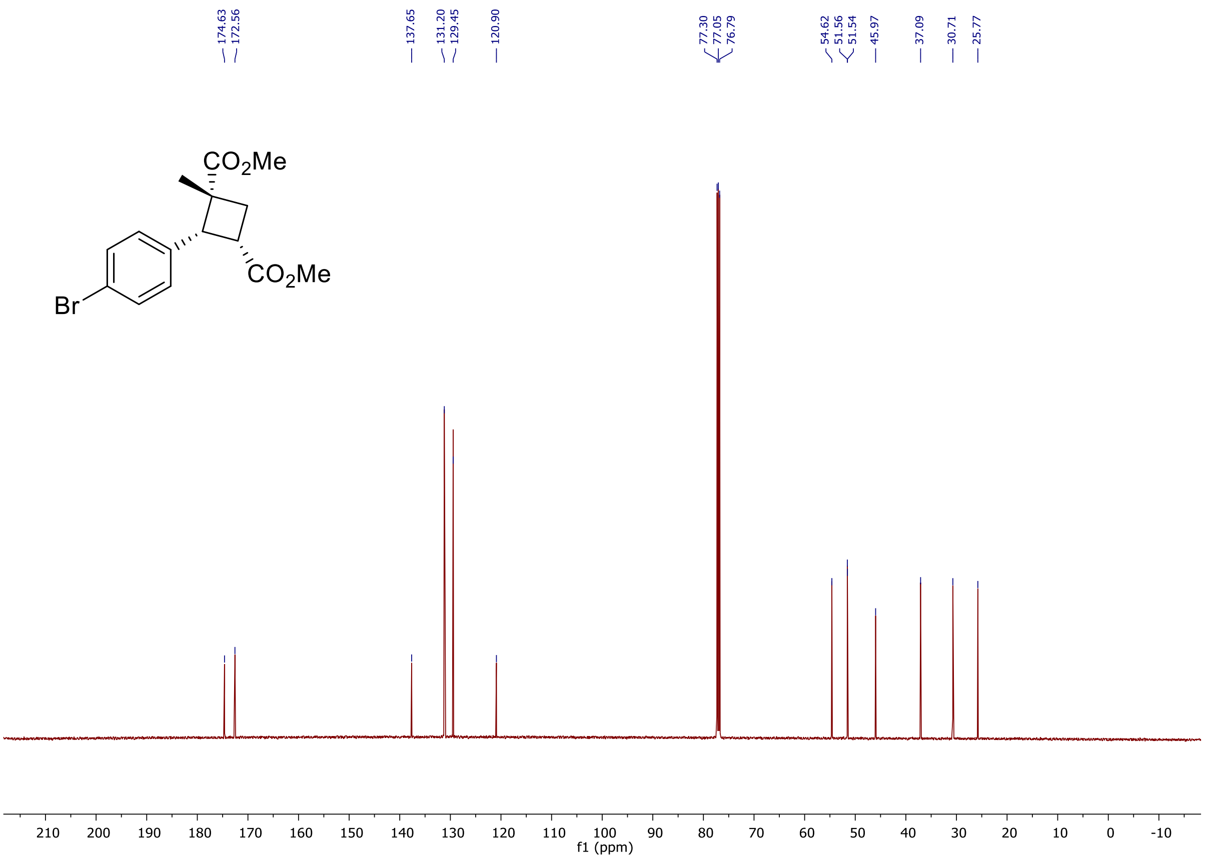

^{1}H NMR of compound 29

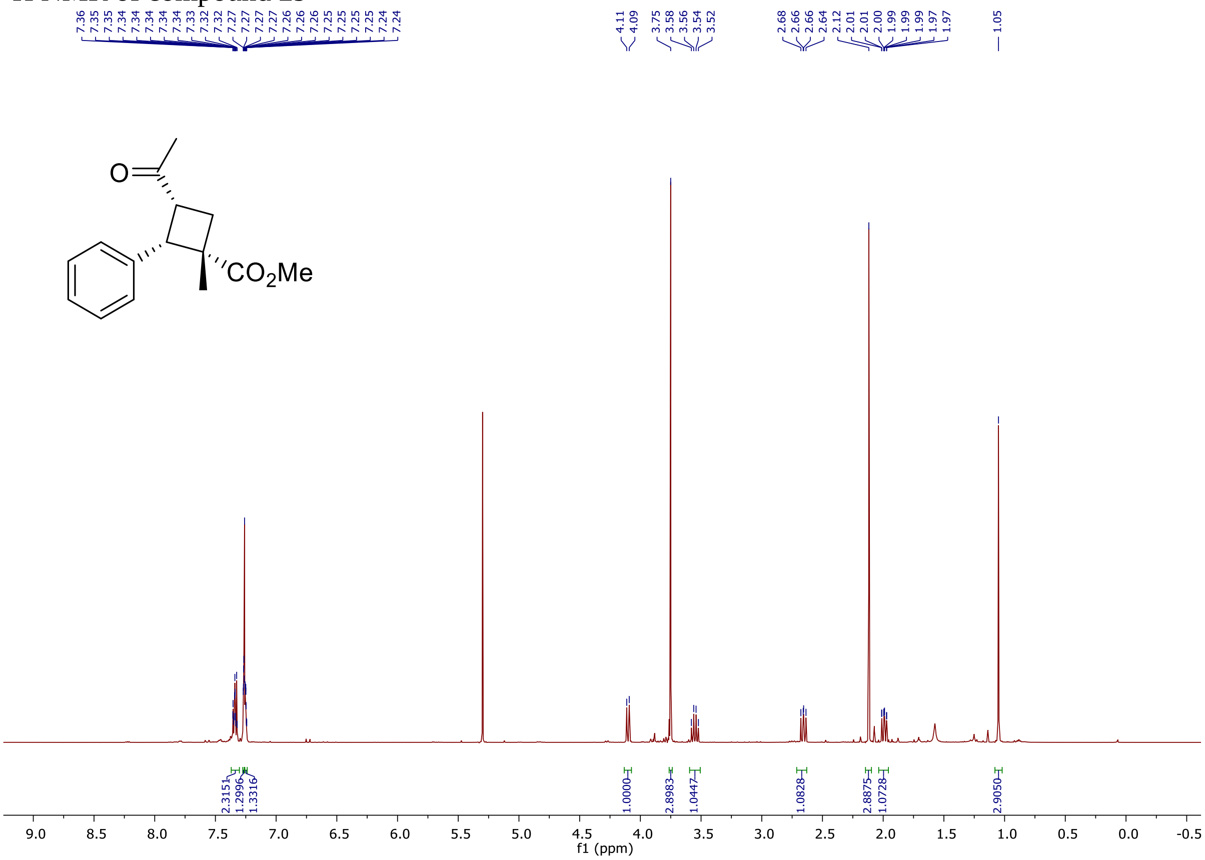

^{13}C NMR of compound 29

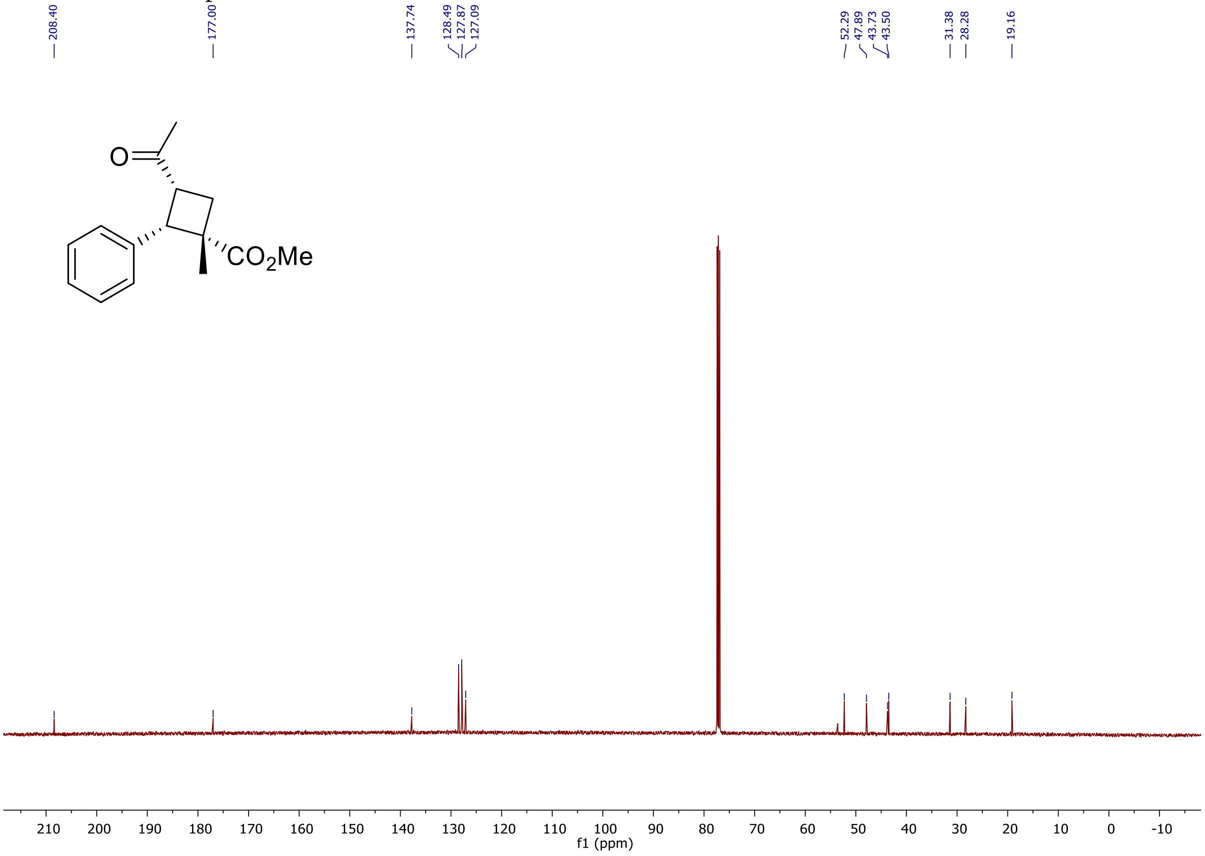

^{1}H NMR of compound 30

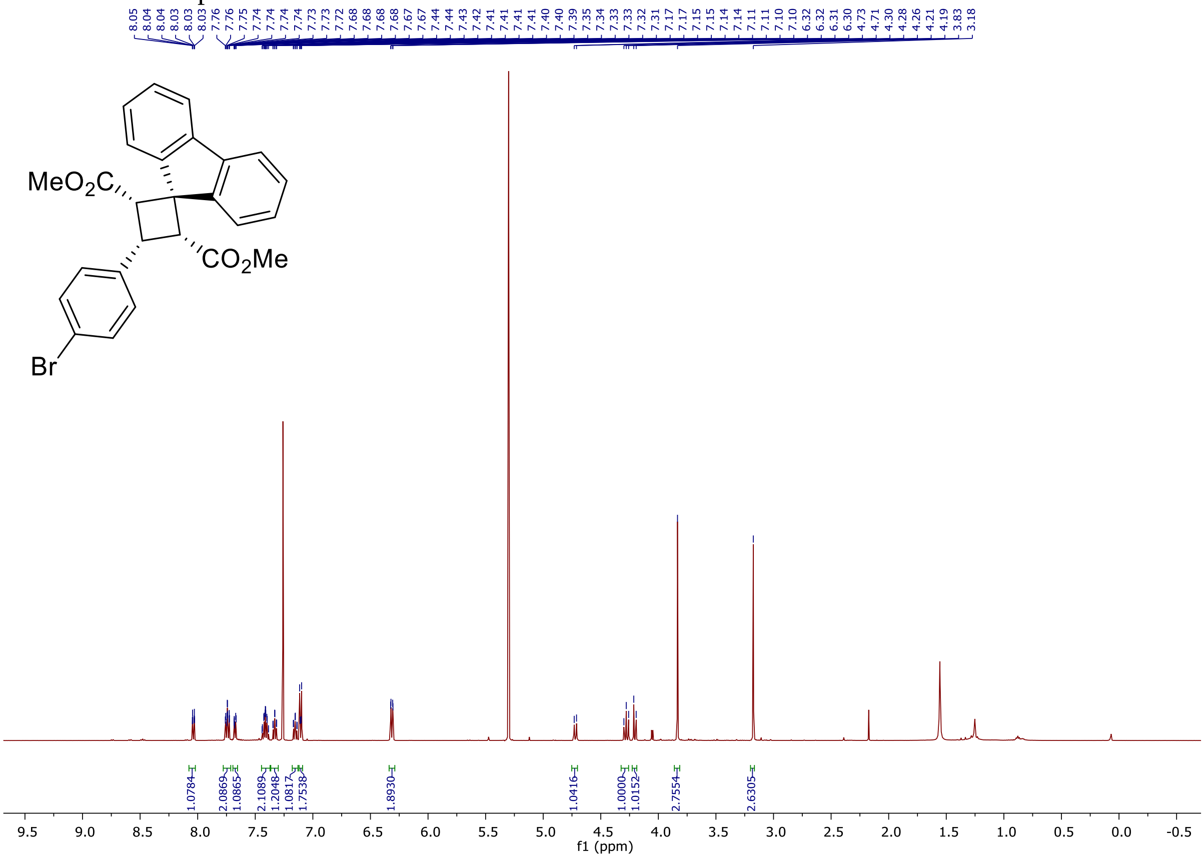

^{13}C NMR of compound 30

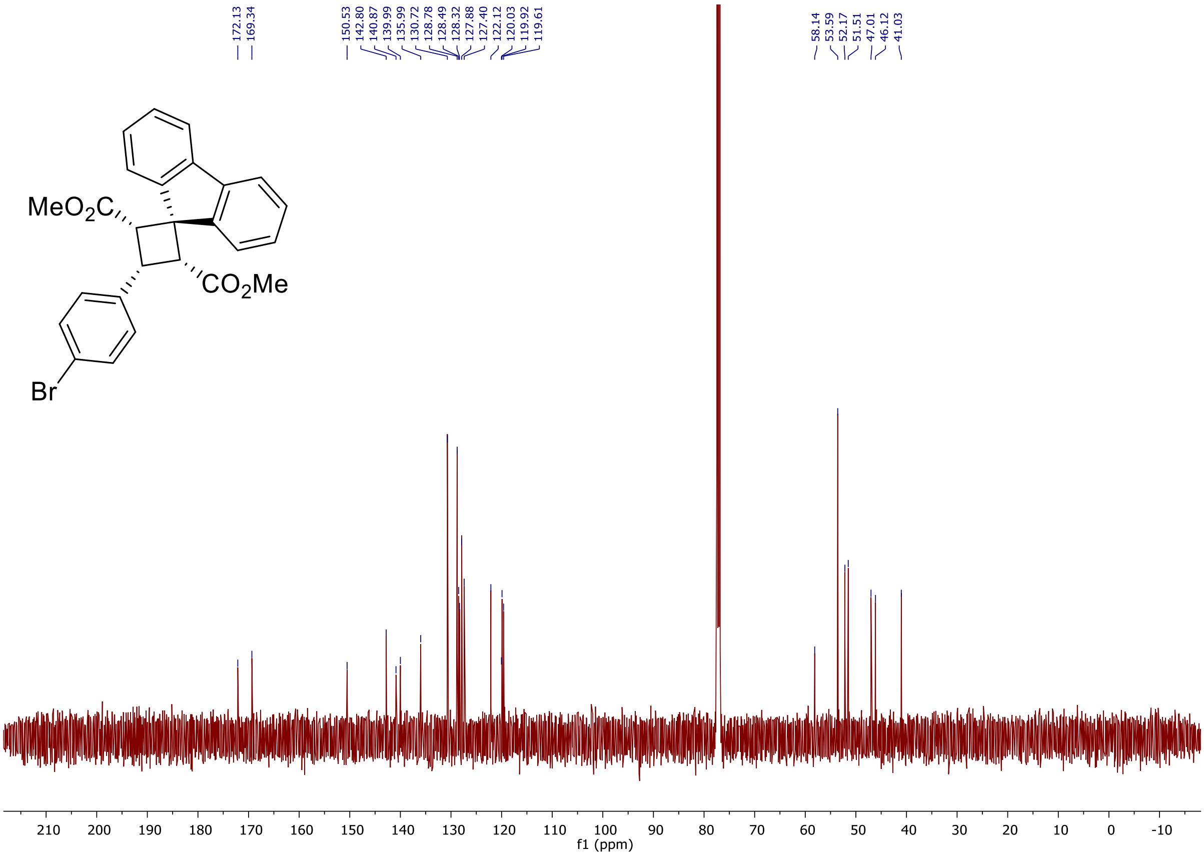

^{1}H NMR of compound 31

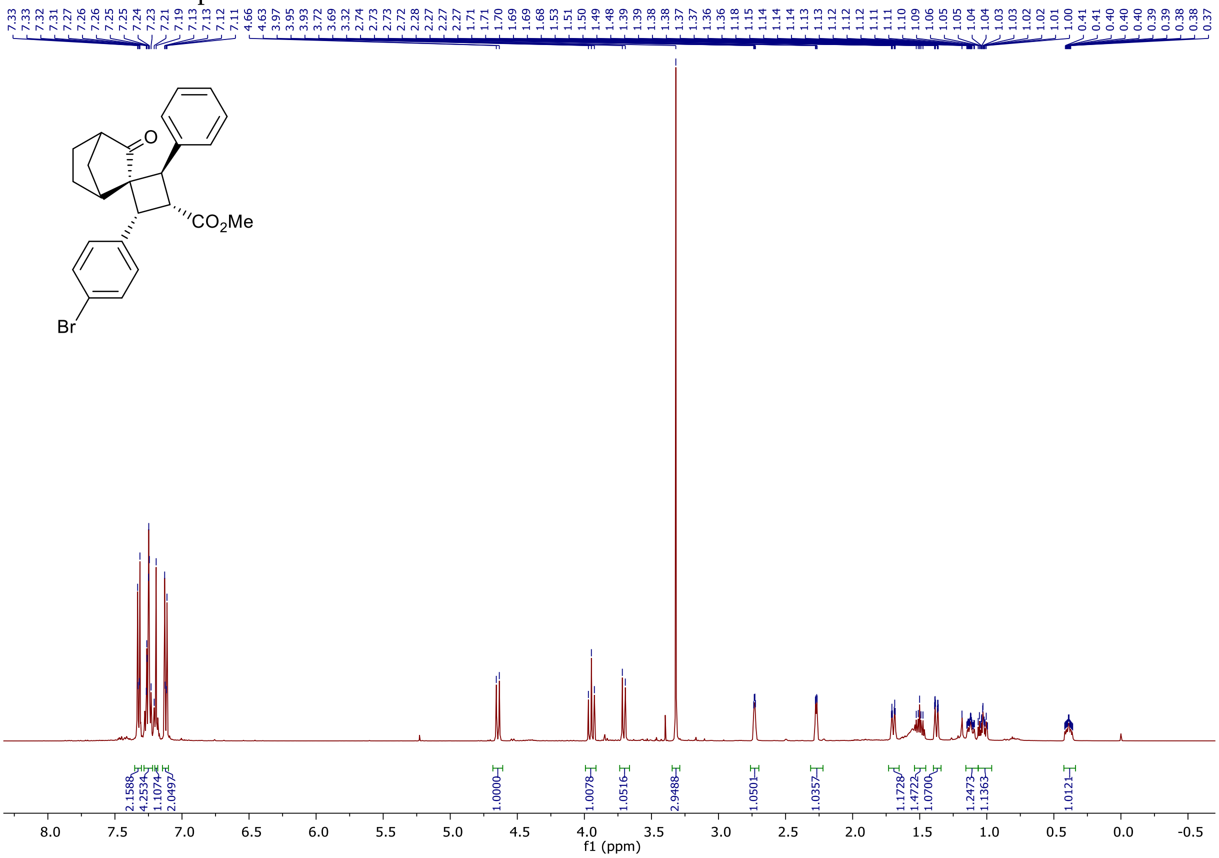

^{13}C NMR of compound 31

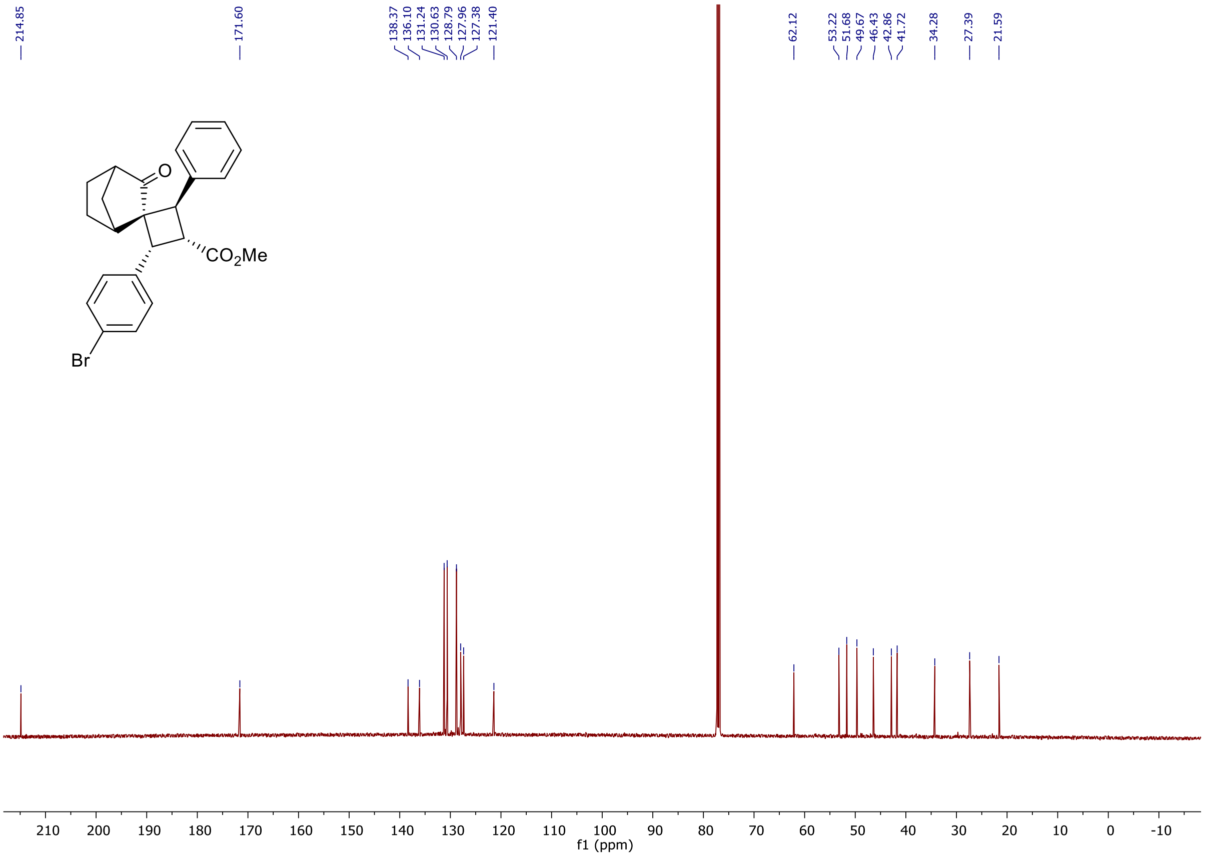

^{1}H NMR of compound 32

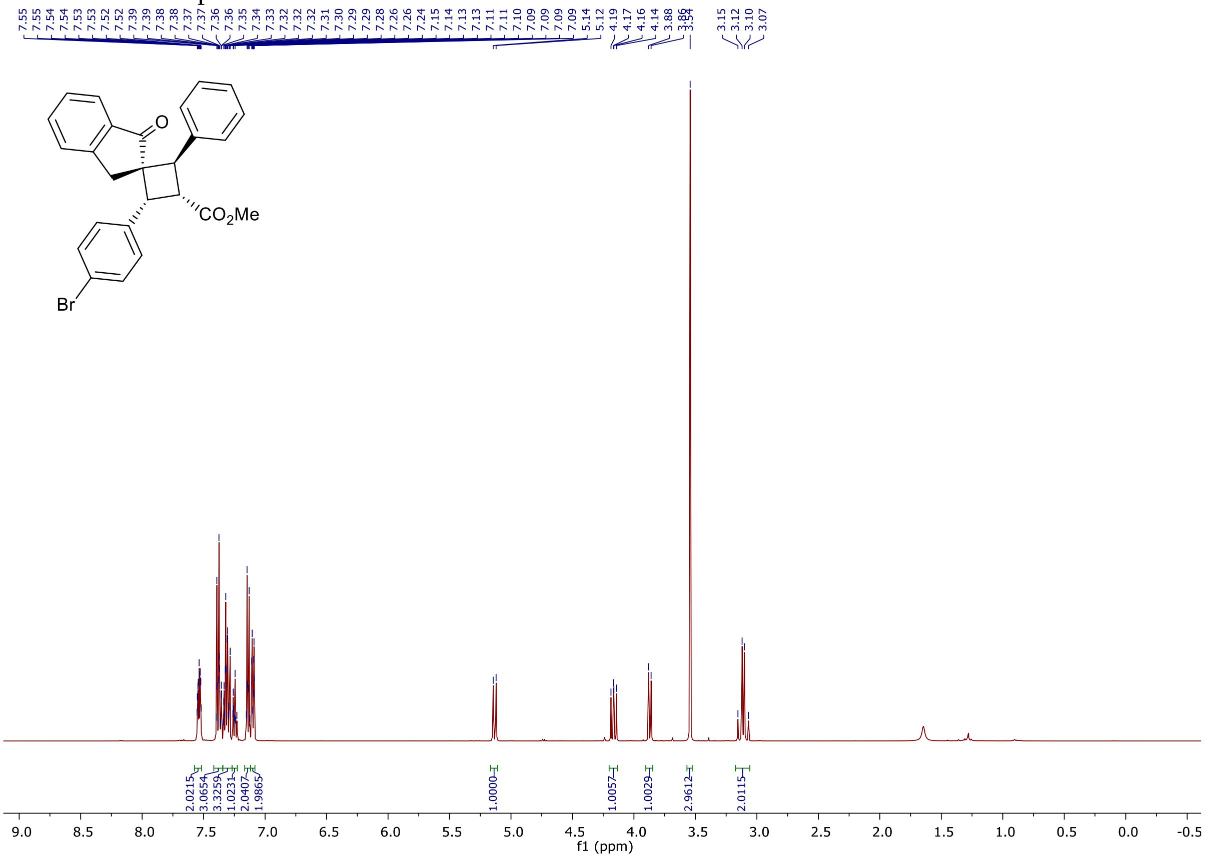

^{13}C NMR of compound 32

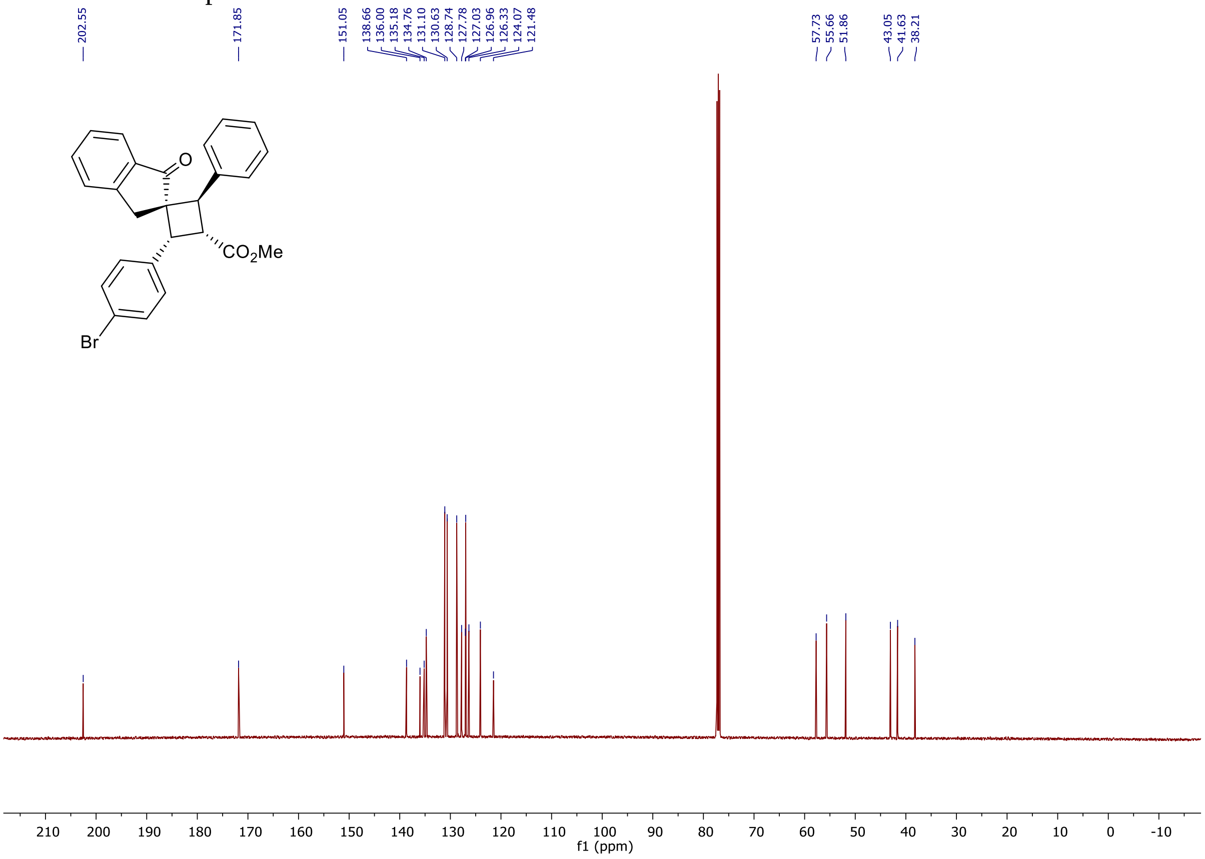

^{1}H NMR of compound 33

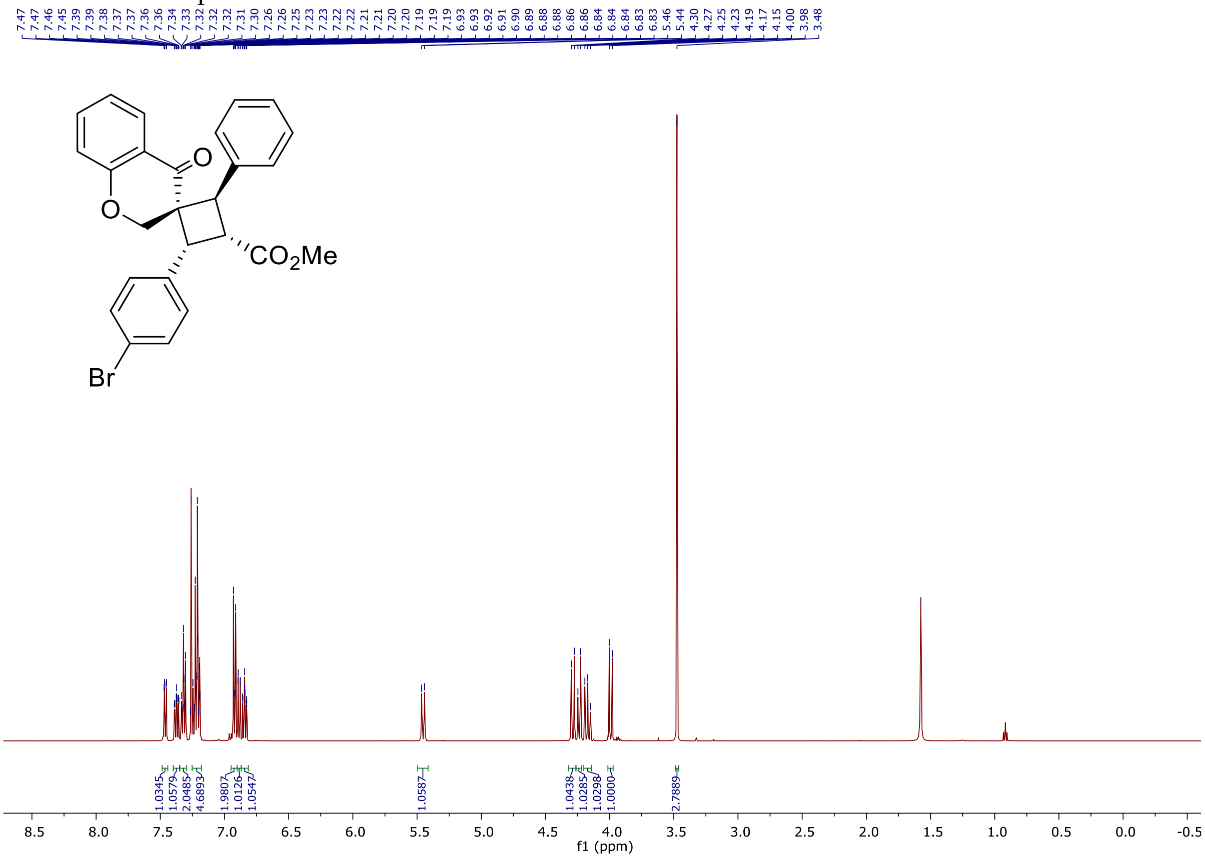

^{13}C NMR of compound 33

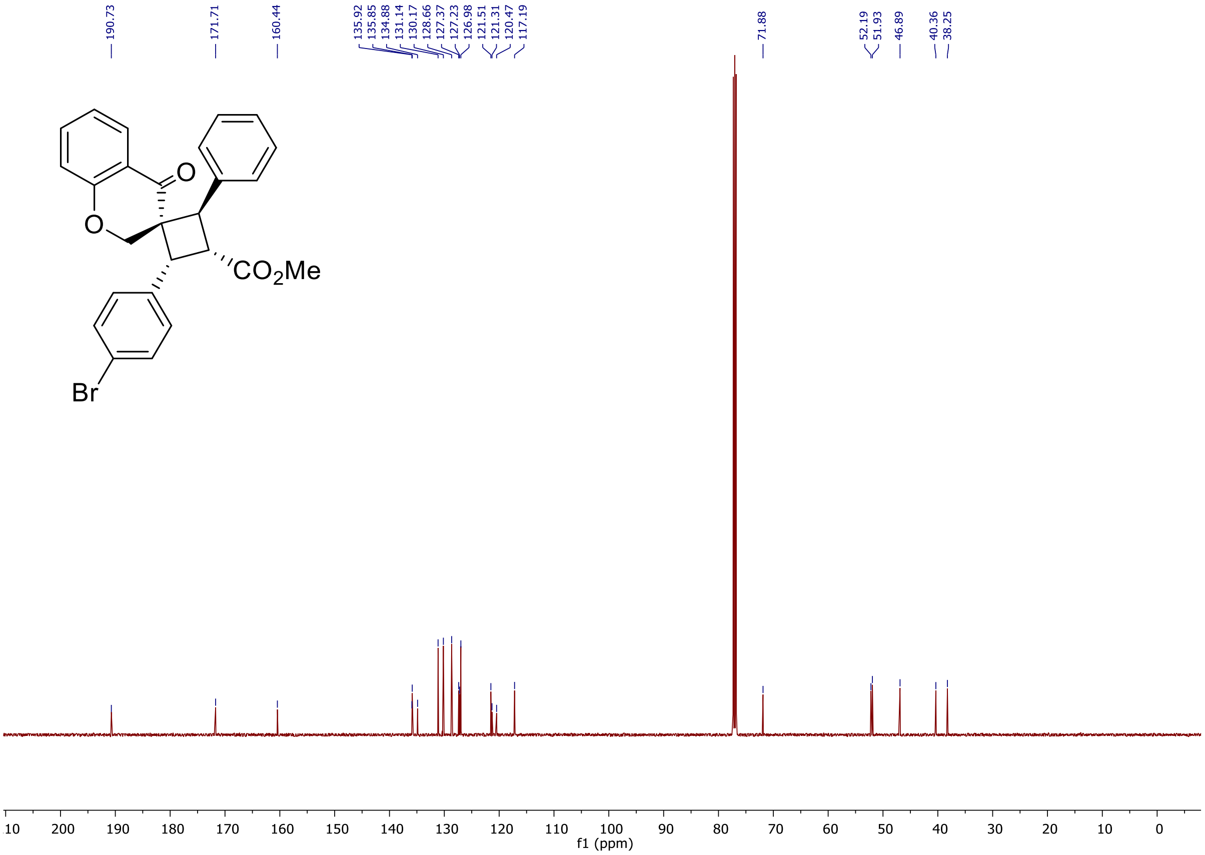

^{1}H NMR of compound 34

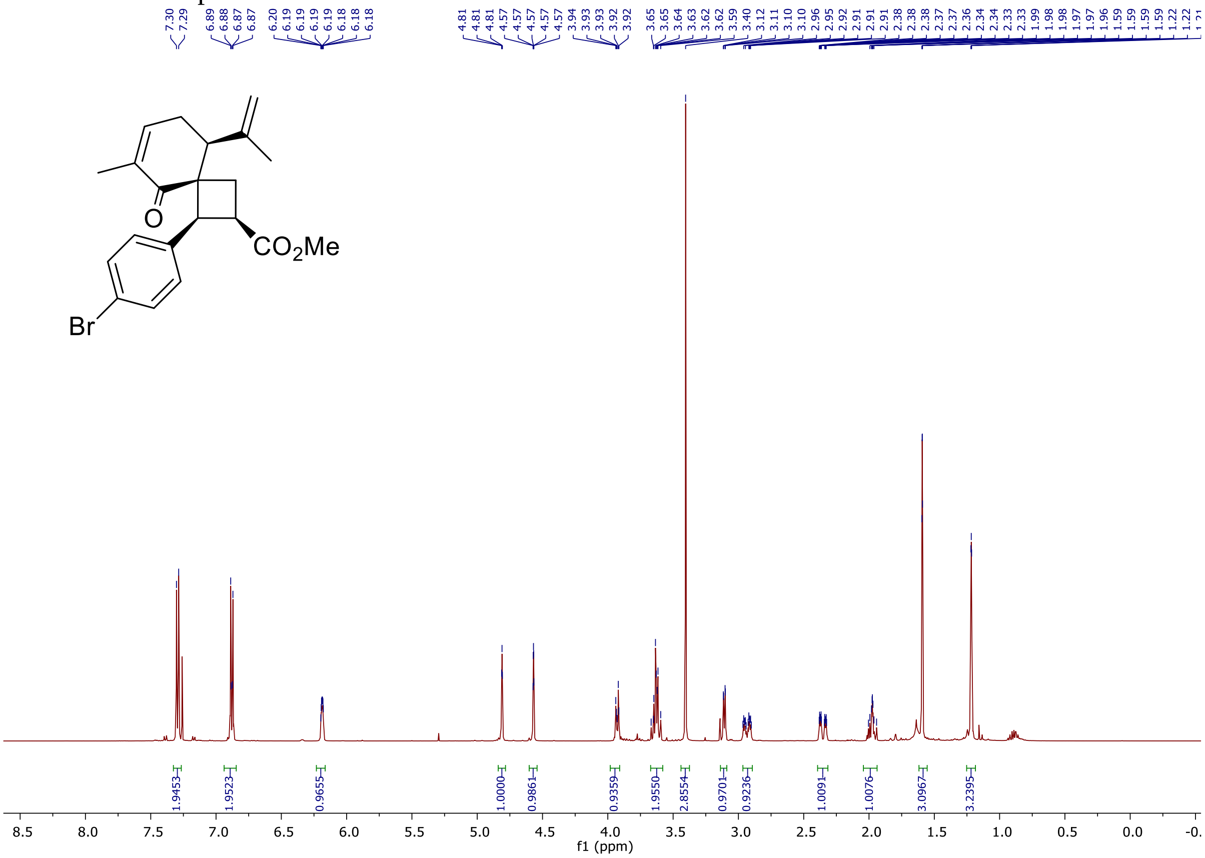

^{13}C NMR of compound 34

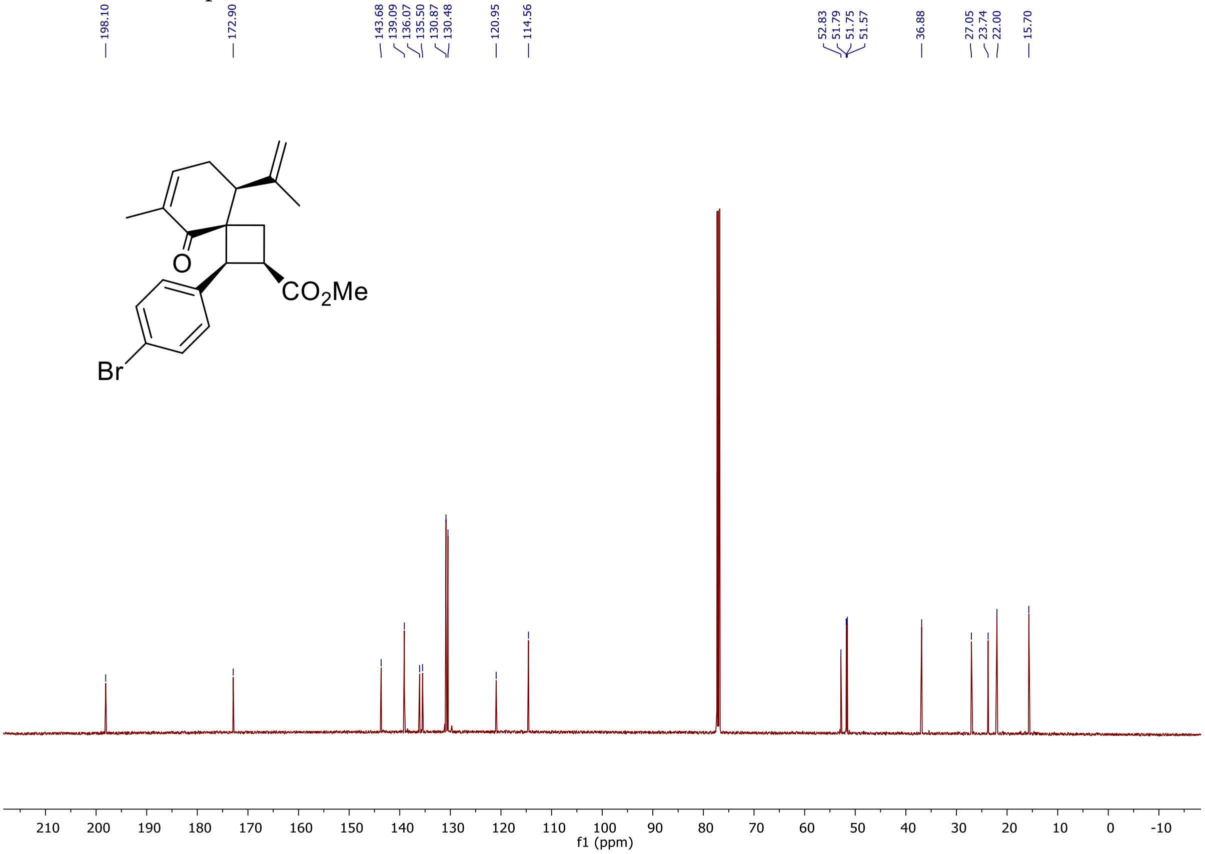

^{1}H NMR of compound 35

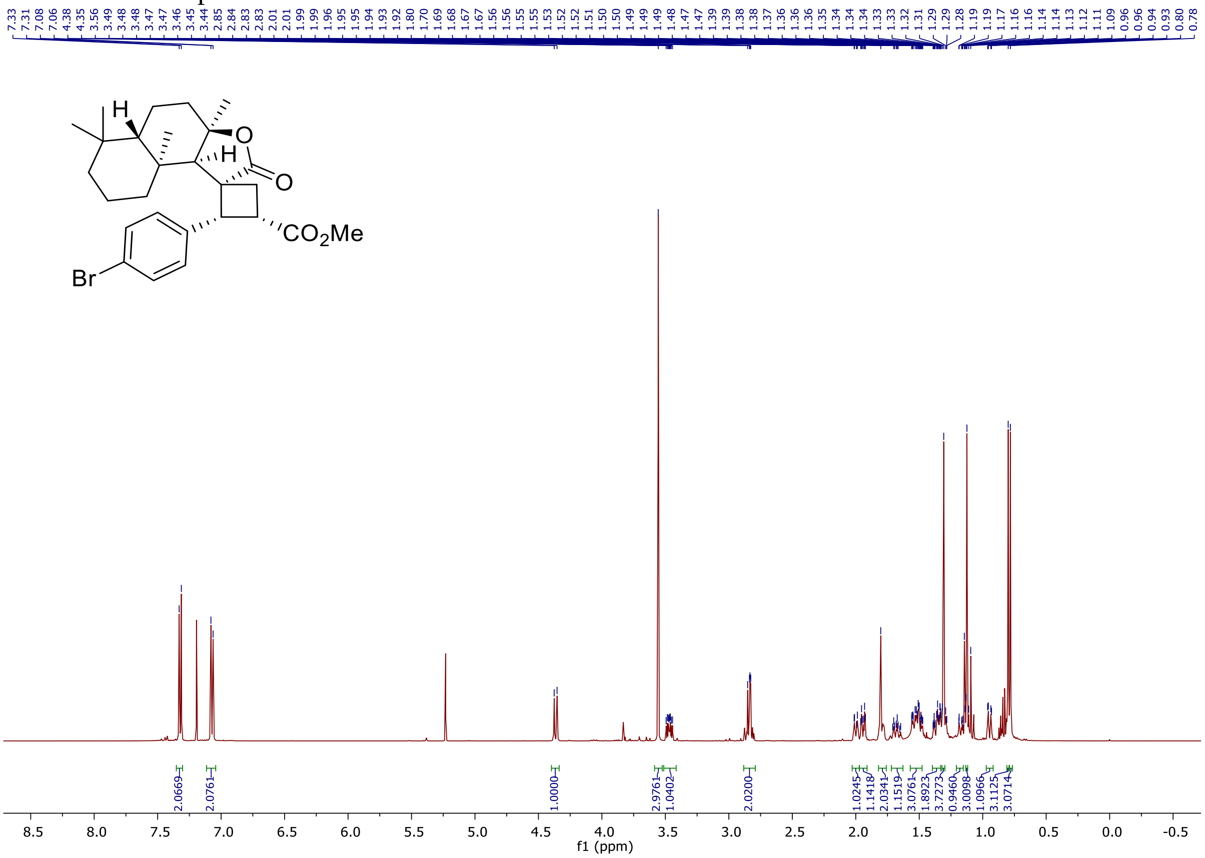

^{13}C NMR of compound 35

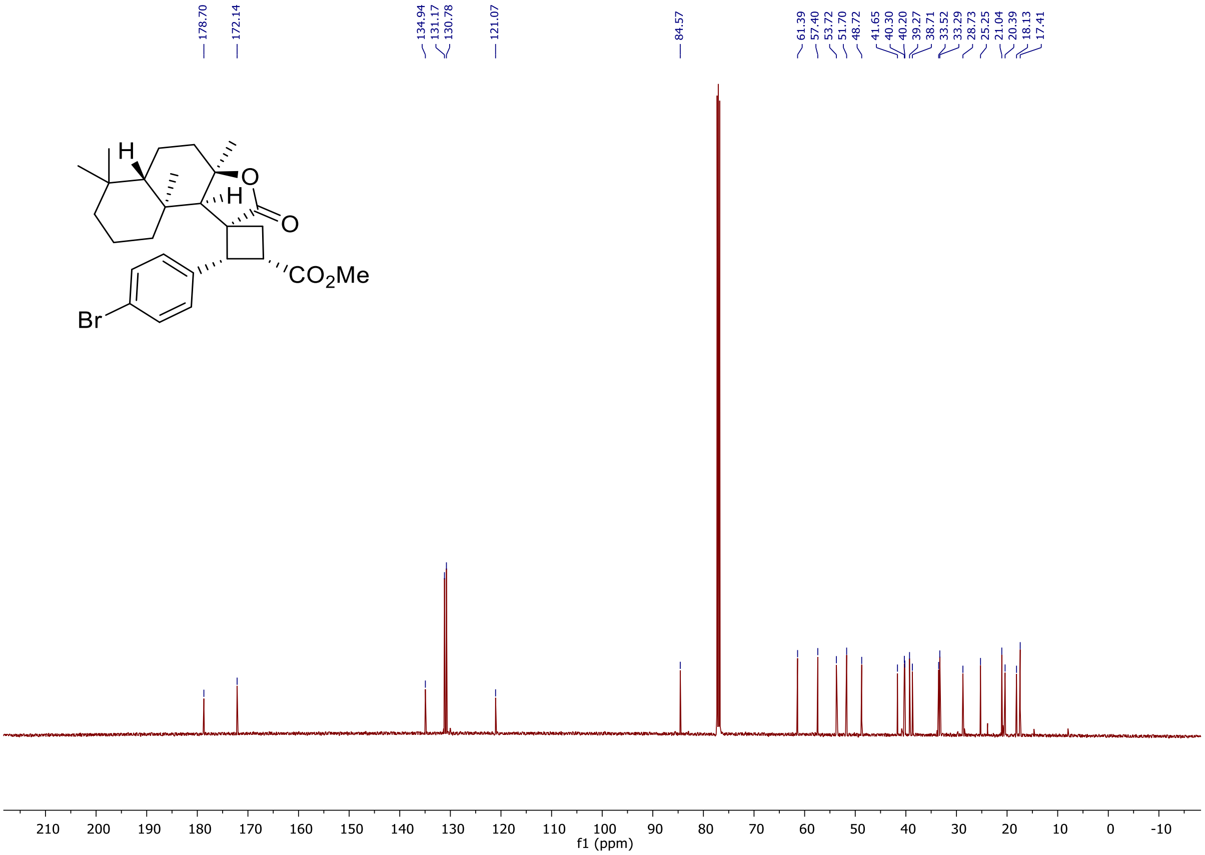

^{1}H NMR of compound 36

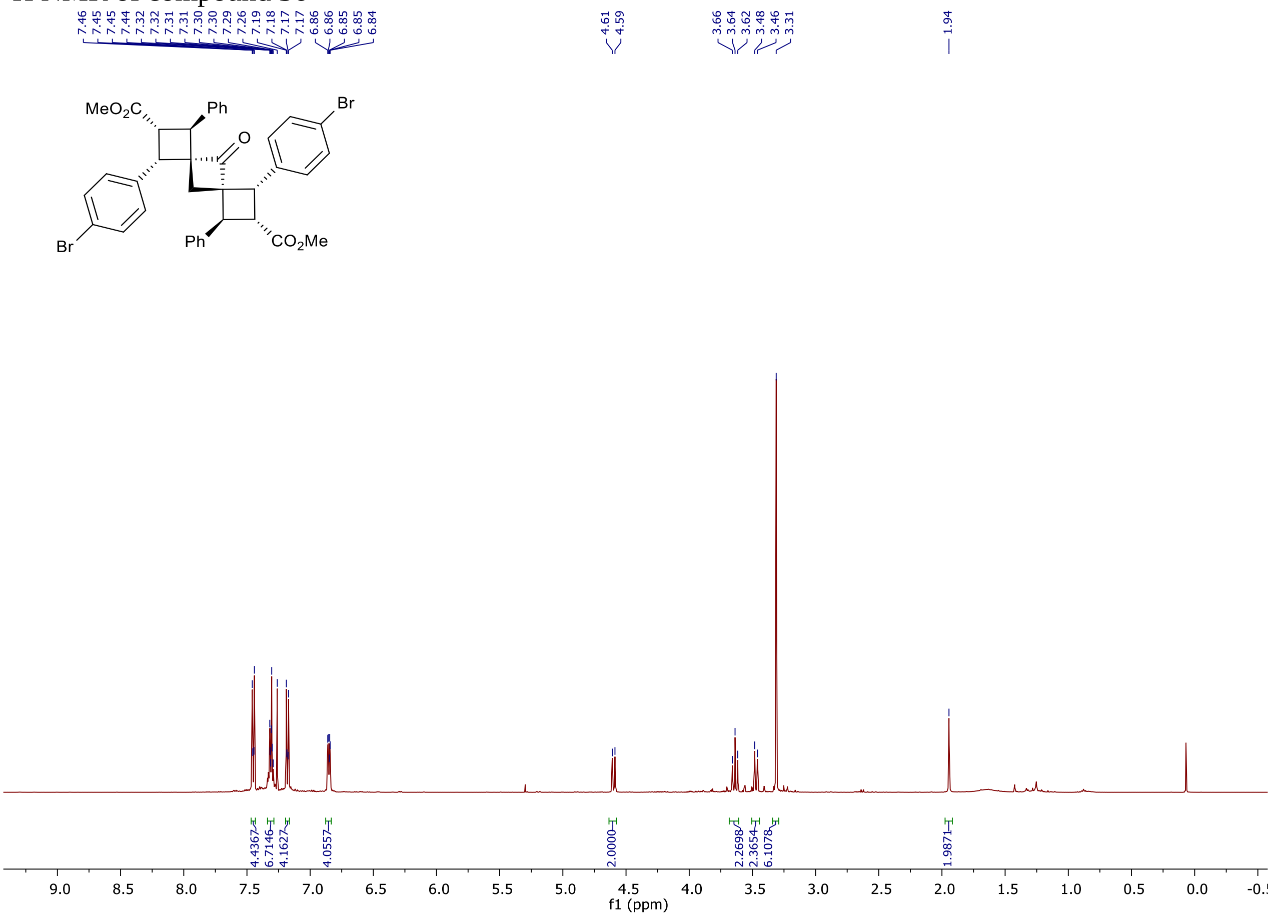

^{13}C NMR of compound 36

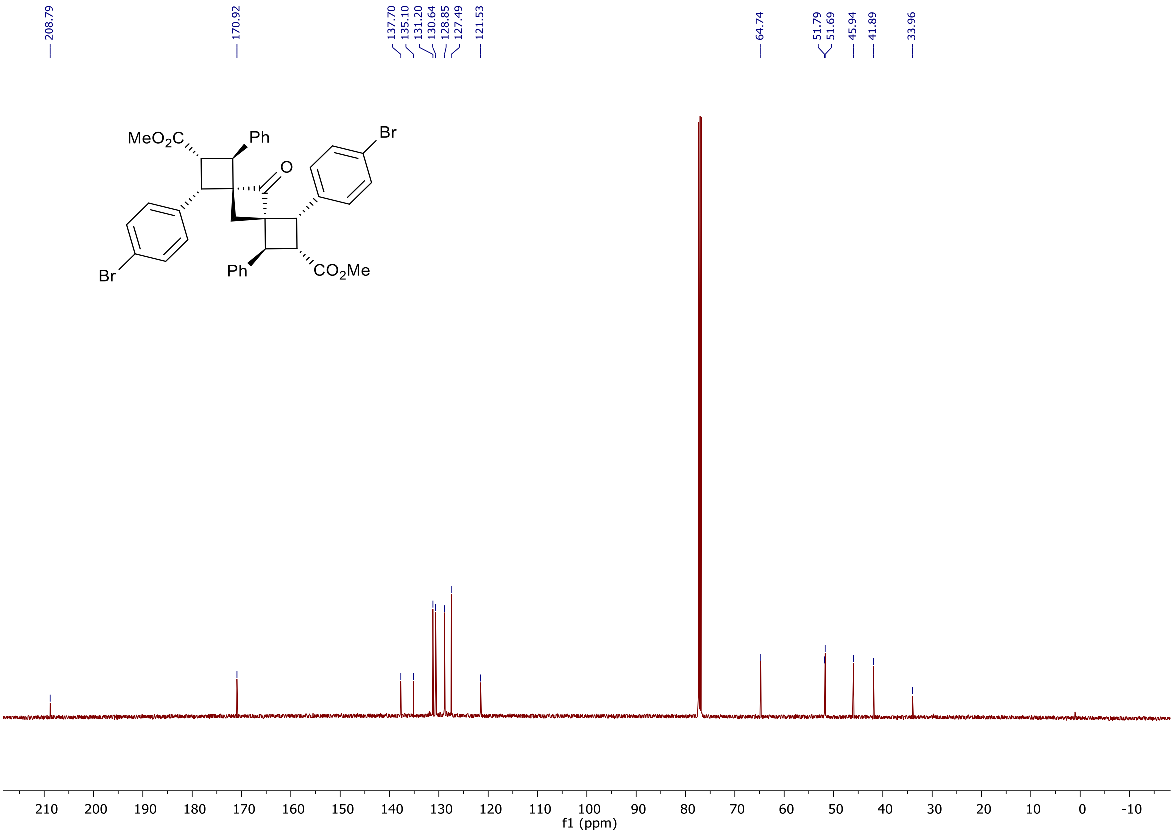

^{1}H NMR of compound 37

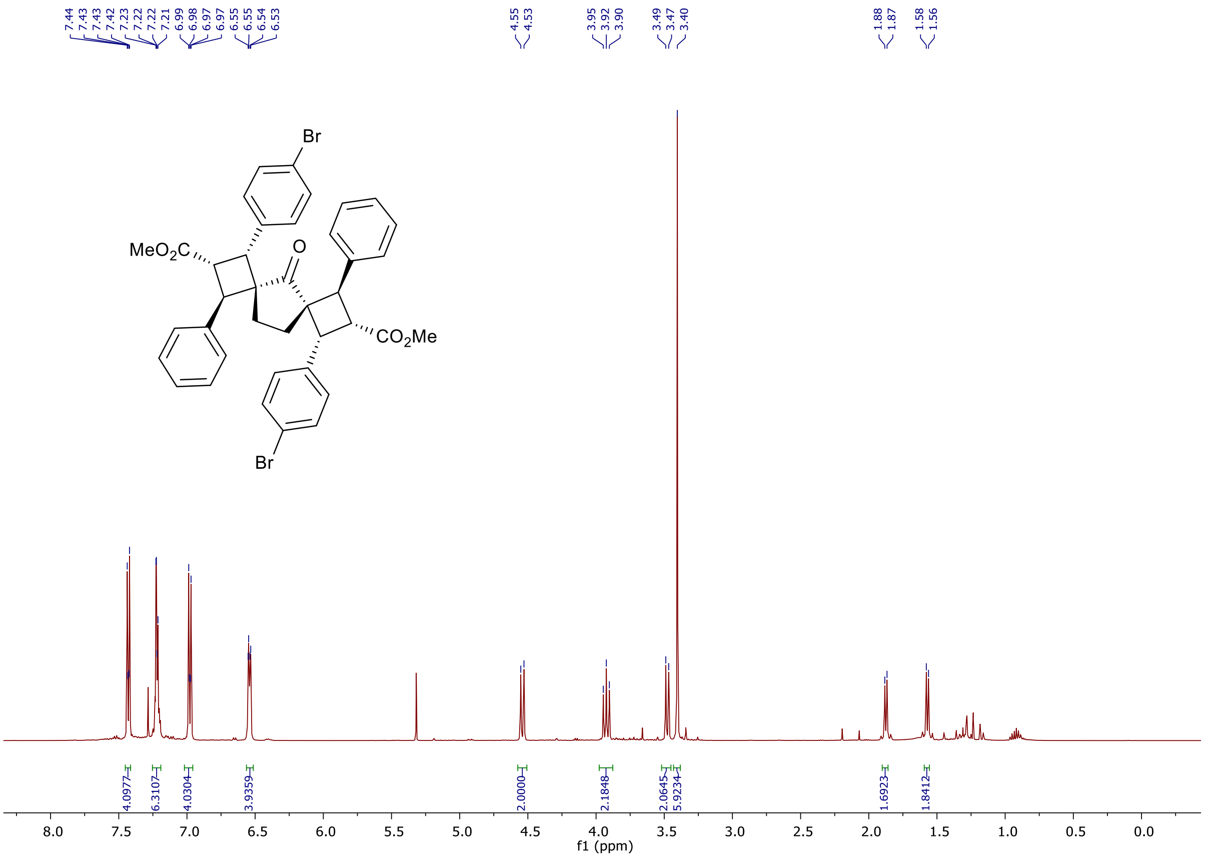

^{13}C NMR of compound 37

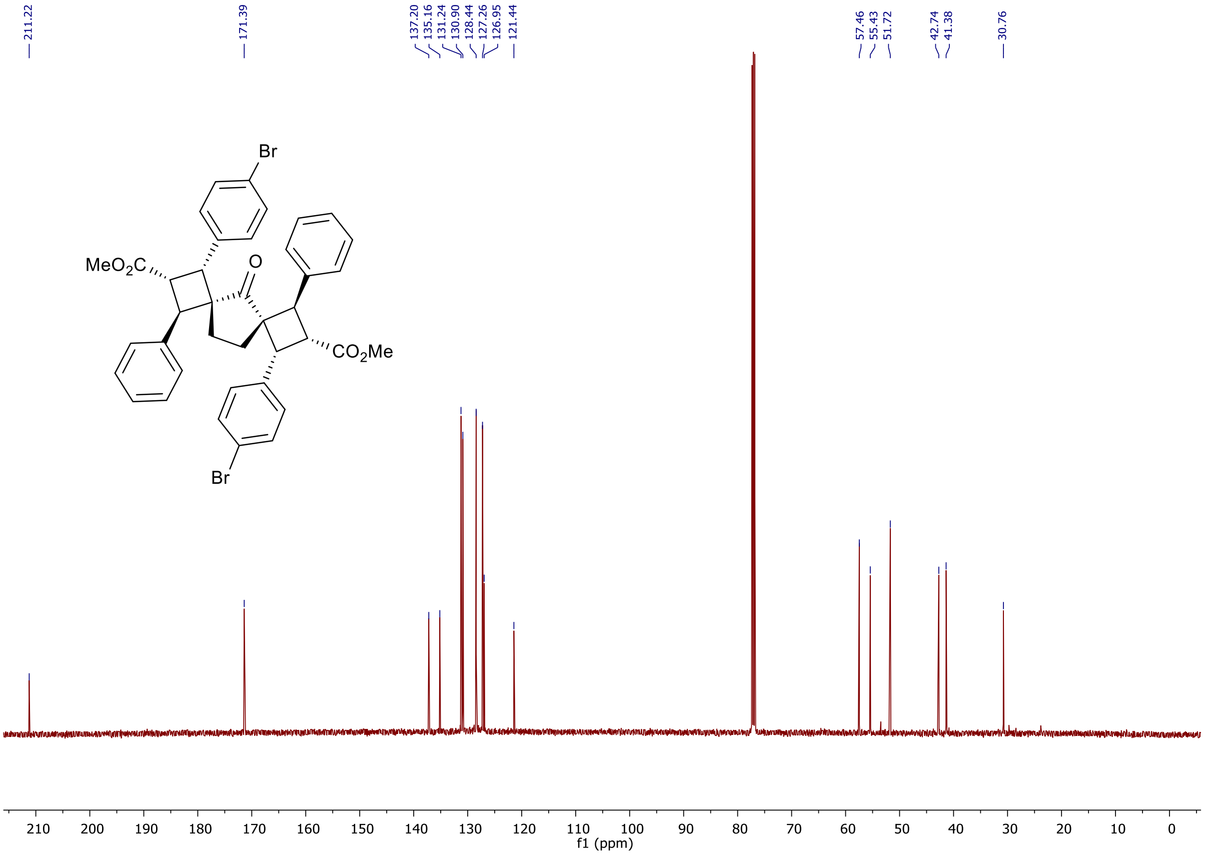

^{1}H NMR of compound 44

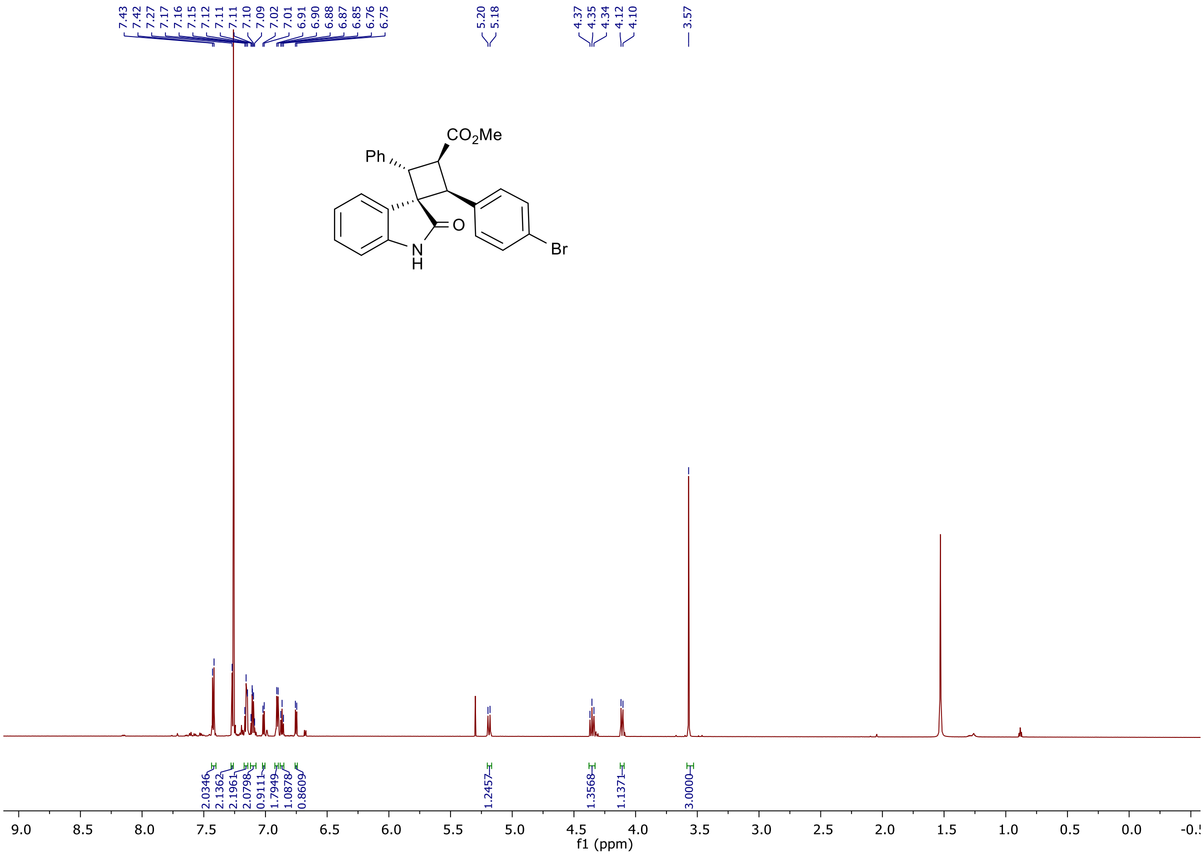

^{13}C NMR of compound 44

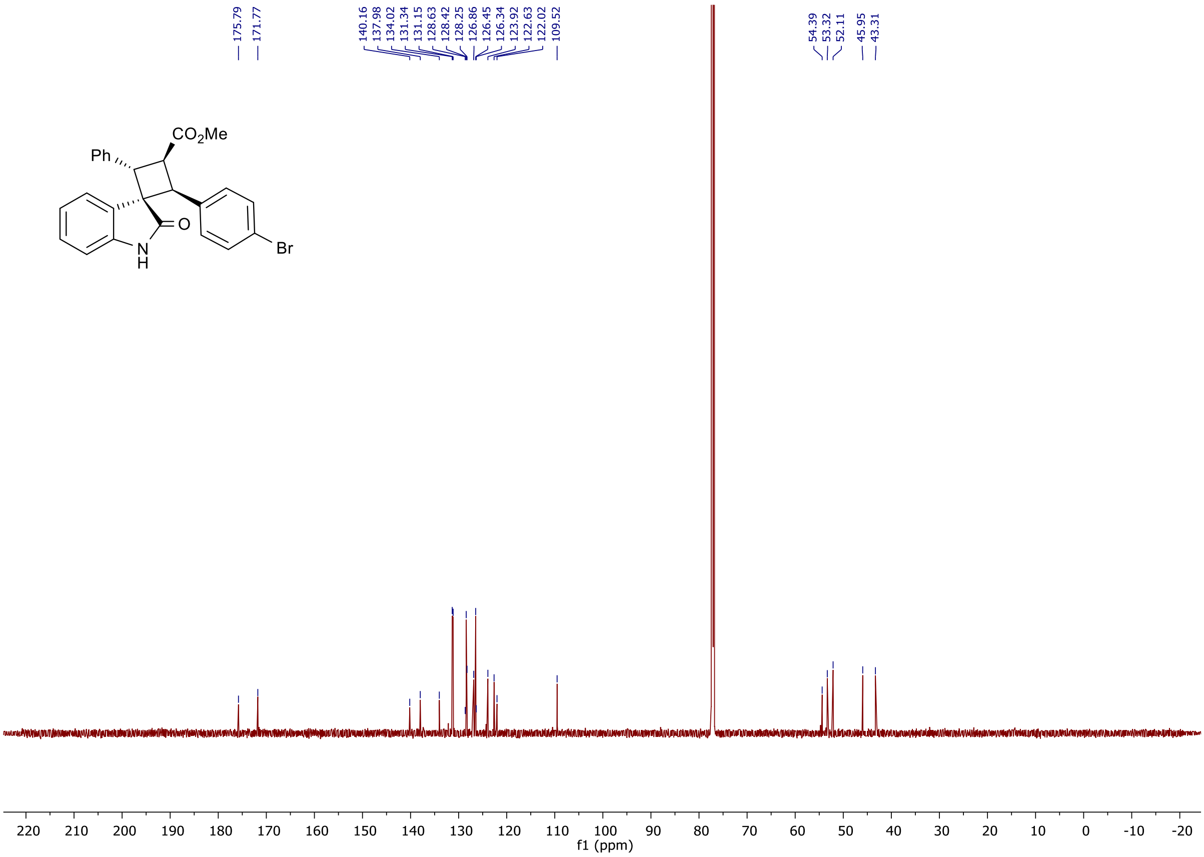

^{1}H NMR of compound *cis*-46

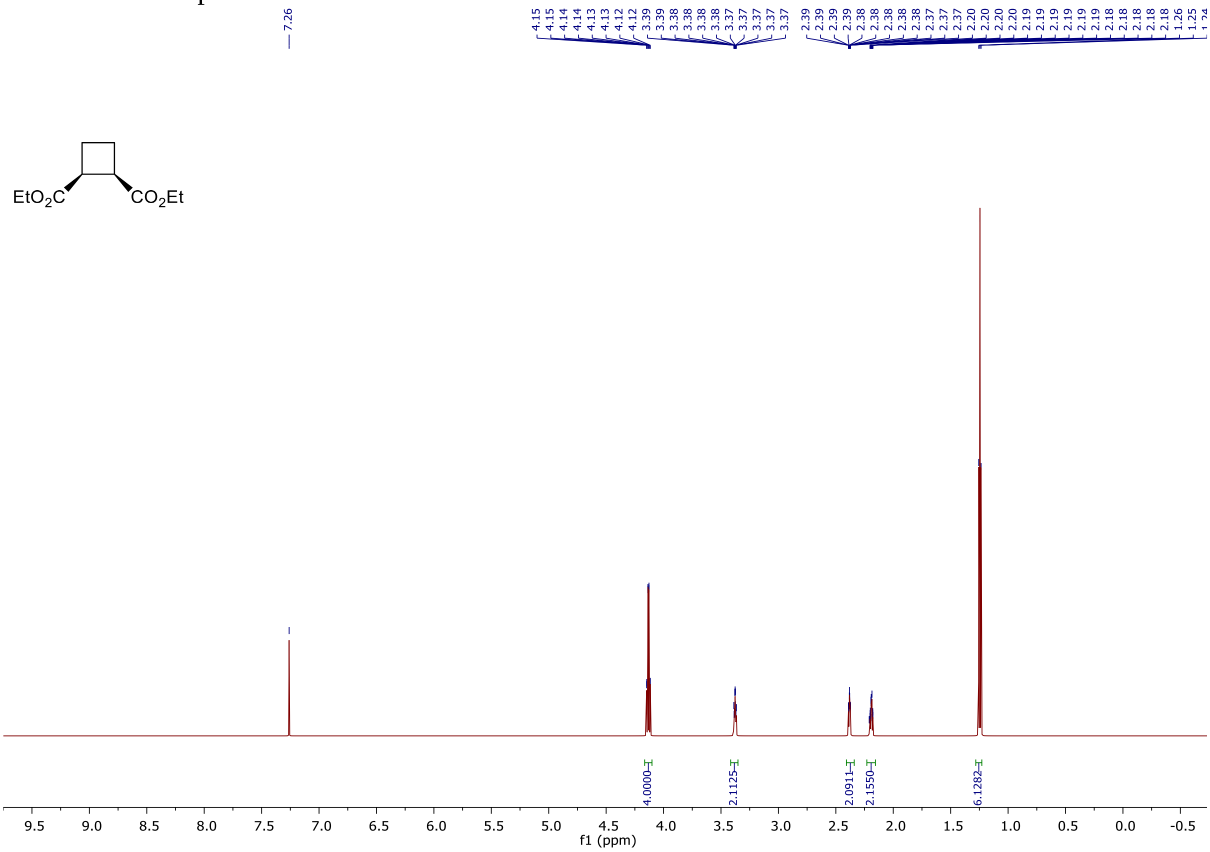

^{13}C NMR of *cis*-46

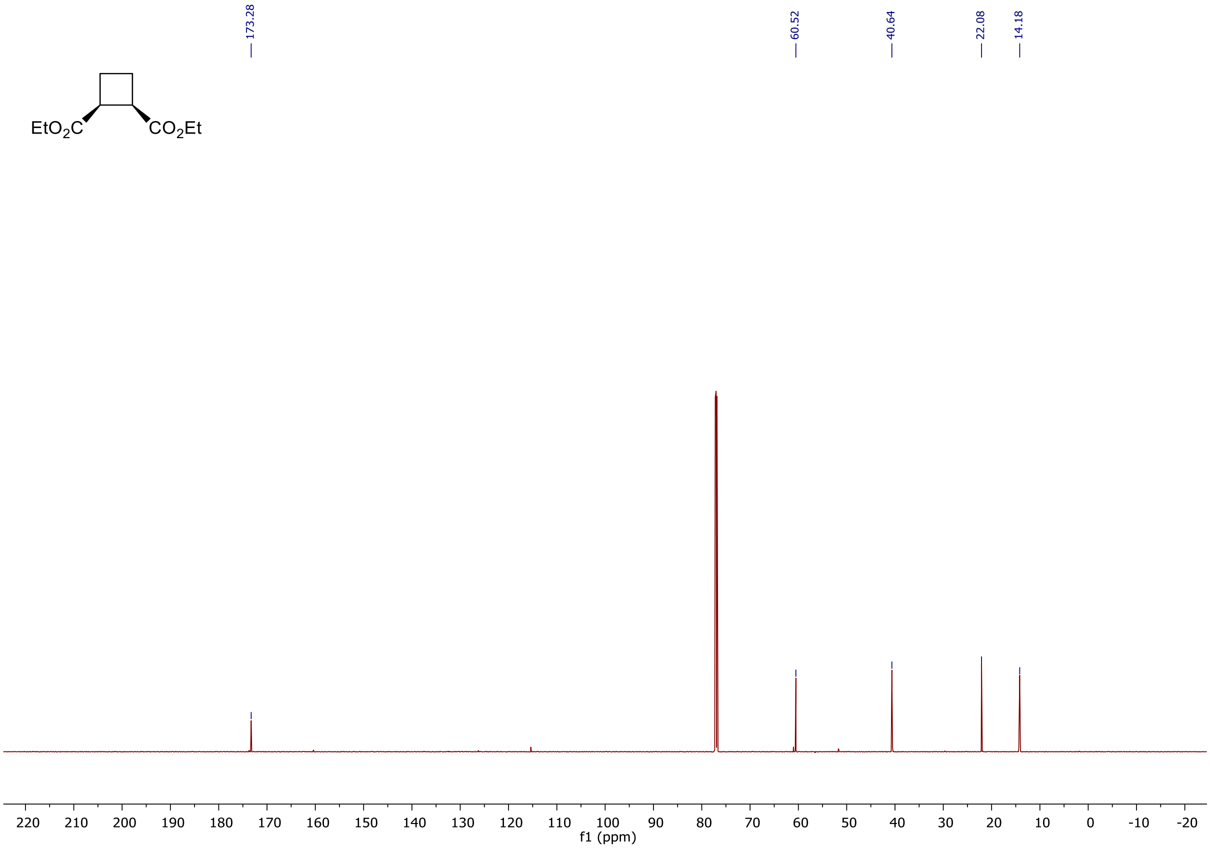

1H NMR of compound *cis*-48

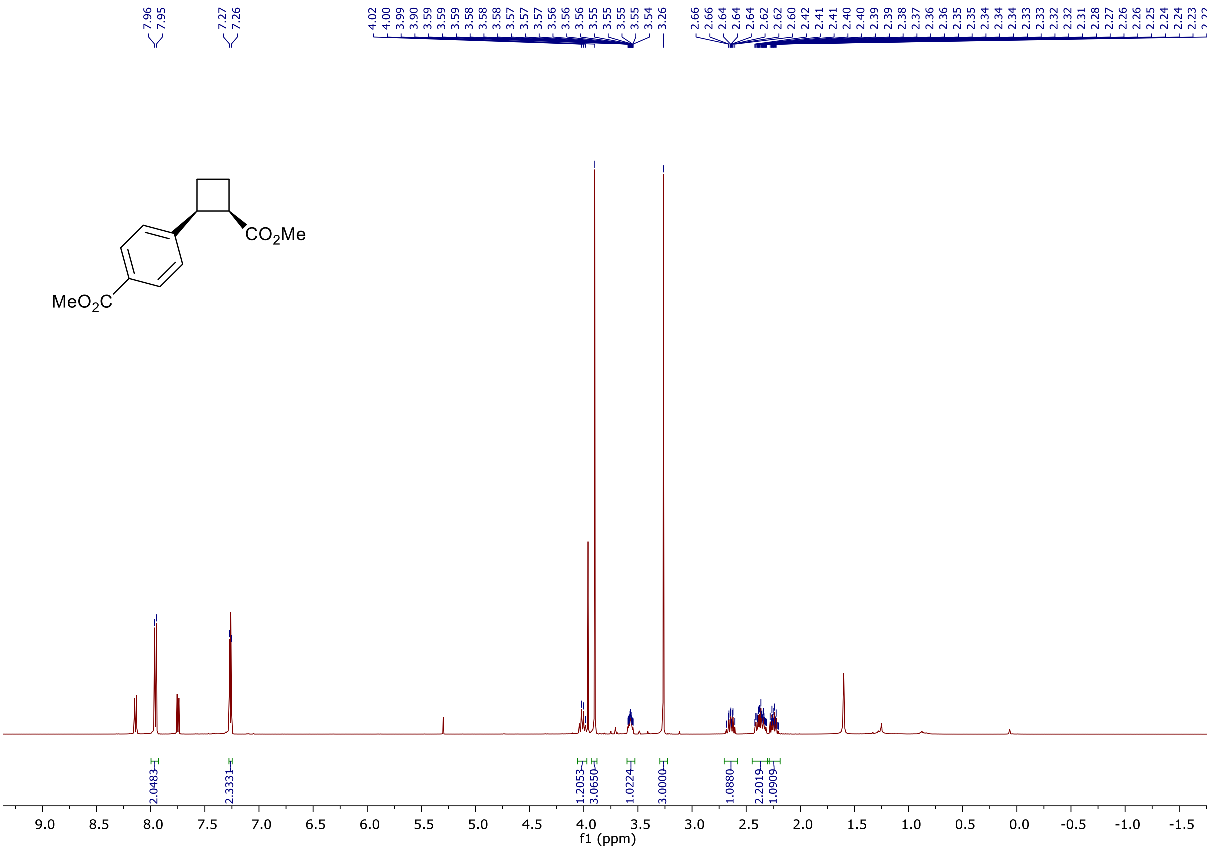

13C of compound *cis*-48

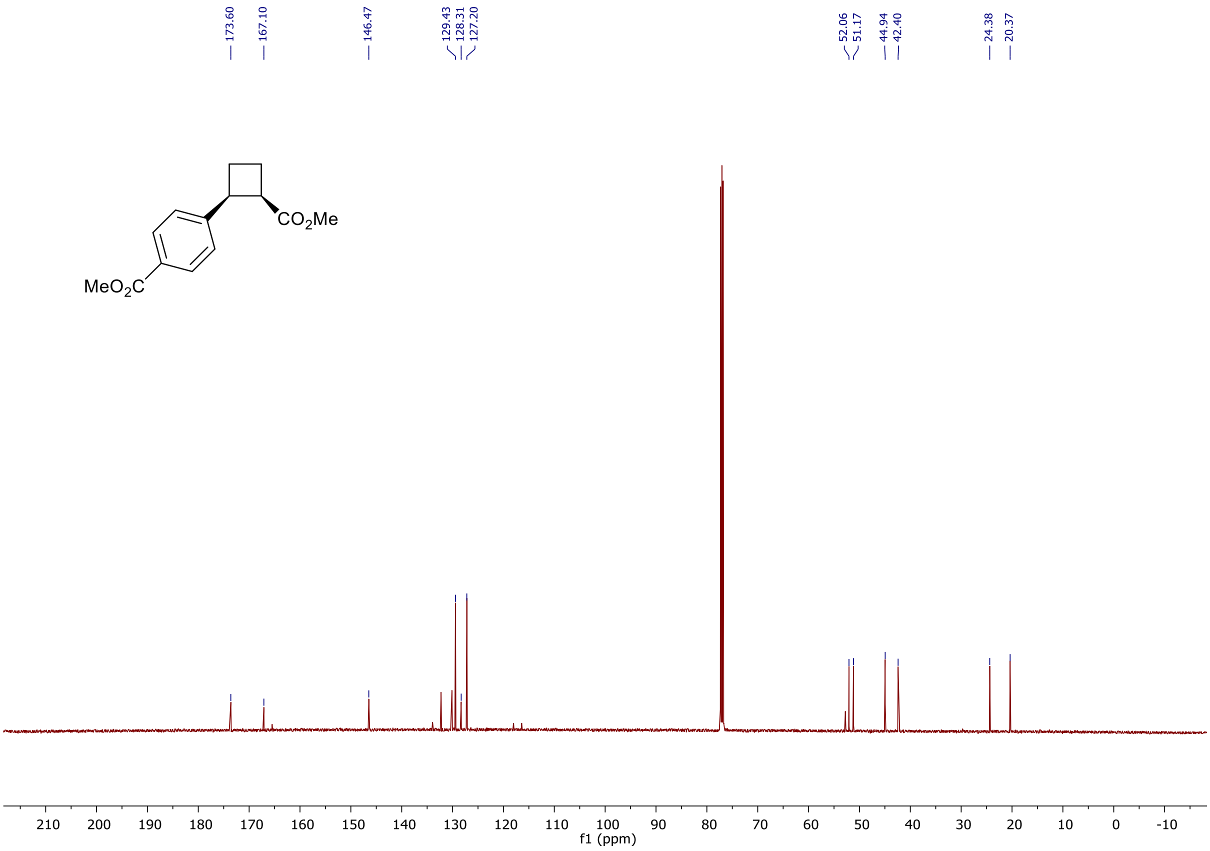

1H NMR of compound *trans*-50

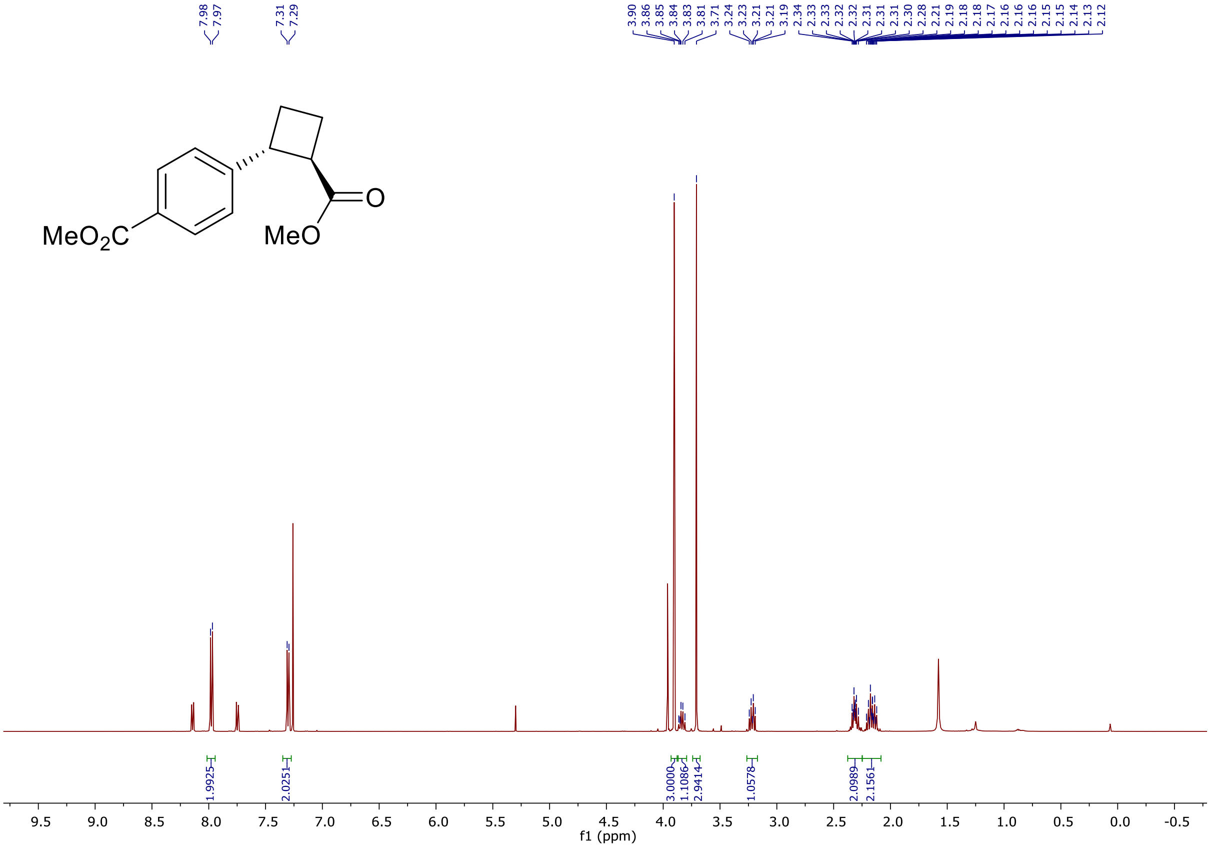

13C NMR of compound *trans*-50

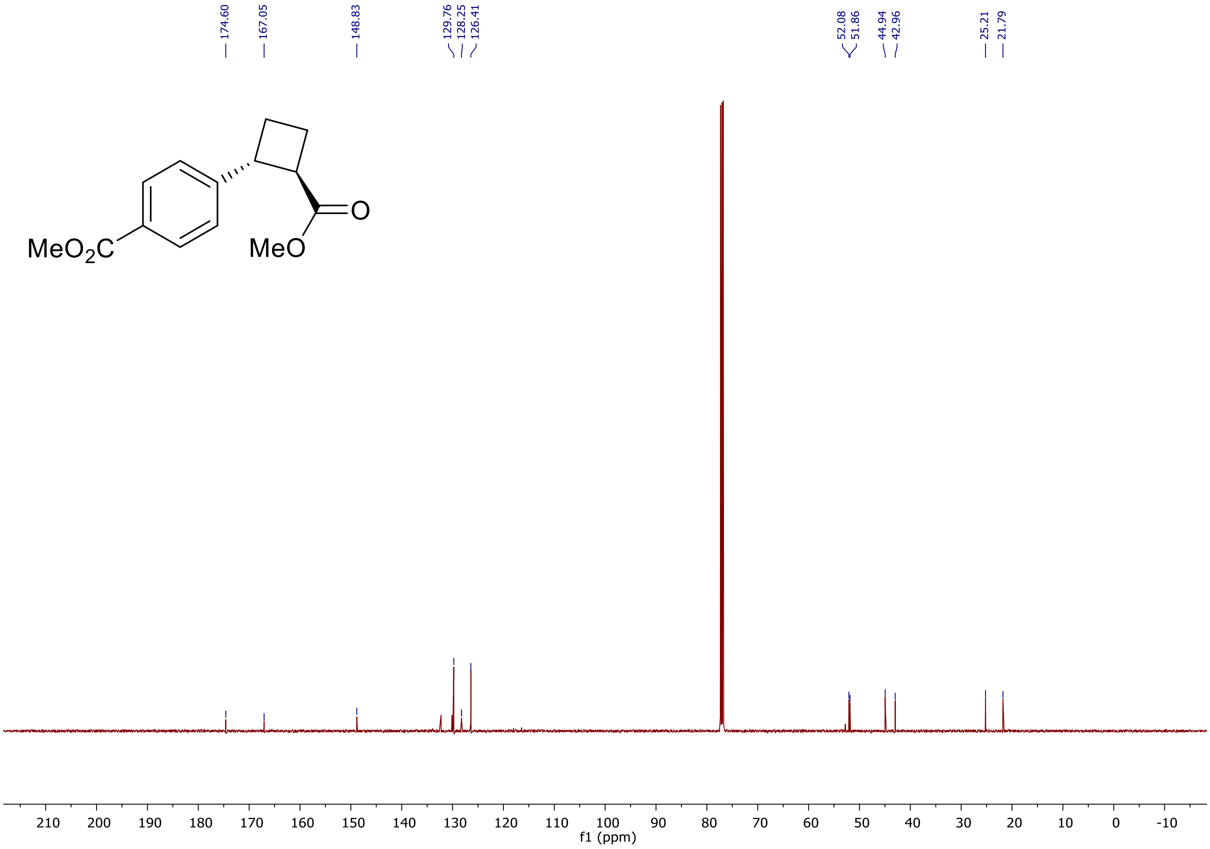

^{1}H NMR of compound 52

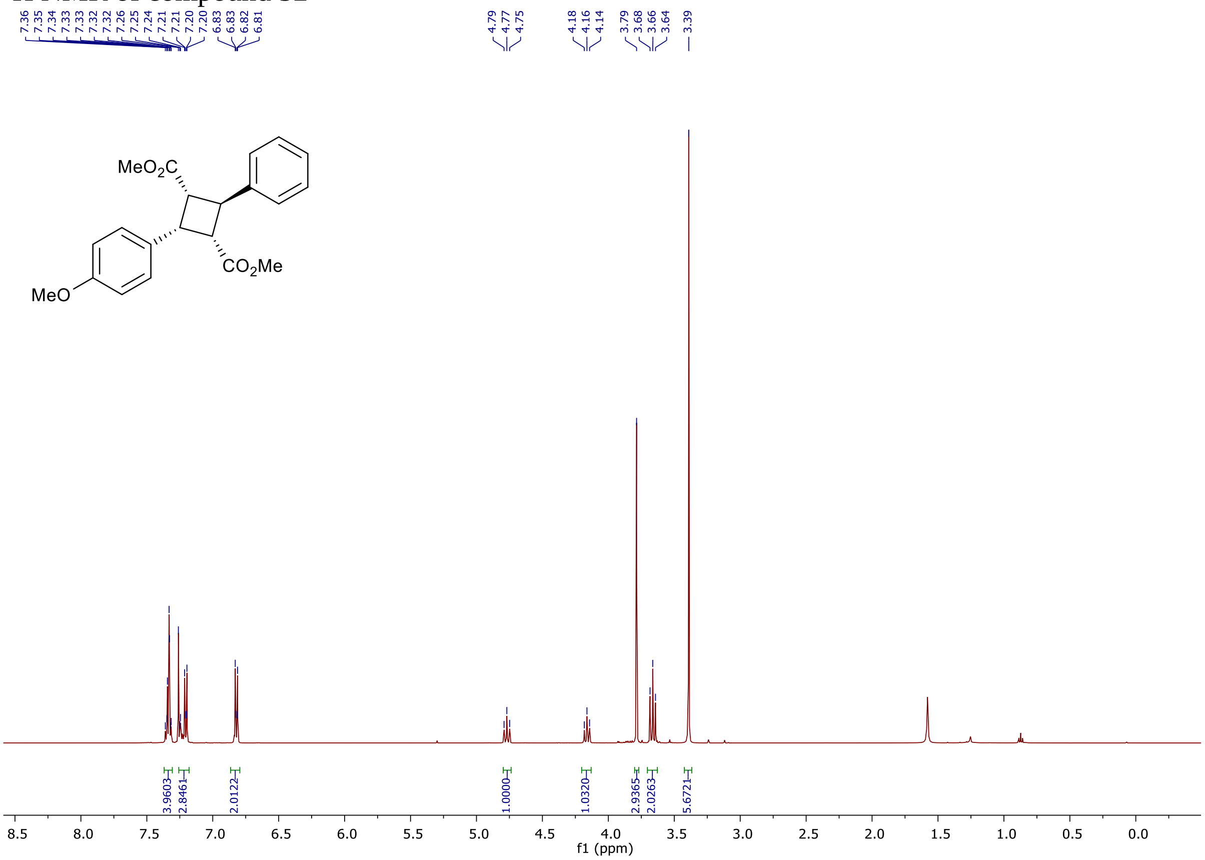

^{13}C NMR of compound 52

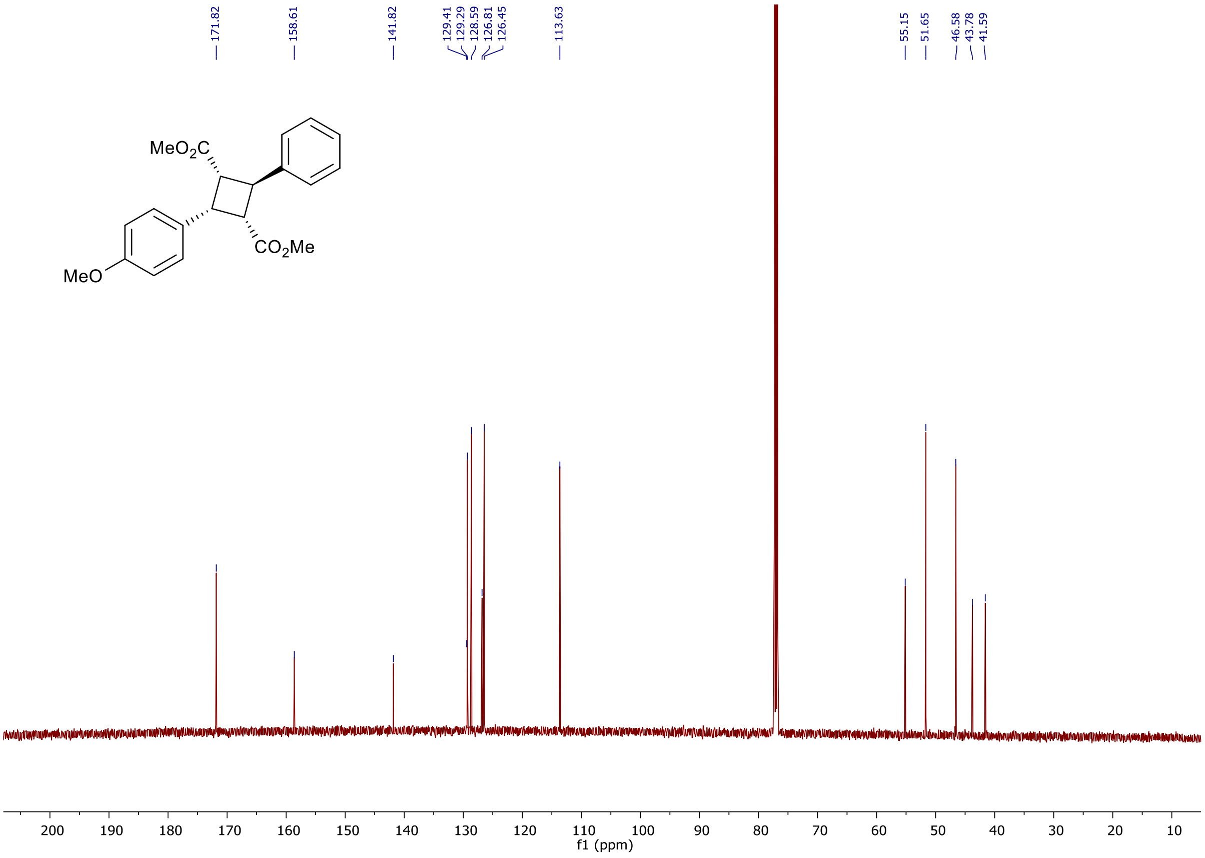

Spectra for formal synthesis of piperarborenine B

^{1}H NMR of compound 60

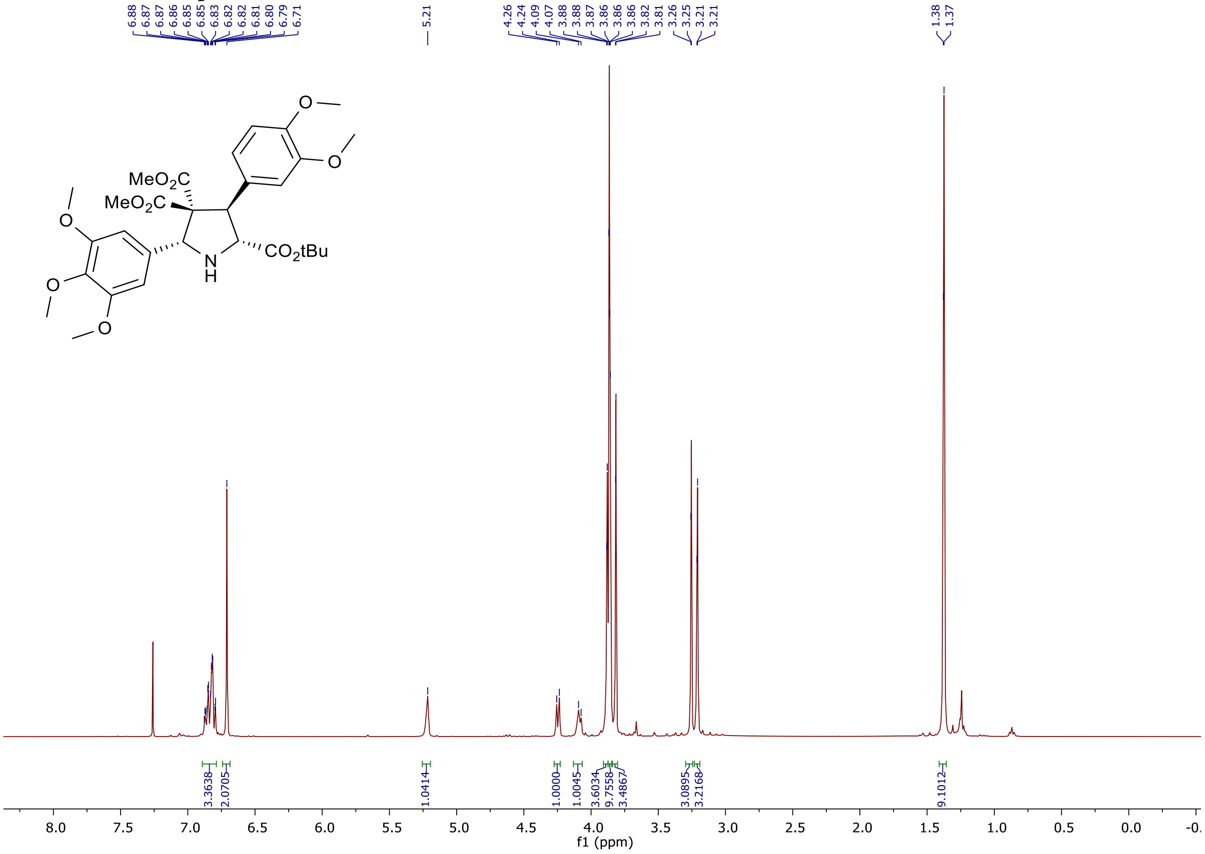

^{13}C NMR of compound 60

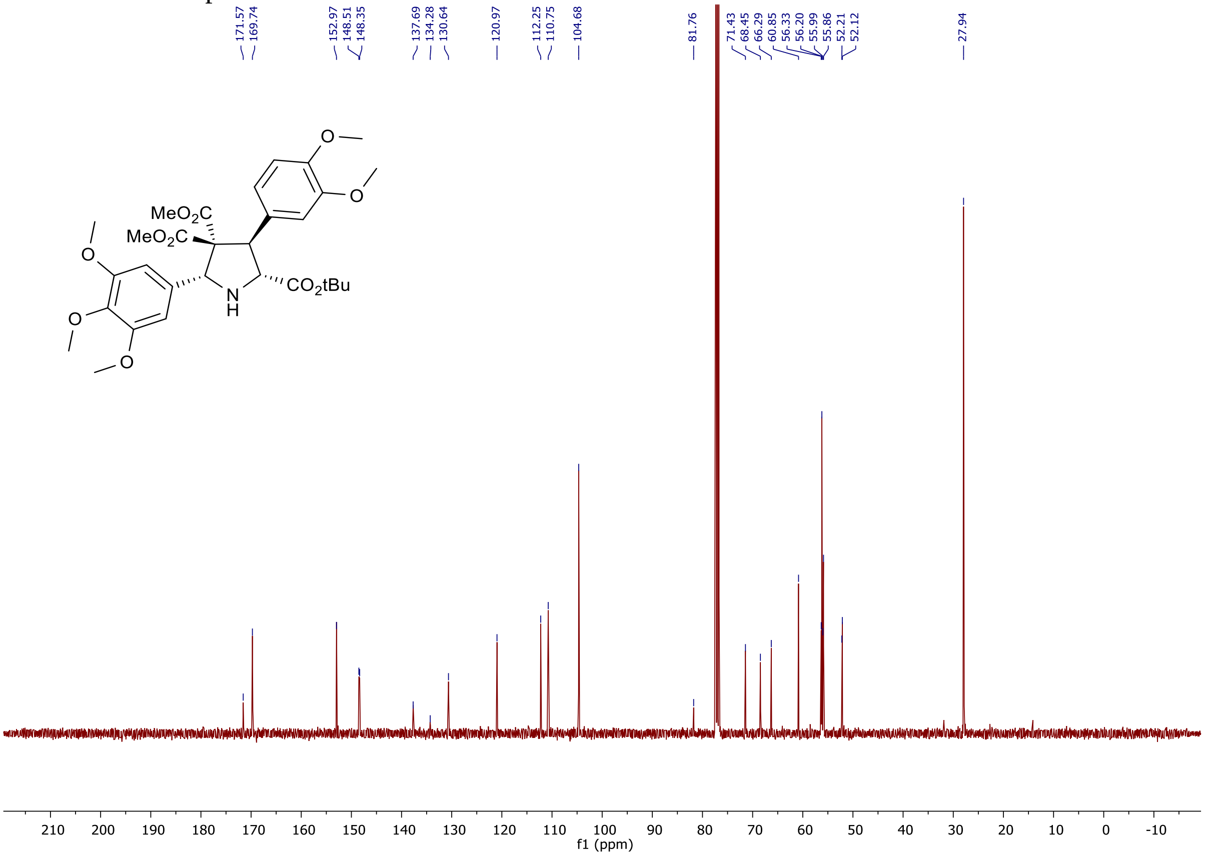

^{1}H NMR of compound 61

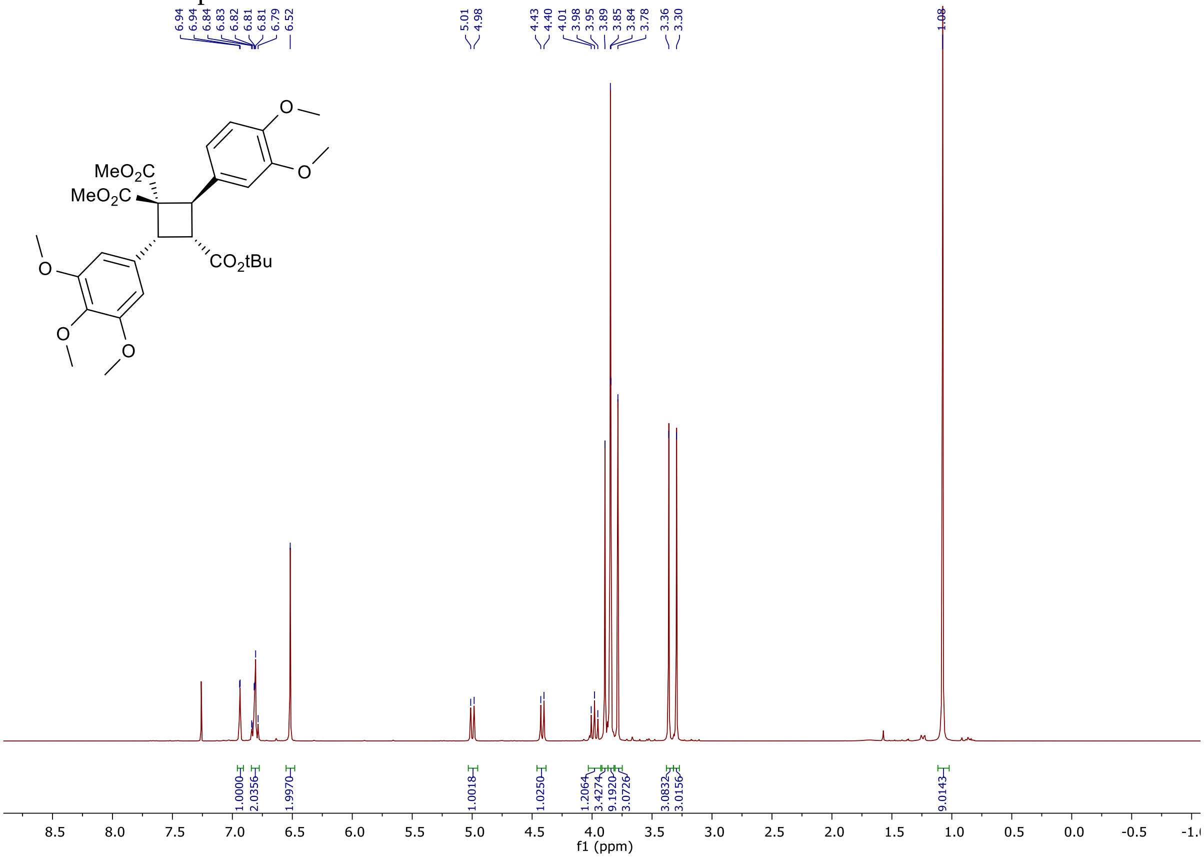

^{13}C NMR of compound 61

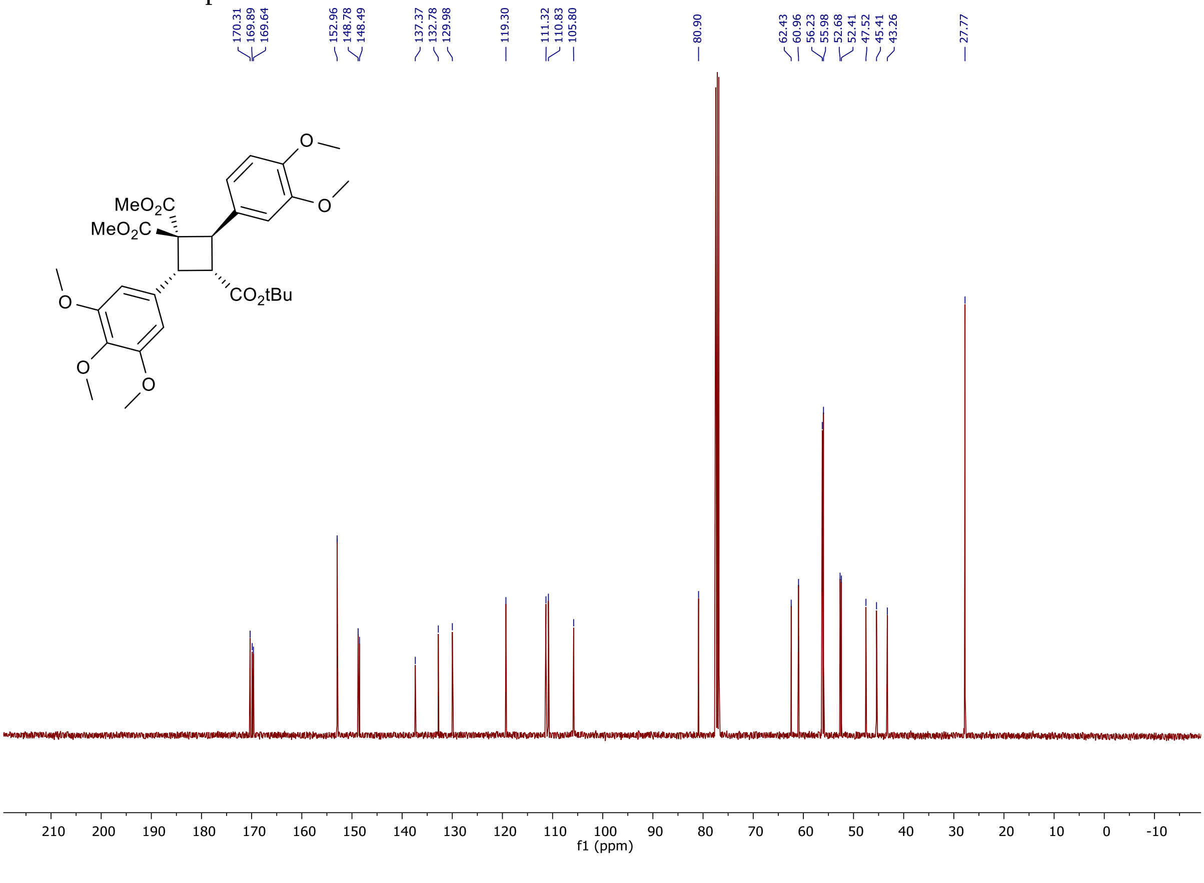

^{1}H NMR of compound 63

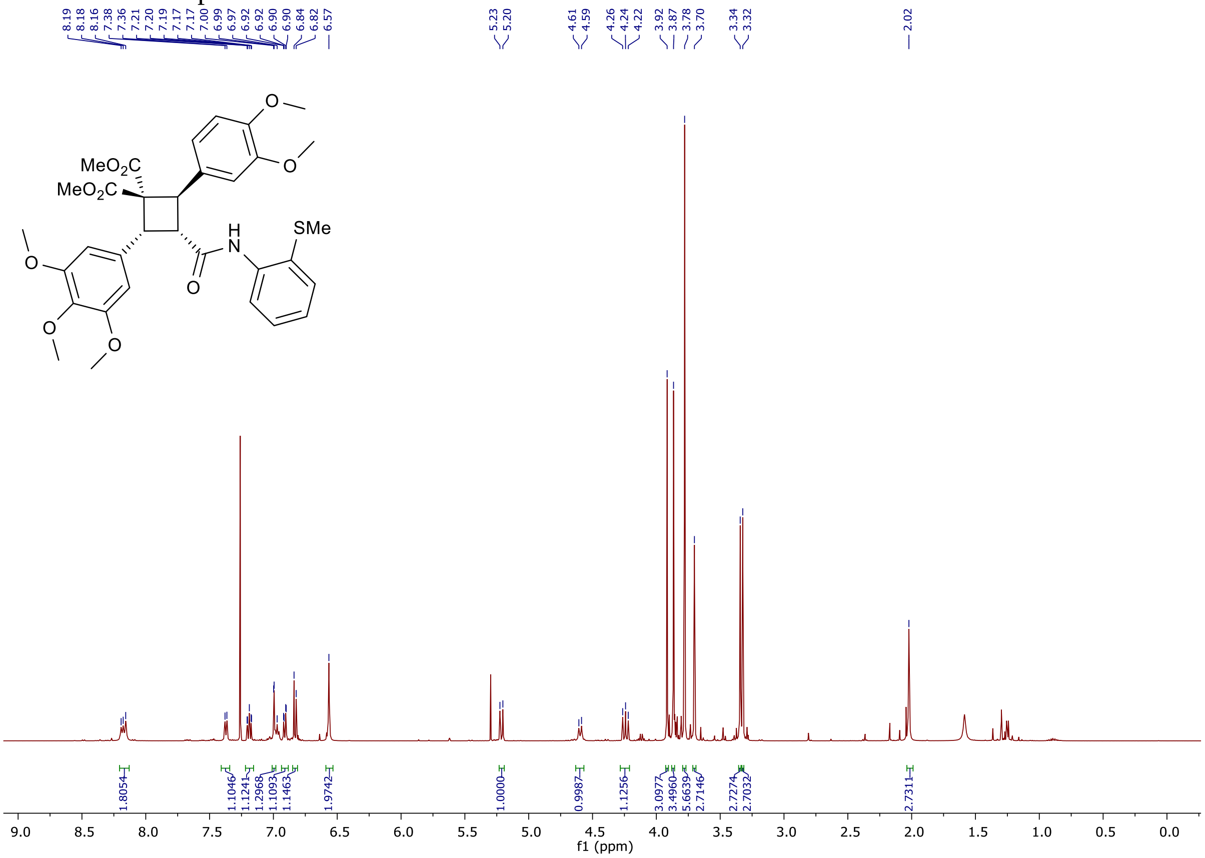

^{13}C NMR of compound 63

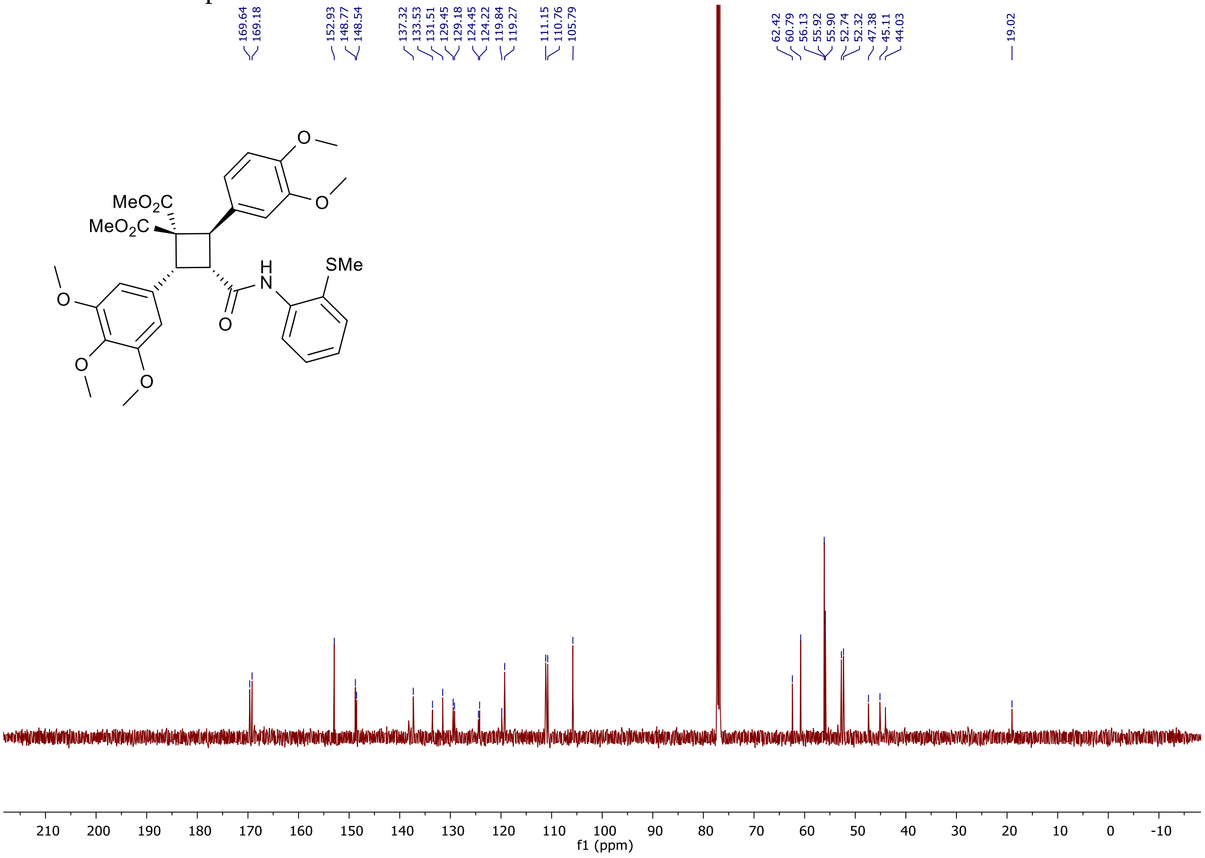

^{1}H NMR of compound 64

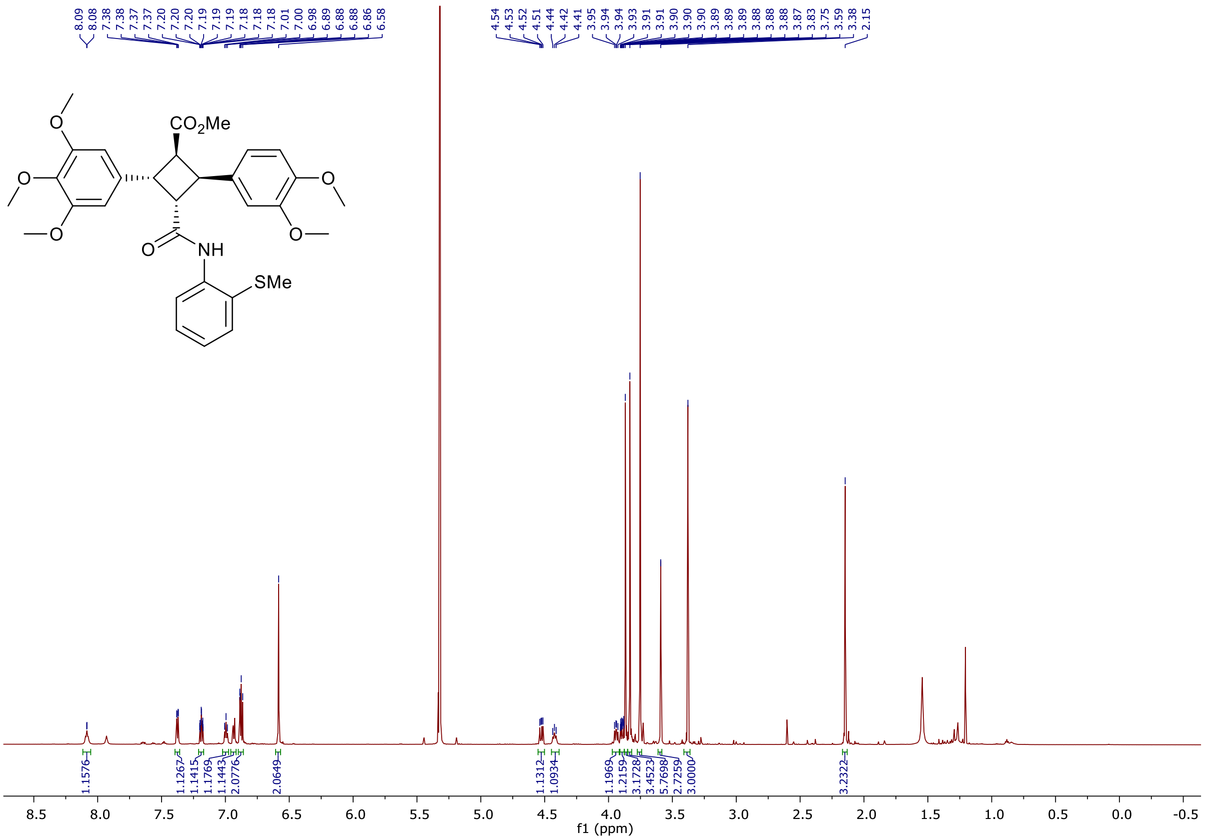

^{13}C NMR of compound 64

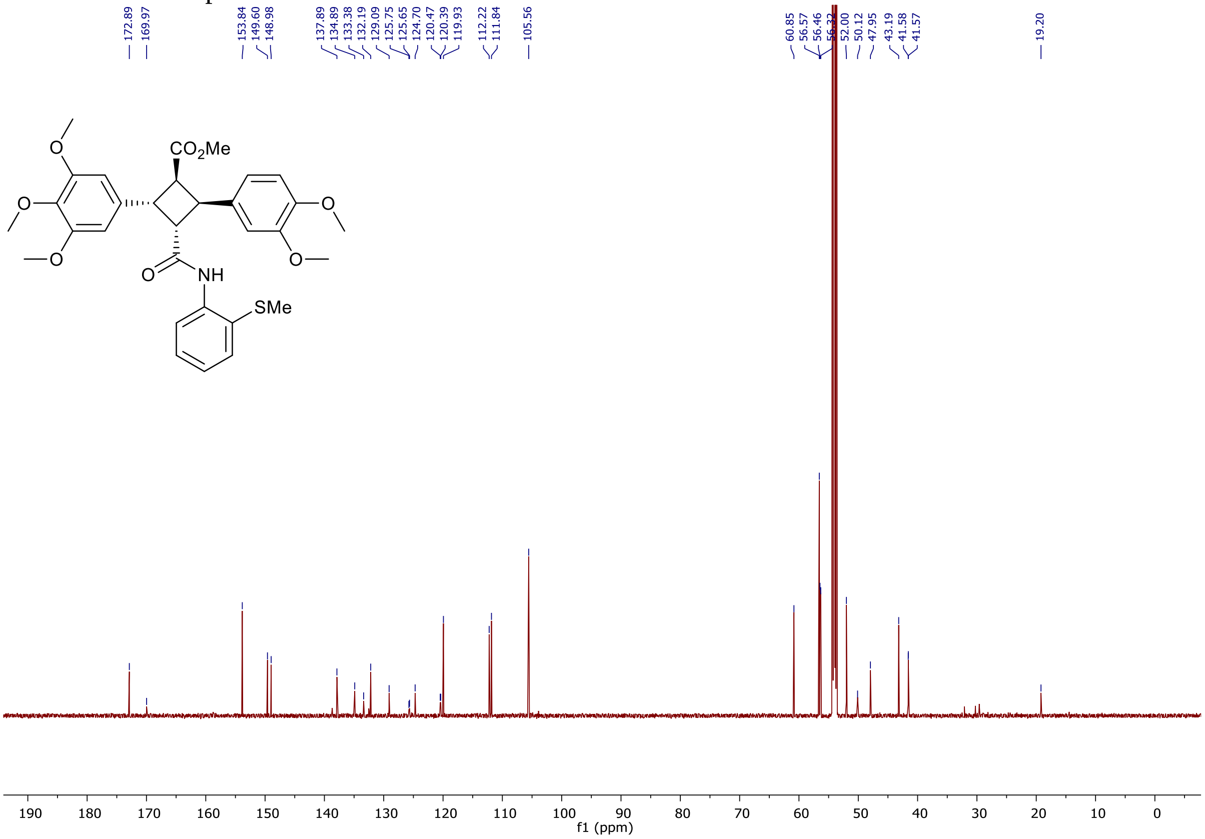

^{1}H NMR of compound E1

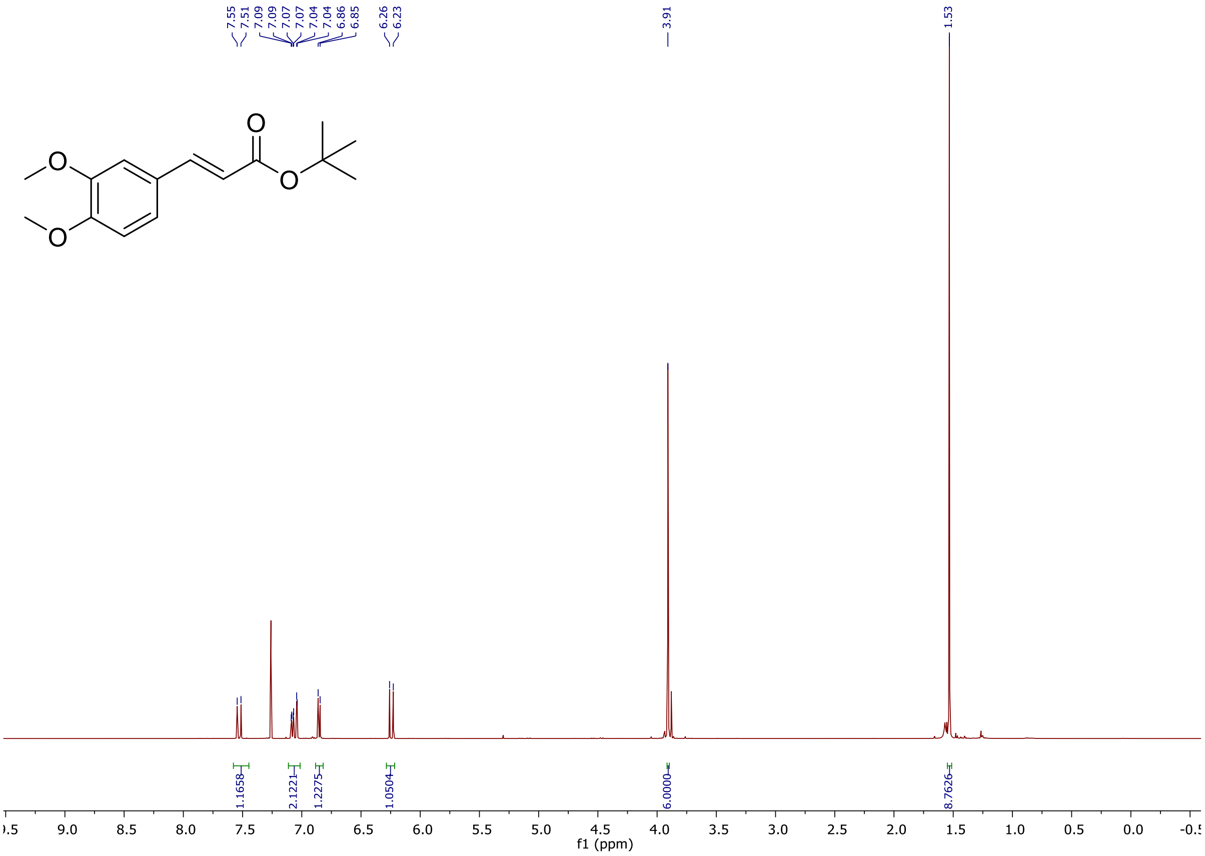

^{13}C NMR of compound E1

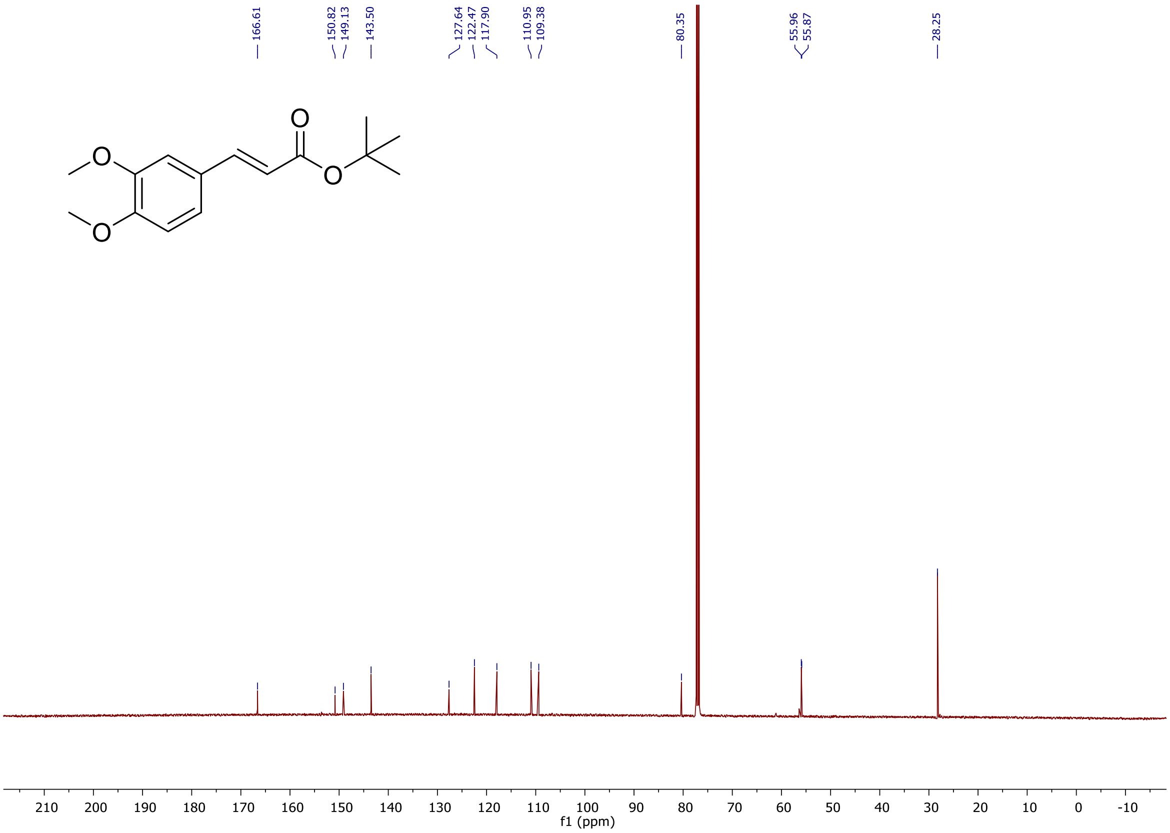

NMR spectra of pyrrolidines

^{1}H NMR of Compound 4

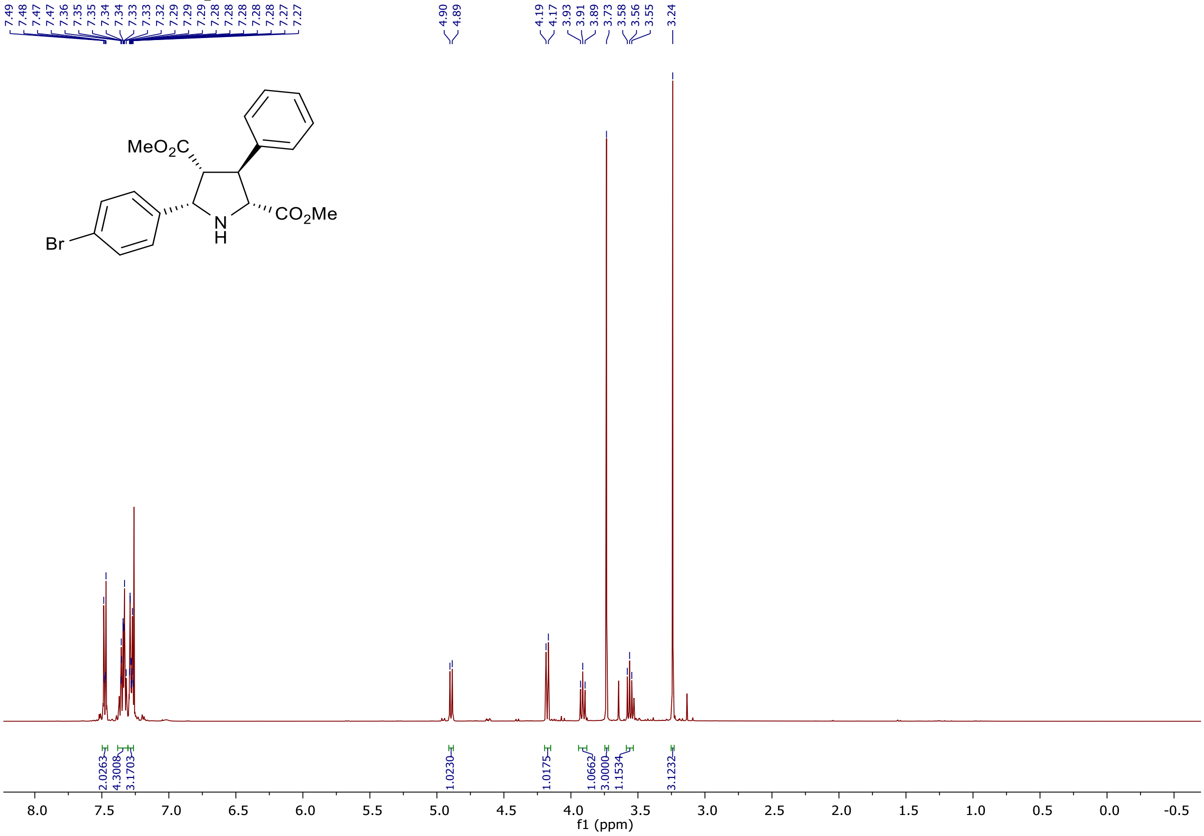

^{13}C NMR of compound 4

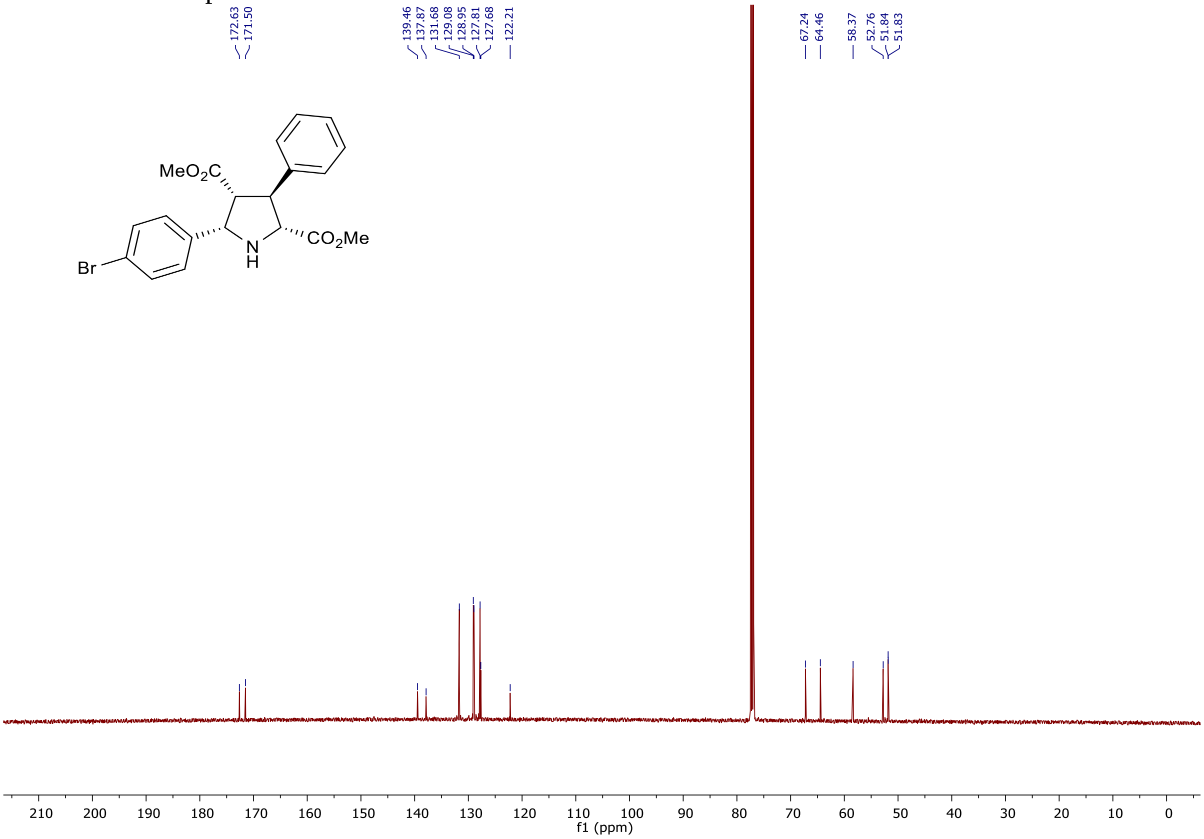

^{1}H NMR of compound A1

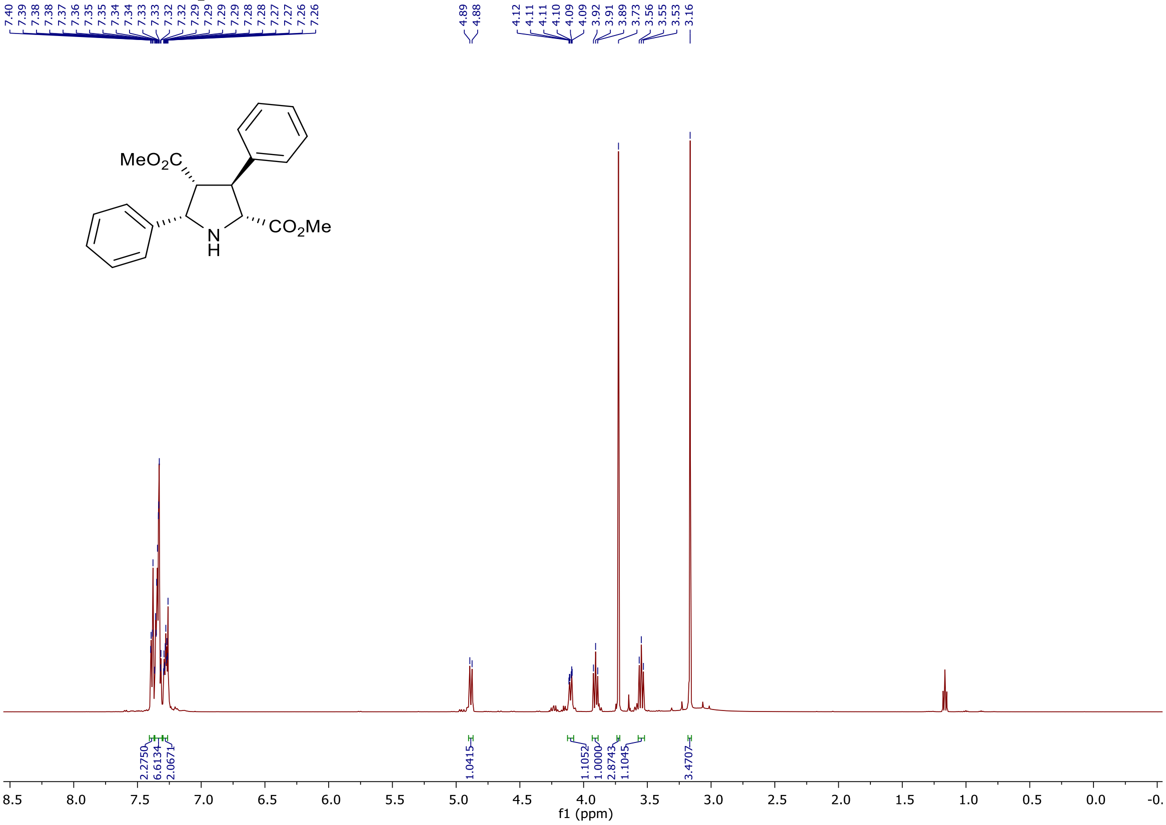

^{13}C NMR of compound A1

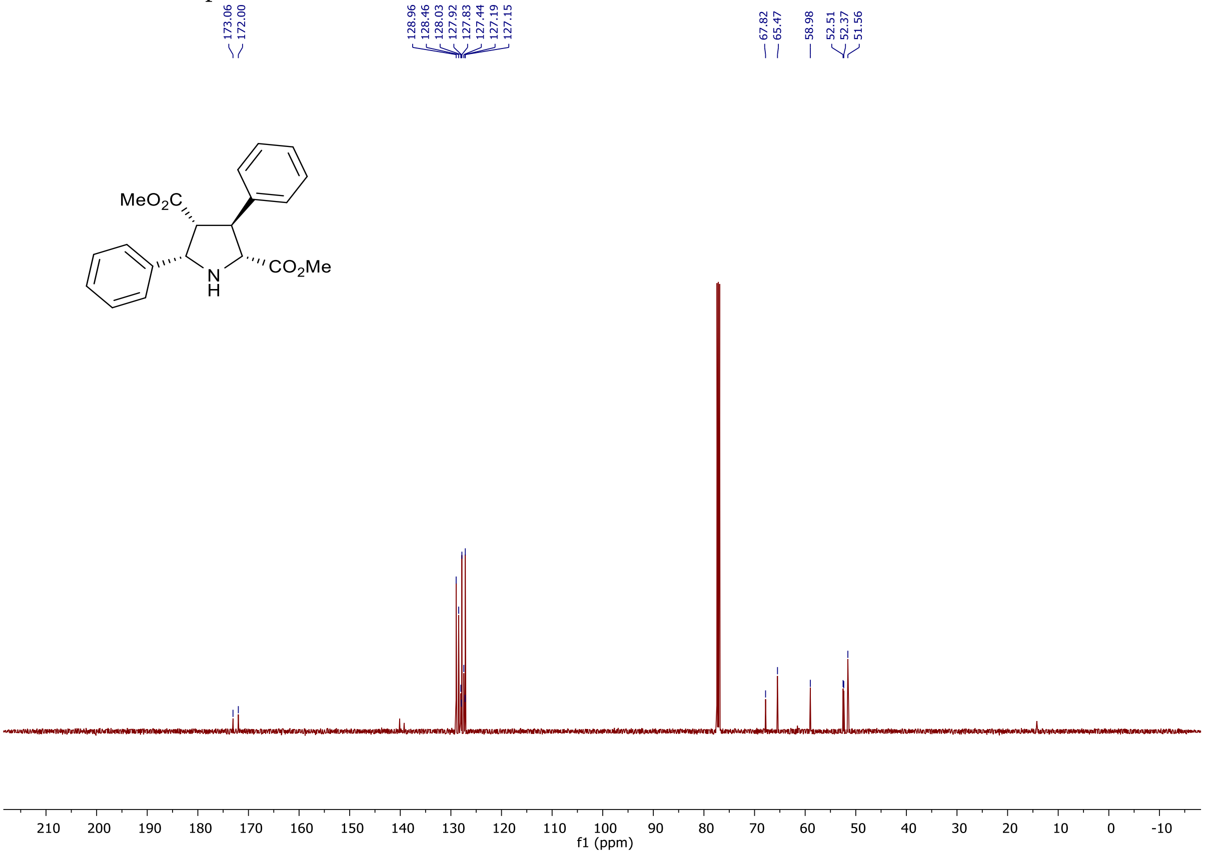

^{1}H NMR of compound A2

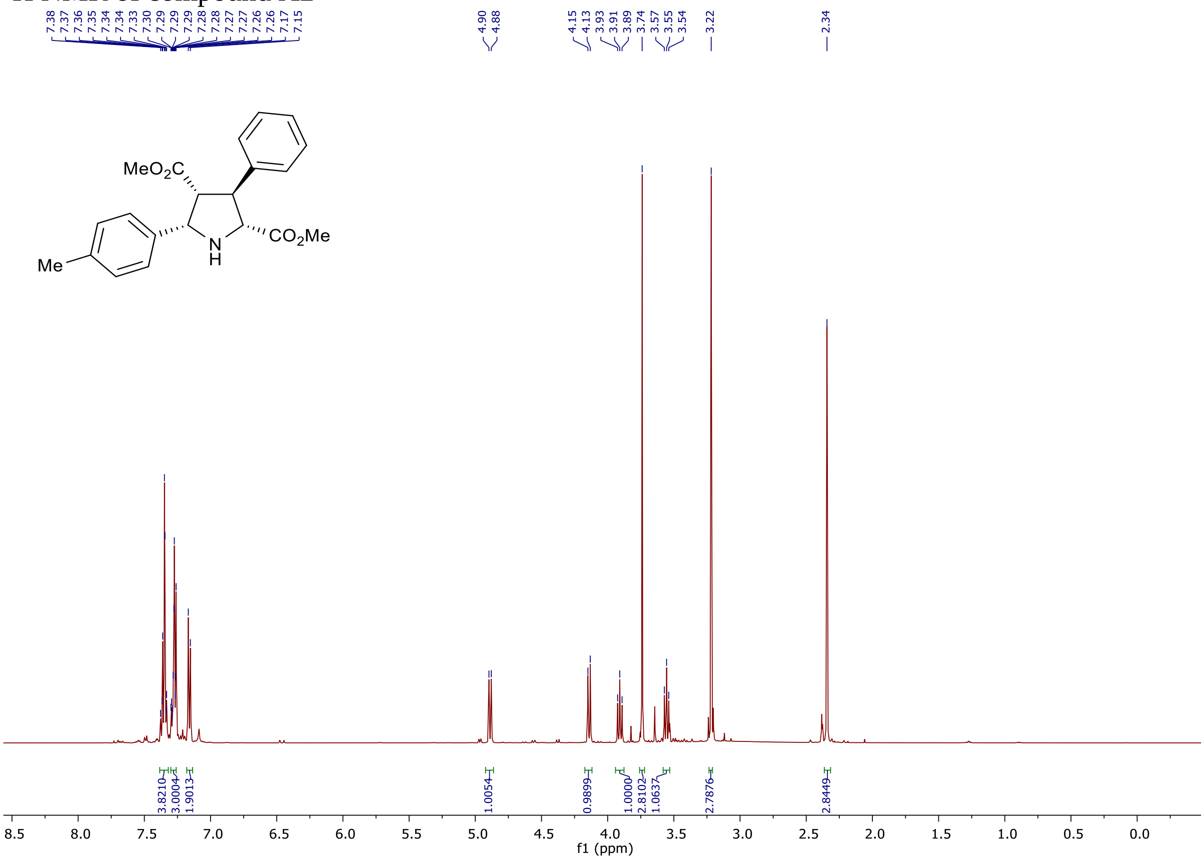

^{13}C NMR of compound A2

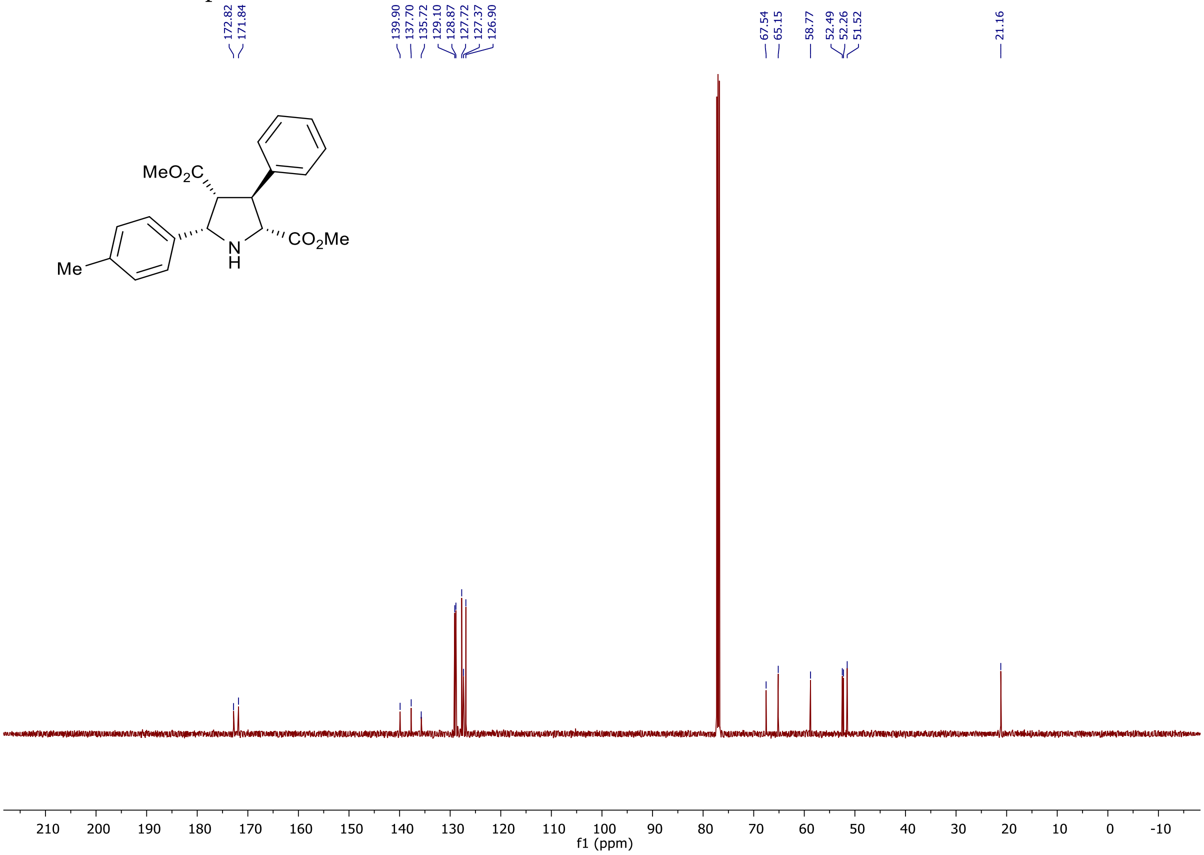

^{1}H NMR of Compound 51

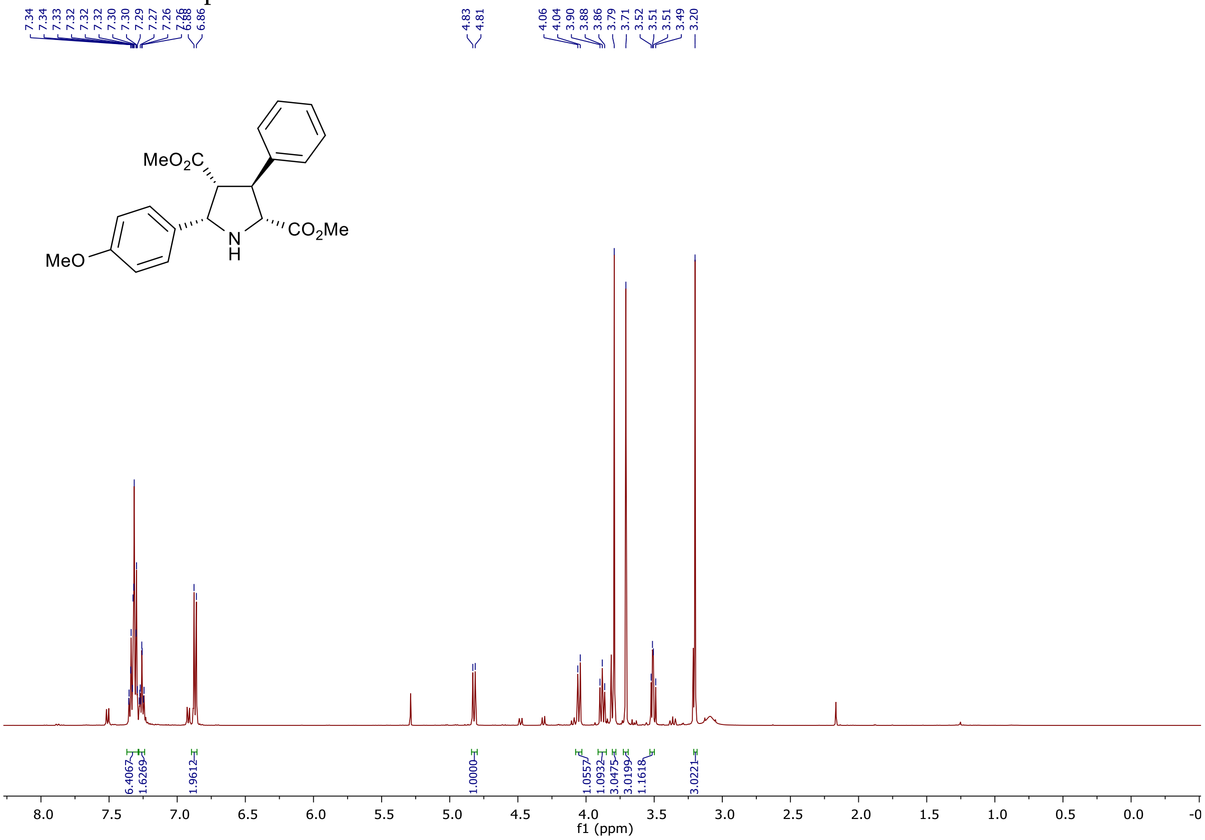

^{13}C NMR of compound 51

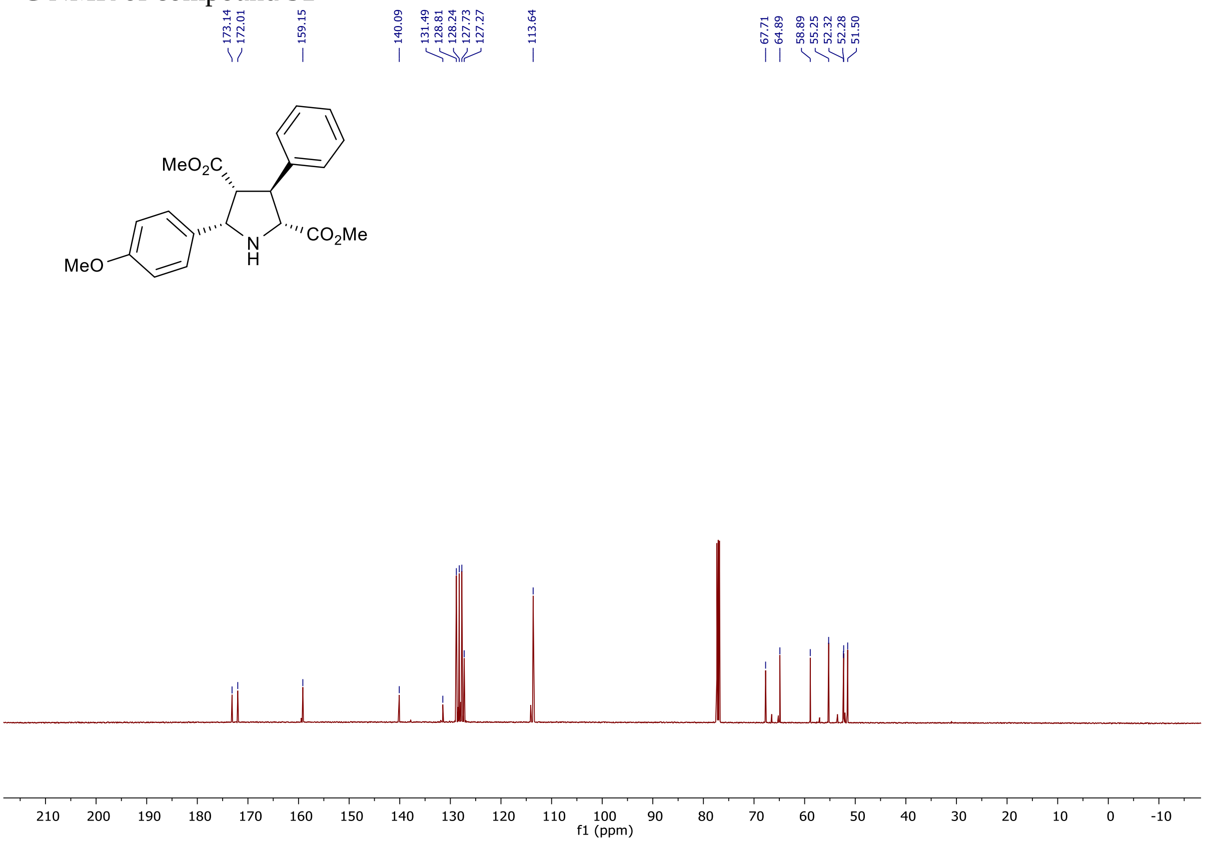

^{1}H NMR of Compound A3

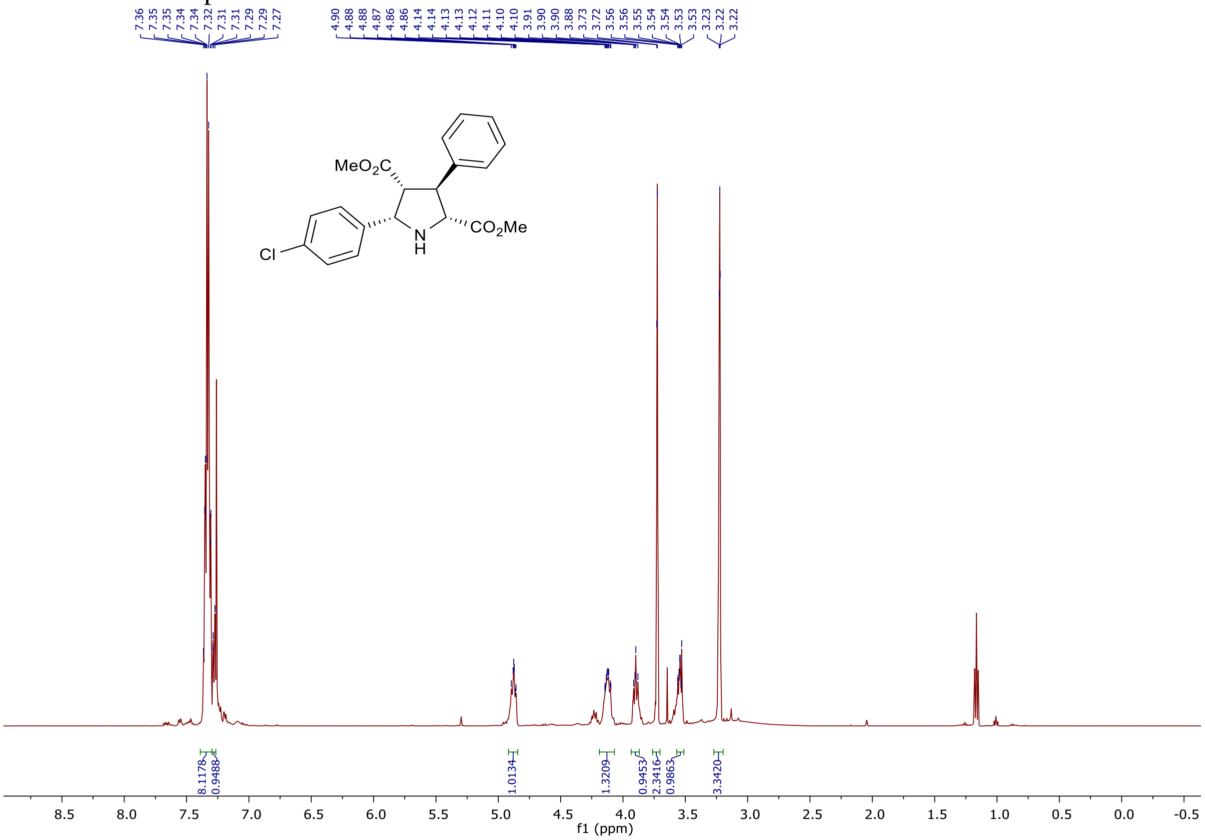

^{13}C NMR of compound A3

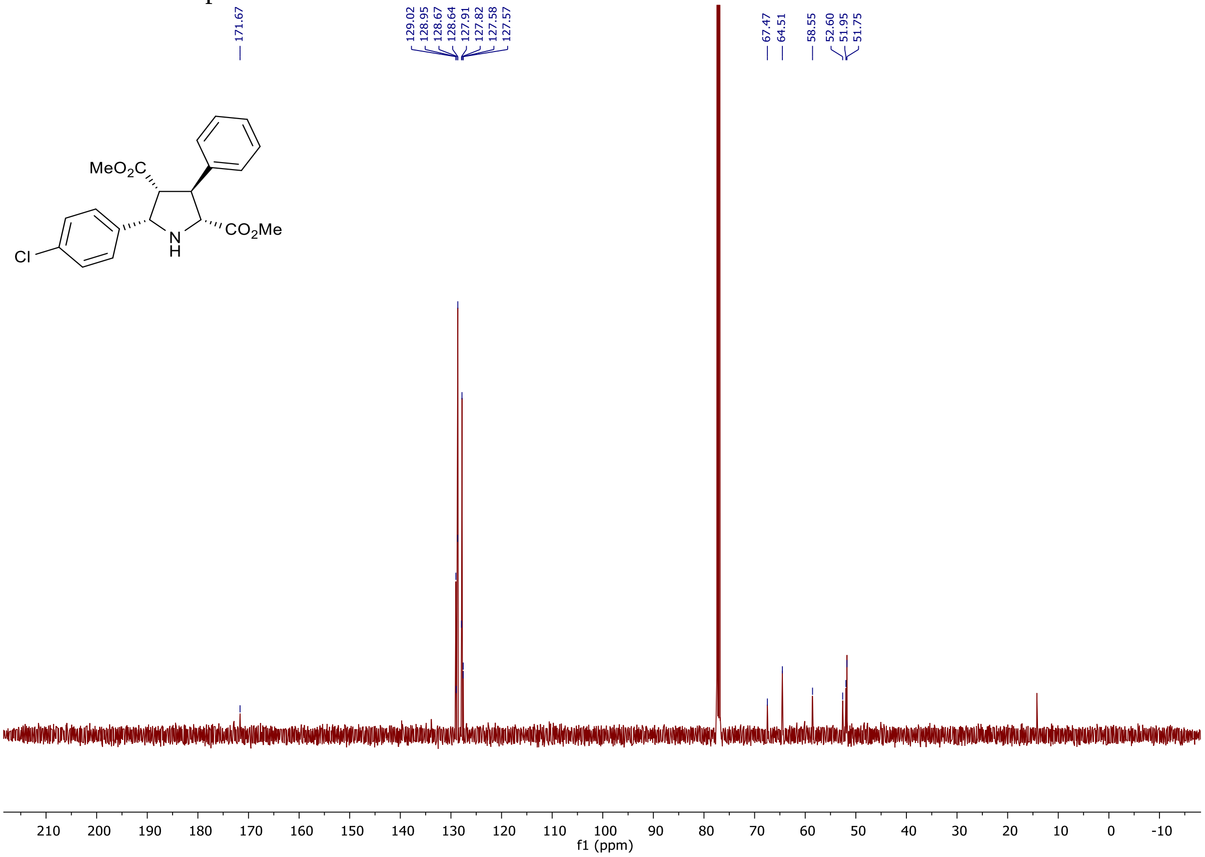

^{1}H NMR of Compound A4

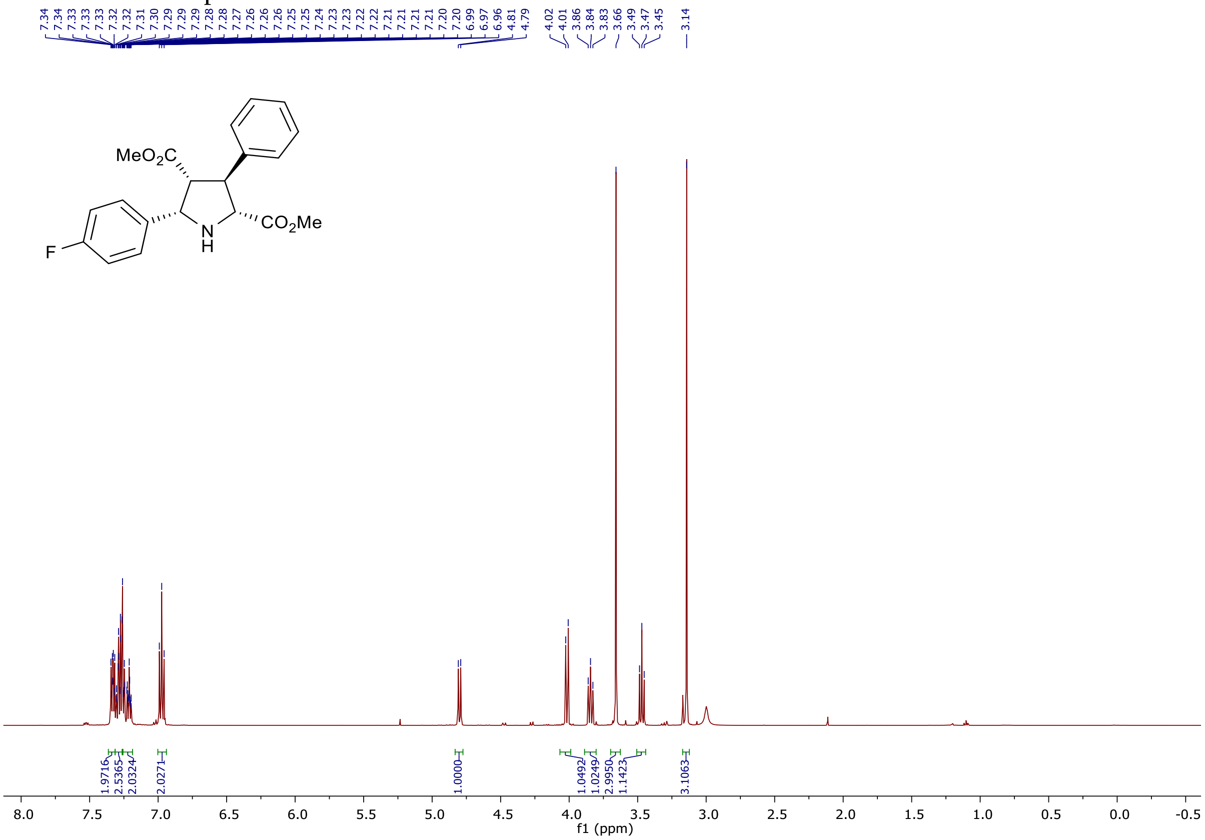

^{13}C NMR of compound A4

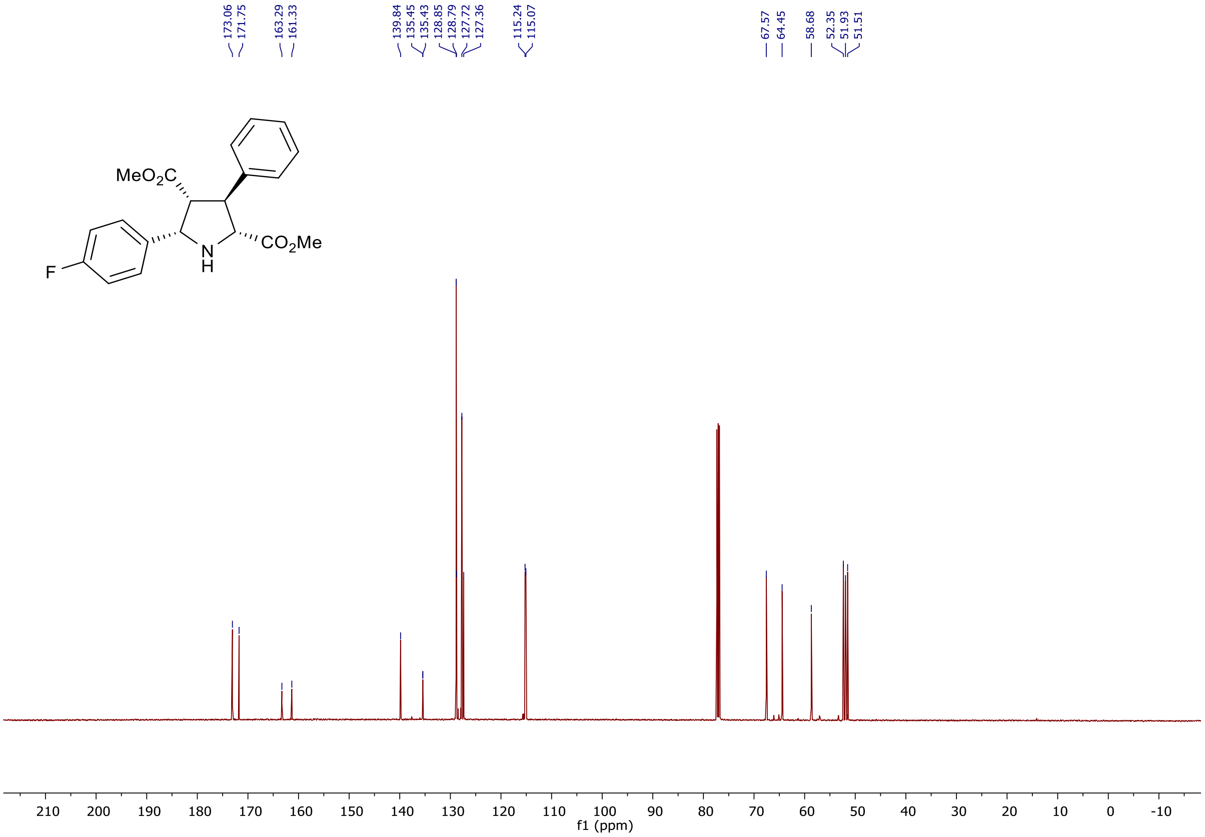

^{13}C NMR of compound A4

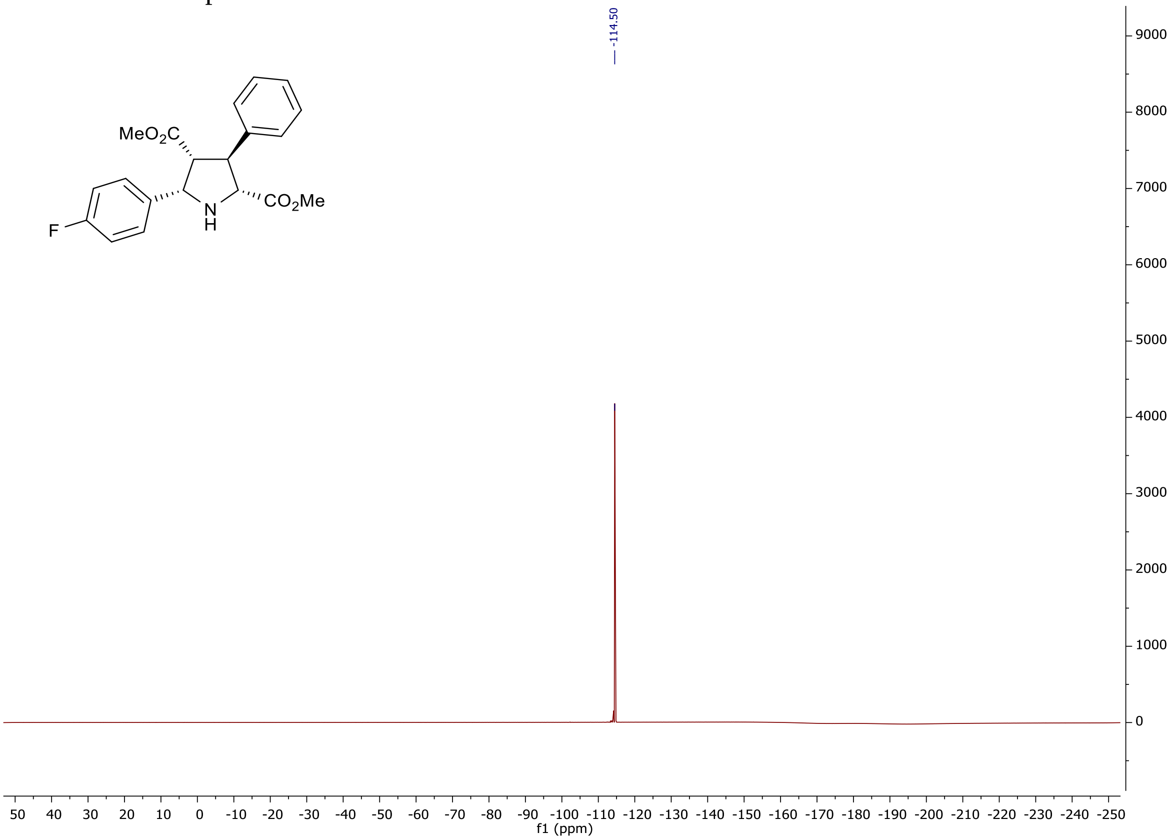

^{1}H NMR of Compound A5

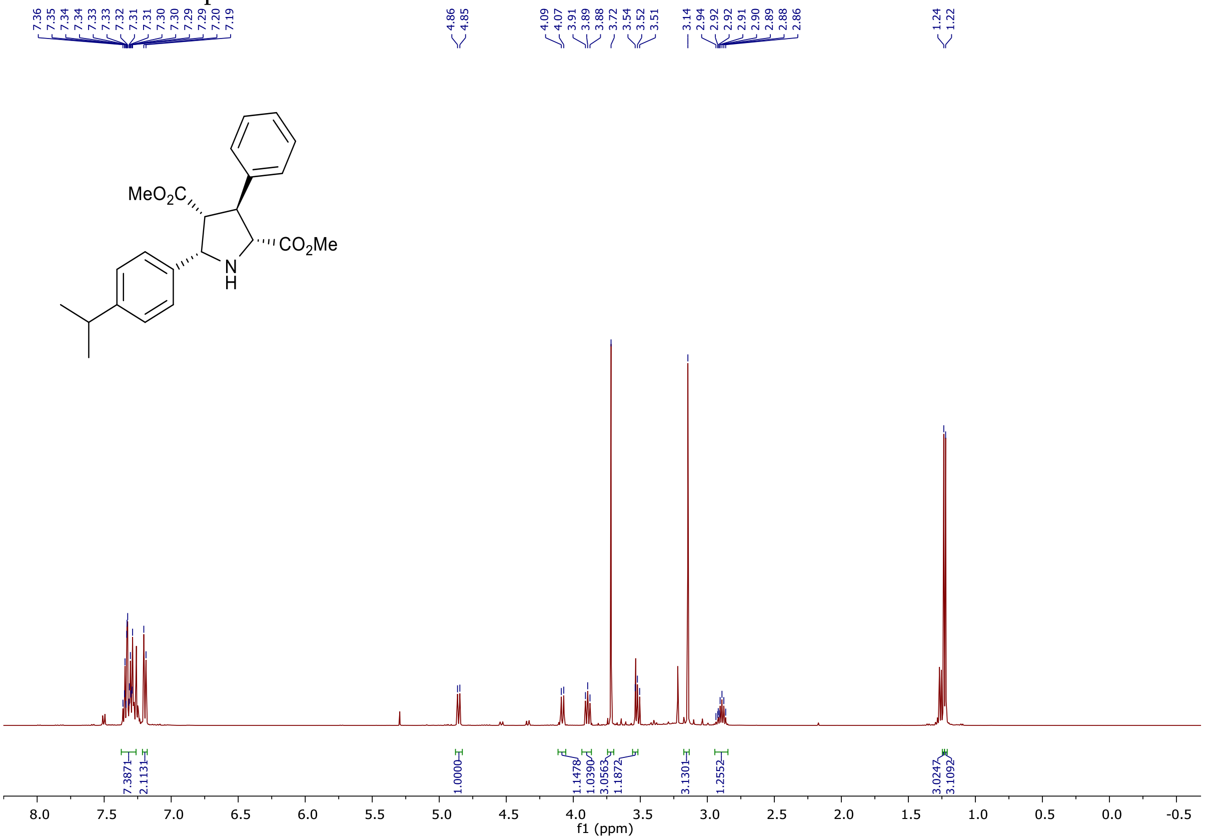

^{13}C NMR of compound A5

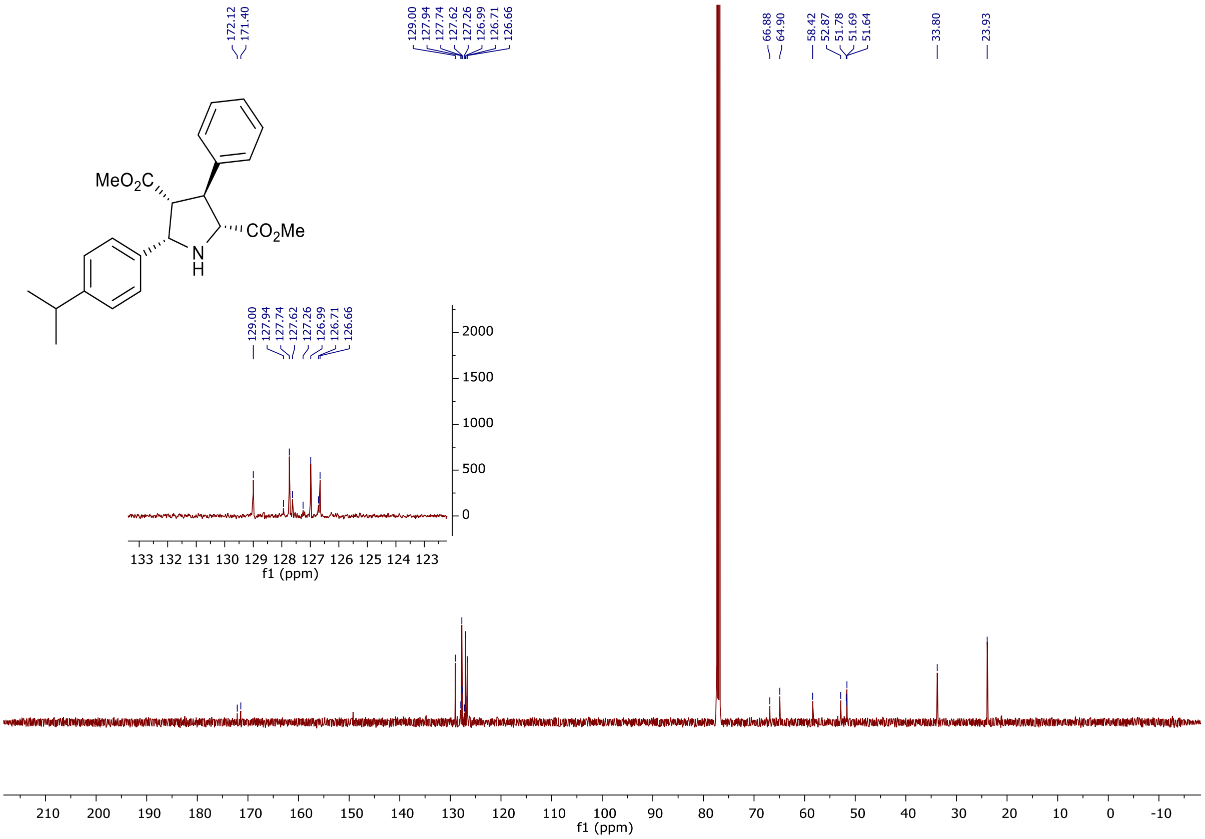

^{1}H NMR of Compound A6

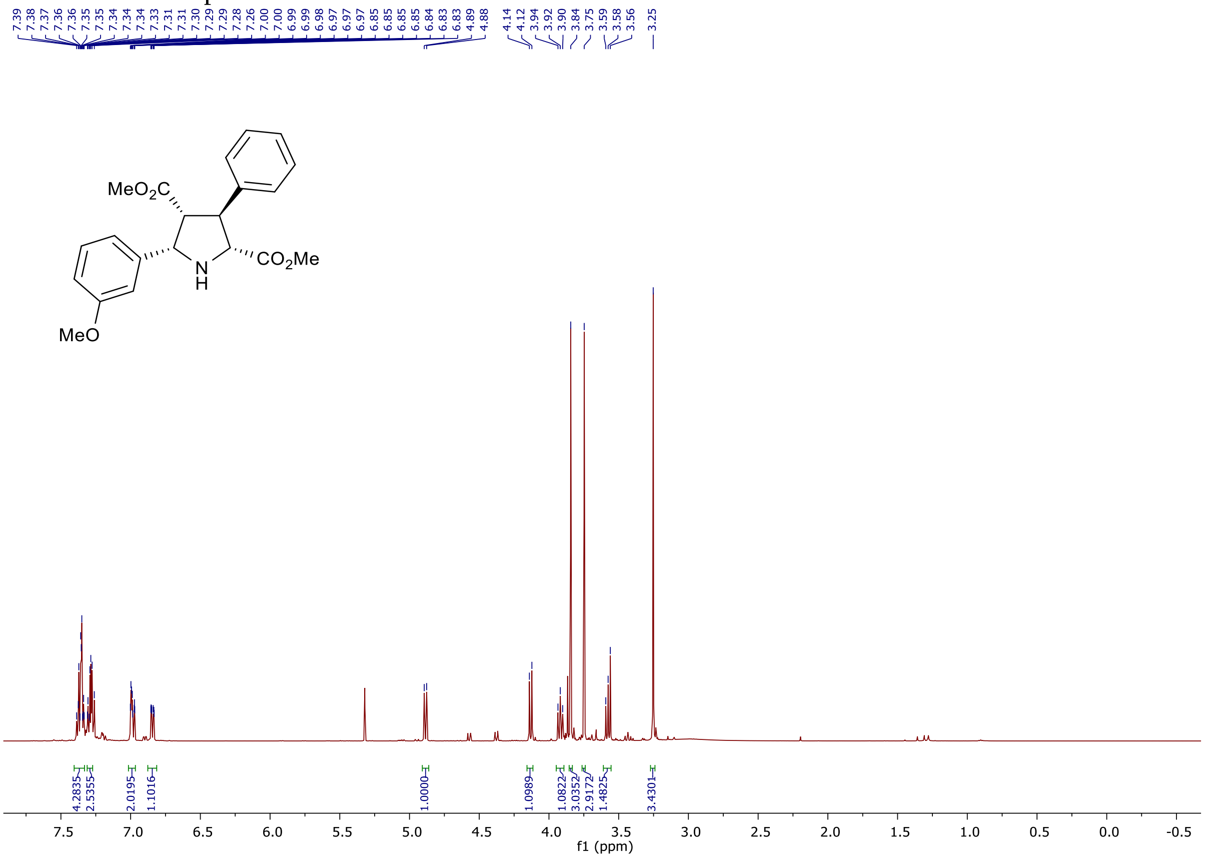

^{13}C NMR of compound A6

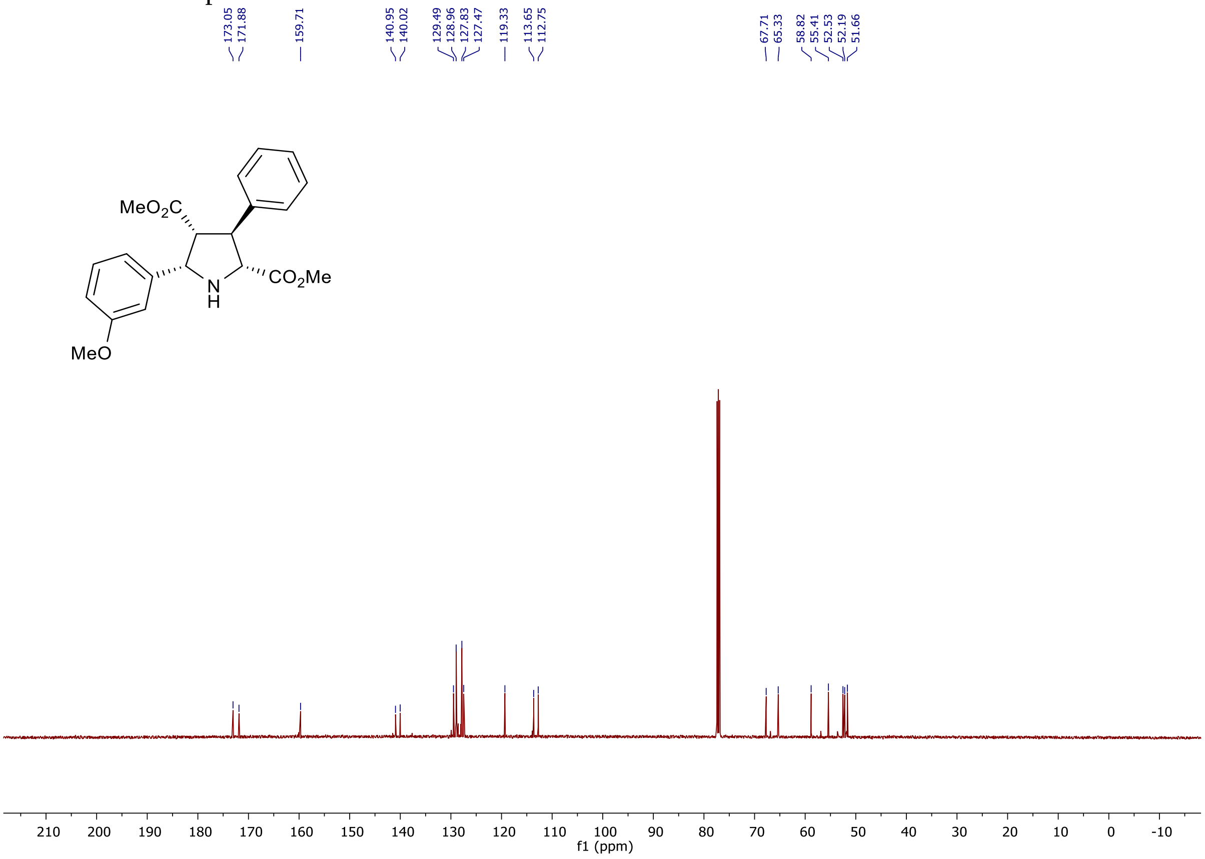

^{1}H NMR of Compound A7

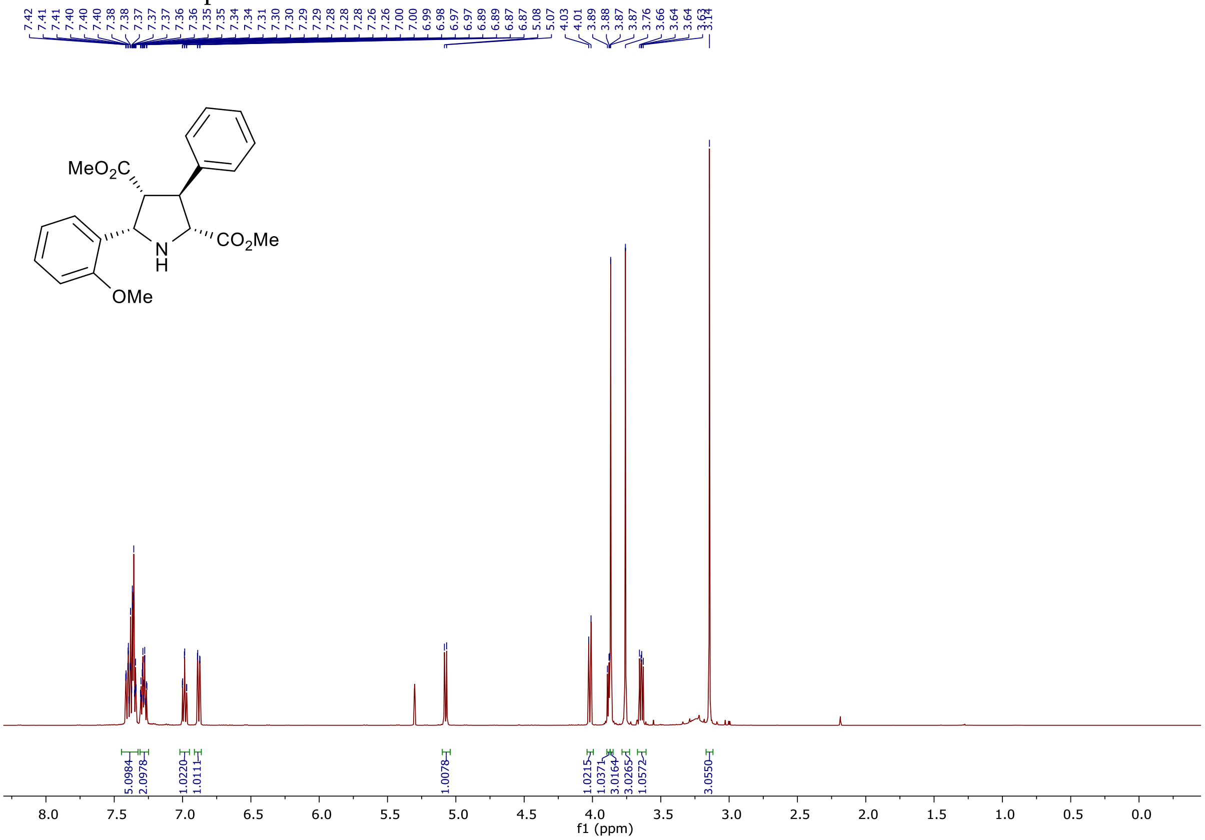

^{13}C NMR of compound A7

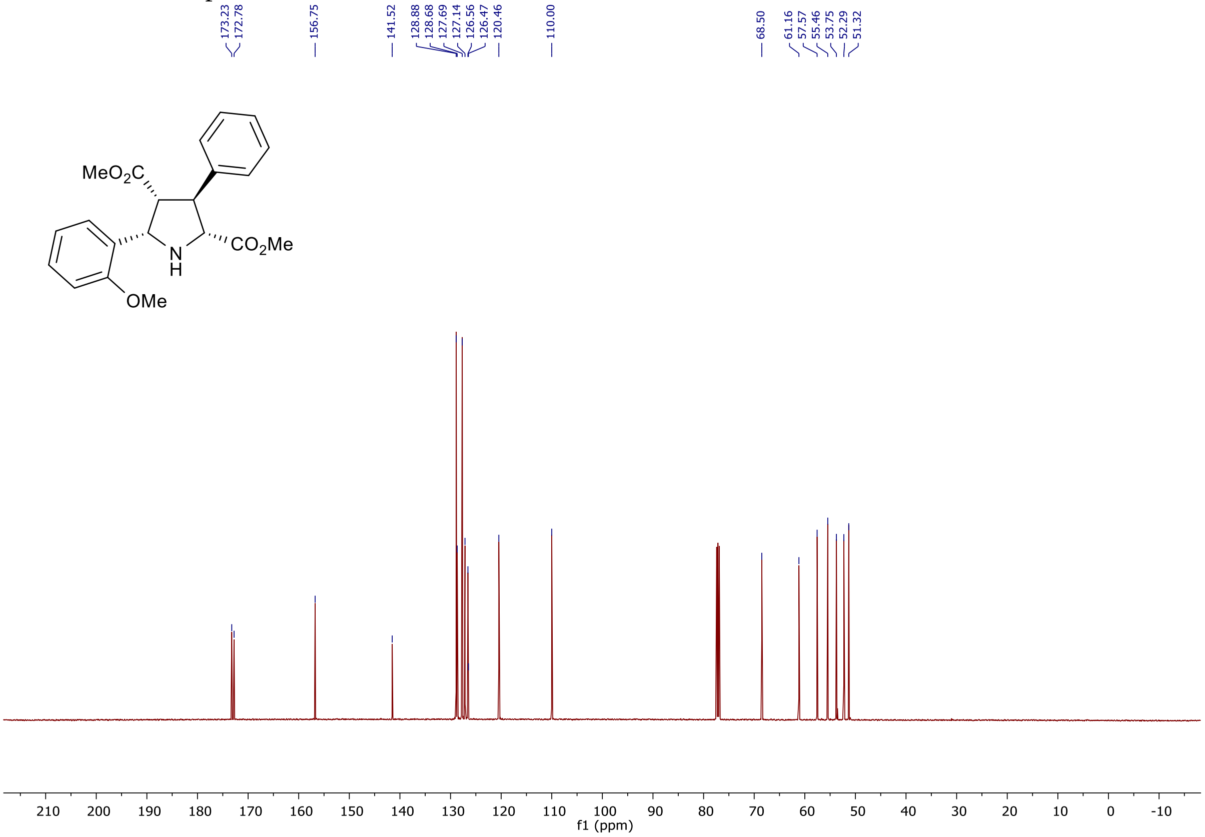

^{1}H NMR of Compound A8

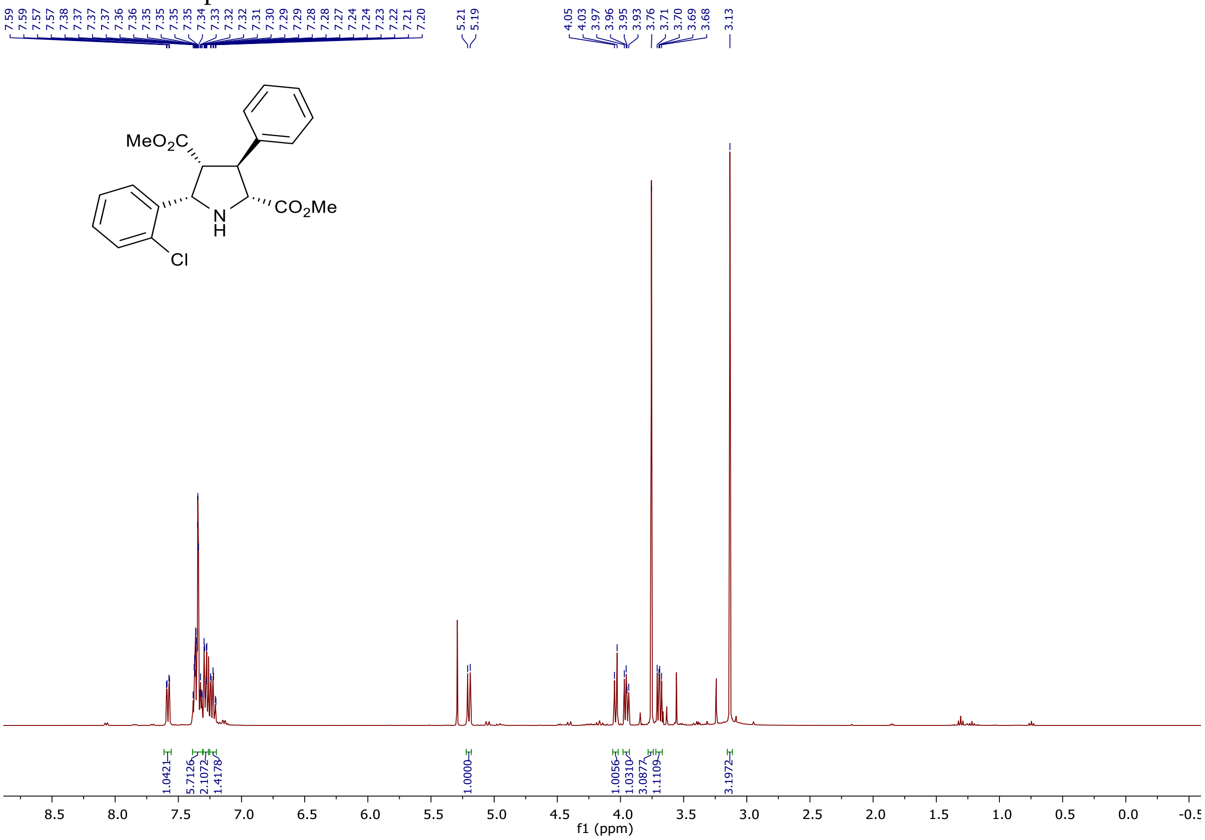

^{13}C NMR of compound A8

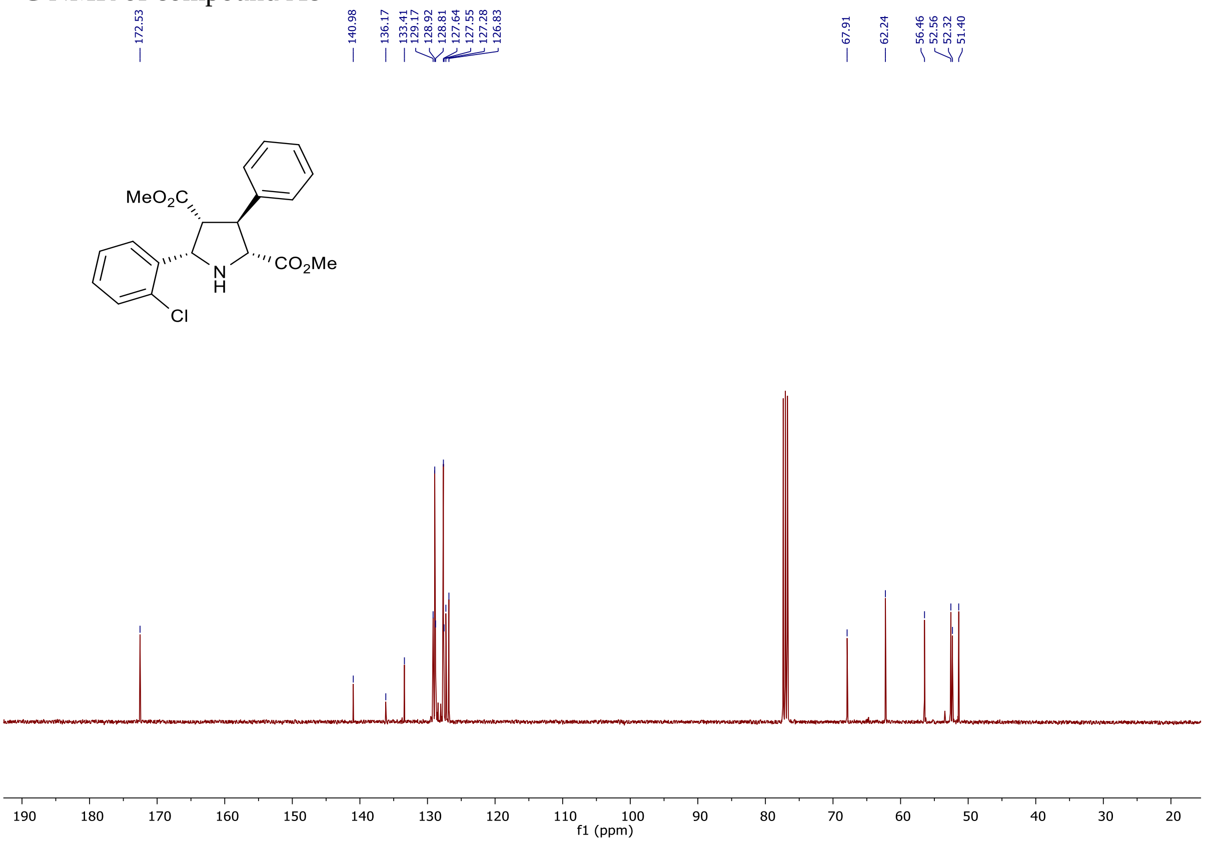

^{1}H NMR of Compound A9

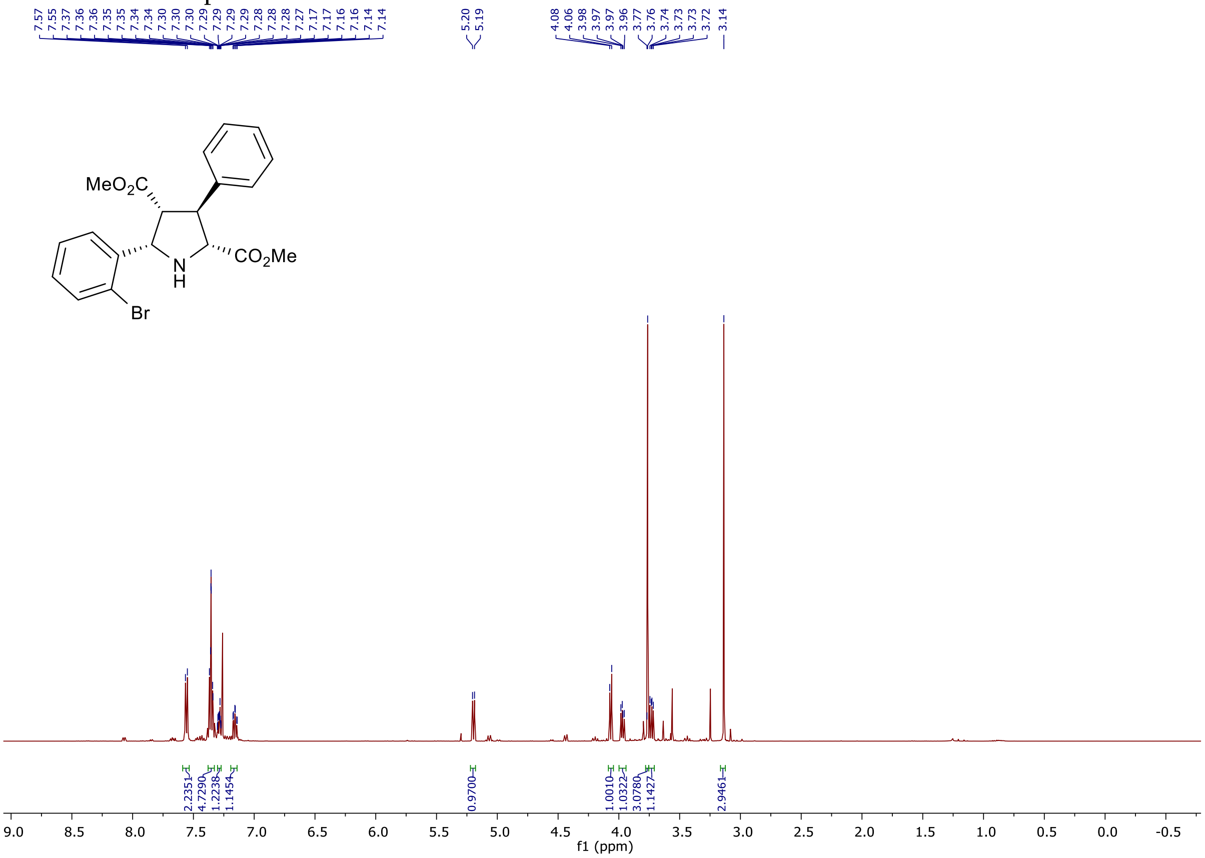

^{13}C NMR of compound A9

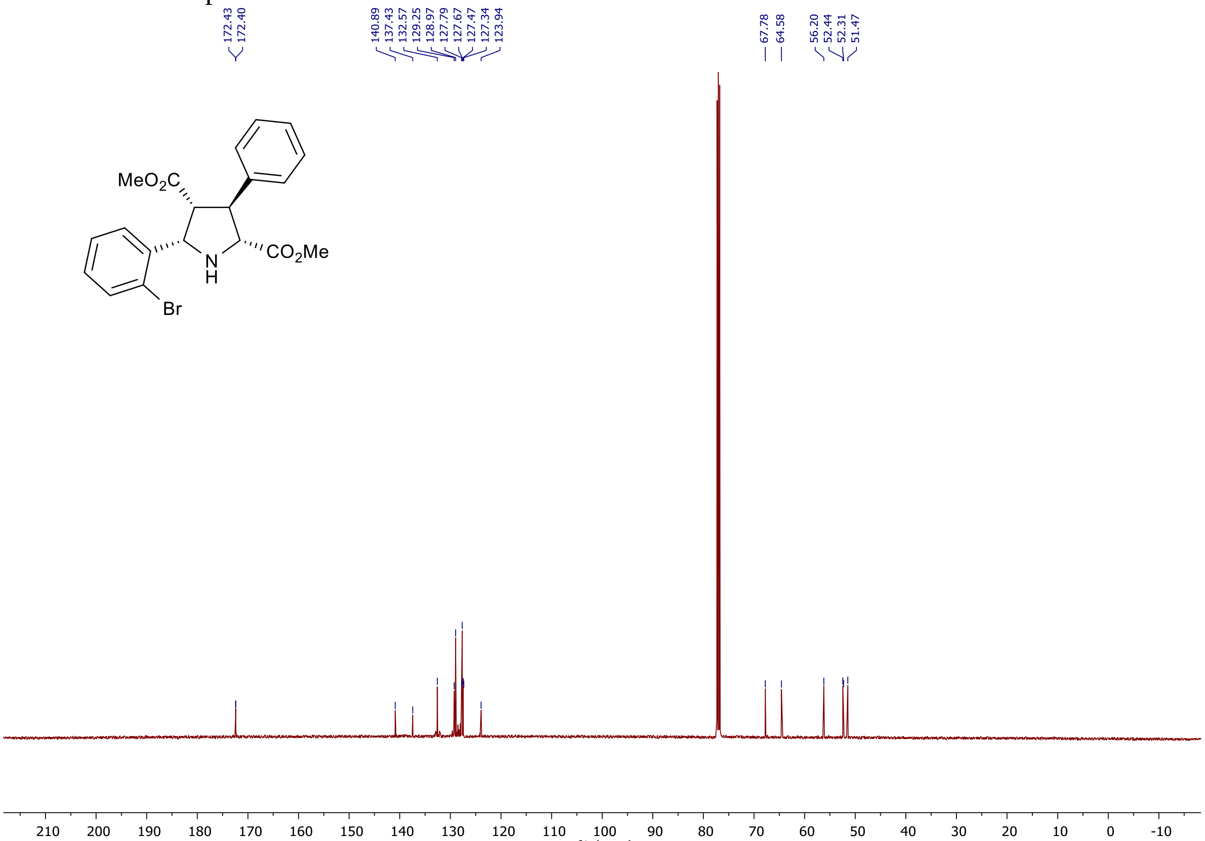

^{1}H NMR of compound A10

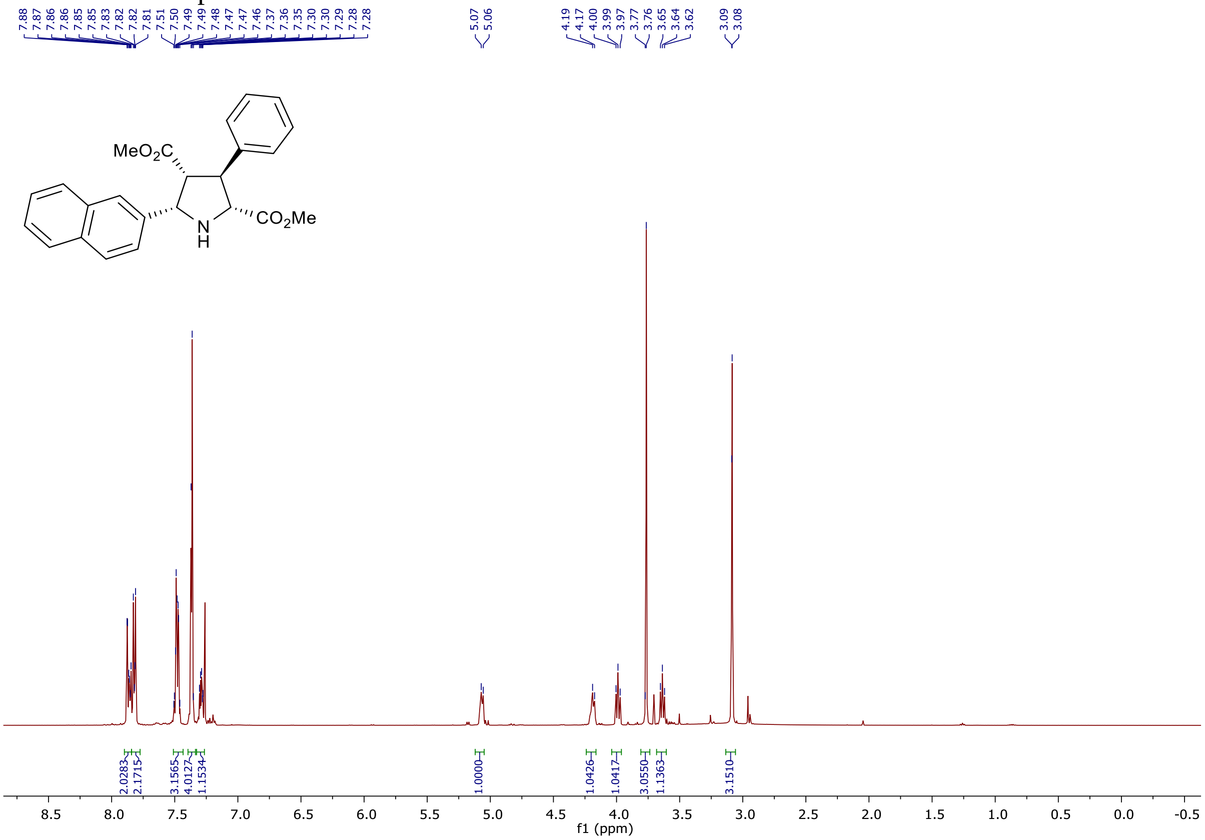

^{13}C NMR of compound A10

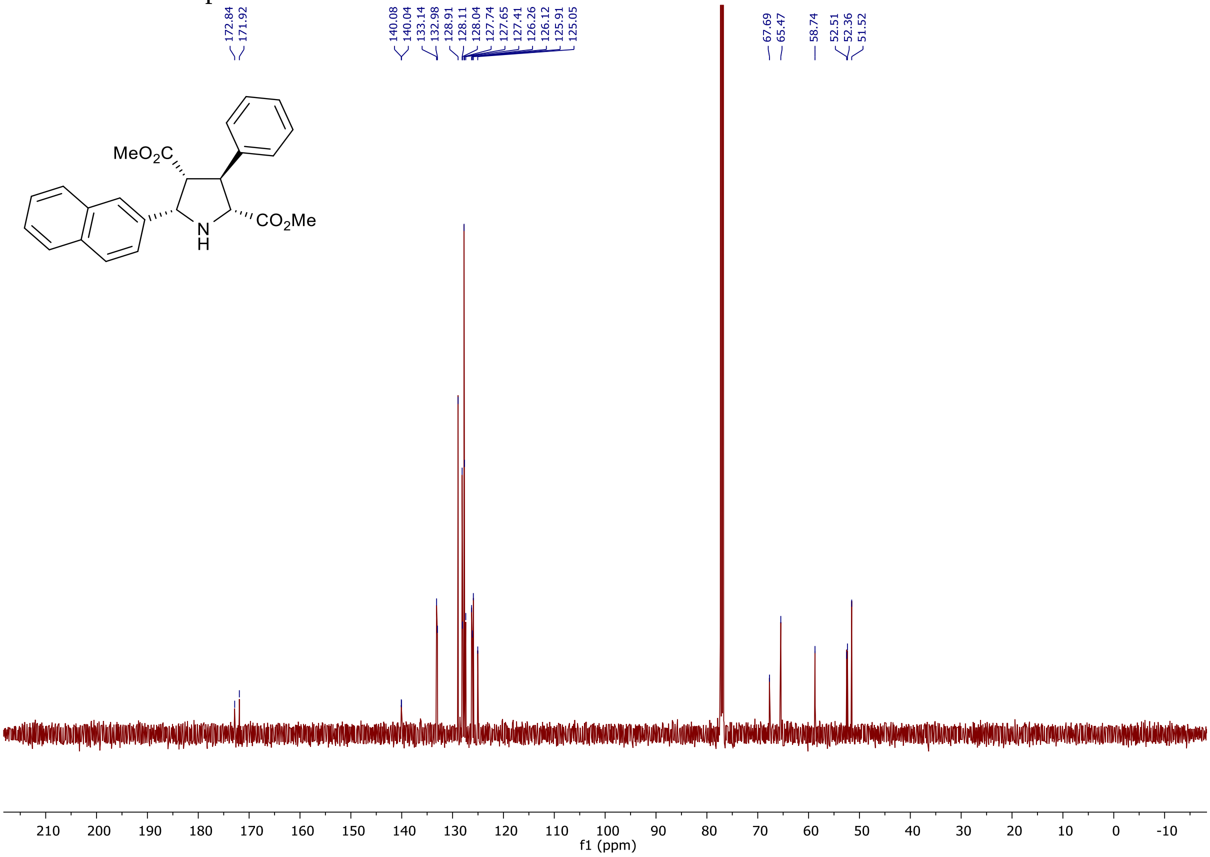

^{1}H NMR of compound A11

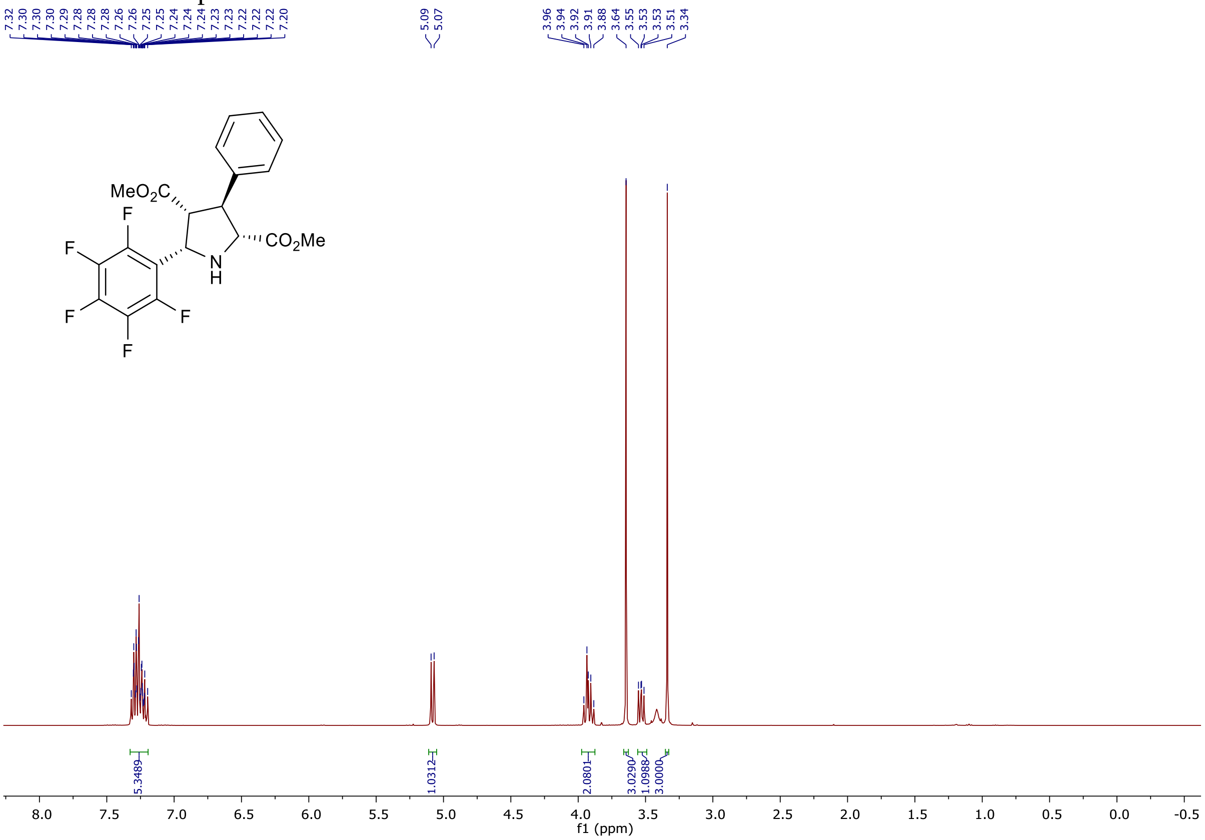

^{13}C NMR of compound A11

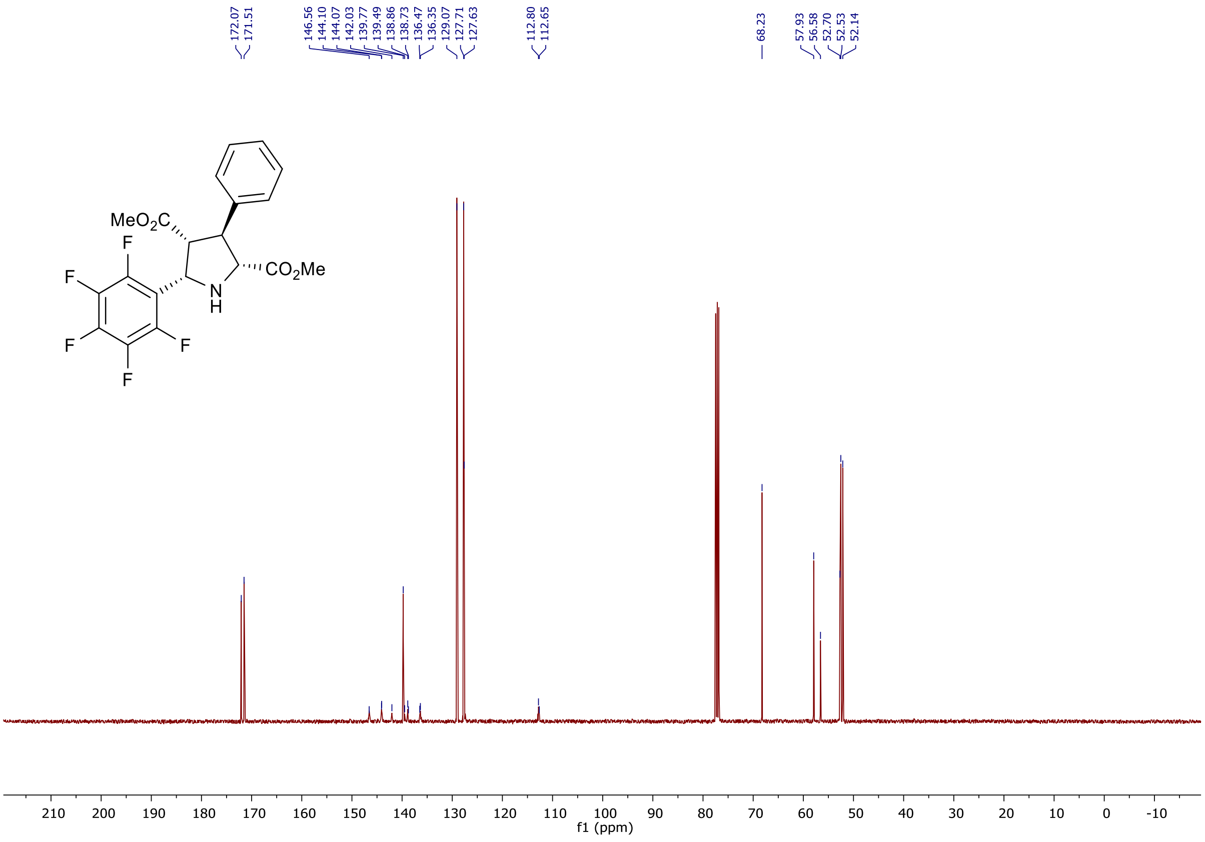

^{19}F NMR of compound A11

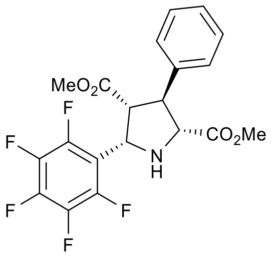

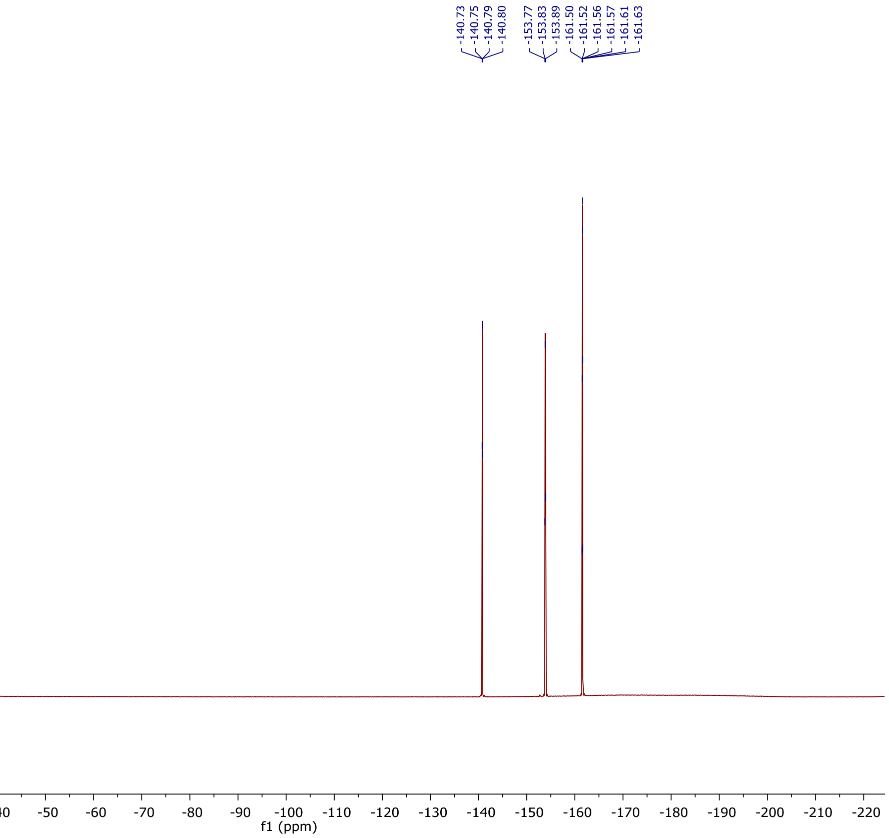

^{1}H NMR of compound A12

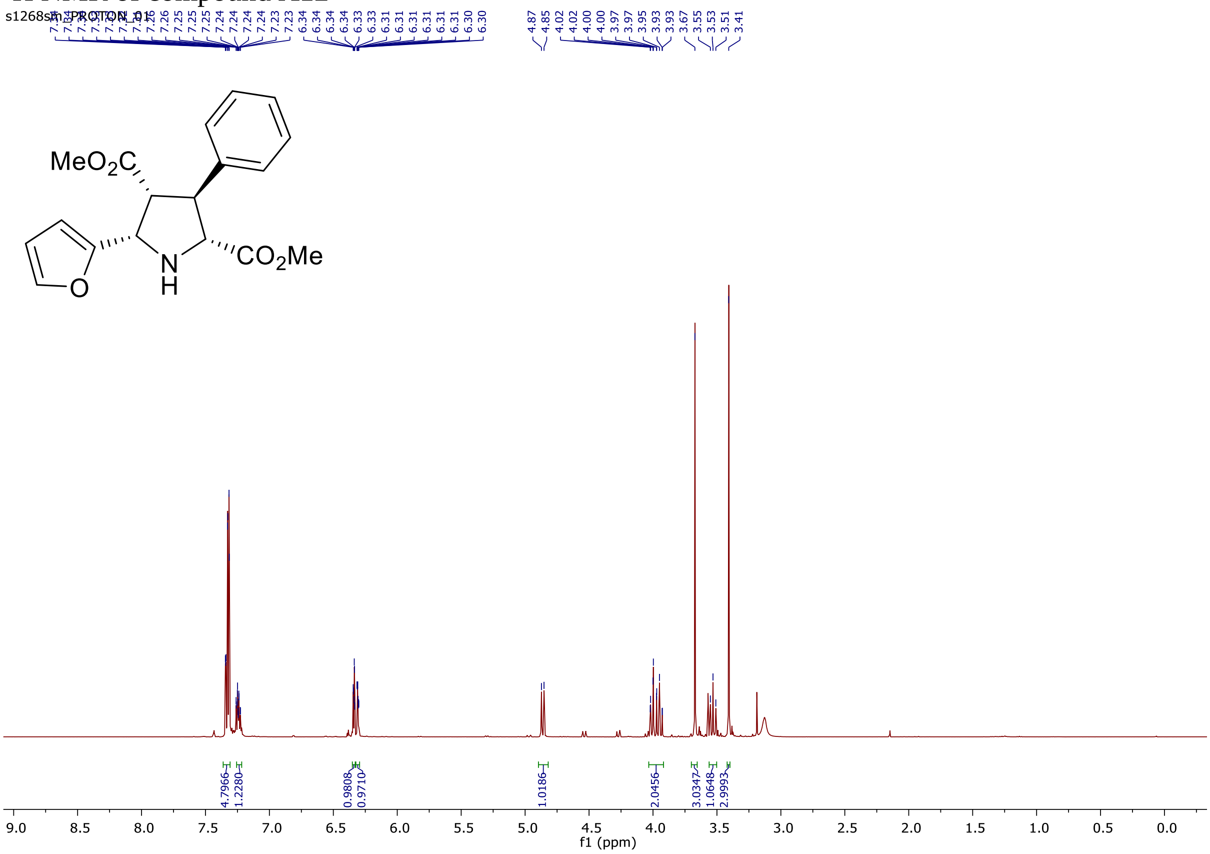

13C NMR of compound A12

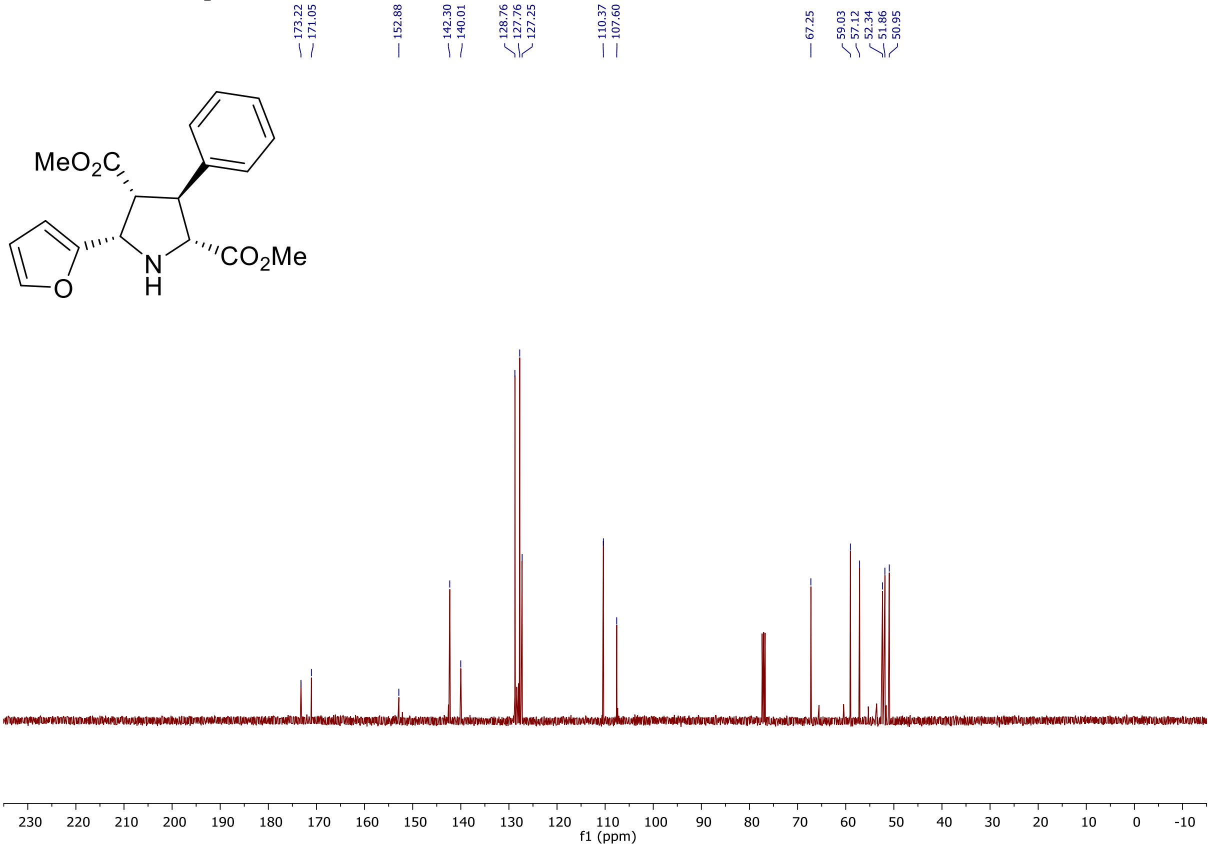

^{1}H NMR of compound A13

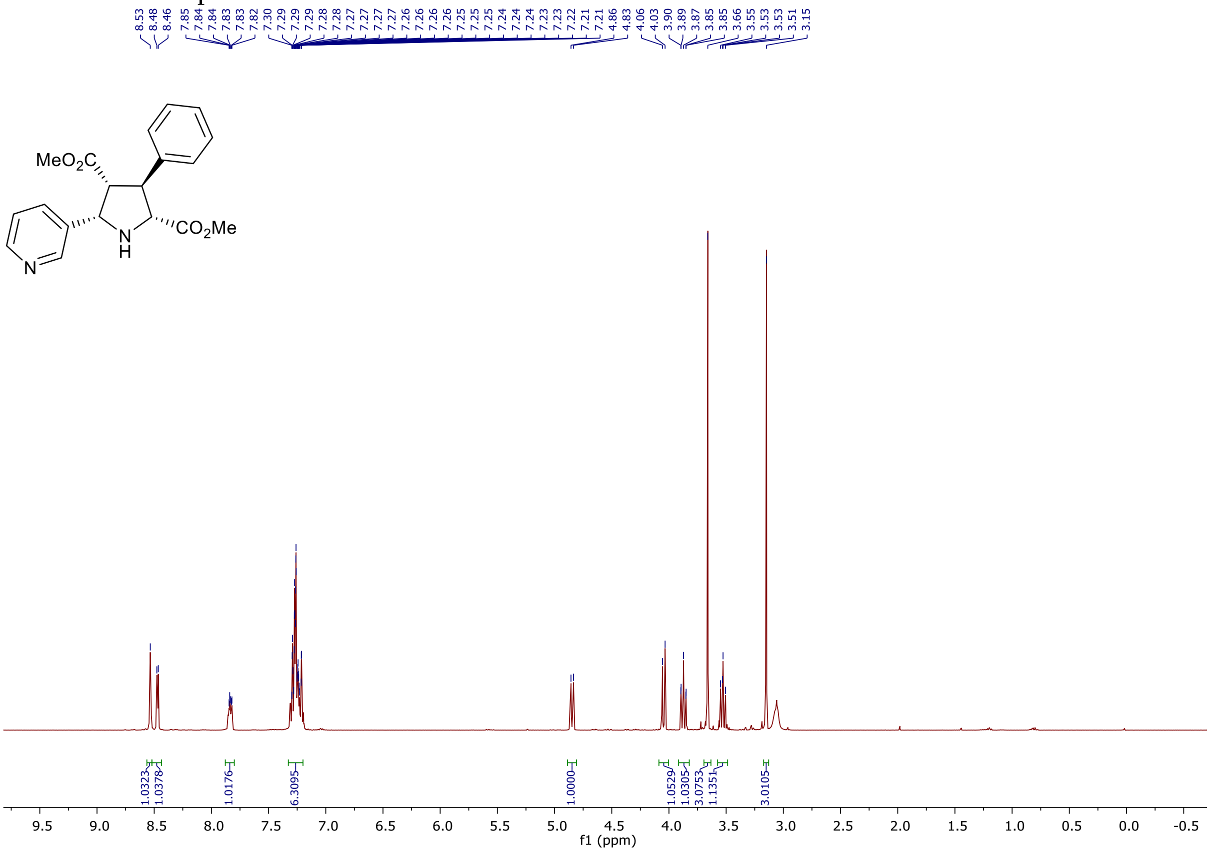

13C NMR of compound A13

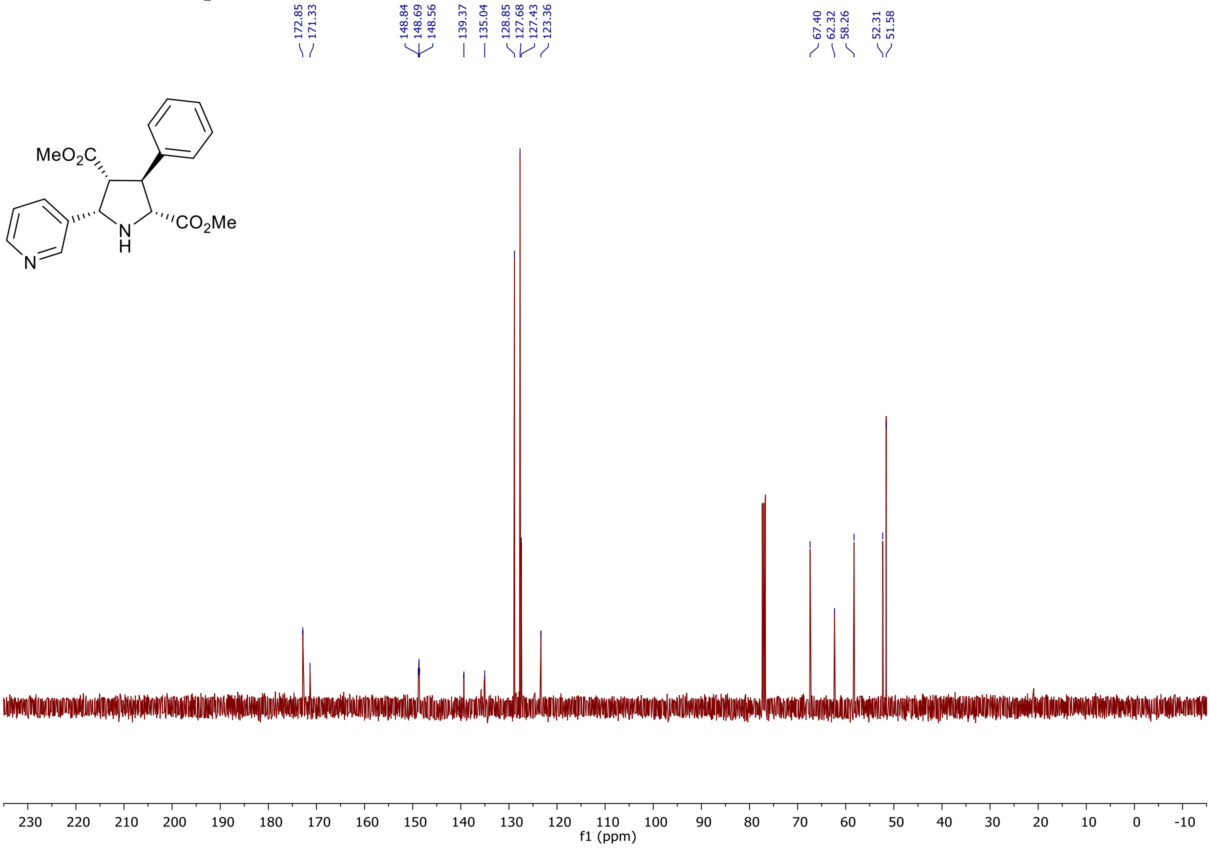

^{1}H NMR of compound A14

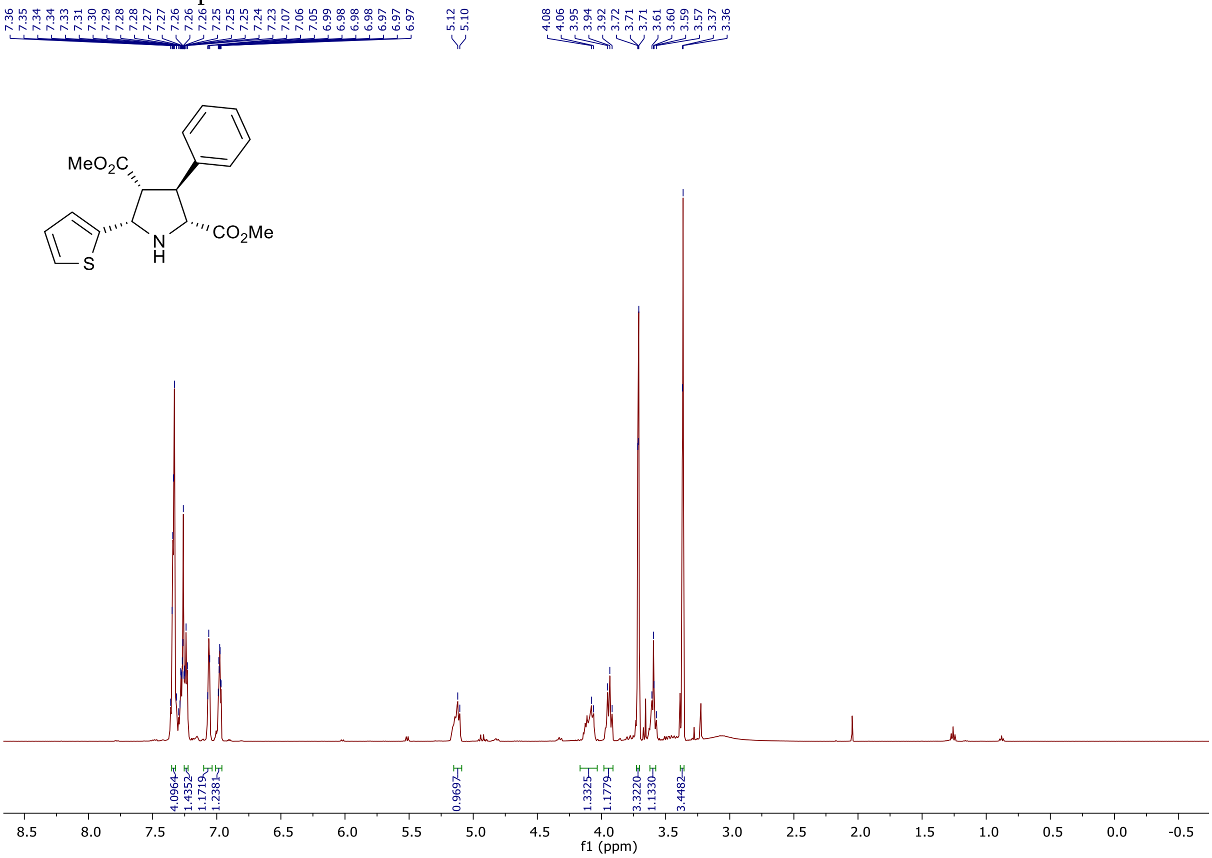

^{13}C NMR of compound A14

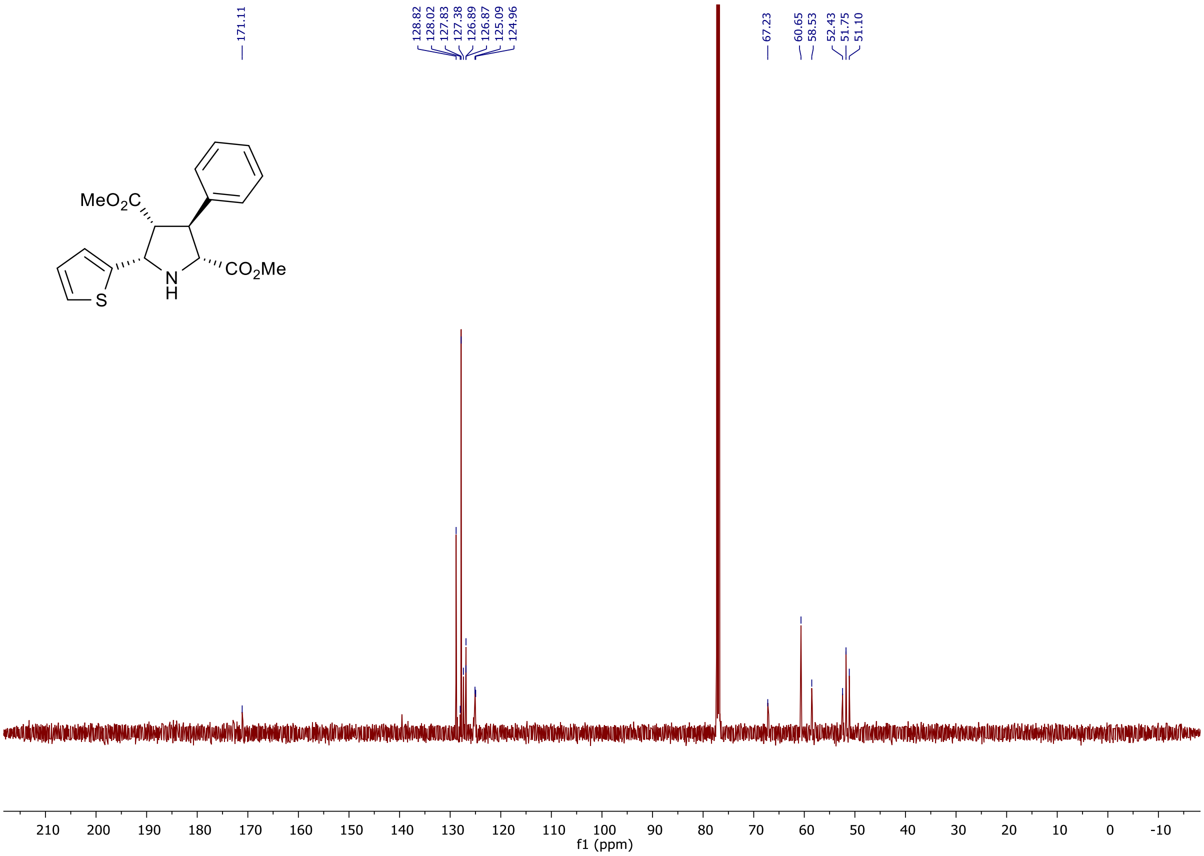

^{1}H NMR of compound A15

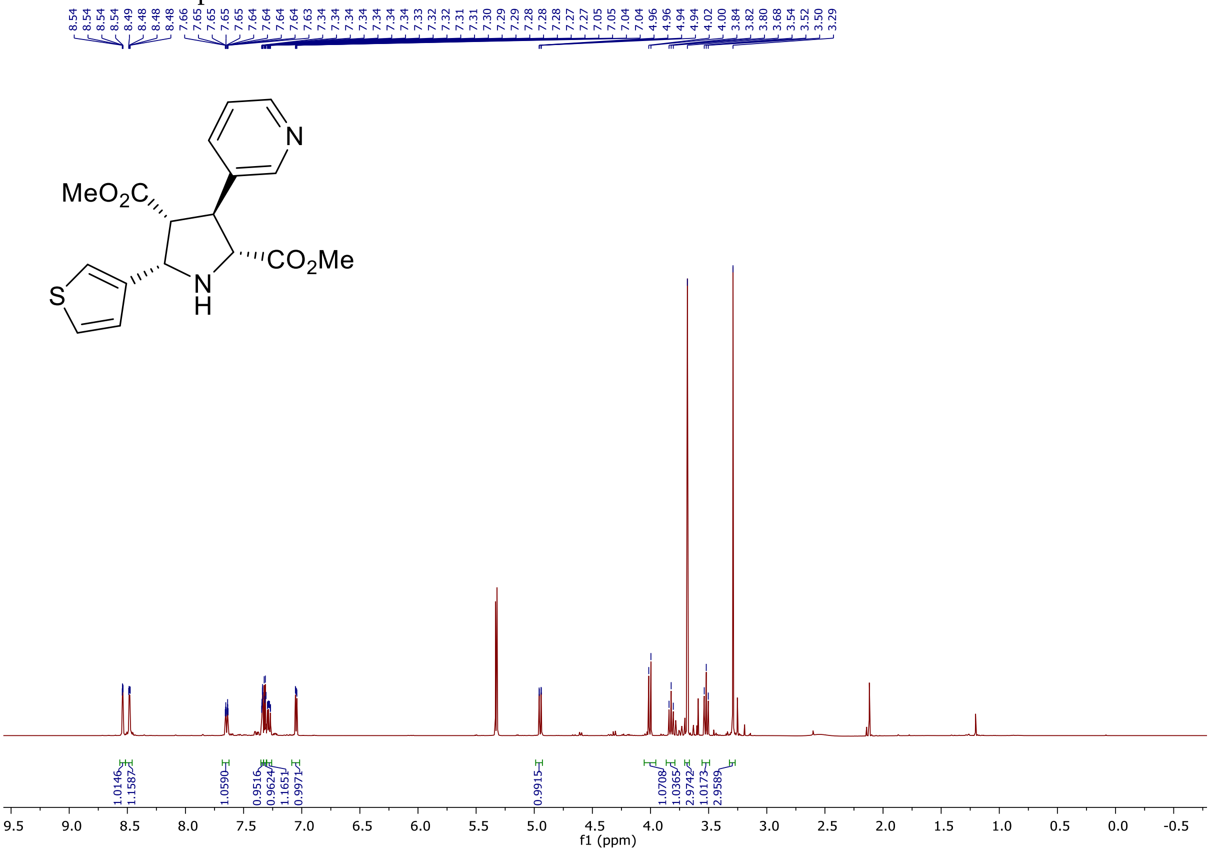

13C NMR of compound A15

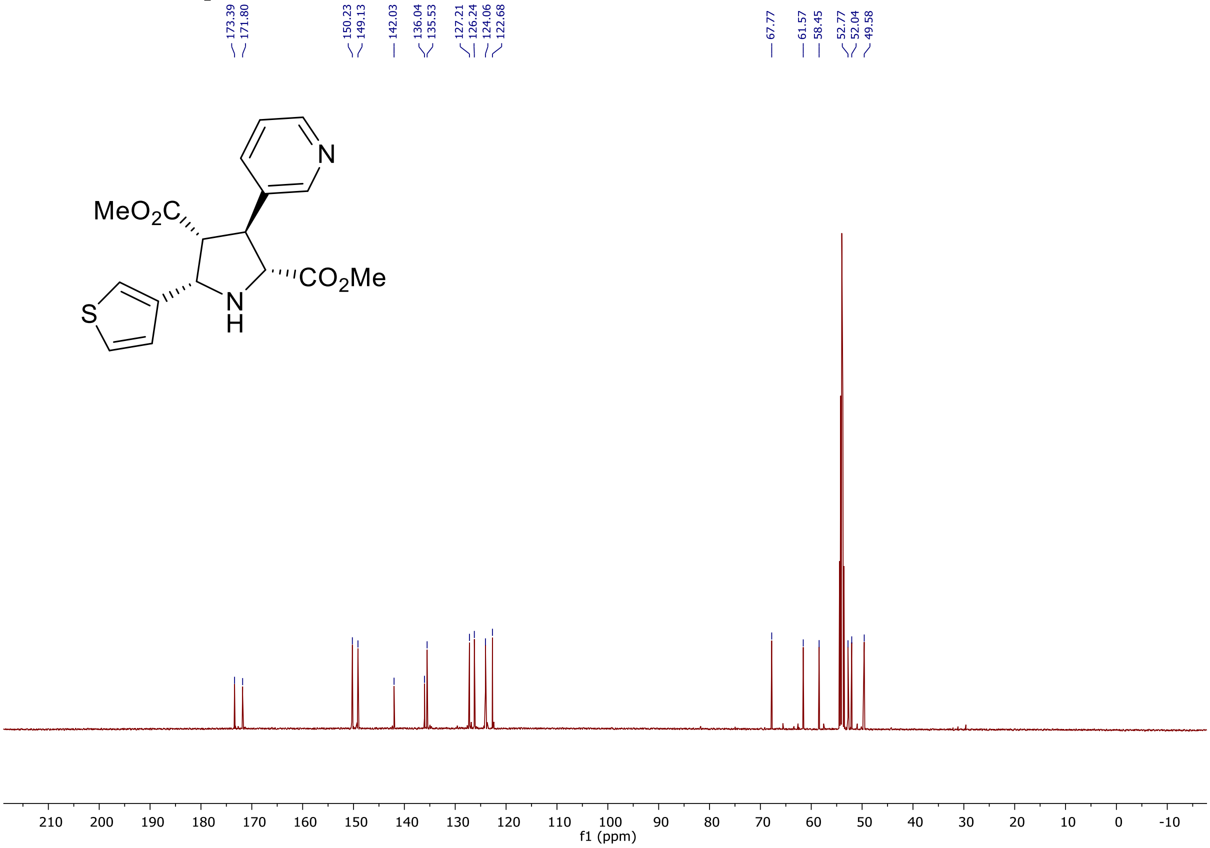

^{1}H NMR of compound A16

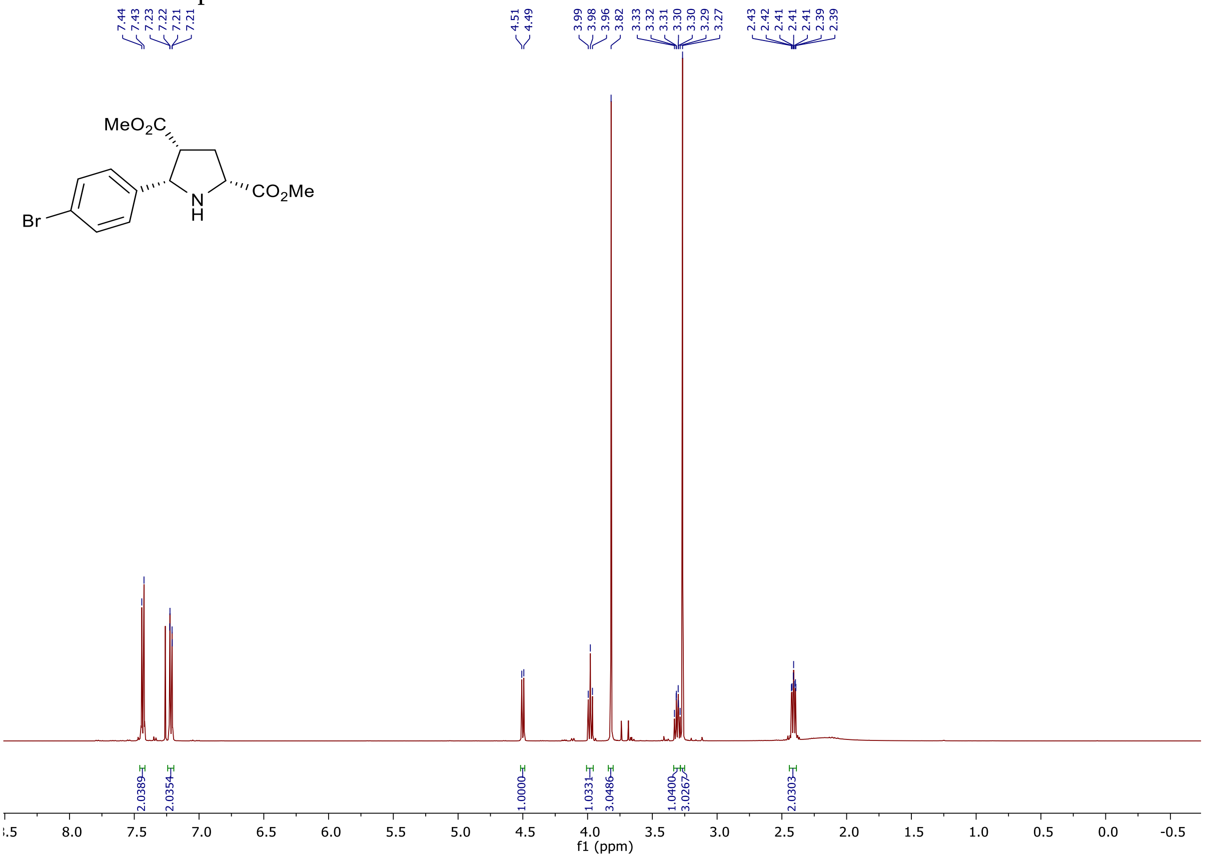

^{13}C NMR of compound A16

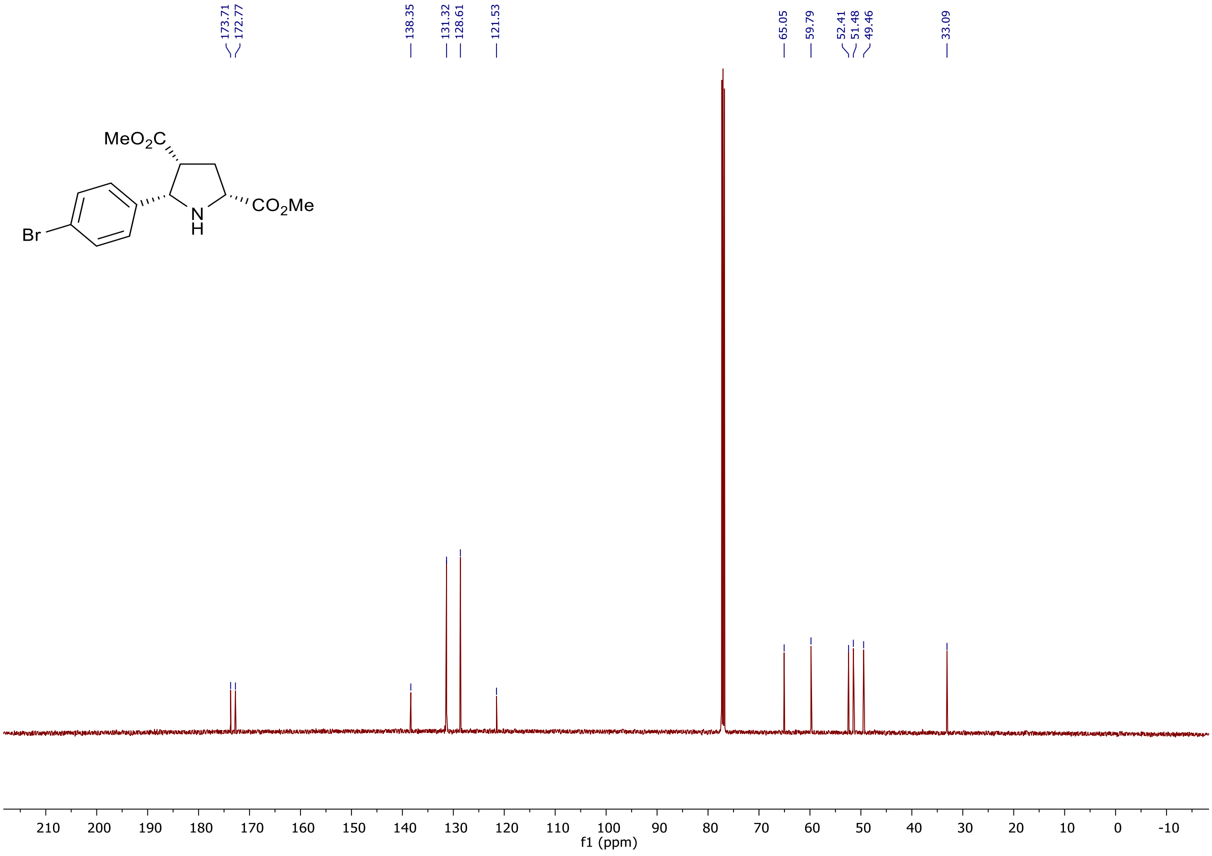

^{1}H NMR of compound A17

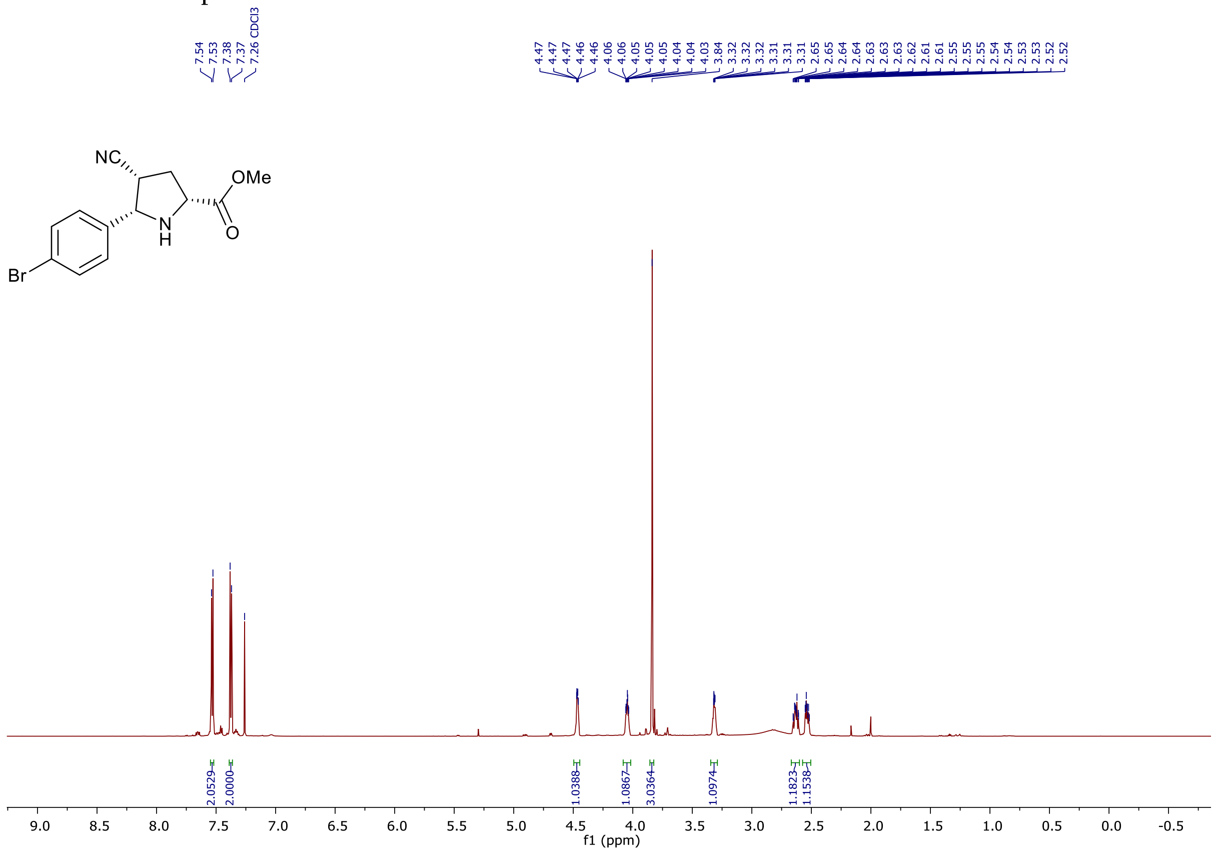

13C NMR

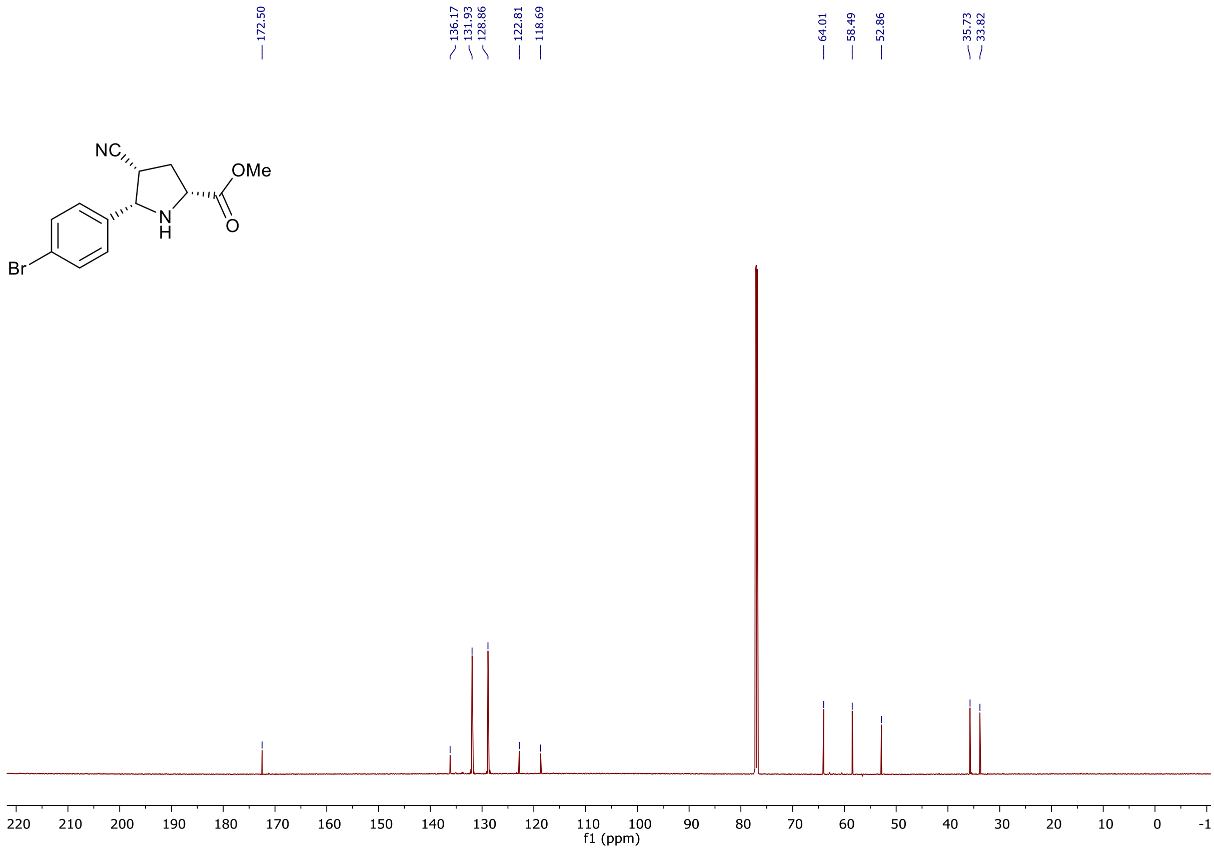

^{1}H NMR of compound A18

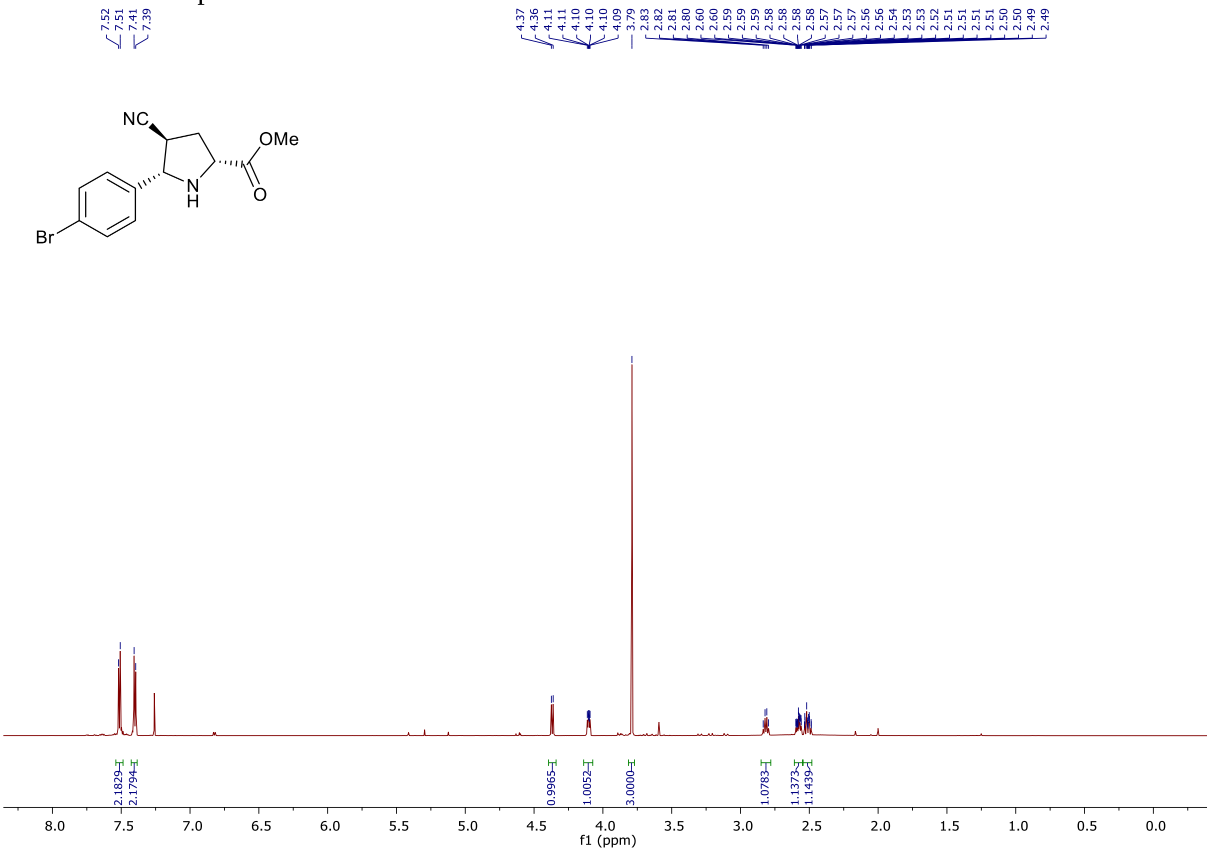

13C NMR of compound A18

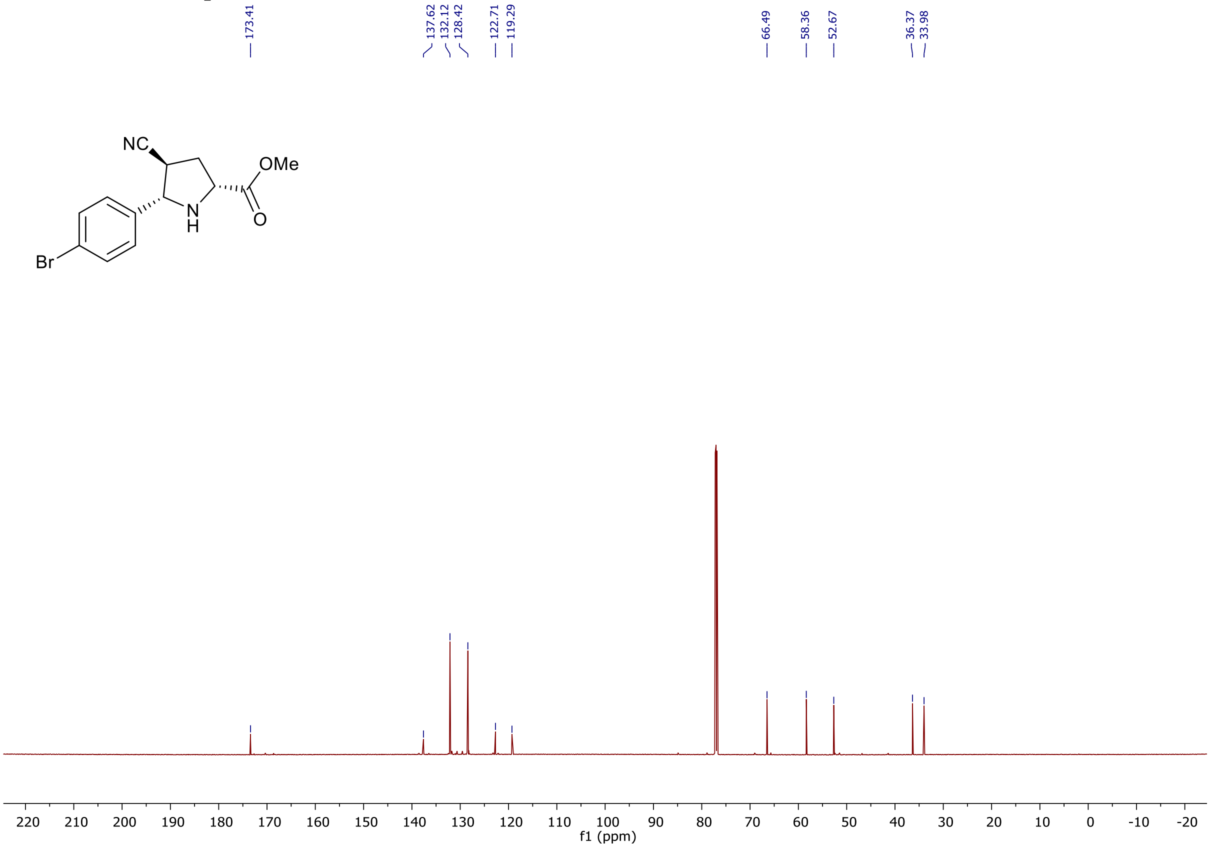

^{1}H NMR of compound A19

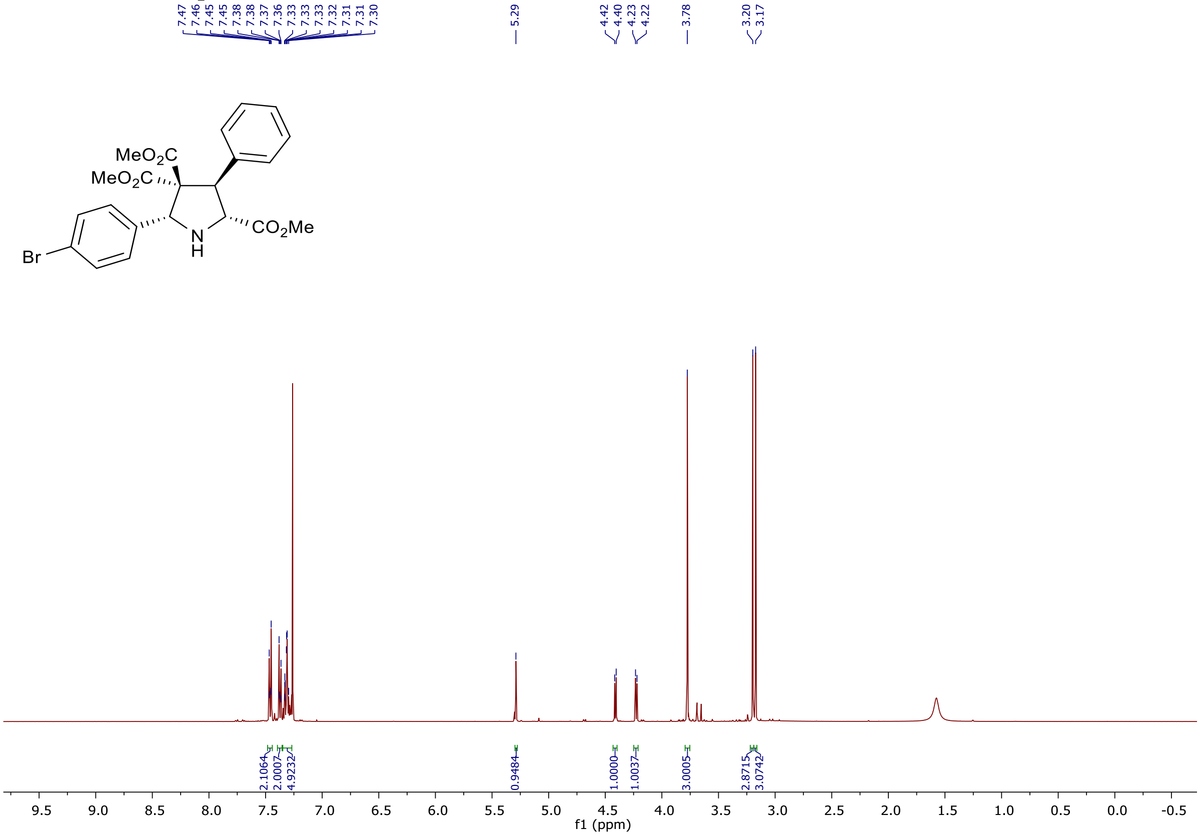

^{13}C NMR of compound A19

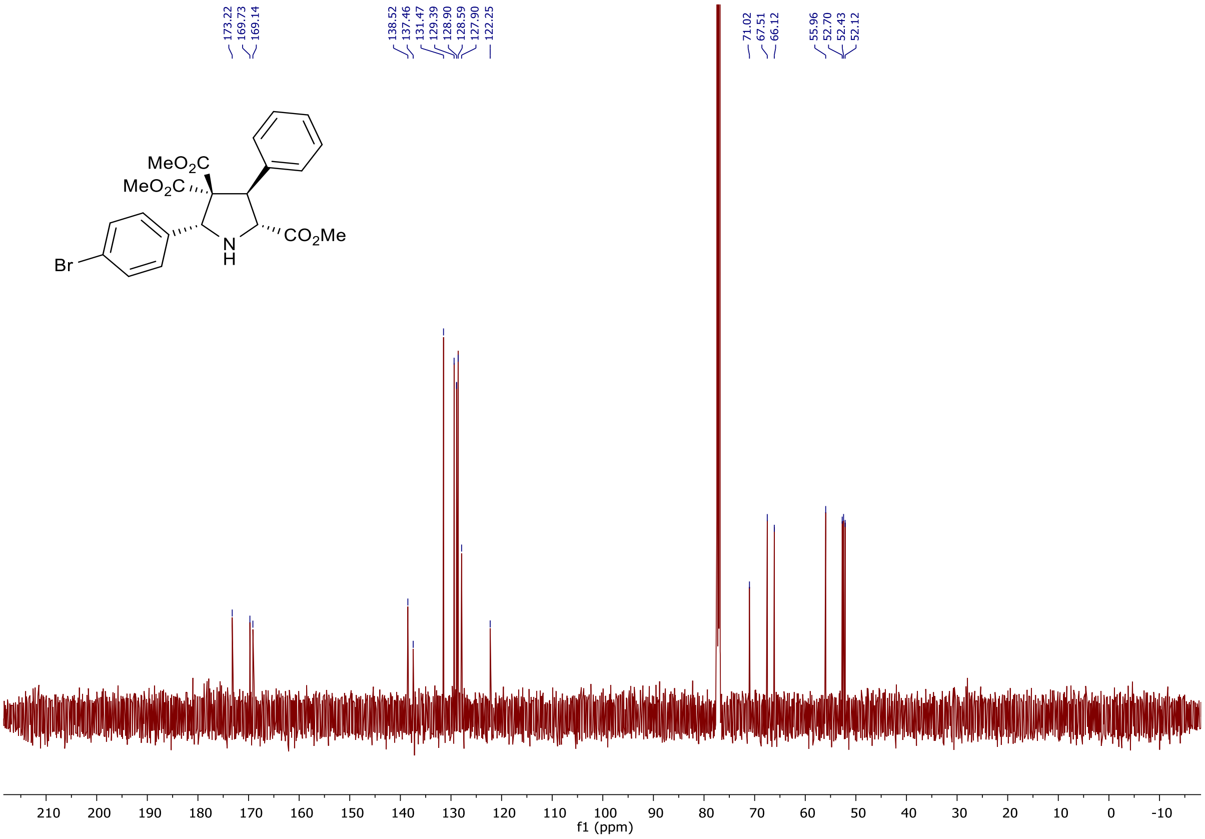

^{1}H NMR of compound A20

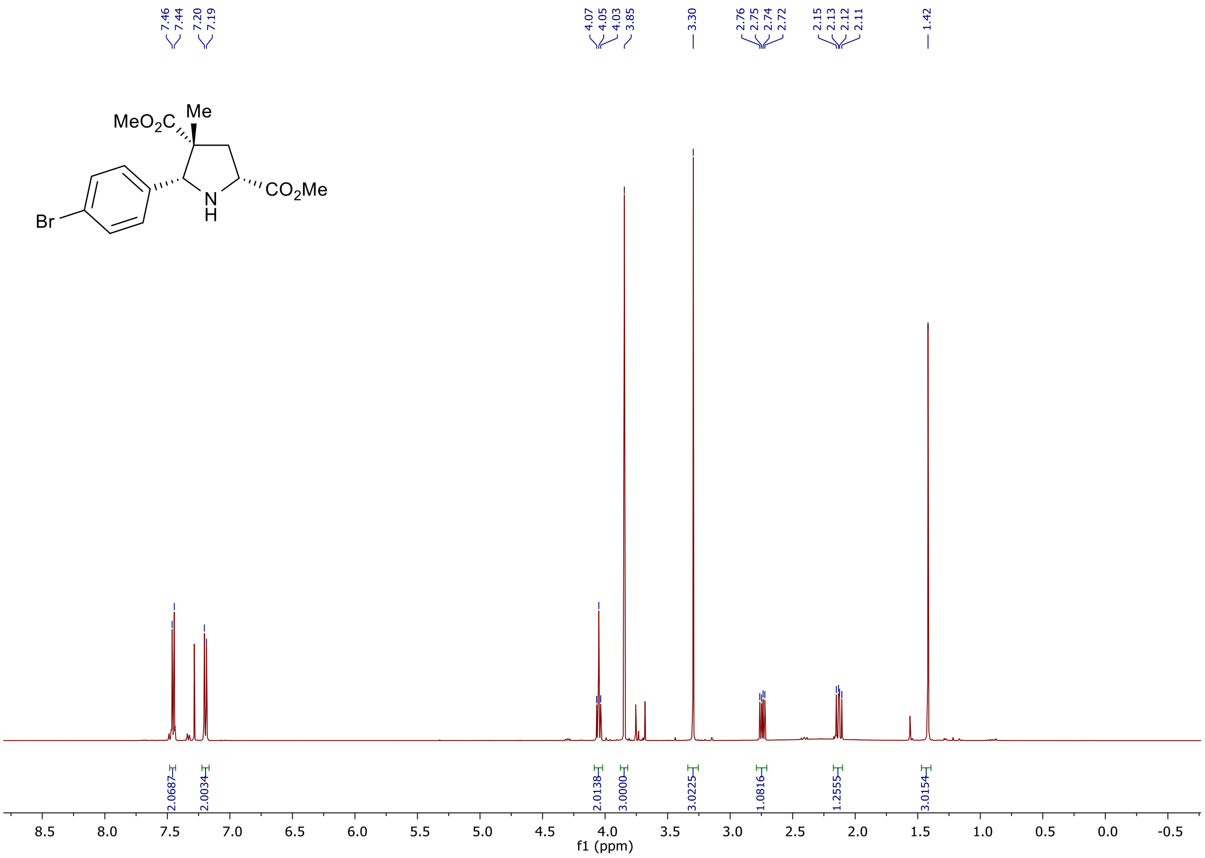

^{13}C NMR of compound A20

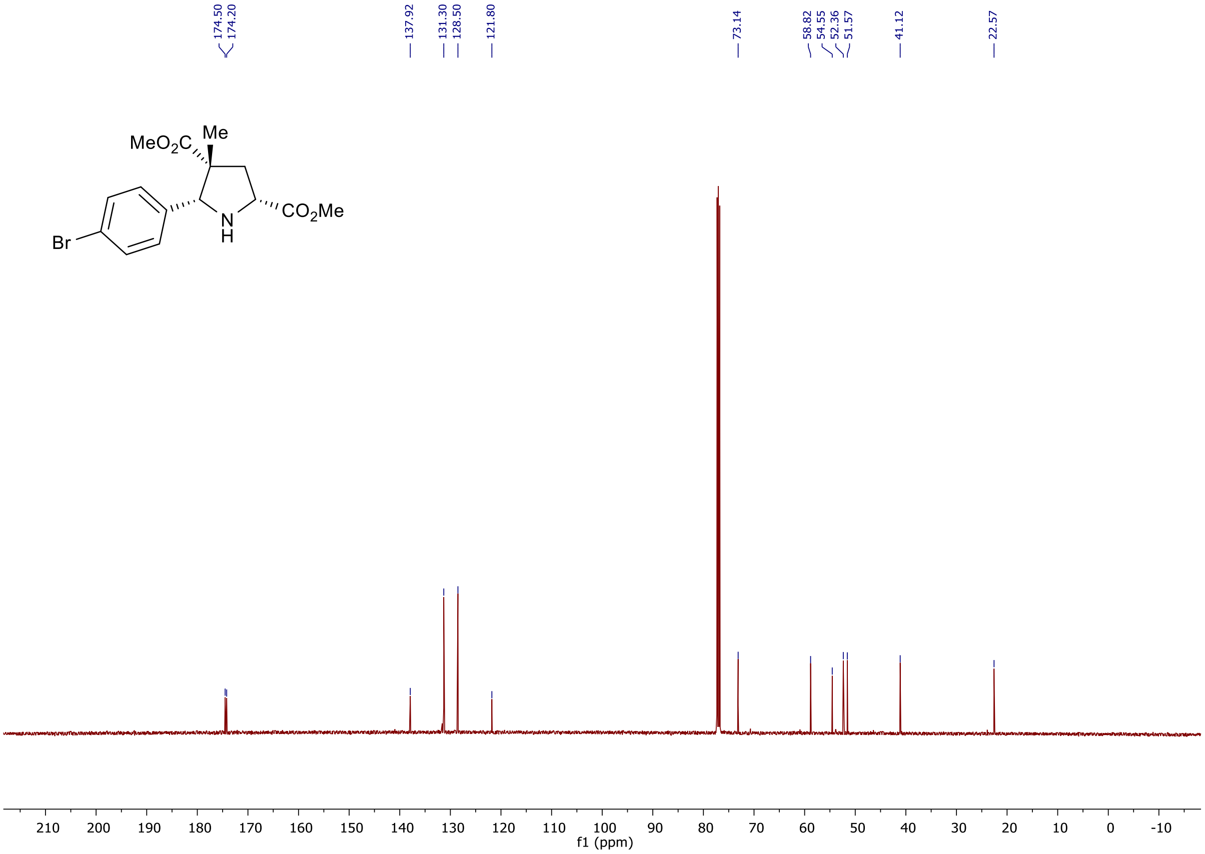

^{1}H NMR of compound A21

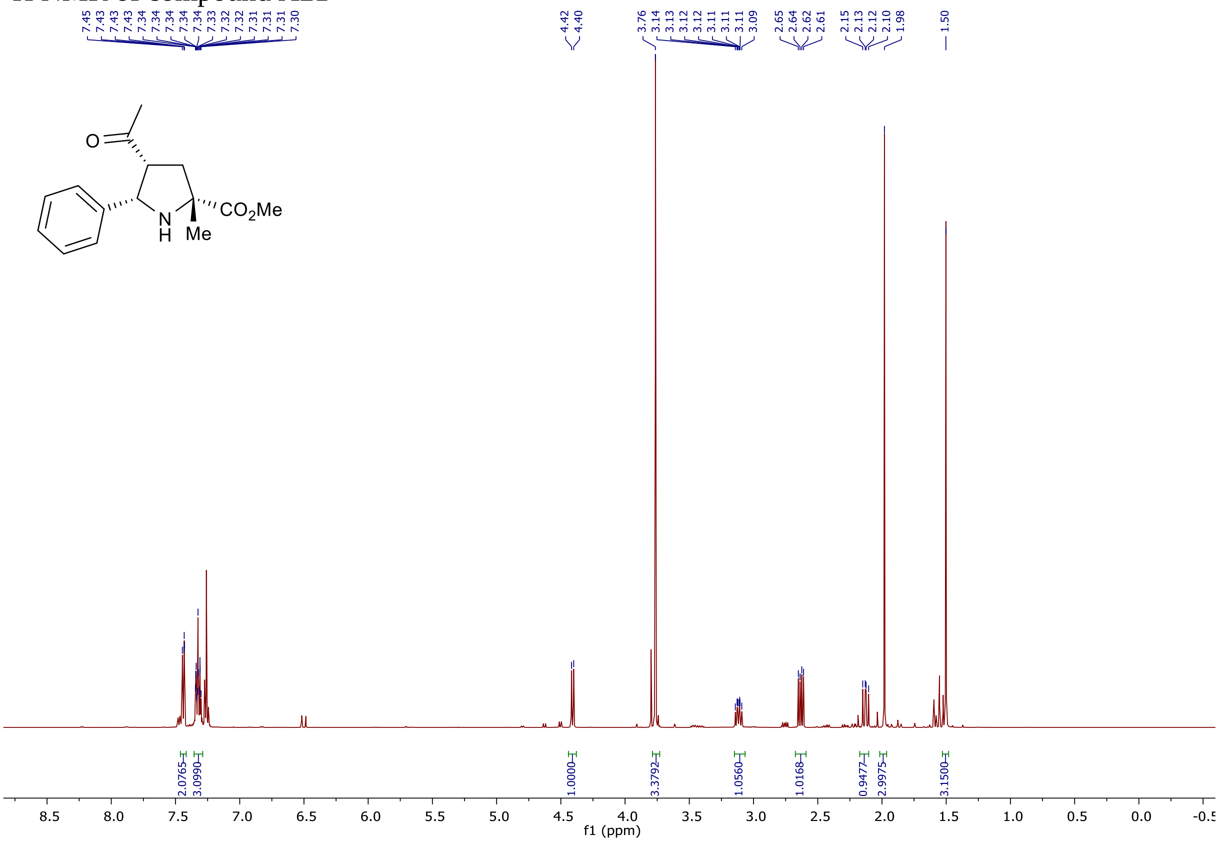

^{13}C NMR of compound A21

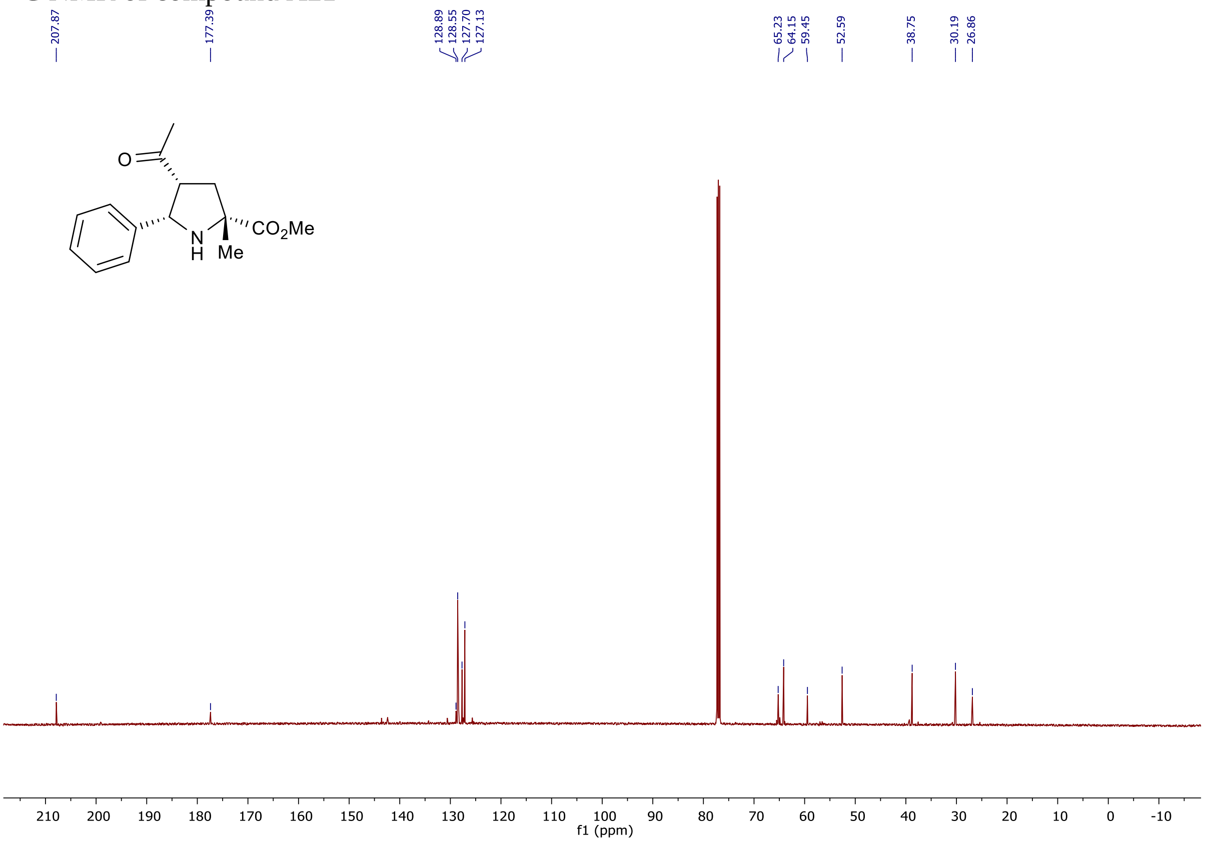

^{1}H NMR of compound A22

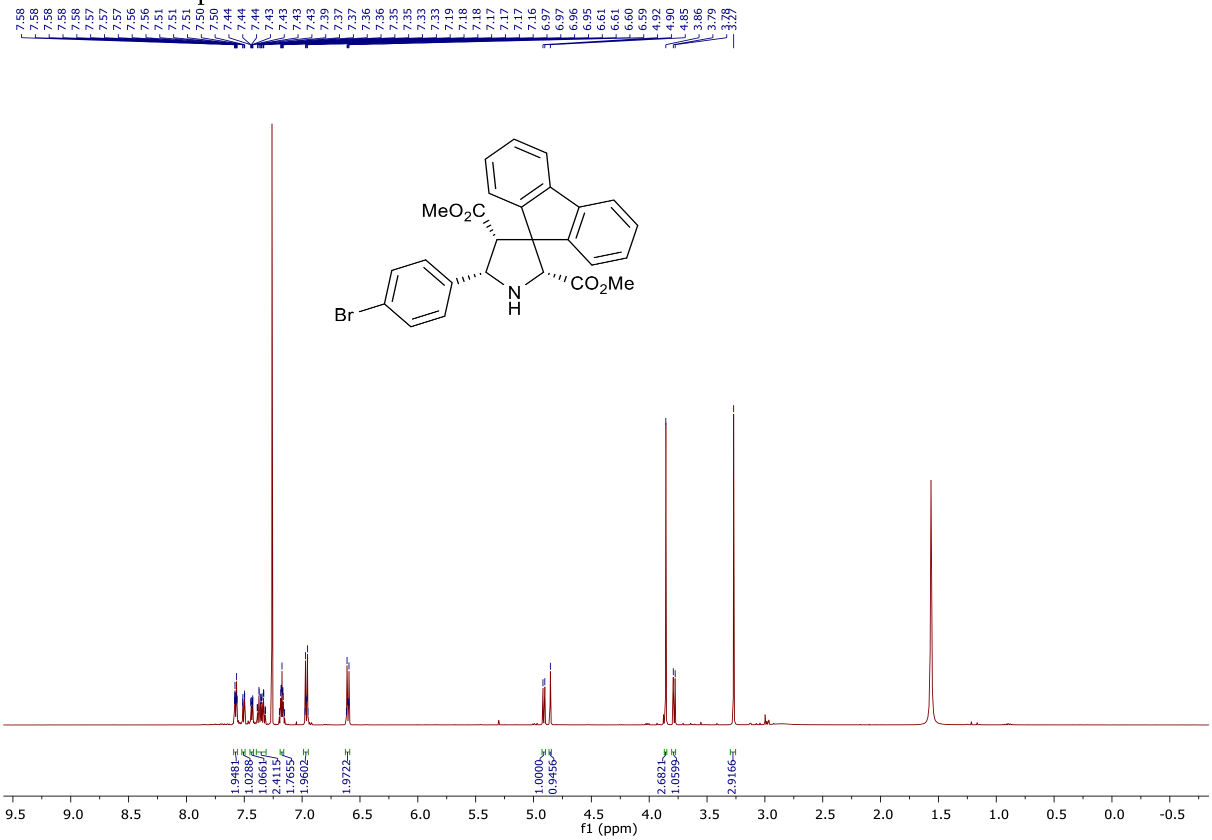

^{13}C NMR of compound A22

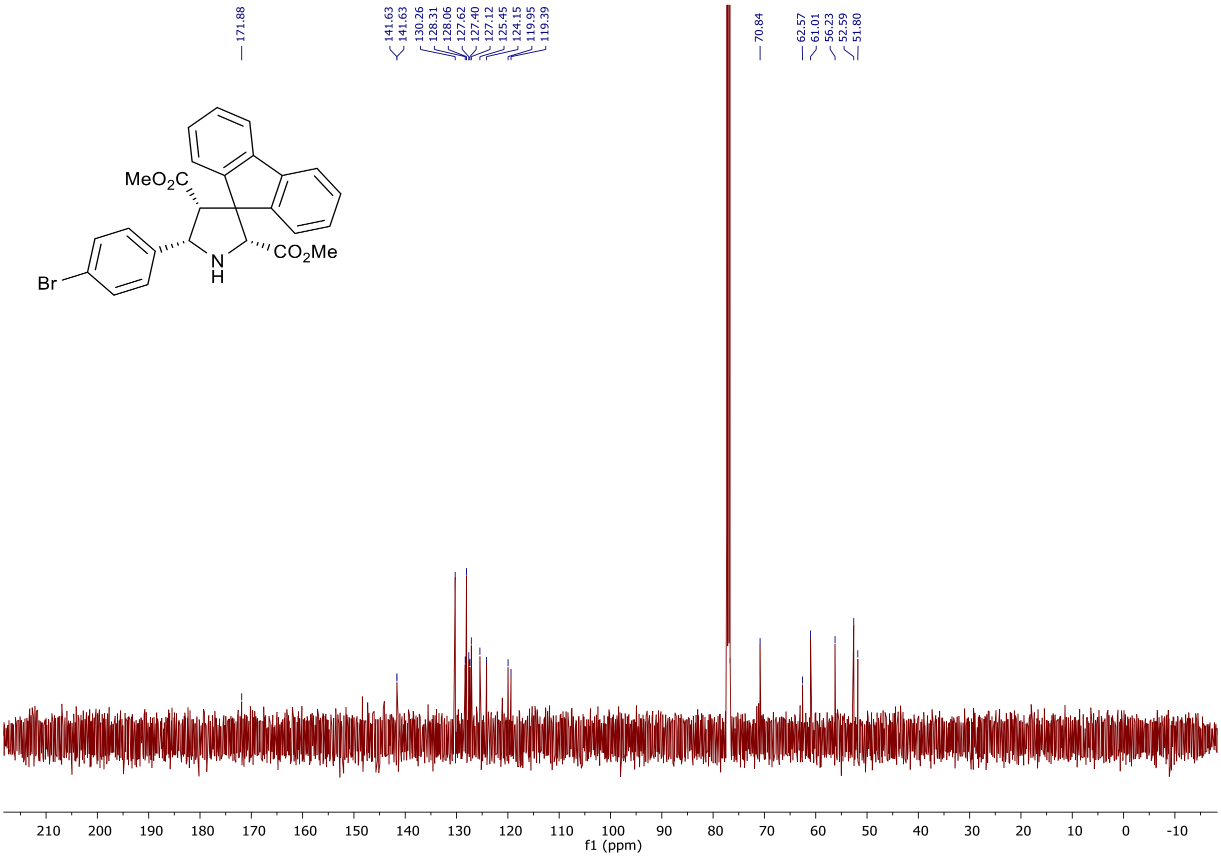

^{1}H NMR of compound A23

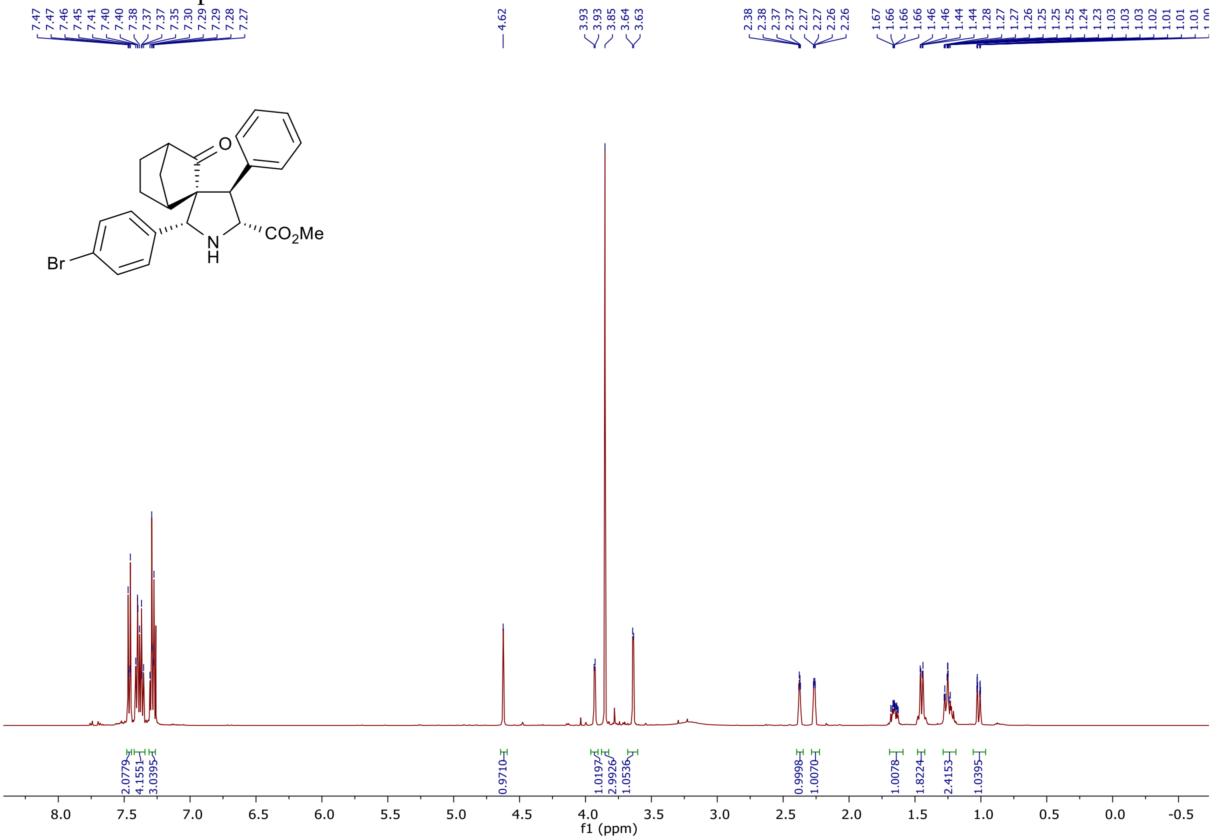

^{13}C NMR of compound S23

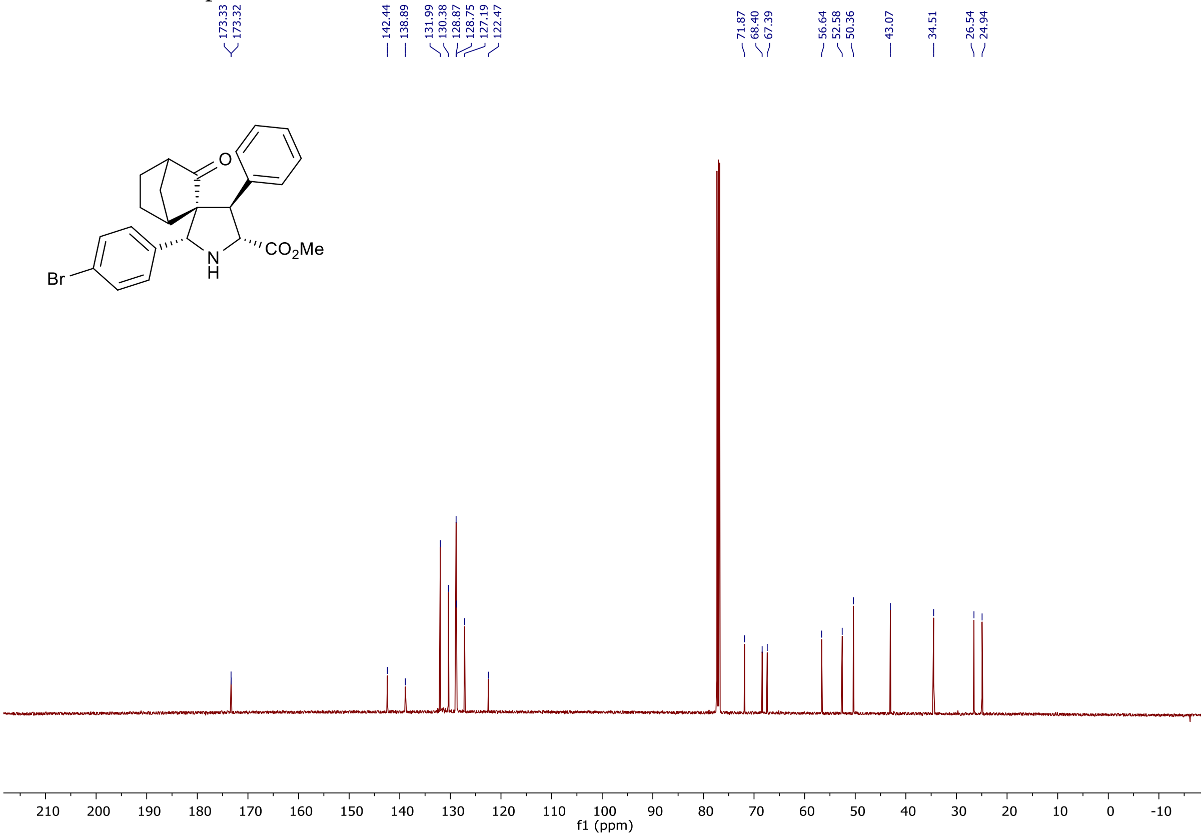

^{1}H NMR of compound A24

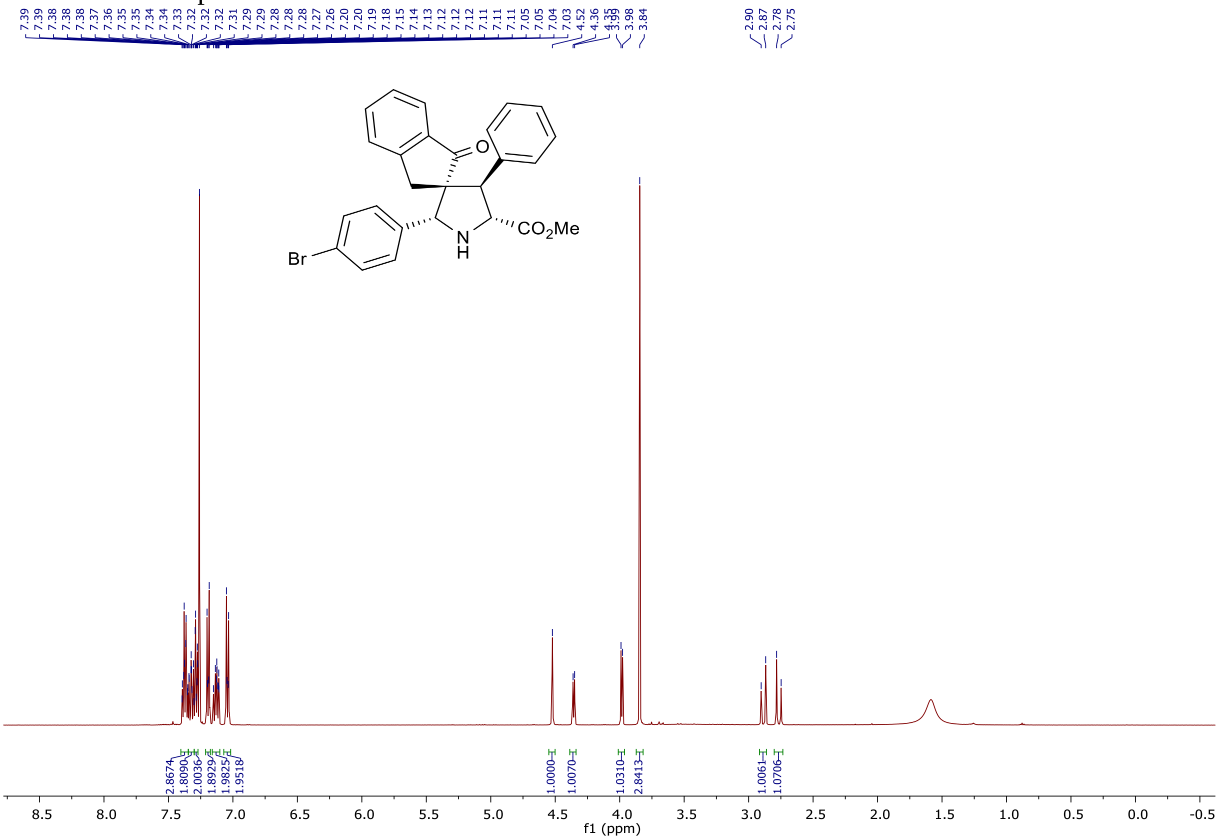

^{13}C NMR of compound A24

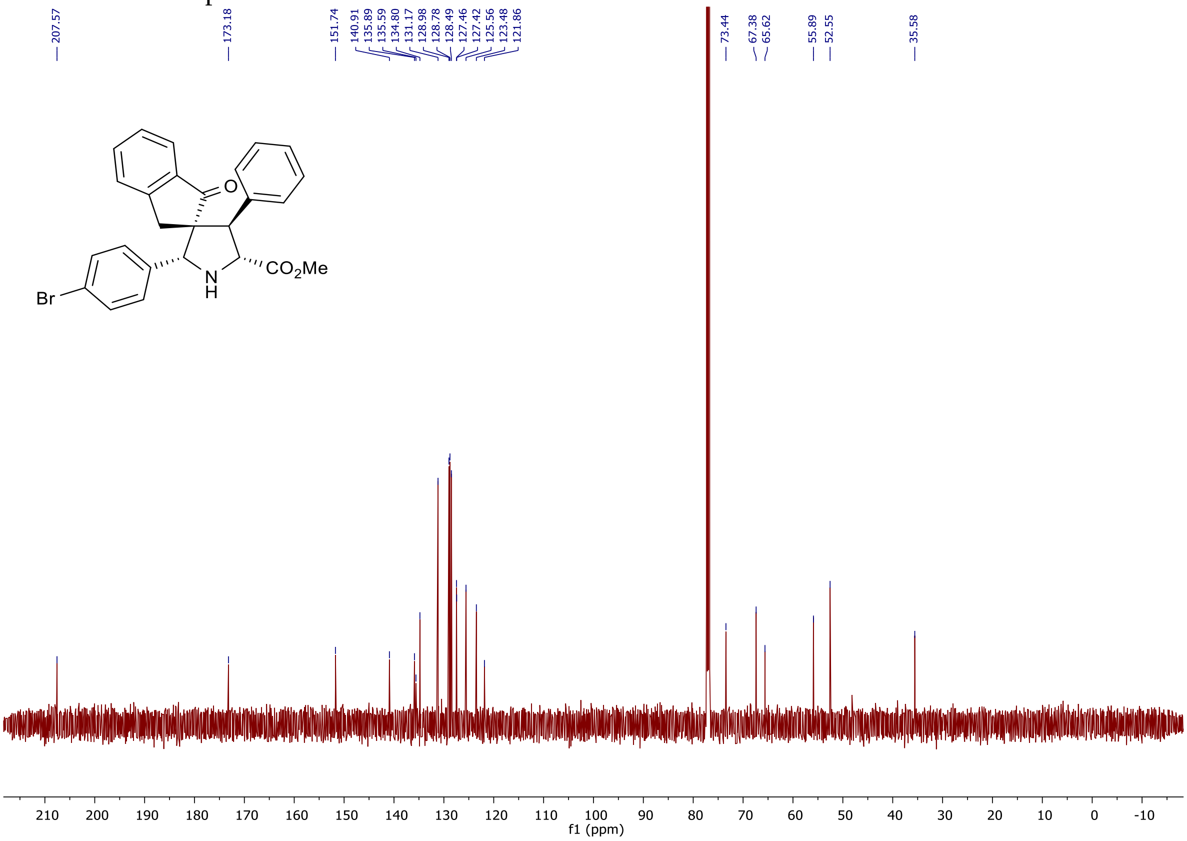

¹H NMR of compound A25

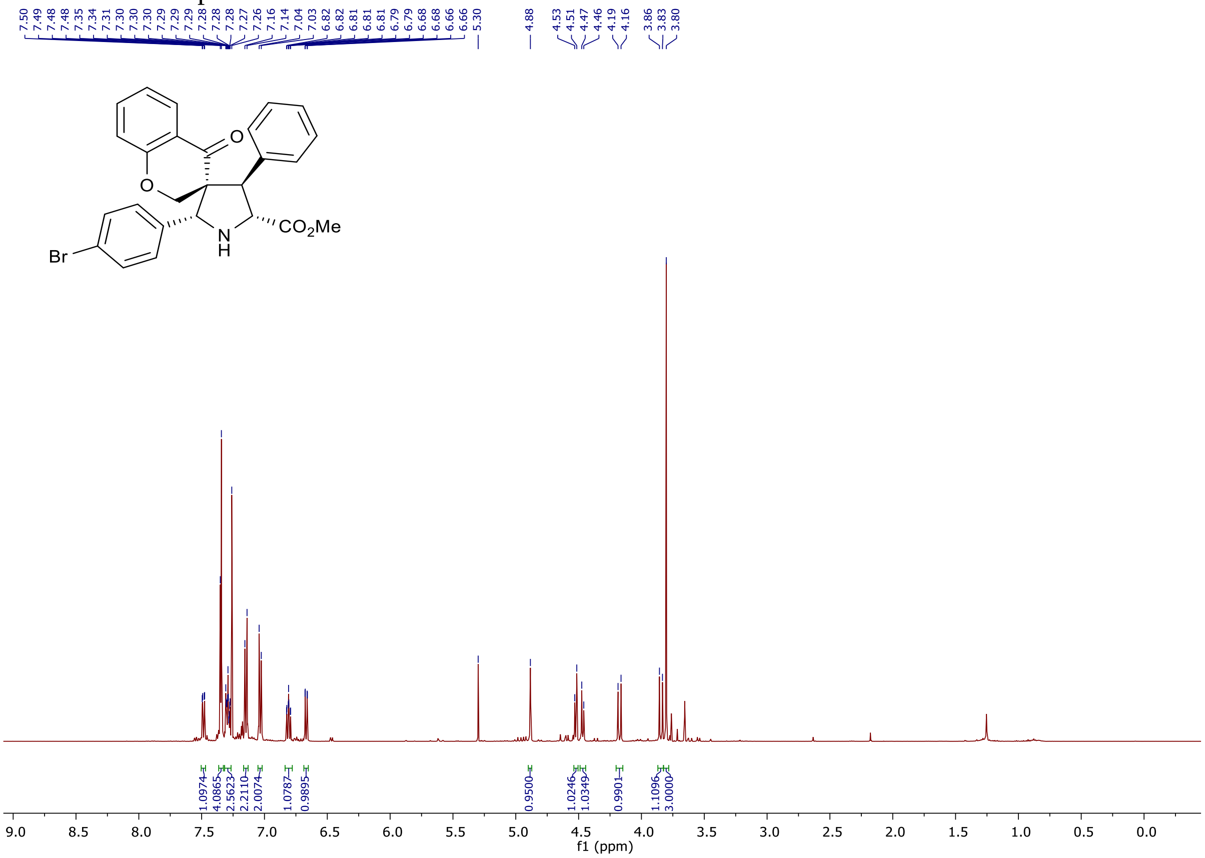

¹H NMR of compound A25

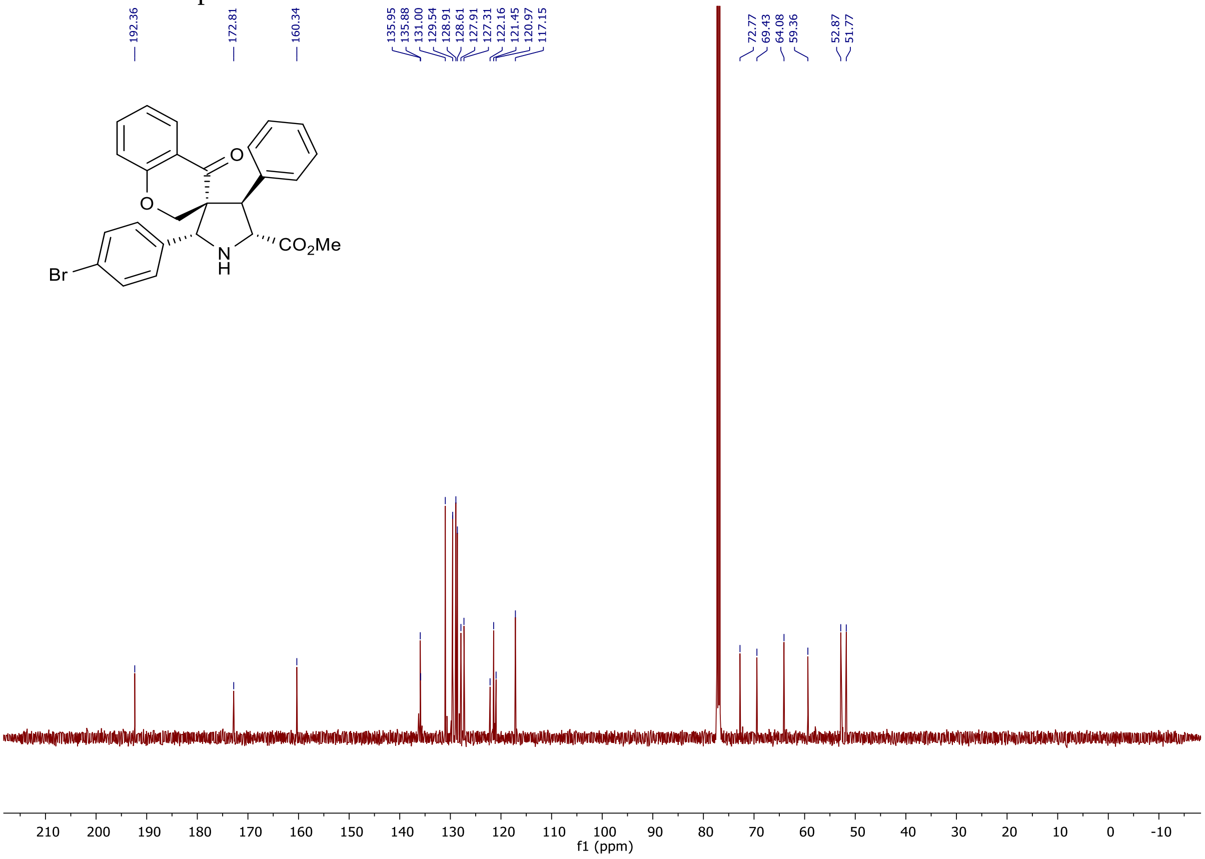

^{1}H NMR of compound A26

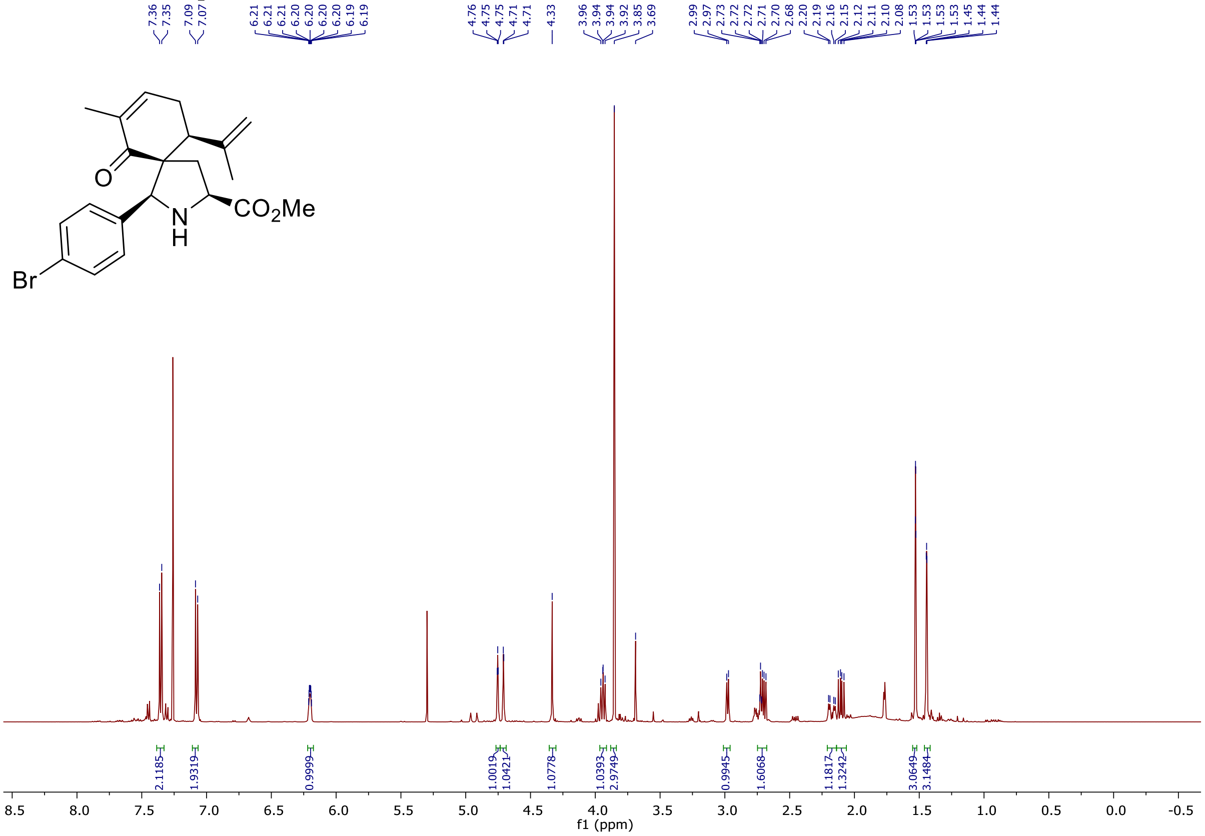

^{13}C NMR of compound A26

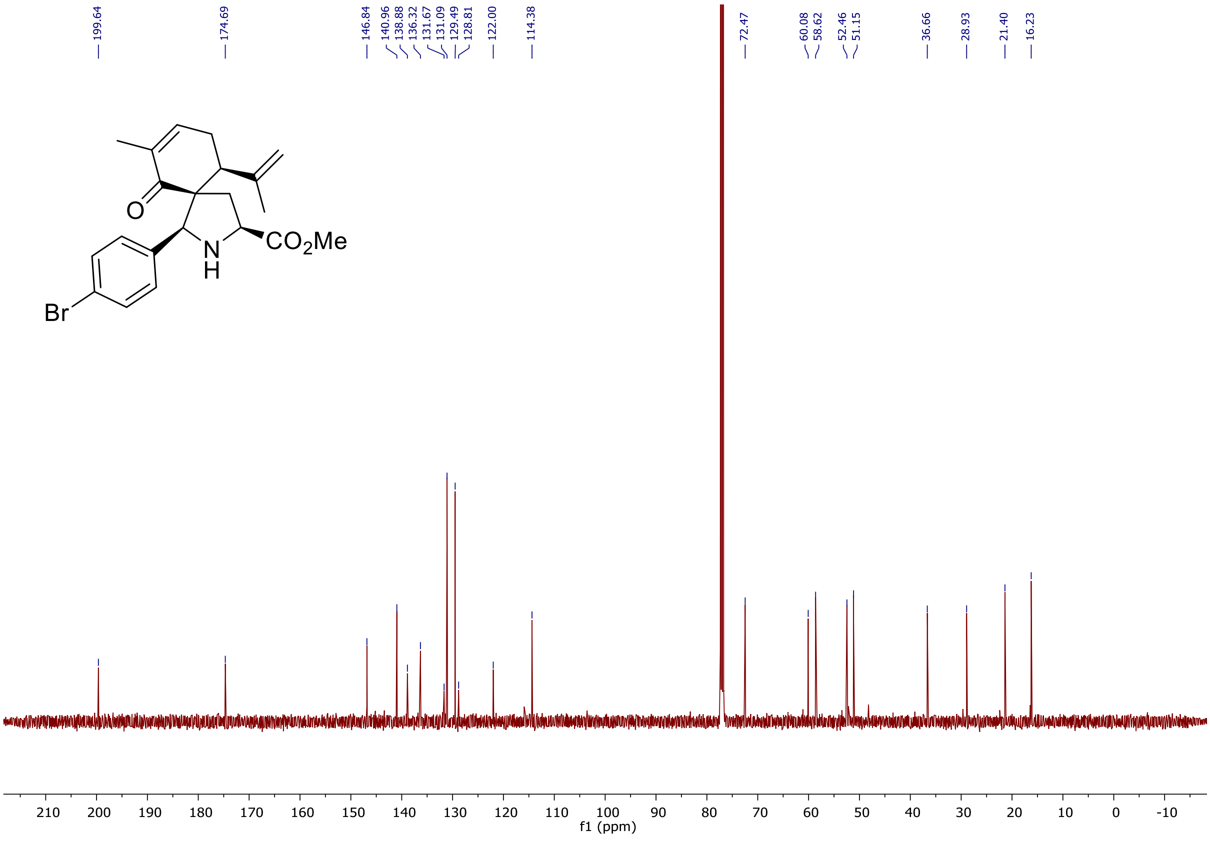

^{1}H NMR of compound A27

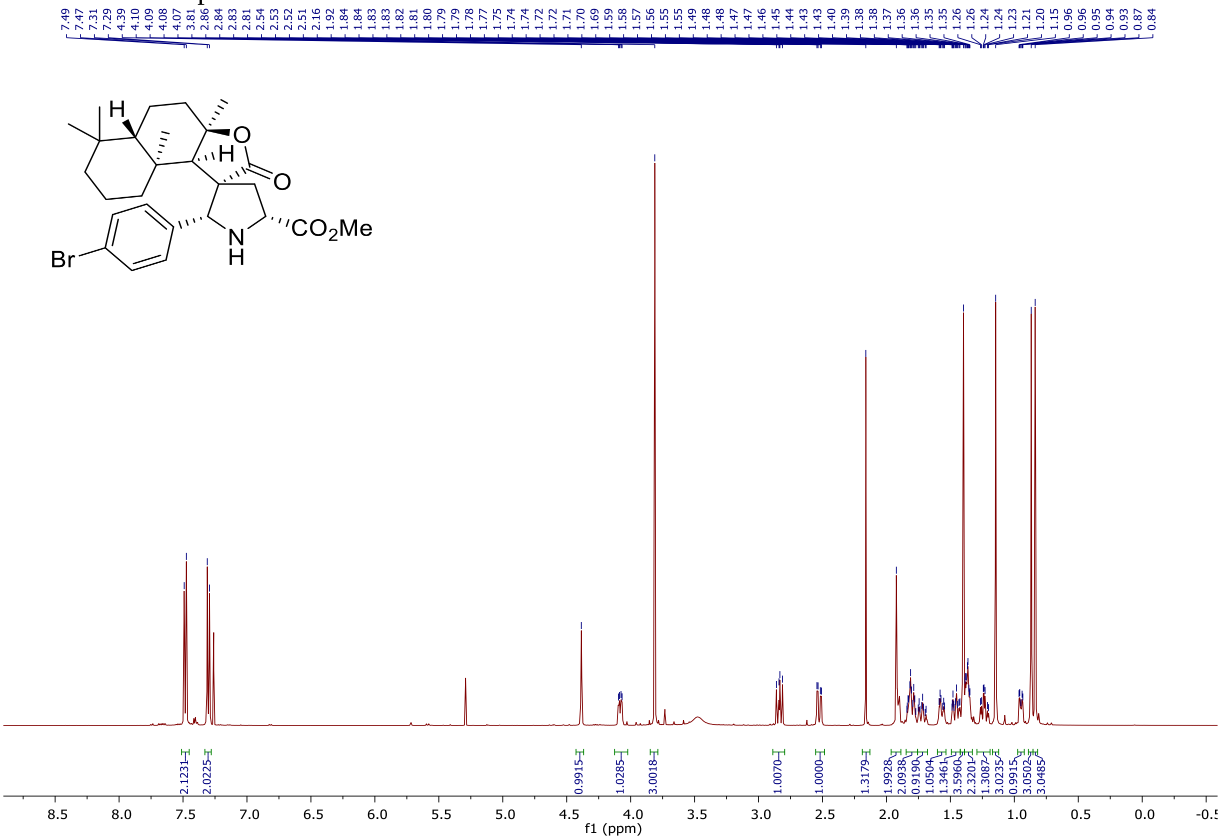

^{13}C NMR of compound A27

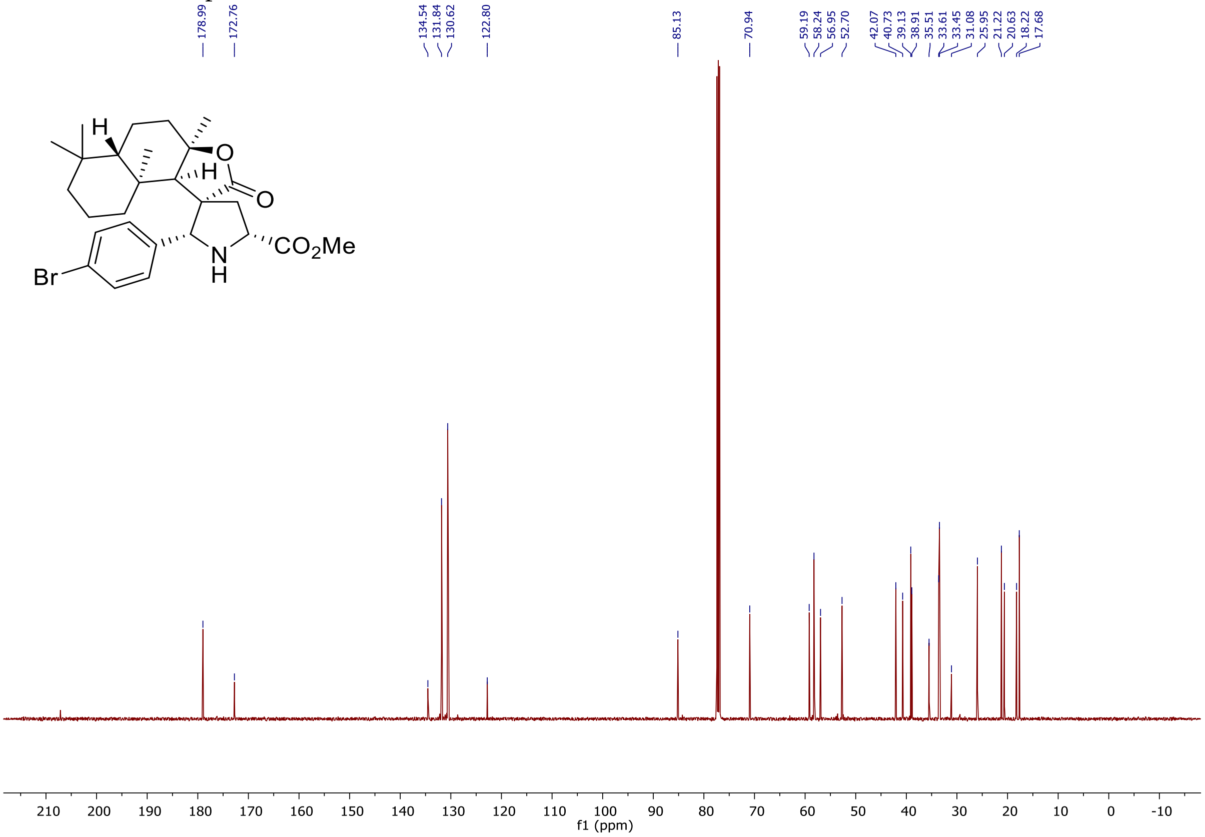

^{1}H NMR of compound A28

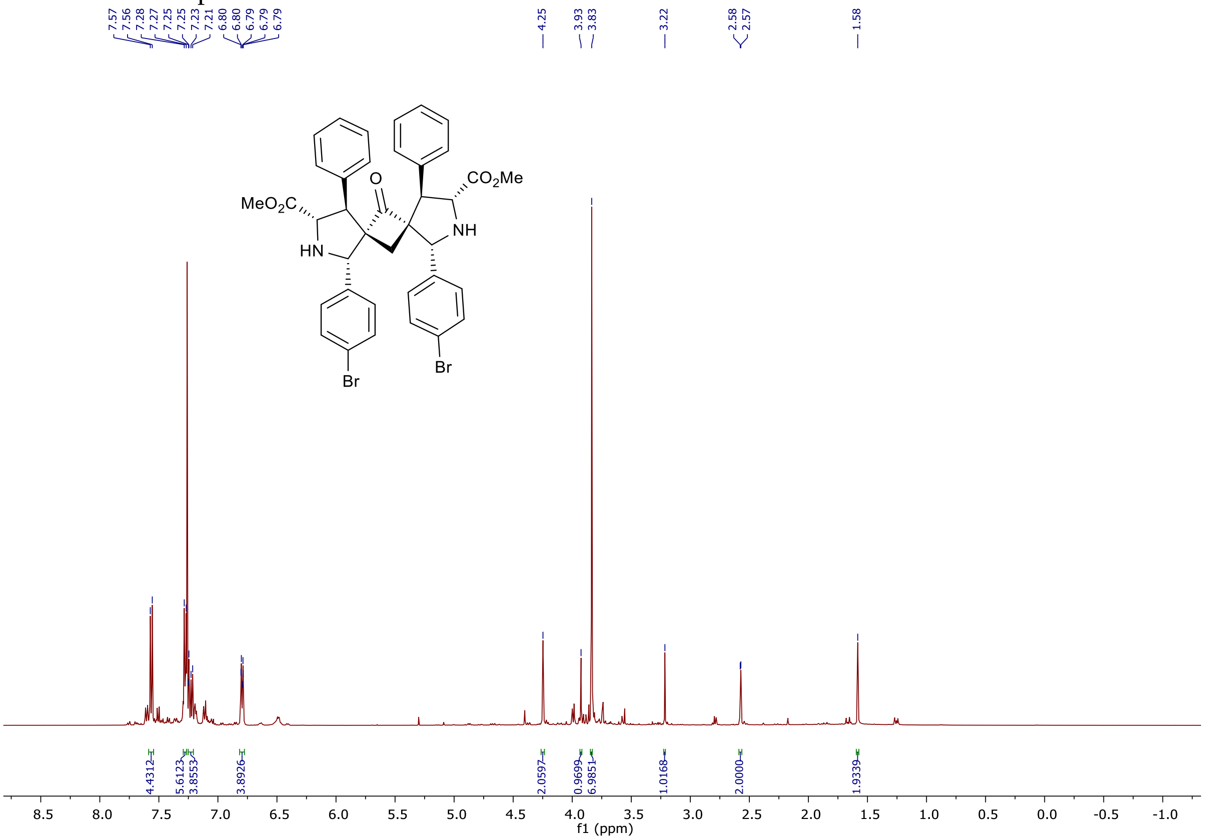

^{13}C NMR of compound A28

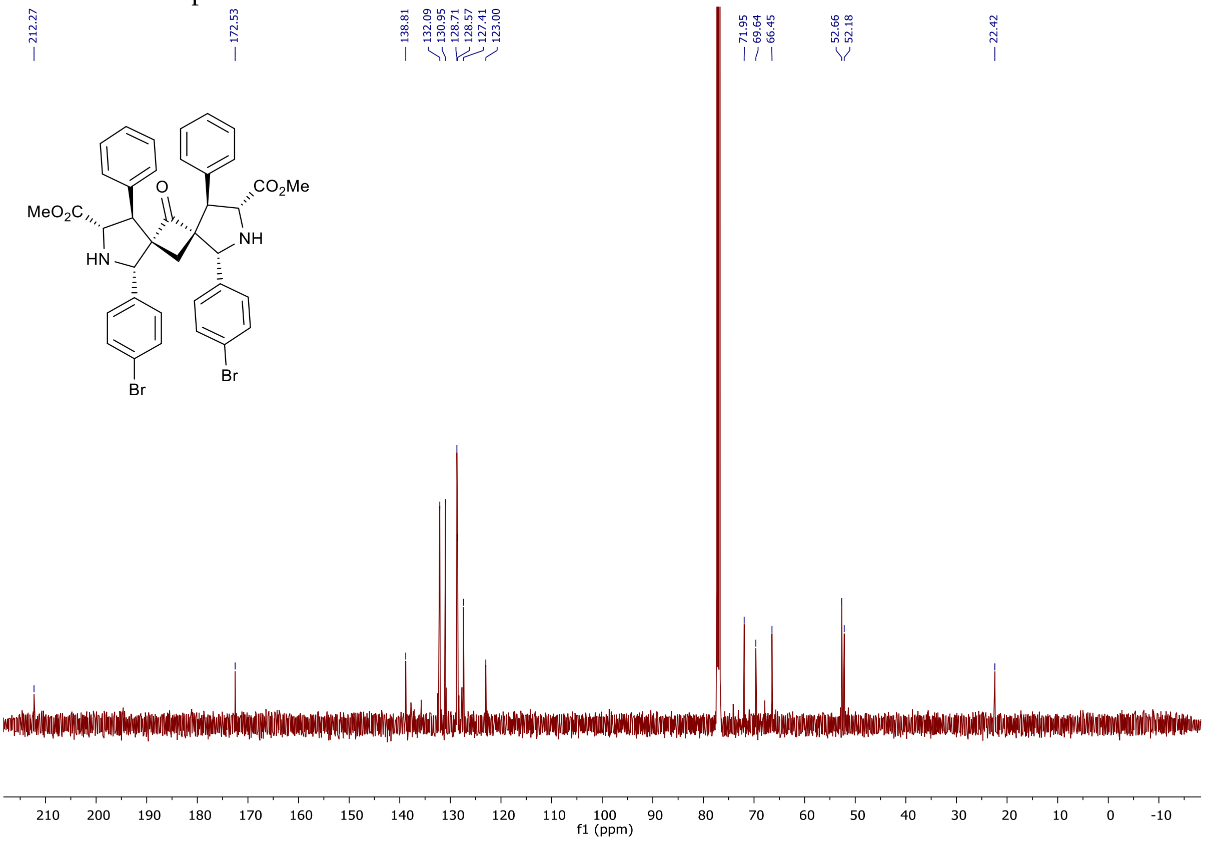

^{1}H NMR of compound A29

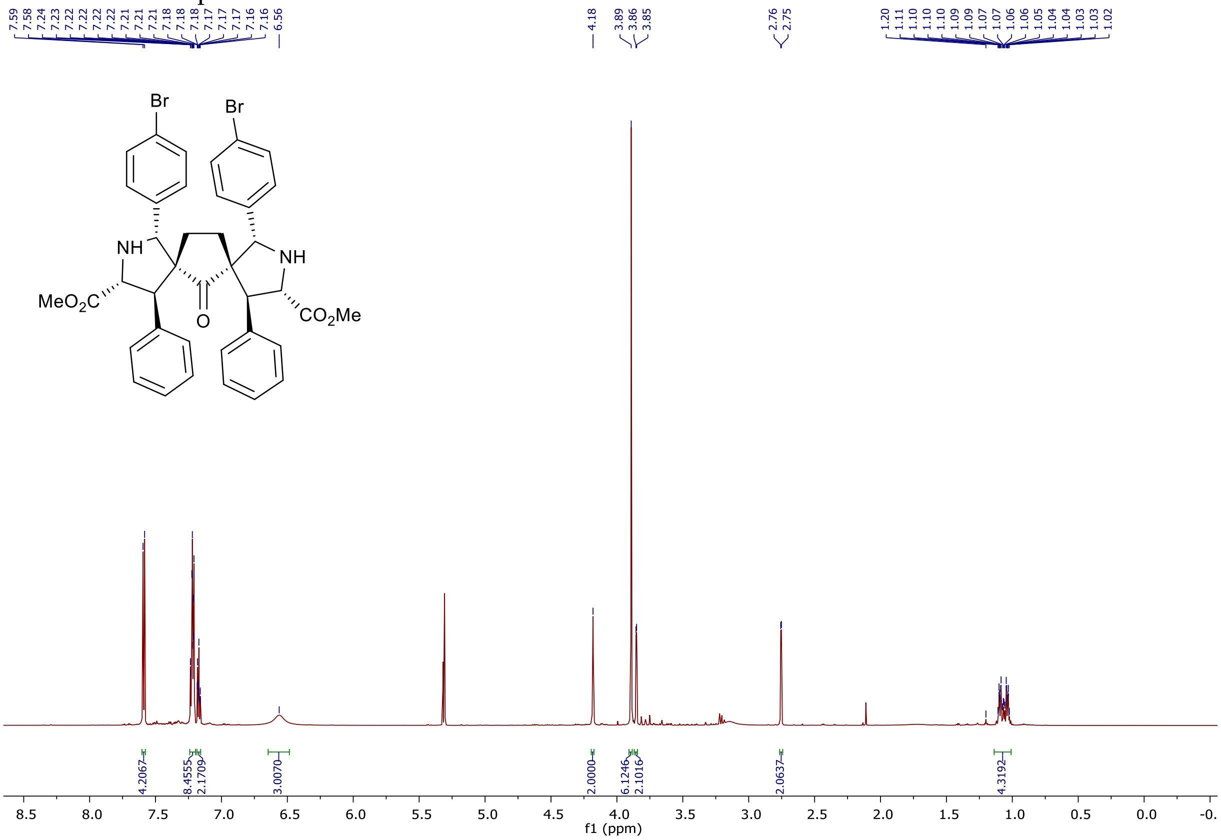

^{13}C NMR of compound A29

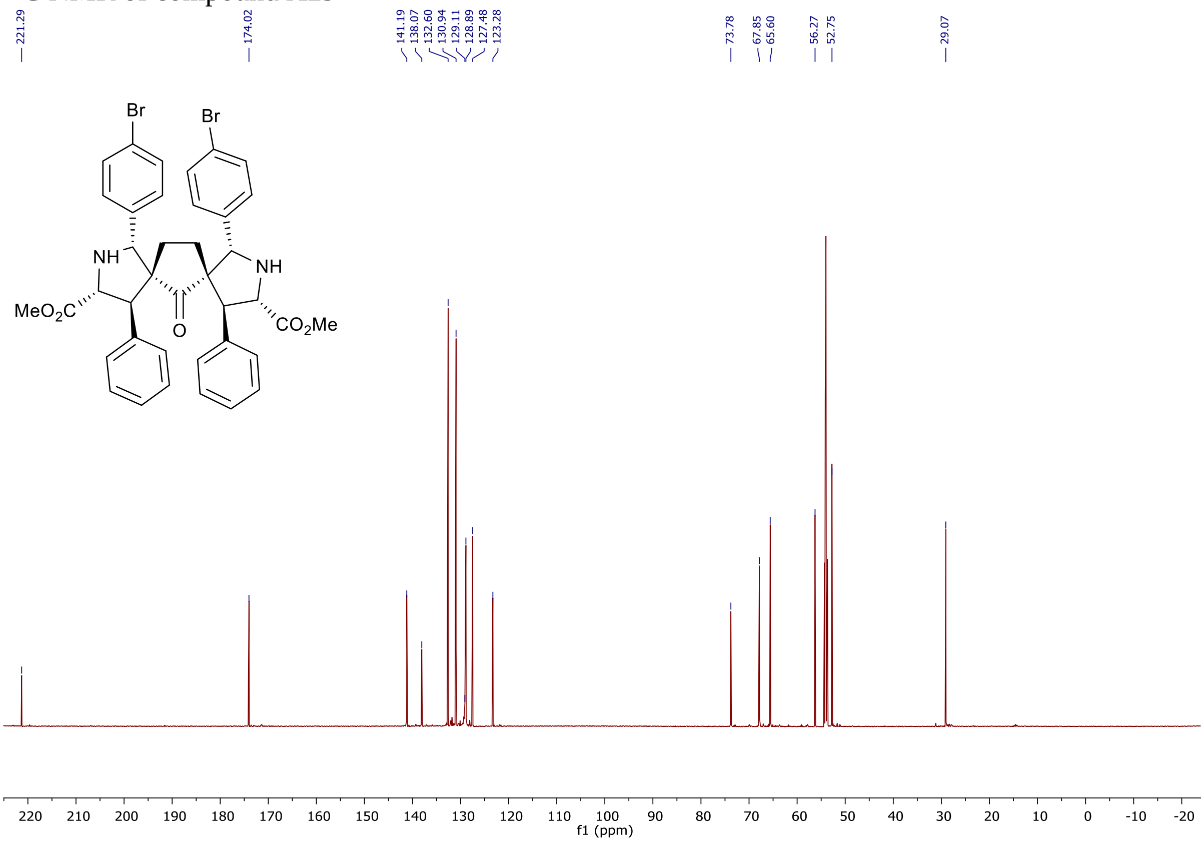

1H NMR of compound *cis*-47

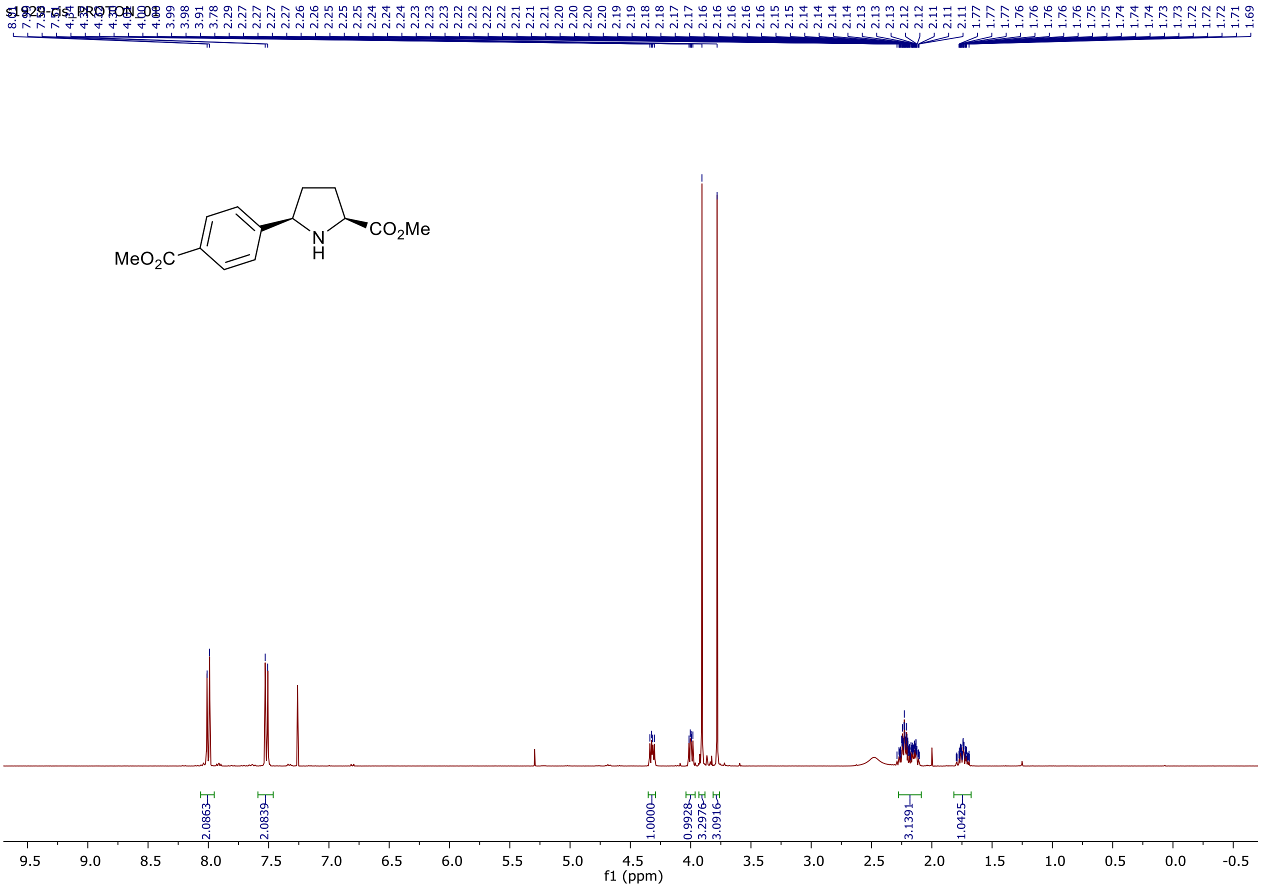

13C NMR of compound *cis*-47

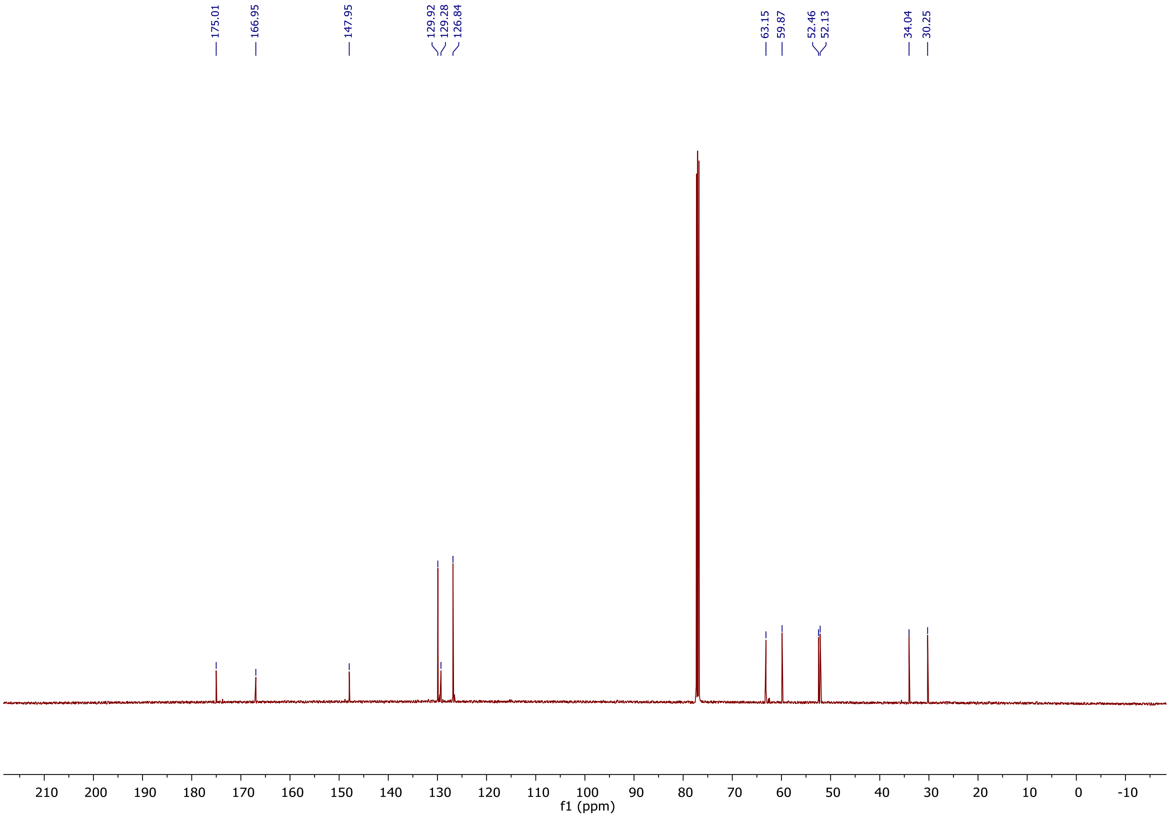

1H NMR of compound *trans*-49

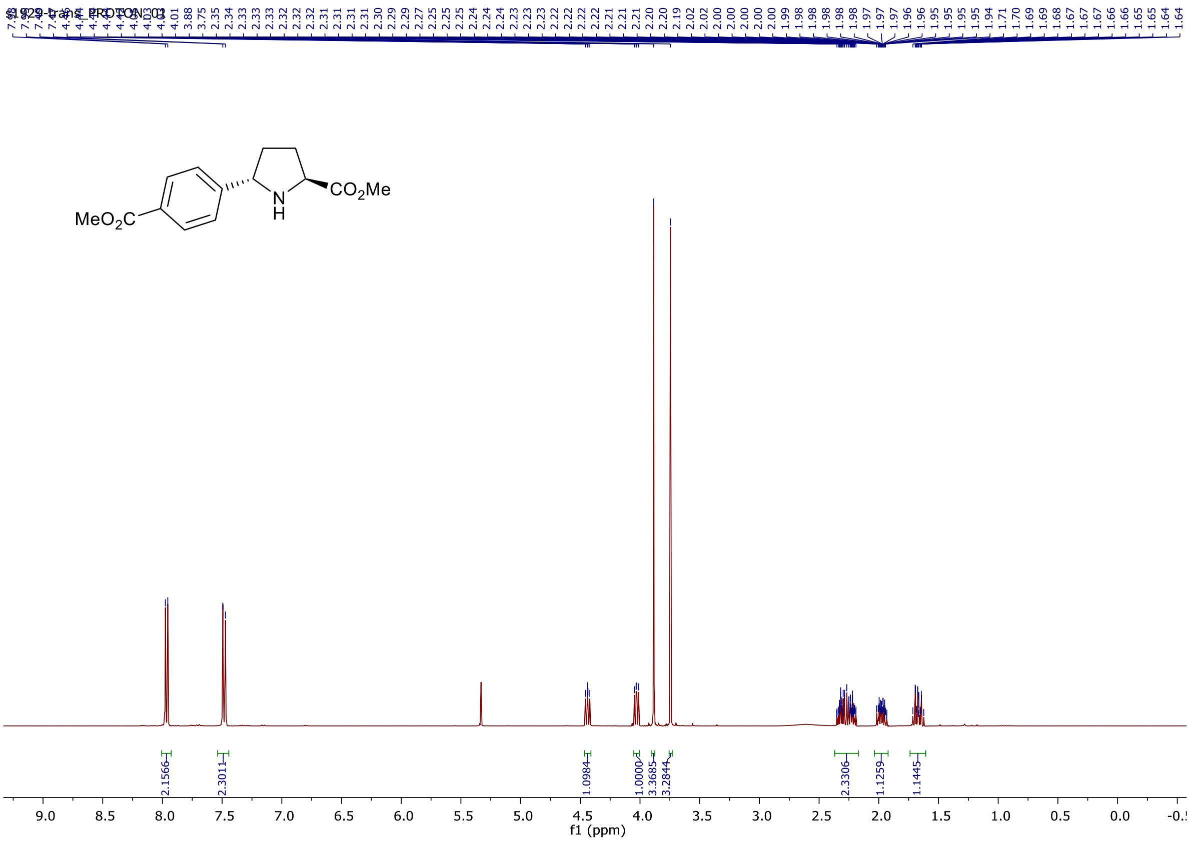

13C NMR of compound *trans*-49

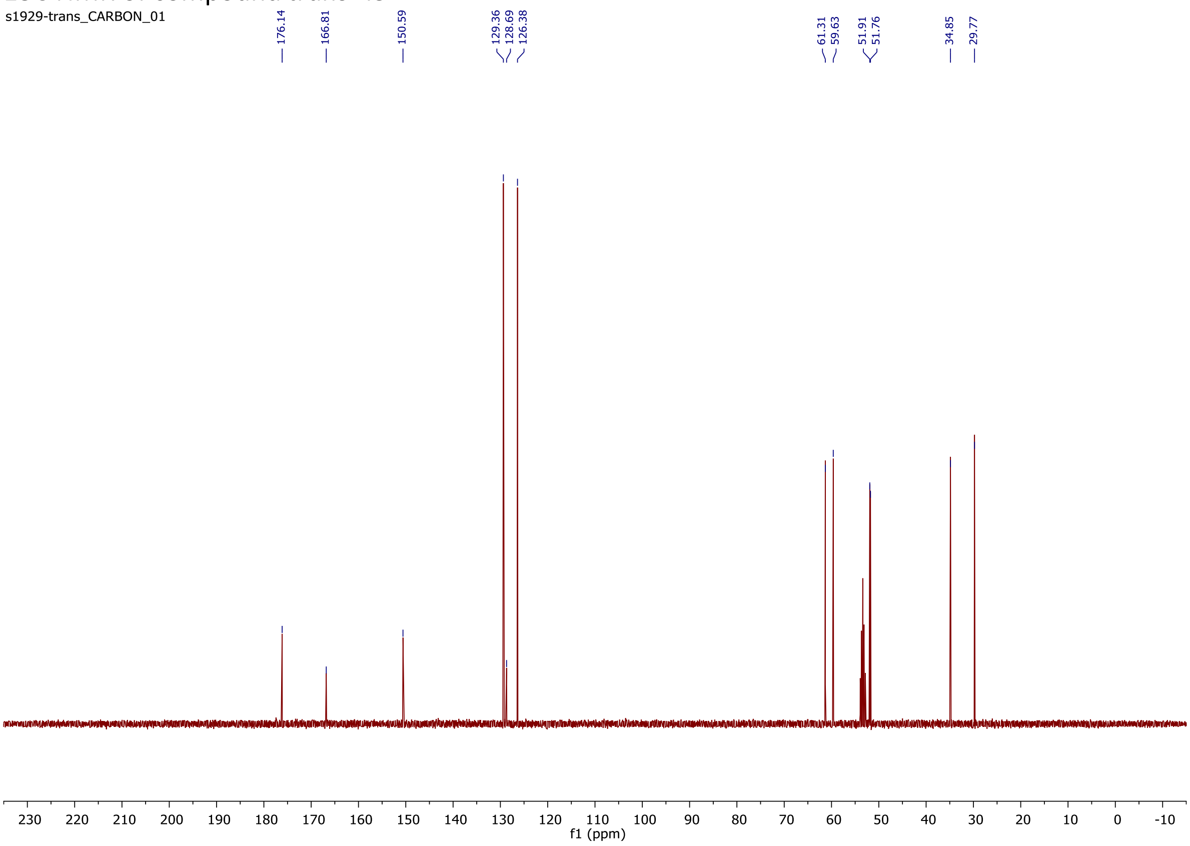

^{1}H NMR of compound 7

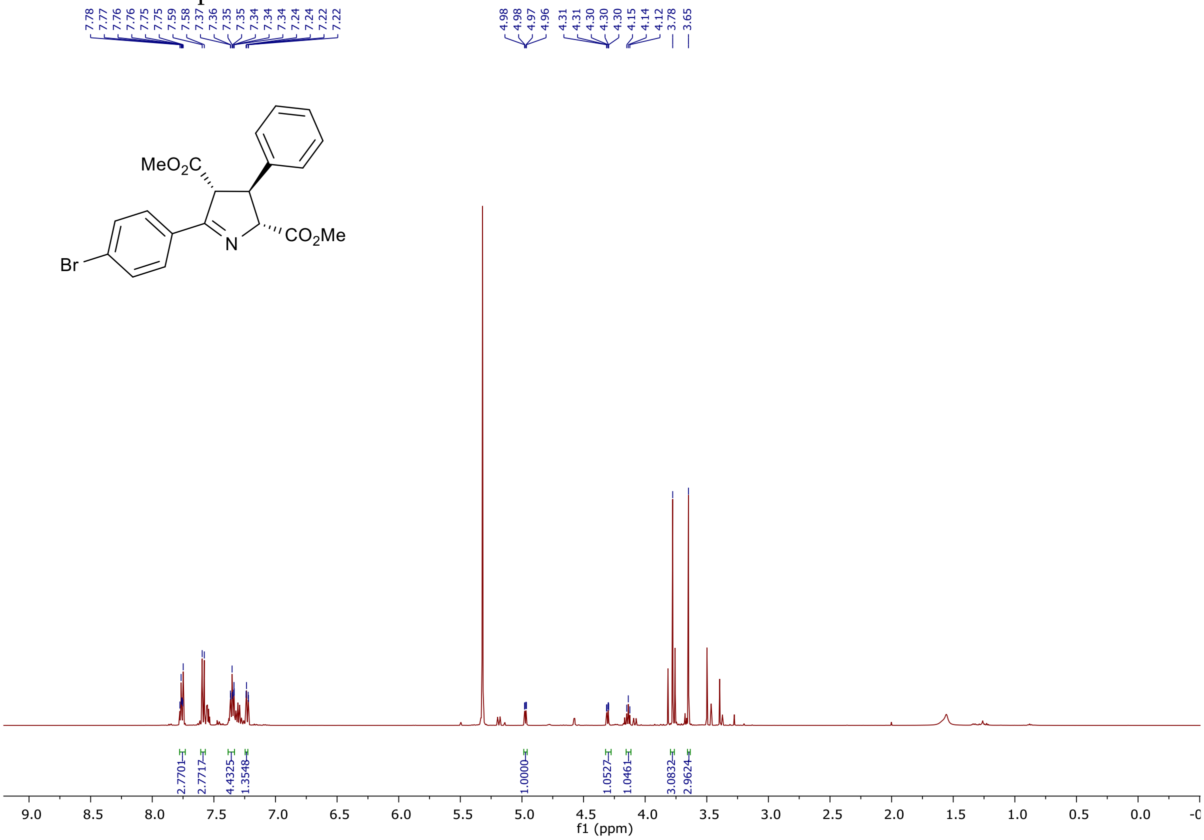

^{13}C NMR of compound 7

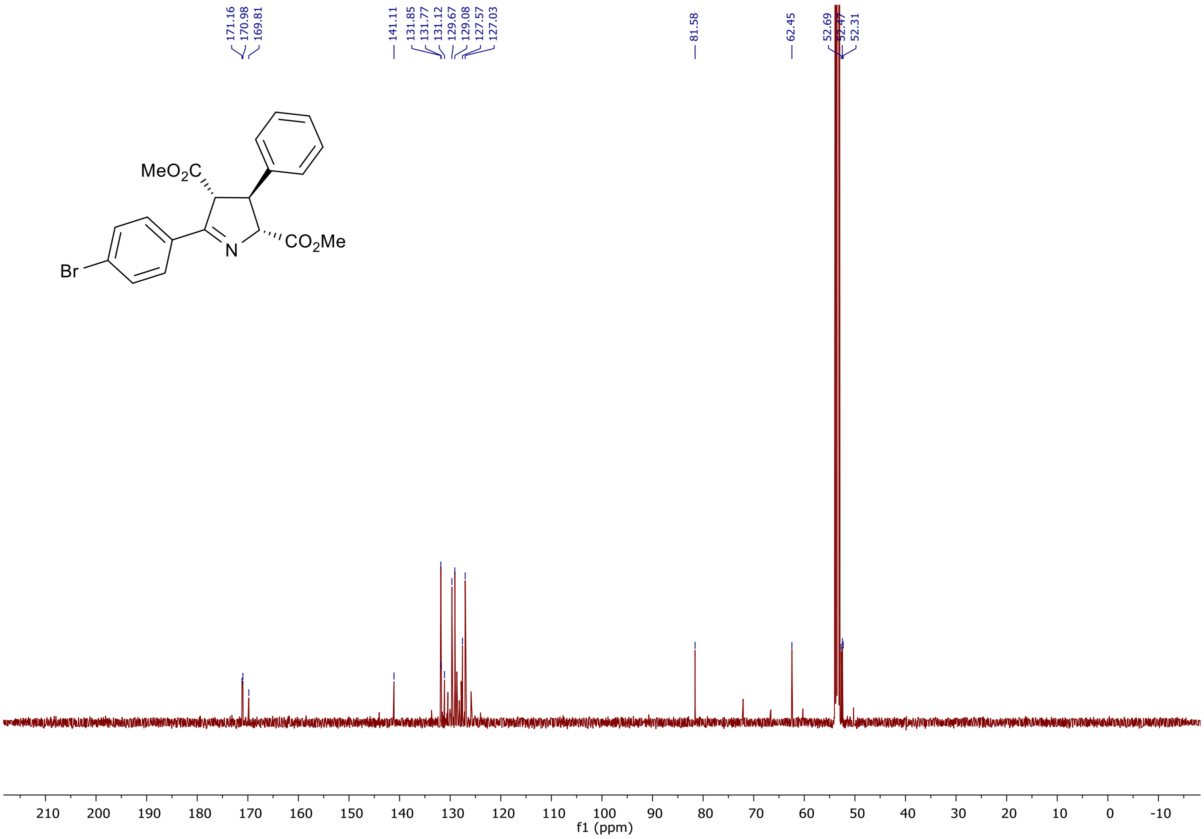

^{1}H NMR of compound 54

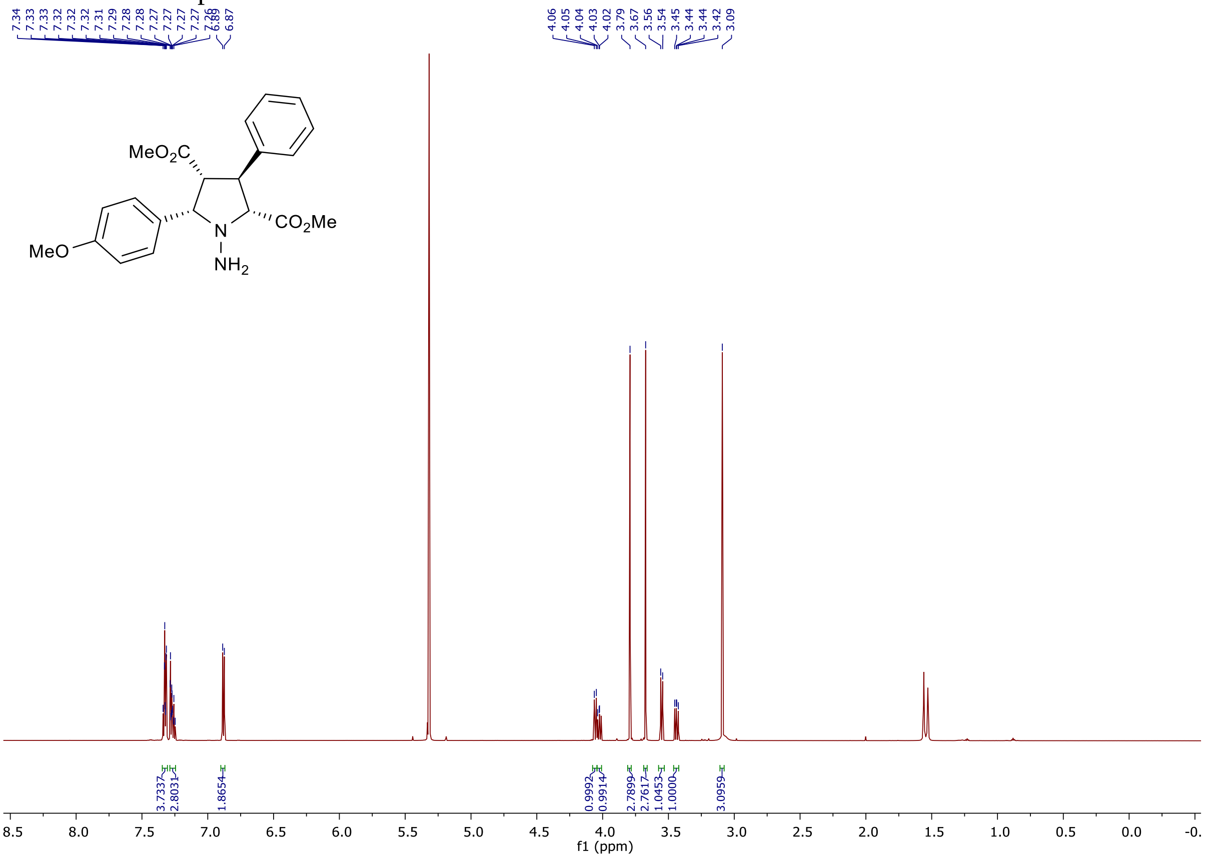

^{13}C NMR of compound 54

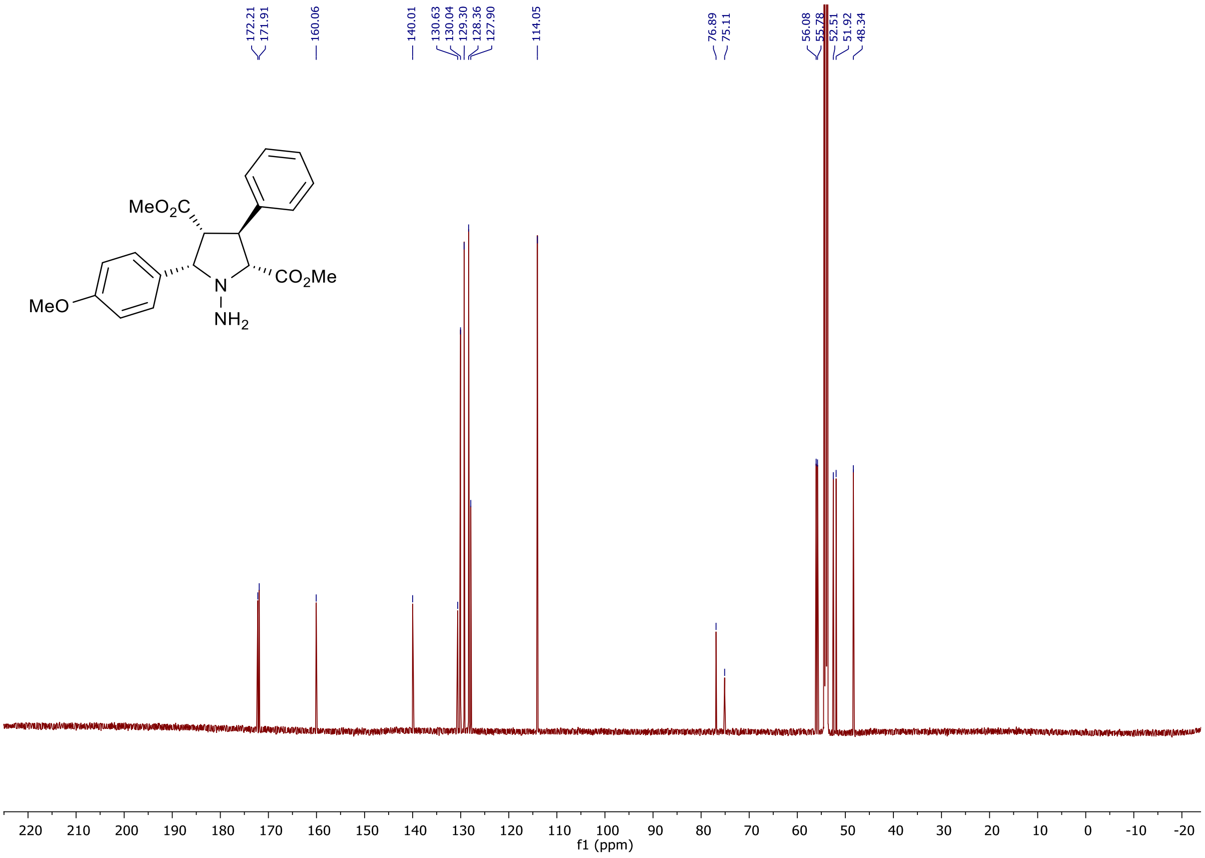

Supplement: Supplementary file 1 — ja1c10175_si_001.pdf [file ja1c10175_si_001.pdf]
